# Maternal complications following open and fetoscopic fetal surgery: A systematic review and meta‐analysis

**DOI:** 10.1002/pd.5421

**Published:** 2019-02-27

**Authors:** Adalina Sacco, Lennart Van der Veeken, Emma Bagshaw, Catherine Ferguson, Tim Van Mieghem, Anna L. David, Jan Deprest

**Affiliations:** ^1^ Department of Maternal and Fetal Medicine Institute for Women's Health, University College London London UK; ^2^ Department of Development and Regeneration, Cluster Woman and Child, Biomedical Sciences KU Leuven Leuven Belgium; ^3^ Department of Obstetrics and Gynaecology Mount Sinai Hospital and University of Toronto Toronto Ontario Canada; ^4^ National Institute for Health Research University College London Hospitals Biomedical Research Centre London UK; ^5^ Clinical Department Obstetrics and Gynaecology University Hospitals Leuven Leuven Belgium

## Abstract

**Objective:**

To establish maternal complication rates for fetoscopic or open fetal surgery.

**Methods:**

We conducted a systematic literature review for studies of fetoscopic or open fetal surgery performed since 1990, recording maternal complications during fetal surgery, the remainder of pregnancy, delivery, and after the index pregnancy.

**Results:**

One hundred sixty‐six studies were included, reporting outcomes for open fetal (n = 1193 patients) and fetoscopic surgery (n = 9403 patients). No maternal deaths were reported. The risk of any maternal complication in the index pregnancy was 20.9% (95%CI, 15.22‐27.13) for open fetal and 6.2% (95%CI, 4.93‐7.49) for fetoscopic surgery. For severe maternal complications (grades III to V Clavien‐Dindo classification of surgical complications), the risk was 4.5% (95% CI 3.24‐5.98) for open fetal and 1.7% (95% CI, 1.19‐2.20) for fetoscopic surgery. In subsequent pregnancies, open fetal surgery increased the risk of preterm birth but not uterine dehiscence or rupture. Nearly one quarter of reviewed studies (n = 175, 23.3%) was excluded for failing to report the presence or absence of maternal complications.

**Conclusions:**

Maternal complications occur in 6.2% fetoscopic and 20.9% open fetal surgeries, with serious maternal complications in 1.7% fetoscopic and 4.5% open procedures.

Reporting of maternal complications is variable. To properly quantify maternal risks, outcomes should be reported consistently across all fetal surgery studies.

## Abstract 2

What's already known about this topic?

- Fetal surgery, both open and fetoscopic, is now widely performed.
- Fetoscopy is perceived as safe for the mother, although specific data on maternal complications is lacking.
- Open fetal surgery is known to cause maternal morbidity, but the exact nature and frequency of complications is not well established across different centres and types of surgery.

What does this study add?

- This study estimates the nature and frequency of maternal complications following fetoscopic and open fetal surgery.
- For open fetal surgery, the severe complication rate (grades III to V according to the Clavien‐Dindo classification of surgical complications) is approximately 4% and minor complication rate is 16%.
- For fetoscopic fetal surgery, the severe complication rate is approximately 2% and minor complication rate is 4%.

## 1 INTRODUCTION

The last 35 years have witnessed an expansion of fetal therapy options,^1^, ^2^ with surgery on the fetus, placenta, or cord now relatively common in tertiary‐level fetal medicine units. Enabled by advancements in imaging, surgical instrumentation and techniques, early diagnosis, and treatment of fetal anomalies are now possible for a wide range of conditions.^3^

The mother has been called an “innocent bystander” in fetal surgery,^4^ and generally, fetal therapy is almost exclusively offered to women who are healthy themselves. Fetal surgery poses risks to the mother not only during the procedure itself but also throughout the remainder of the index pregnancy, potentially during any future pregnancies and throughout the woman's entire life. Fetal surgery offers no direct medical benefit to the mother, and from an ethical perspective, maternal risks should be minor and acceptable to the mother and family.^5^

Information regarding safety of surgery is important for counselling and informed decision making; however, robust data on maternal complications of fetal surgery are lacking. One single‐centre study of maternal outcomes following both open fetal and fetoscopic surgery performed between 1989 and 2003 found a number of short‐term morbidities.^6^ A systematic review of maternal complications following fetoscopic laser coagulation for twin‐to‐twin transfusion syndrome (TTTS) in 1785 patients treated between^7^ 1990 and 2009 observed an overall adverse event rate of 5.4% with severe complications in 1.0%. The aim of this study was to estimate the incidence of immediate and long‐term maternal complications of fetoscopic or open fetal surgery through a systematic review of the literature.

**What's already known about this topic?**

- Fetal surgery, both open and fetoscopic, is now widely performed.
- Fetoscopy is perceived as safe for the mother, although specific data on maternal complications is lacking.
- Open fetal surgery is known to cause maternal morbidity, but the exact nature and frequency of complications is not well established across different centres and types of surgery.

**What does this study add?**

- This study estimates the nature and frequency of maternal complications following fetoscopic and open fetal surgery.
- For open fetal surgery, the severe complication rate (grades III to V according to the Clavien‐Dindo classification of surgical complications) is approximately 4% and minor complication rate is 16%.
- For fetoscopic fetal surgery, the severe complication rate is approximately 2% and minor complication rate is 4%.

## 2 METHODS

### 2.1 Protocol and registration

This systematic review was conducted in accordance with preferred reporting items for systematic reviews and meta‐analyses (PRISMA) guidance.^8^ The protocol was registered with the international prospective register of systematic reviews (PROSPERO‐CRD42017082411).

### 2.2 Eligibility criteria

All randomised, cohort, and case‐controlled studies and case series reporting the results of open fetal or fetoscopic fetal surgery in humans from January 1990 to October 2018 were considered eligible. No language restrictions were applied. Systematic reviews, narrative review articles, and case reports were excluded. There is no accepted numerical definition of a case series.^9^ We used an empirical cut‐off of at least three cases because of the rarity of some procedures and conditions searched for.

### 2.3 Search strategy

A systematic review was conducted in MEDLINE, EMBASE, and Cochrane databases using free text and Medical Subject Headings (MESH). The electronic search strategy is described in the Supporting Information. Subsequently, a grey literature (first 100 hits in PubMed and Google Scholar) search was performed, and reference lists of relevant review articles were manually checked. Covidence software (Veritas Health Innovation Ltd, Melbourne, Australia) was used to eliminate duplicate articles and manage study screening.

### 2.4 Study selection

Two authors (A.S. and L.VdV.) screened all titles and abstracts independently, excluded irrelevant studies and then independently assessed the remaining full‐text articles for eligibility; disagreements were resolved by consensus. Studies were excluded if the full text was unavailable online and the abstract contained insufficient information. Studies with interventions which were not fully described or were performed on the neonate instead of the fetus were excluded. Interventions involving access to the uterus using a device with a total outer diameter of less than 1.5 mm were excluded; this cut‐off was chosen to avoid procedures performed with needles only (eg, amniocentesis, fetal blood transfusion, thoracocentesis or vesicocentesis). Studies of shunting were only included if the outer shunt diameter was greater than or equal to 1.5 mm or the shunt was inserted fetoscopically. Studies which did not report maternal outcomes were excluded. For the purpose of this study, preterm rupture of membranes (PROM), chorionic membrane separation (CMS), preterm labour, preterm delivery, and gestational age at delivery, although relevant, were not considered to be maternal complications. Studies from which data could not be extracted (eg, composite or combined outcomes given) and studies containing patient cohorts which appeared to have been published previously by the same authors were excluded.

### 2.5 Data extraction

Two authors independently extracted data (A.S. and E.B. for open fetal surgery studies, A.S. and C.F. for fetoscopic studies) and entered them into a standardised Excel (Microsoft, Washington, USA) form. Disagreements were resolved by consensus. The ex utero intrapartum treatment (EXIT) procedure was classified as open fetal surgery. Study characteristics noted included study design, underlying fetal condition, type of intervention, presence of a control group, gestational age at surgery, and number of patients in each study. Outcomes recorded for the duration of the index pregnancy included intraoperative complications (maternal death, placental abruption, uterine bleeding/haemorrhage, blood transfusion, organ damage, or anaesthetic complications), post‐operative complications (classified from the end of surgery until delivery; maternal death, placental abruption, uterine bleeding/haemorrhage, blood transfusion, sepsis, chorioamnionitis, other infections, pulmonary oedema, amniotic fluid embolism and other respiratory, gastro‐intestinal, cardiac, or wound problems), complications at delivery of the index pregnancy (uterine dehiscence or rupture or blood transfusion), and the need for additional treatment at any time during the pregnancy. Outcomes noted at any time following the index pregnancy (late outcomes) included fertility (number of further pregnancies, difficulty conceiving, mean time to conception), future pregnancy complications (miscarriage or preterm delivery), complications during future deliveries (uterine dehiscence or rupture or haemorrhage at delivery), gynaecological and psychological symptoms. When a study reported “haemorrhage” or an actual blood loss of greater than 1000 mL we noted this as “haemorrhage.” This cut‐off is an accepted definition of severe bleeding both in pregnancy^10^ and post‐partum.^11^ If a study did not specify whether a complication occurred intraoperatively or post‐operatively (eg, placental abruption and requirement for blood transfusion), then this was assumed to have occurred post‐operatively.

All complications were independently graded according to the Clavien‐Dindo classification of surgical complications^12^ by two authors (A.S. and L.VdV.) (Supporting Information). Clavien‐Dindo grade I or II complications were defined as mild; grades III to V complications were defined as severe.^12^

### 2.6 Quality assessment of studies

Study quality and risk of bias were analysed by two authors (A.S. and L.VdV.) independently using a standardised form. Randomised trials were analysed using the Cochrane Collaboration's tool for assessing risk of bias.^13^ Case‐control studies were analysed using the Newcastle‐Ottawa scale for assessing the quality of nonrandomised studies.^14^ Case series were analysed using the National Institutes of Health study quality assessment tool.^15^

### 2.7 Assessment of heterogeneity

Methodological and clinical heterogeneity of data per study were evaluated. Variables were tested for statistical heterogeneity by applying the I^2^ test to determine whether data could be pooled. An I^2^ value less than 40% was taken to indicate minor heterogeneity; 40% to 75% moderate heterogeneity and greater than 75% substantial heterogeneity.^13^

### 2.8 Meta‐analysis

Meta‐analysis for all outcomes was carried out using MedCalc statistical software version 15.4 (MedCalc Software, Ostend, Belgium). Results were expressed as proportions with 95% confidence intervals (CI) as all outcomes were categorical variables. Pooled proportions were calculated using both the fixed and random effects model in case of homogeneity or heterogeneity respectively.

## 3 RESULTS

### 3.1 Study selection

**Figure 1:**
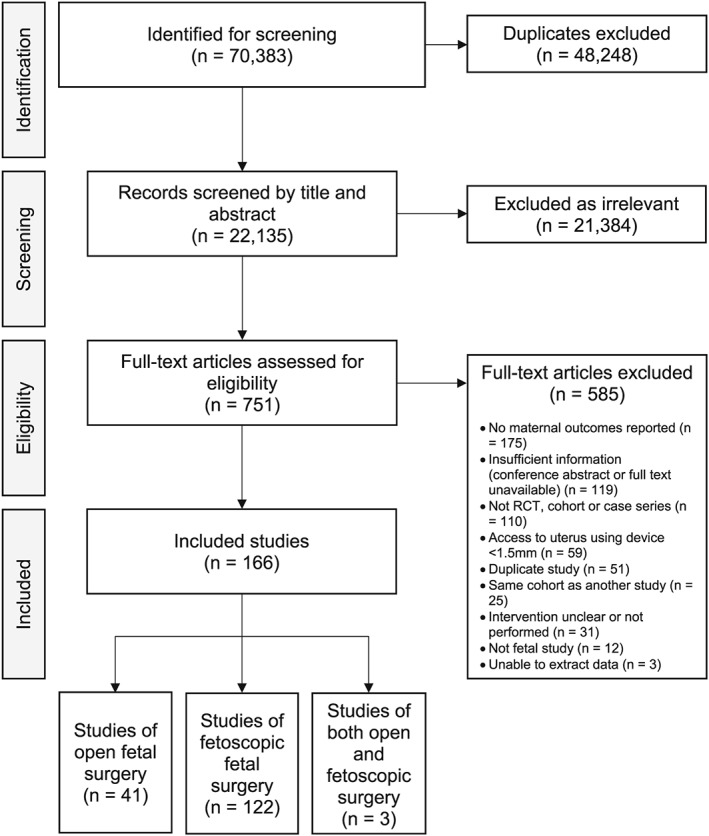
Flow diagram of study selection adapted from preferred reporting items for systematic reviews and meta‐analyses (PRISMA)^8^ 2009

The electronic literature search identified 70 367 studies published between 1990 and 2018 (Figure 1); search of the grey literature and reference lists identified a further 16 studies. Following this, 48 248 studies were immediately removed as duplicates. The remaining studies (22 135) were screened by title and abstract, and a further 21 384 were excluded as irrelevant. Full texts of the remaining 751 articles were reviewed, and 585 were excluded for the following reasons: no reporting of maternal outcomes (175/585, 29.9% of studies excluded and 23.3% [175/751] of all studies assessed), insufficient information available (conference abstract/poster only or full text unavailable) (119/585, 20.3%), study design other than randomised trial, case‐control trial or case series (110/585, 18.8%), and uterine access using a device less than 1.5 mm (59/585, 10.1%). Thirty studies were translated from French (10), Spanish (seven), Polish (five), German (three), Dutch (two), Portuguese (two), and Turkish (one), of which 16 were included following review. Two Chinese‐language papers were identified, but the full text could not be accessed online. Eventually, 166 studies were included; 41 on open fetal surgery, 122 on fetoscopic surgery, and three studies including both surgery types.

### 3.2 Study characteristics

Characteristics of included studies are shown in Tables 1, 2, 3. Studies of open fetal (Table 1) and fetoscopic (Table 2) surgery are presented and analysed separately as the difference in surgical technique was considered too great for combined analysis. Seven studies specifically focused on late complications, ie, after the index pregnancy and are presented separately (Table 3). The majority of studies (68.1%, 113/166) were case series, ie, without a control group; 27.1% (45/166) were case control studies, and 4.8% (8/166) were randomised trials.

**Table 1:** Included studies of open fetal surgery

Category	First Author and Year of Publication	Condition	Procedure	Study Design	No of Patients
EXIT	Barthod,^16^ 2013	Neck mass, CHAOS	EXIT	Case series	5
	Cass,^17^ 2013	Lung mass, mediastinal mass	EXIT	Case series	9
	Chen,^18^ 2018	Omphalocele	EXIT	Case control	7
	Dahlgren,^19^ 2004	Head or neck tumour	EXIT	Case series	4
	Flake,^20^ 2000^a^	CDH	EXIT	Case series	15
	George,^21^ 2007	Skeletal dysplasia, micrognathia	EXIT	Case series	3
	Hedrick,^22^ 2003	Multiple	EXIT	Case series	43
	Hedrick,^23^ 2005	Lung lesions	EXIT	Case series	9
	Kern,^24^ 2007	CCAM, hydrothorax	EXIT	Case series	5
	Kornacki,^25^ 2017	Neck mass, CHAOS	EXIT	Case series	4
	Kunisaki,^26^ 2007	CDH	EXIT	Case control	14
	Laje,^27^ 2012	Cervical teratoma	EXIT	Case series	17
	Laje,^28^ 2013	Neck mass	EXIT	Case series	4
	Laje,^29^ 2015	Cervical lymphatic mass	EXIT	Case series	13
	Lazar,^30^ 2011	Neck mass	EXIT	Case series	12
	Noah,^31^ 2002	Not stated	EXIT	Case control	34
	Pellicer,^32^ 2007	Neck mass	EXIT	Case series	3
	Stoffan,^33^ 2012	CDH	EXIT	Case control	7
	Tuncay Ozgunen,^34^ 2010	Neck mass	EXIT	Case series	3
	Zamora,^35^ 2013^c^	MMC, lung mass, SCT	EXIT	Case series	26
MMC	Bennett,^36^ 2014	MMC	Neurosurgical repair	Case control	43
	Botelho,^37^ 2017	MMC	Neurosurgical repair	Case series	45
	Bruner,^38^ 1999	MMC	Neurosurgical repair	Case control	29
	Bruner,^39^ 2000^c^	MMC	Neurosurgical repair	Case control	4
	Farmer,^40^ 2003	MMC	Neurosurgical repair	Case series	12
	Friszer,^41^ 2016	MMC	Neurosurgical repair	Case series	3
	Johnson,^42^ 2016	MMC	Neurosurgical repair	Randomised	91
	Marenco,^43^ 2013	MMC	Neurosurgical repair	Case series	4
	Moldenhauer,^44^ 2015	MMC	Neurosurgical repair	Case series	100
	Moron,^45^ 2018	MMC	Neurosurgical repair	Case series	237
	Ochsenbein‐Kolble,^46^ 2017	MMC	Neurosurgical repair	Case control	30
	Sinskey,^47^ 2017	MMC	Neurosurgical repair	Case series	47
	Soni,^48^ 2016	MMC	Neurosurgical repair	Case series	88
	Zamlynski,^49^ 2014	MMC	Neurosurgical repair	Case control	46
CDH	Flake,^20^ 2000^a^	CDH	Tracheal occlusion	Case series	15
	Harrison,^50^ 1990	CDH	Diaphragm repair	Case series	6
	Harrison,^51^ 1993	CDH	Diaphragm repair	Case series	14
	Harrison,^52^ 1998^b^	CDH	Tracheal occlusion	Case control	13
CCAM	Adzick,^53^ 2003	CCAM	Lung resection	Case series	22
SCT	Hedrick,^54^ 2004	SCT	Debulking	Case series	4
Mixed	Golombeck,^6^ 2006^a^, ^b^	MMC, CCAM, SCT	Mixed	Case control	79
	Longaker,^55^ 1991	LUTO, CDH, SCT, CCAM	Mixed	Case series	17
	Zamora,^35^ 2013^c^	MMC, lung mass, SCT	Mixed	Case series	7
TOTAL				43 studies	1193 patients

Abbreviations: CCAM, congenital cystic adenomatoid malformation; CD, congenital diaphragmatic hernia; CHAOS, congenital high airway obstruction syndrome; EXIT, ex utero intrapartum treatment; LUTO, lower urinary tract obstruction; MMC, myelomeningocele; SCT, sacrococcygeal teratoma.

a Studies including patients undergoing a primary fetal and later an EXIT procedure.

b Studies including both open and fetoscopic procedures also included in Table 2.

c Studies including immediate and late complications also included in Table 3.

**Table 2:** Included studies of fetoscopic surgery

Category	First Author and Year of Publication	Condition	Procedure	Study Design	No of Patients
Multiple pregnancy complications treated with laser	Aboudiab,^56^ 2017	TTTS	Laser photocoagulation	Case series	18
	Baschat,^57^ 2013	TTTS	Laser photocoagulation	Case control	147
	Chalouhi,^58^ 2016	TTTS (triplets)	Laser photocoagulation	Case series	22
	Chang,^59^ 2006	TTTS	Laser photocoagulation	Case series	27
	Chang,^60^ 2016	TTTS	Laser photocoagulation	Case control	100
	Chmait,^61^ 2013	TTTS	Laser photocoagulation	Case control	318
	Chmait,^62^ 2017	TTTS	Laser photocoagulation	Case series	19
	Crombleholme,^63^ 2007	TTTS	Laser photocoagulation	Randomised	20
	De Lia,^64^ 1995	TTTS	Laser photocoagulation	Case series	26
	De Lia,^65^ 1999	TTTS	Laser photocoagulation	Case series	67
	De Lia,^66^ 2009	TTTS (triplets)	Laser photocoagulation	Case series	10
	Deprest,^67^ 1998	TTTS	Laser photocoagulation	Case series	6
	Draga,^68^ 2016	TTTS	Laser photocoagulation	Case series	37
	Duron,^69^ 2014	TTTS	Laser photocoagulation	Case control	85
	Ek,^70^ 2012	TTTS	Laser photocoagulation	Case series	67
	Habli,^71^ 2009	TTTS	Laser photocoagulation	Case series	152
	Has,^72^ 2014	TTTS	Laser photocoagulation	Case series	85
	Hecher,^73^ 2000	TTTS	Laser photocoagulation	Case control	200
	Hernandez‐Andrade,^74^ 2011	TTTS	Laser photocoagulation	Case series	35
	Huber,^75^ 2008	TTTS	Laser photocoagulation	Case control	176
	Ishii,^76^ 2014	TTTS (triplets)	Laser photocoagulation	Case series	16
	Ishii,^77^ 2015	sFGR	Laser photocoagulation	Case series	10
	Lanna,^78^ 2017	TTTS	Laser photocoagulation	Case control	373
	Lecointre,^79^ 2017	TTTS	Laser photocoagulation	Case control	200
	Malshe,^80^ 2017	TTTS	Laser photocoagulation	Case series	203
	Martinez,^81^ 2012	TTTS	Laser photocoagulation	Case series	500
	Middeldorp,^82^ 2007	TTTS	Laser photocoagulation	Case series	100
	Miyadahira,^83^ 2018	sFGR	Laser photocoagulation	Case control	67
	Molina‐Garcia,^84^ 2009	TTTS, sFGR	Laser photocoagulation	Case series	22
	Morris,^85^ 2010	TTTS	Laser photocoagulation	Case series	164
	Mullers,^86^ 2015	TTTS	Laser photocoagulation	Case series	105
	Nakata,^87^ 2016	TTTS	Laser photocoagulation	Case series	6
	Nguyen,^88^ 2012	TTTS	Laser photocoagulation	Case series	98
	Ozawa,^89^ 2017	Amniotic fluid discordance	Laser photocoagulation	Case series	11
	Papanna,^90^ 2010	TTTS	Laser photocoagulation	Case control	48
	Papanna,^91^ 2012	TTTS	Laser photocoagulation	Case control	163
	Peeters,^92^ 2014	TTTS	Laser photocoagulation	Case control	338
	Persico,^93^ 2016	TTTS	Laser photocoagulation	Case series	106
	Quintero,^94^ 2000	TTTS	Laser photocoagulation	Case control	92
	Quintero,^95^ 2001	sFGR	Laser photocoagulation	Case series	11
	Rossi,^96^ 2008	TTTS	Laser photocoagulation	Case control	266
	Ruano,^97^ 2009	TTTS	Laser photocoagulation	Case series	19
	Ruegg,^98^ 2018	TTTS	Laser photocoagulation	Case control	37
	Rustico,^99^ 2012	TTTS	Laser photocoagulation	Case series	150
	Said,^100^ 2008	TTTS	Laser photocoagulation	Case series	10
	Senat,^101^ 2004	TTTS	Laser photocoagulation	Randomised	72
	Sepulveda,^102^ 2007	TTTS	Laser photocoagulation	Case series	33
	Shamshirsaz,^103^ 2015	TTTS	Laser photocoagulation	Case control	55
	Slaghekke,^104^ 2014	TTTS	Laser photocoagulation	Randomised	274
	Taniguchi,^105^ 2015	TTTS	Laser photocoagulation	Case series	3
	Tchirikov,^106^ 2011	TTTS	Laser photocoagulation	Case control	80
	Teoh,^107^ 2013	TTTS	Laser photocoagulation	Case series	49
	Thia,^108^ 2017	TTTS	Laser photocoagulation	Case series	5
	Ville,^109^ 1997	TTTS	Laser photocoagulation	Case series	132
	Ville,^110^ 1998	TTTS	Laser photocoagulation	Case control	44
	Weingertner,^111^ 2011	TTTS	Laser photocoagulation	Case series	100
	Wilson,^112^ 2016	TTTS	Laser photocoagulation	Case series	151
	Yamamoto,^113^ 2005	TTTS	Laser photocoagulation	Case series	175
	Yang,^114^ 2010	TTTS	Laser photocoagulation	Case series	30
	Zaretsky,^115^ 2018	TTTS	Laser photocoagulation	Case series	749
	Zhao,^116^ 2016	TTTS	Laser photocoagulation	Case control	62
Multiple pregnancy complications treated with selective reduction	Bebbington,^117^ 2012	TTTS, TRAP, sFGR, discordant anomaly	RFA	Case control	146
	Berg,^118^ 2014	TRAP	RFA	Case control	7
	Delabaere,^119^ 2013	TTTS, TRAP, sFGR, discordant anomaly	BCC, cord compression, cord ligation	Case series	30
	Deprest,^120^ 2000	TTTS, TRAP	BCC	Case series	10
	Gallot,^121^ 2003	TTTS, TRAP	CO	Case series	11
	Gouverneur,^122^ 2009	TTTS, TRAP, sFGR, discordant anomaly	BCC, laser cord photocoagulation	Case series	54
	Gul,^123^ 2008	TTTS, TRAP, discordant anomaly	BCC	Case series	9
	Has,^124^ 2014	TTTS, TRAP, sFGR, discordant anomaly	BCC	Case series	71
	He,^125^ 2010	TTTS, TRAP, sFGR, discordant anomaly	BCC	Case series	14
	Ilagan,^126^ 2008	TTTS, TRAP, discordant anomaly	BCC	Case series	27
	Jelin,^127^ 2010	TRAP	RFA	Case control	7
	King,^128^ 2017	TRAP, discordant anomaly	Laser cord photocoagulation	Case series	43
	Lanna,^129^ 2012	TTTS, TRAP, sFGR, discordant anomaly	BCC	Case series	118
	Lee,^130^ 2013	TRAP	RFA	Case series	98
	Lewi,^131^ 2006	TTTS, TRAP, sFGR, discordant anomaly	Laser cord photocoagulation	Case series	80
	Moise,^132^ 2008	TTTS, discordant anomaly	RFA	Case series	9
	Nobili,^133^ 2013	Discordant anomaly	BCC	Case series	48
	Paramasivam,^134^ 2010	TTTS, TRAP, sFGR, discordant anomaly	RFA	Case series	35
	Peng,^135^ 2016	TTTS, TRAP, sFGR, discordant anomaly, TAPS	BCC	Case control	93
	Quintero,^136^ 1996	TTTS, TRAP, discordant anomaly	CO	Case series	13
	Quintero,^137^ 2006	TRAP	CO or laser photocoagulation	Case control	51
	Roman,^138^ 2010	TTTS, TRAP, sFGR, discordant anomaly	RFA	Case control	60
	Schou,^139^ 2018	TTTS, TRAP, sFGR, discordant anomaly	BCC	Case control	102
	Sugibayashi,^140^ 2016	TRAP	RFA	Case series	40
	Takano,^141^ 2015	TRAP	Laser photocoagulation +/− transection of cord (MCMA)	Case series	10
	Taylor,^142^ 2002	TTTS	BCC	Case series	15
	Tsao,^143^ 2002	TRAP	RFA	Case series	13
	Zhang,^144^ 2018	TRAP	RFA	Case series	25
CDH	Deprest,^145^ 2005	CDH	FETO	Case series	20
	Harrison,^52^ 1998^a^	CDH	Tracheal clip	Case control	8
	Harrison,^146^ 2003	CDH	FETO	Randomised	11
	Jani,^147^ 2005	CDH	FETO	Case series	24
	Jani,^148^ 2006	CDH	FETO	Case series	28
	Jani,^149^ 2009	CDH	FETO	Case series	210
	Jimenez,^150^ 2017	CDH	Fetoscopic balloon removal	Case control	201
	Kosinski,^151^ 2017	CDH	FETO	Case series	28
	Manrique,^152^ 2008	CDH	FETO	Case control	11
	Peralta,^153^ 2011	CDH	FETO	Case series	8
	Persico,^154^ 2017	CDH	FETO	Case series	21
	Ruano,^155^ 2012	CDH	FETO	Case control	35
	Ruano,^156^ 2012	CDH	FETO	Randomised	20
	Ruano,^157^ 2013	CDH	FETO	Case control	17
MMC	Arens,^158^ 2017	MMC	Patch	Case series	59
	Belfort,^159^ 2017	MMC	Single layer suture (skin + dura)	Case series	22
	Bruner,^39^ 2000^a^	MMC	Maternal skin graft	Case control	4
	Degenhardt,^160^ 2014	MMC	Patch	Case series	51
	Kohn,^161^ 2018	MMC	Patch	Case series	34
	Pedreira,^162^ 2014	MMC	Patch + skin suture	Case series	4
	Pedreira,^163^ 2016	MMC	Patch + skin suture	Case series	10
	Verbeek,^164^ 2012	MMC	Patch	Case control	19
	Ziemann,^165^ 2018	MMC	Patch	Case series	65
LUTO	Morris,^166^ 2013	LUTO	Vesicoamniotic shunting	Randomised	16
	Ruano,^167^ 2010	LUTO	Cystoscopy	Case control	11
	Welsh,^168^ 2003	LUTO	Cystoscopy	Case series	13
Shunts	Cavalheiro,^169^ 2011	Ventriculomegaly	Shunting	Case series	30
	Mallman,^170^ 2017	Hydrothorax	Shunting	Case series	78
Mixed	Golombeck,^6^ 2006^a^	TTTS, TRAP, CDH, LUTO	Mixed	Case control	99
	Kohl,^171^ 2006	MMC, CDH, CHAOS	Mixed	Case series	16
	Kohl,^172^ 2010	MMC, TTTS, CDH, CHAOS, ABS	Mixed	Case series	37
	Nivatpumin,^173^ 2016	TTTS, LUTO, CDH, TRAPS	Mixed	Case series	152
	Peralta,^174^ 2010	TTTS, CDH, TRAP	Mixed	Case series	56
TOTAL				122 studies	9403 patients

Abbreviations: BCC, bipolar cord coagulation; CDH, congenital diaphragmatic hernia; CHAOS, congenital high airway obstruction syndrome; CO, cord occlusion; FETO, fetoscopic endoluminal tracheal occlusion; LUTO; lower urinary tract obstruction; MCMA, monochorionic monoamniotic; MMC, myelomeningocele; RFA, cord radiofrequency ablation; sFGR; selective fetal growth restriction; TAPS, twin anaemia‐polycythaemia sequence; TO, tracheal occlusion; TRAP, twin reversed arterial perfusion sequence; TTTS, twin‐to‐twin transfusion syndrome.

a Studies including both open and fetoscopic procedures also included in Table 1.

**Table 3:** Included studies of open and fetoscopic surgery focusing on late complications

First Author and Year of Publication	Type of Surgery	Condition	Study Design	Number of Patients
Farrell,^4^ 1999	Open	CDH, CCAM, LUTO, SCT,	Case series	45
Thom,^175^ 2016	Open	MMC	Randomised	87
Wilson,^176^ 2010	Open	MMC, CCAM, CDH, SCT, mediastinal teratoma	Case series	47
Zamora,^35^ 2013^a^	Open	MMC, lung mass, SCT, EXIT	Case series	33
Gregoir,^177^ 2016	Fetoscopic	CDH	Case control	89
Le Lous,^178^ 2018	Fetoscopic	TTTS	Case control	122
Vergote,^179^ 2018	Fetoscopic	TTTS	Case control	92
TOTAL			7 studies	515 patients

Abbreviations: CCAM, congenital cystic adenomatoid malformation; CDH, congenital diaphragmatic hernia; EXIT, ex utero intrapartum treatment; LUTO, lower urinary tract obstruction; MMC, myelomeningocele; SCT, sacrococcygeal teratoma; TTTS, twin‐to‐twin transfusion syndrome.

a Studies including immediate and late complications, also included in Table 1.

### 3.3 Risk of bias

Quality assessment of the studies is given in the Supporting Information. Most studies (139/166, 83.7%) had a low risk of bias or were high quality. All remaining studies (27/166, 16.3%) had an unclear risk of bias or were fair quality. No studies were found to have a high risk of bias or be low quality overall. For randomised trials, included studies had a high risk of bias with regards to blinding. For case control studies, included studies did not describe statistical methods well overall.

### 3.4 Statistical heterogeneity

Maternal outcome data were pooled in 64 separate meta‐analyses, of which 37.5% (24/64) had no or minor heterogeneity. In 39.1% (25/64), there was moderate heterogeneity, and in 23.4% (15/64), there was considerable heterogeneity. The levels of heterogeneity per outcome measure are listed in the Supporting Information. As both clinical and statistical heterogeneity were found, pooled proportions were given using the random effects model for meta‐analysis.

### 3.5 Maternal complications in the index pregnancy—intraoperative

Table 4 summarises maternal complications according to type of surgery performed. No maternal deaths (Clavien‐Dindo grade V) due to fetal surgery were reported in any study (10 596 procedures). One study^86^ reported a patient at 20 weeks' gestation experiencing a cardiorespiratory arrest prior to fetoscopy for laser photocoagulation. The cause was considered to be a combination of morbid obesity, spinal anaesthesia, and aortocaval compression and not related to the procedure, which had not commenced. An immediate delivery was conducted by hysterotomy as part of maternal resuscitation, and the patient made a full recovery. Another study^47^ reported brief maternal seizure‐like activity during open fetal surgery, which was thought to be anaesthesia‐related.

**Table 4:** Maternal complications occurring with open or fetoscopic fetal surgery

	Severe Complications				Minor Complications		All Complications
Clavien‐Dindo classification	IV (requiring ICU care)		III (requiring surgical intervention)		I‐II (requiring treatment)		I‐IV
Open surgery n = 1193	Complication	n	Complication	n	Complication	n	**ALL COMPLICATIONS: 20.86% (95% CI, 15.22‐27.13)**
	Severe infection	2	Haemorrhage requiring delivery	3	Bleeding during procedure	13	
	Pulmonary oedema	4	Placental abruption	28	Transfusion during/after procedure	41	
	Complete heart block^a^	1	Bowel obstruction	1	Chorioamnionitis/endometritis	45	
			Wound drainage	2	Other infections^b^	8	
			Uterine rupture	5	Pulmonary oedema	50	
			Laparotomy/dehiscence repair	1	Transfusion at delivery	17	
			Caesarean hysterectomy	1			
					
	**TOTAL SEVERE: 4.51%** **(95% CI, 3.24‐5.98)**				**TOTAL MINOR: 16.26%** **(95% CI, 11.17‐22.09)**		
Fetoscopic surgery n = 9403	Maternal cardiac arrest and delivery by hysterotomy	1	Sepsis requiring delivery	1	Bleeding during procedure	165	**ALL COMPLICATIONS: 6.15% (95% CI, 4.93‐7.49)**
	Severe infection	2	Haemorrhage requiring delivery	8	Transfusion during/after procedure	16	
	Pulmonary oedema	3	Placental abruption	159	Venous thromboembolism^c^	2	
	Lung collapse	1			Chorioamnionitis	114	
	DIC + caesarean hysterectomy	1			Other infections^d^	2	
	Amniotic fluid embolism	2			Pulmonary oedema	45	
					Upper GI bleed^e^	1	
					Diathermy skin burns	4	
					“Epidural headache” + blood patch	1	
					Wound hernia	1	
					Pleural effusions	1	
					
	**TOTAL SEVERE: 1.66%** **(95% CI, 1.19‐2.20)**				**TOTAL MINOR: 4.33%** **(95% CI, 3.33‐5.45)**		

Abbreviations: CI, confidence interval; GI, gastrointestinal; n, number of women. Pooled proportions calculated using random effect model for meta‐analysis.

a Complete heart block considered to be tocolysis‐related (magnesium sulphate).

b Other infections in open surgery: wound (6), chest (1), urinary tract (1).

c Venous thromboembolism: confirmed pulmonary embolism (1); suspected PE with confirmed deep vein thrombosis (1).

d Other infections in fetoscopic surgery: wound (1), chest (1).

e Upper GI bleed considered to be tocolysis‐related (indomethacin).

Haemorrhage severe enough to prompt delivery or termination of pregnancy at the time of surgery as a life‐saving procedure for the mother (Clavien‐Dindo grade III) occurred in 0.92% of open fetal (95% CI, 0.46‐1.62) and 0.26% of fetoscopic surgeries (95% CI, 0.17‐0.38). Three cases^38^, ^45^, ^46^ occurred because of placental abruption during open fetal surgery for myelomeningocele (MMC) repair, following which delivery occurred, with all three fetuses surviving. Two cases^59^, ^75^ occurred following laser photocoagulation for TTTS said to be due to “excessive bleeding from placental anastomoses” and the uterine wall, respectively. Two cases^119^, ^121^ occurred during selective reduction, with haemorrhage from the uterine wall prompting delivery. Finally, one pregnancy was terminated because of bleeding from a trocar placental injury during fetoscopic MMC repair.^172^

In total, placental abruption (Clavien‐Dindo grade III) occurred intraoperatively in 1.28% of open fetal (95% CI, 0.73‐1.98) and in 0.28% of fetoscopic surgeries (95% CI, 0.18‐0.39). Bleeding during the procedure was noted in 1.97% of open fetal (95% CI, 0.97‐3.31) and in 1.74% of fetoscopic surgery cases (95% CI, 1.25‐2.32). Intraoperative blood transfusion was required in 1.00% of patients undergoing open fetal surgery (95% CI, 0.53‐1.64) and in 0.27% undergoing fetoscopic surgery (95% CI, 0.18‐0.38). Intraoperative skin burns at the site of diathermy pads occurred in 0.26% of patients (95% CI, 0.17‐0.37) during fetoscopic surgery; this outcome was not reported in any open fetal surgery.

### 3.6 Maternal complications in the index pregnancy—postoperative

One study on laser photocoagulation for TTTS (n = 132)^110^ reported a maternal death from disseminated intravascular coagulation (DIC) 4 weeks following an uneventful procedure. A post‐mortem examination did not find any evidence of chorioamnionitis or amniotic fluid embolism, and the authors therefore concluded that this death was unrelated to the procedure.

Haemorrhage severe enough to prompt return to theatre for termination or delivery of the pregnancy within 24 hours was not reported following any open fetal surgeries but occurred following 0.25% of fetoscopic procedures (95% CI, 0.16‐0.37). This included one^171^ 4‐hour post‐fetoscopic tracheal balloon removal with no cause of the bleeding found. There were two late placental abruptions, one^113^ 12‐hour post‐laser photocoagulation, and one^142^ within 24 hours of bipolar cord coagulation.

Placental abruption occurred in 1.80% of patients following open fetal (95% CI, 1.14‐2.63) and in 1.29% following fetoscopic surgery (95% CI, 0.90‐1.75). Post‐operative blood transfusion was given to 3.36% after open fetal surgery (95% CI, 1.85‐5.29) and in 0.32% following fetoscopic surgery (95% CI, 0.22‐0.44).

Chorioamnionitis following open fetal surgery or endometritis following an EXIT procedure occurred in 4.13% of women (95% CI, 3.03‐5.40), and in 1.45% undergoing fetoscopic surgery (95% CI, 1.06‐1.90). Of those, PROM was reported to have occurred in 47.78% following open fetal surgery (95% CI, 23.01‐73.16) and in 36.31% following fetoscopic surgery (95% CI, 22.00‐51.99). One study reported severe chorioamnionitis 5 days after bipolar cord coagulation^131^ with septic shock and acute kidney injury, which resolved leaving 70% residual renal function. Sepsis was also reported in one patient^61^ with chorioamnionitis following fetoscopic laser photocoagulation and in one patient^49^ following open MMC repair who developed post‐operative peritonitis requiring an emergency laparotomy and delivery. Post‐operative pneumonia occurred in two patients—one^132^ following fetoscopic radiofrequency ablation (RFA), necessitating 3 days of intubation and intensive care unit (ICU) care and one requiring ICU admission^36^ following open MMC repair.

Pulmonary oedema occurred in 4.32% of open fetal surgery cases (95% CI, 2.32‐6.90), and in 0.63% of fetoscopic cases (95% CI, 0.43‐0.87). Three studies in which post‐operative pulmonary oedema occurred reported on perioperative fluid management (3/102, 2.9%), and 33 reported on the use of magnesium sulphate (33/102, 32.4%) without specifically suggesting causality. Six women required ICU admission, with four requiring intubation and ventilation; three following open fetal surgery,^6^, ^20^ and three following fetoscopic surgery.^69^, ^87^, ^99^

### 3.7 Maternal complications in the index pregnancy—at delivery

Only a few fetoscopic surgery studies (4/121 studies, 0.33%) reported findings or complications at delivery. Complications at delivery following open fetal surgery are shown in Table 4. Hysterectomy at or around the time of delivery was reported in two patients (Clavien‐Dindo grade III). In one case,^44^ caesarean delivery following open MMC repair in a woman with two previous caesareans, intra‐abdominal scarring and friable tissue eventually resulted in hysterectomy. In the second case,^99^ following laser photocoagulation for TTTS and PROM, a caesarean section was performed at 33 weeks' gestation. A hysterectomy was eventually required because of haemorrhage with DIC, and the patient spent 5 days in ICU, where she also experienced an iatrogenic pneumothorax.

Uterine rupture occurred in 0.90% of patients at delivery following open fetal surgery (excluding EXIT procedures) in the index pregnancy (95% CI, 0.41‐1.59), and uterine dehiscence occurred in 3.67% (95% CI, 2.01‐5.81). Blood transfusion was given to 1.83% of women (95% CI, 1.16‐2.65) at delivery following open fetal surgery.

### 3.8 Overall maternal complication rates

Table 4 displays maternal complications. In open fetal surgery, there was a 4.51% severe (95% CI, 3.24‐5.98), a 16.26% minor complication rate (95% CI, 11.17‐22.09), and a total complication rate of 20.86% (95% CI, 15.22‐27.13). For fetoscopic surgery, the corresponding rates were: 1.66% severe (95% CI, 1.19‐2.20), 4.33% minor (95% CI, 3.33‐5.45), and 6.15% total complications (95% CI, 4.93‐7.49). Complication rates in the six commonest fetal surgical procedures performed are displayed in Table 5.

**Table 5:** Maternal complications according to type of fetal surgery in the six most common procedures

	Severe Complications				Minor Complications		All Complications
Clavien‐Dindo classification	IV (requiring ICU care)		III (requiring surgical intervention)		I‐II (requiring treatment)		I‐IV
EXIT n = 237	Complication	n	Complication	n	Complication	n	**ALL COMPLICATIONS: 20.19% (95% CI, 4.93‐7.49)**
			Placental abruption	5	Bleeding during procedure	11	
					Transfusion during/after procedure	19	
					Endometritis	10	
					Wound infection	5	
					
	**TOTAL SEVERE: 3.62% (95% CI, 1.69‐6.24)**				**TOTAL MINOR: 17.53% (95% CI, 9.86‐26.86)**		
Open MMC repair n = 779	Severe infection	2	Haemorrhage requiring delivery	3	Bleeding during procedure	1	**ALL COMPLICATIONS: 11.54% (95% CI, 7.73‐15.99)**
	Complete heart block	1	Placental abruption	16	Transfusion during/after procedure	5	
	Pulmonary oedema	1	Bowel obstruction	1	Chorioamnionitis	21	
			Uterine rupture	4	Other infections^a^	2	
			Caesarean hysterectomy	1	Pulmonary oedema	15	
					Transfusion at delivery	16	
					
	**TOTAL SEVERE: 3.35% (95% CI, 1.70‐5.53)**				**TOTAL MINOR: 6.63% (95% CI, 3.63‐10.45)**		
Fetoscopic MMC repair n = 268			Placental abruption	6	Bleeding during procedure	3	**ALL COMPLICATIONS: 12.49% (95% CI, 4.83‐23.06)**
					Chorioamnionitis	10	
					Pulmonary oedema	5	
					
	**TOTAL SEVERE: 2.75% (95% CI, 0.56‐6.52)**				**TOTAL MINOR: 9.04% (95% CI, 3.27‐17.40)**		
FETO (insertion or fetoscopic removal of balloon) n = 634			Placental abruption	4	Bleeding during procedure	1	**ALL COMPLICATIONS: 3.44% (95% CI, 0.98‐7.32)**
					Transfusion during/after procedure	1	
					Chorioamnionitis	7	
					Wound infection	1	
					Pulmonary oedema	3	
					
	**TOTAL SEVERE: 1.08% (95% CI, 0.23‐2.54)**				**TOTAL MINOR: 2.39% (95% CI, 0.71‐5.02)**		
Fetoscopic laser photo‐coagulation n = 6746	Maternal arrest and delivery	1	Haemorrhage requiring delivery	2	Bleeding during procedure	148	**ALL COMPLICATIONS: 5.86% (95% CI, 4.33‐7.61)**
	Pulmonary oedema	3	Sepsis requiring delivery	1	Transfusion during/after procedure	9	
	Lung collapse	1	Placental abruption	130	VTE^b^	2	
	Amniotic fluid embolism	2			“Epidural headache” + blood patch	1	
	DIC + caesarean hysterectomy	1			Chorioamnionitis	68	
					Pulmonary oedema	11	
					Upper GI bleed^c^	1	
					Wound hernia	1	
					
	**TOTAL SEVERE: 1.51% (95% CI, 0.91‐2.25)**				**TOTAL MINOR: 4.03% (95% CI, 2.73‐5.56)**		
Fetoscopic selective reduction n = 1239	Severe infection	2	Haemorrhage requiring delivery	3	Bleeding during procedure	10	**ALL COMPLICATIONS: 5.20% (95% CI, 3.00‐7.96)**
			Placental abruption	14	Diathermy skin burns	4	
					Chorioamnionitis	19	
					Chest infection	1	
					Pleural effusion	1	
					
	**TOTAL SEVERE: 1.98% (95% CI, 0.97‐3.35)**				**TOTAL MINOR: 3.00% (95% CI, 1.68‐4.68)**		

Abbreviations: DIC, disseminated intravascular coagulation; EXIT, ex utero intrapartum treatment; FETO, fetoscopic endoluminal tracheal occlusion; GI, gastrointestinal; MMC, myelomeningocele; n, number of women. Pooled proportions calculated using random effect model for meta‐analysis.

a Other infections in MMC surgery: chest (1), urinary tract (1).

b Venous thromboembolism: confirmed pulmonary embolism (1); suspected PE with confirmed deep vein thrombosis (1).

c Upper GI bleed considered to be tocolysis‐related (indomethacin).

### 3.9 Maternal outcomes following the index pregnancy (long‐term)

Table 6 shows subsequent pregnancy outcomes and long‐term maternal outcomes following a pregnancy in which fetal surgery was performed. New difficulties in conceiving were described in 3.81% of women after open fetal surgery (95% CI, 1.22‐7.76, reported in four studies); this outcome was not reported to occur after fetoscopic surgery (three studies). Pregnancy loss prior to 24 weeks' gestation occurred in 19.95% of pregnancies conceived following open fetal surgery (95% CI, 13.37‐27.48, three studies) and 13.67% of pregnancies conceived after fetoscopic surgery (95% CI, 9.34‐18.68, three studies). Preterm birth occurred in 20.49% of pregnancies following open fetal surgery (95% CI, 10.48‐32.81, four studies) and in 2.12% of pregnancies following fetoscopic surgery (95% CI, 0.02‐9.01; three studies). Uterine rupture or dehiscence occurred, respectively, in 6.89% (95% CI, 1.34‐16.27, reported in three studies) and 11.09% (95% CI, 5.34‐18.59) of pregnancies following open fetal surgery. None were mentioned in fetoscopy studies.

**Table 6:** Long‐term maternal complications following open and fetoscopic fetal surgery

		**Open Surgery** a % (95% CI)	**Fetoscopic Surgery** b % (95% CI)
Conception	Women attempting further pregnancy	50.11 (21.55‐78.63)	51.76 (18.63‐84.03)
	Women conceiving further pregnancy	48.33 (26.74‐70.26)	48.20(31.46‐65.16)
	New sub‐fertility	3.81(1.22‐7.76)	NR
Pregnancy outcomes	Miscarriage	19.95 (13.37‐27.48)	13.67 (9.34‐18.68)
	Preterm delivery	20.49 (10.48‐32.81)	2.12 (0.02‐9.01)
	Uterine rupture	6.89 (1.34‐16.27)	0
	Uterine dehiscence	11.09 (5.34‐18.59)	NR
	Excessive bleeding at delivery	6.84 (2.16‐13.88)	5.52 (2.83‐9.03)
Nonpregnancy	Abdominal pain	6.38^b^	9.01 (3.84‐16.06)
	Abnormal menstrual bleeding	NR	6.54 (3.43‐10.57)
	Gynaecological surgery^c^	8.68 (1.81‐19.96)	NR
	Psychological symptoms	9.09^b^	32.56 (7.70‐64.58)

Abbreviation: NR, not reported. Pooled proportions calculated using random effect model for meta‐analysis.

a Variable denominator as not all outcomes were reported by all studies.

b No meta‐analysis possible as reported by single study.

c Gynaecological surgery following open fetal surgery: endometrial ablation (1), hysterectomy (6): caesarean hysterectomy (1), ovarian cysts+/−menstrual disorder (2), fibroids (1), unknown reason (2).

## 4 DISCUSSION

In this systematic review of the literature, we found an overall complication rate of approximately 21% for open fetal surgery and 6% for fetoscopic fetal surgery, of which minor complications occurred in 16% and 4% of surgeries, respectively. This maternal complication rate excludes obstetric complications, which may also have occurred (eg, PROM, CMS, preterm labour, and preterm delivery). Additionally, many studies of fetal surgery fail to document maternal complications. Out of 751 full‐text articles reviewed, 175 (23.3%) were excluded as no maternal outcomes were stated. Although 68 of these studies focused on a specific aspect of the surgery or its neonatal outcome, 107 studies (92 fetoscopic and 15 open) involving over 9000 patients did not comment on the presence or absence of any complications specifically affecting the mother's health. Often, the “maternal outcomes” stated meant in reality obstetric outcomes (eg, PROM and preterm labour). We also found that maternal complications were often presented from the fetal perspective (eg, fetal demise caused by placental abruption). Thirty included studies (18.1%) contained a statement that no adverse maternal outcomes were observed without specifying what was meant by maternal outcomes. Among these studies were some large series, including a study of 201 patients undergoing fetoscopic tracheal balloon removal^150^ and studies of 200^73^ and 500^81^ patients undergoing fetoscopic laser coagulation. It is unlikely that such large numbers of procedures had no maternal complications, and more likely that complications were either not perceived as serious, not reported and/or the patient follow‐up was incomplete. This lack of reporting has most likely led to an underestimation of the actual risk of maternal complications in our meta‐analysis. Conversely, when maternal complications were reported, there was a wide variability in which outcomes were discussed and how they were presented.

There was a severe complication rate (Clavien‐Dindo grade III or IV) of 4.5% in women undergoing open fetal surgery and 1.7% undergoing fetoscopic surgery. This is in keeping with a previous multi‐centre review of maternal complications following laser photocoagulation for TTTS,^7^ which found a 1.0% rate of severe complications and a 5.4% total rate of complications across all studies; however, when the authors only included studies, which systematically assessed maternal complications as a primary or secondary outcome, this rose to 1.8% for severe and 17.4% for all complications.

In almost all studies of fetal surgery reviewed, long‐term maternal follow up was not described. The seven studies that did so had a wide variation in the parameters described. Fertility does not appear to be negatively affected by fetal surgery, with the rates of de novo difficulties for conceiving in this review (3.81% following open fetal surgery and none following fetoscopic surgery) being comparable, if not less, than published rates of secondary infertility in the general population.^180^ Similarly, the rates of miscarriage described (19.85% following open fetal and 13.67% following fetoscopic surgery) are similar to rates of spontaneous miscarriage in women who have not undergone fetal surgery.^181^, ^182^, ^183^ Epidemiological studies^184^ have suggested a worldwide preterm birth rate of 11.1% with a rate of 8.6% in “developed regions.”^184^ In the United States and United Kingdom, it is estimated at 9.8%^185^ and 7.3%,^186^ respectively. The preterm birth rate in this review following open fetal surgery (20.49%) is higher than the usual prevalence, but not higher following fetoscopic surgery (2.12%). Open fetal surgery was followed by uterine rupture or dehiscence in 6.89% and 11.09% of subsequent pregnancies, respectively, which is in line with published rates of rupture (6.2%) and dehiscence (12.5%) following a classical caesarean section.^187^ Conversely, no uterine ruptures were reported following fetoscopic surgery.

This study included the commonest fetal procedures and, from a maternal perspective, involved similar surgical manipulations yet variable operating times. We included studies from multiple centres worldwide and attempted to identify the non‐English literature. It is therefore likely that these results are generalisable to fetal surgery performed outside the included studies. An obvious weakness of this systematic review is that most studies did not include a control group. Furthermore, we decided to pool data for meta‐analysis despite having high heterogeneity in some results. Another weakness is the extraction of patient data from papers, which is prone to error given the variable reporting; it is possible that some patients had more than one complication and this was not noted or cumulative rates were as a consequence miscalculated.

This systematic review has identified a significant rate of maternal complications, which should be discussed with patients before embarking on fetal surgery. Large studies allow an estimation of the likelihood of these events, insomuch as the cases in these series are unselected and consecutive. Our systematic review search strategy may have missed relevant yet rare complications. For example, a letter to a journal editor describing maternal convulsions during general anaesthesia^188^ was excluded as a case report according to our criteria. In this circumstance, it appears that the patient was also part of the cohort of a study that was included,^47^ but it is possible that other rare events published as case reports have been missed. An international, prospective registry of fetal and fetoscopic surgery, such as the Eurofoetus^189^ and NAFTNet^190^ registries, would be the best way to accurately determine complication types and rates and avoid missing rare complications.

## 5 CONCLUSION

The maternal risks of fetal surgery are accepted by many patients and health care professionals for the possible benefit to the fetus.^191^, ^192^ This systematic review finds that studies of fetal surgery focus on the fetal outcomes of the procedure, and many fail to describe maternal complications. Fetal surgery comes at a risk to the mother, which may be underestimated by fetal therapists because of under‐reporting and variable reporting quality. In order to properly quantify maternal risks, outcomes should be reported consistently across all studies of fetal surgery, preferentially in prospective registries.

## CONFLICT OF INTEREST

All authors report no conflict of interest.

## FUNDING INFORMATION

This research is funded by the Wellcome Trust (WT101957) and Engineering and Physical Sciences Research Council (ESPRC) (NS/A000027/1). J.D. is also funded by the Great Ormond Street Hospital Children's Charity Fund. A.L.D. is supported by the National Institute for Health Research University College London Hospitals Biomedical Research Centre. LvdV is funded with support of the Erasmus + Programme of the European Union (Framework Agreement number: 2013‐0040). This publication reflects the views only of the author, and the commission cannot be held responsible for any use, which may be made of the information contained therein.

## Supporting information

Data S1: Search Strategy
Table S2: Classification of maternal surgical complications^12^
Table S3: Summary of risk of bias according to study type.
Table S4: Statistical heterogeneity according to outcome analysed.

Data S2. Supporting information
