# Supplementary material for: Maternal complications following open and fetoscopic fetal surgery: A systematic review and meta‐analysis
Source: Prenat Diagn. 2019 Feb 27;39(4):251–68. doi: 10.1002/pd.5421 (PMC6492015; doi:10.1002/pd.5421)
Supplement: Supplementary file 1 — Data S1: Search Strategy Table S2: Classification of maternal surgical complications12 Table S3: Summary of risk of bias according to study type. Table S4: Statistical heterogeneity according to outcome analysed. [file PD-39-251-s001.docx]

Supplement 1: Search Strategy

Search terms were: “F(o)etal surgery”, “Fetoscopy”; “Fetoscopic surgery”; “Endoscopic f(o)etal surgery”; “Ex-utero intrapartum treatment”; “EXIT procedure”; “Operation on placental support”; “OOPS”; “Airway management on placental support”; “Bipolar cord coagulation”; “Cord ablation”; “Cord coagulation”; “Cord occlusion”; “Cord radiofrequency ablation”; “Selective f(o)etal reduction”; “Selective termination”; “Microwave ablation”; “Multifetal pregnancy reduction”; “F(o)etal endoluminal tracheal occlusion”; “FETO”; “Discordant anomaly”; “Selective fetal growth restriction”; “sFGR”; “Selective intrauterine growth restriction”; “sIUGR”; “Twin anaemia polycythaemia sequence”; “TAPS”; “Twin reversed arterial perfusion”; “TRAP sequence”; “Twin to twin transfusion syndrome”; “TTTS”.

“Maternal” AND [“F(o)etal surgery” OR “Fetoscopy” OR “Fetoscopic surgery” OR “Endoscopic f(o)etal surgery”].

“F(o)etal” AND: [“Amniotic band syndrome” OR “BCC” OR “BPS” OR “Bronchopulmonary sequestration” OR “CCAM” OR “CDH” OR “Cervical lymphangioma” OR “Cervical teratoma” OR “CHAOS” OR “Chest mass” OR “Chorangioma” OR “Congenital cystic adenomatoid malformation” OR “Congenital diaphragmatic hernia” OR “Congenital high airway obstruction syndrome”; OR “Congenital pulmonary airways malformation”; OR “CPAM” OR “Cystoscopy” OR “Endotracheal occlusion” OR “Hydrothorax” OR “Laser” OR “Lower urinary tract obstruction”; OR “LUTO” OR “Mediastinal teratoma”; OR “Meningomyelocele” OR “Micrognathia” OR “MMC” OR “Myelomeningocele” OR “Neck mass” OR “RFA” OR “Sacrococcygeal teratoma” OR “Spina bifida” OR “Teratoma” OR “Uterocele”].

“Delivery” OR “C(a)esarean AND: [“BPS” OR “Bronchopulmonary sequestration” OR “Cervical lymphangioma” OR “Cervical teratoma” OR “CHAOS” OR “Chest mass” OR “Congenital high airway obstruction syndrome” OR “Mediastinal teratoma” OR “Micrognathia” OR “Neck mass”].

Supplement 2: Classification of maternal surgical complications (Dindo et al 2004^12^)

| Grade | Definition |
| --- | --- |
| I | Any deviation from the normal postoperative course without the need for pharmacological treatment or surgical, endoscopic or radiological interventions. Allowed treatments: antiemetics, antipyretics, analgesics, diuretics, physiotherapy, wound infections opened at the bedside. |
| II | Requiring pharmacological treatment with drugs other than those allowed for grade I complications, including blood transfusion |
| III   - IIIa - IIIb | Requiring surgical, endoscopic or radiological intervention  Intervention not under general anaesthesia  Intervention under general anaesthesia |
| IV   - IVa - IVb | Life-threatening complications requiring Intensive Care Unit management  Single organ dysfunction (including dialysis)  Multiorgan dysfunction |
| V | Death of a patient |

Supplement 3: Summary of risk of bias according to study type.

Tools used to assess the risk of bias: Cochrane Collaboration tool^13^ for randomised trials, Newcastle-Ottawa scale^14^ for case control studies, National Institutes of Health study tool^15^ for case series.

Supplement 4: Statistical heterogeneity according to outcome analysed.

| Category | Surgery type | Outcome | Heterogeneity (I^2^) | 95% CI for I^2^ |
| --- | --- | --- | --- | --- |
| Intraoperative | Open | Delivery/ termination | 0.00 | 0.00-0.00 |
|  | Fetoscopic | Delivery/ termination | 0.00 | 0.00-0.00 |
|  | Open | Placental abruption | 0.00 | 0.00-0.00 |
|  | Fetoscopic | Placental abruption | 0.00 | 0.00-0.00 |
|  | Open | Bleeding | 40.38 | 13.92-58.71 |
|  | Fetoscopic | Bleeding | 68.42 | 61.97-73.77 |
|  | Open | Blood transfusion | 0.00 | 0.00-0.00 |
|  | Fetoscopic | Blood transfusion | 0.00 | 0.00-0.00 |
|  | Fetoscopic | Skin burns at site of diathermy pads | 0.00 | 0.00-0.00 |
| Postoperative | Fetoscopic | Delivery/ termination in 24hrs following procedure | 0.00 | 0.00-0.00 |
|  | Open | Placental abruption | 0.00 | 0.00-28.35 |
|  | Fetoscopic | Placental abruption | 61.59 | 53.24-68.45 |
|  | Open | Blood transfusion | 53.27 | 33.89-66.97 |
|  | Fetoscopic | Blood transfusion | 0.00 | 0.00-0.00 |
|  | Open | Chorioamnionitis | 4.62 | 0.00-32.00 |
|  | Fetoscopic | Chorioamnionitis | 56.71 | 47.02-64.63 |
|  | Open | Chorioamnionitis with PPROM | 76.26 | 62.15-85.11 |
|  | Fetoscopic | Chorioamnionitis with PPROM | 73.14 | 63.18-80.41 |
|  | Open | Pulmonary oedema | 67.07 | 54.77-76.02 |
|  | Fetoscopic | Pulmonary oedema | 30.92 | 13.31-44.95 |
| Delivery | Open | Uterine rupture | 0.00 | 0.00-1.64 |
|  | Open | Uterine dehiscence | 48.49 | 16.39-68.26 |
|  | Open | Blood transfusion | 0.00 | 0.00-0.00 |
| Combined outcomes | Open | Severe complications | 14.06 | 0.00-41.38 |
|  | Open | Minor complications | 82.19 | 76.71-86.38 |
|  | Open | All complications | 81.79 | 76.15-86.10 |
|  | Fetoscopic | Severe complications | 65.95 | 58.84-71.83 |
|  | Fetoscopic | Minor complications | 81.57 | 78.37-84.30 |
|  | Fetoscopic | All complications | 82.46 | 79.47-85.02 |
| EXIT | Open | Severe complications | 0.00 | 0.00-0.00 |
|  | Open | Minor complications | 65.91 | 45.38-78.72 |
|  | Open | All complications | 65.52 | 44.68-78.51 |
| MMC | Open | Severe complications | 44.59 | 0.00-70.35 |
|  | Open | Minor complications | 66.03 | 40.27-80.69 |
|  | Open | All complications | 61.72 | 31.59-78.58 |
| MMC | Fetoscopic | Severe complications | 50.24 | 0.00-76.75 |
|  | Fetoscopic | Minor complications | 72.99 | 47.18-86.19 |
|  | Fetoscopic | All complications | 78.61 | 59.71-88.64 |
| CDH | Fetoscopic | Severe complications | 32.98 | 0.00-65.36 |
|  | Fetoscopic | Minor complications | 57.00 | 20.20-76.83 |
|  | Fetoscopic | All complications | 72.69 | 52.39-84.33 |
| Laser photocoagulation | Fetoscopic | Severe complications | 75.99 | 69.35-81.20 |
|  | Fetoscopic | Minor complications | 86.79 | 83.76-89.25 |
|  | Fetoscopic | All complications | 85.91 | 82.61-88.58 |
| Pregnancy reduction | Fetoscopic | Severe complications | 50.11 | 22.82-67.75 |
|  | Fetoscopic | Minor complications | 53.11 | 27.90-69.50 |
|  | Fetoscopic | All complications | 71.06 | 57.65-80.23 |
| Late | Open | Subfertility | 36.68 | 0.00-78.14 |
|  | Open | Miscarriage | 0.00 | 0.00-92.63 |
|  | Fetoscopic | Miscarriage | 0.00 | 0.00-92.94 |
|  | Open | Preterm birth | 60.30 | 0.00-86.74 |
|  | Fetoscopic | Preterm birth | 80.78 | 39.78-93.87 |
|  | Open | Uterine rupture | 43.11 | 0.00-82.90 |
|  | Open | Uterine dehiscence | 0.00 | 0.00-0.00 |
|  | Open | Attempting pregnancy | 93.20 | 83.48-97.20 |
|  | Fetoscopic | Attempting pregnancy | 95.87 | 88.23-98.55 |
|  | Open | Achieving pregnancy | 90.75 | 79.37-95.85 |
|  | Fetoscopic | Achieving pregnancy | 89.27 | 70.83-96.05 |
|  | Open | Bleeding at delivery | 0.00 | 0.00-0.00 |
|  | Fetoscopic | Bleeding at delivery | 0.00 | 0.00-53.90 |
|  | Fetoscopic | Abdominal pain | 54.58 | 0.00-88.89 |
|  | Fetoscopic | Gynaecological abnormal bleeding | 0.00 | 0.00-0.00 |
|  | Open | Gynaecological surgery | 55.46 | 0.00-89.20 |
|  | Fetoscopic | Psychological symptoms | 95.74 | 87.72-98.52 |

PPROM - preterm prelabour rupture of membranes, EXIT - ex-utero intrapartum treatment, MMC - myelomeningocele, CDH - congenital diaphragmatic hernia,
