# Supplementary material for: Maternal complications following open and fetoscopic fetal surgery: A systematic review and meta‐analysis
Source: Prenat Diagn. 2019 Feb 27;39(4):251–68. doi: 10.1002/pd.5421 (PMC6492015; doi:10.1002/pd.5421)
Supplement: Supplementary file 2 — Data S2. Supporting information [file PD-39-251-s002.docx]

# Meta-analysis: intraoperative open delivery

| Variable for studies | Study |
| --- | --- |
| Variable for total number of cases | N |
| Variable for number of positive cases | Outcome |

| Study | Sample size | Proportion (%) | 95% CI | Weight (%) | |
| --- | --- | --- | --- | --- | --- |
|  |  |  |  | Fixed | Random |
| Barthod 2013 (n=5) | 5 | 0,000 | 0,000 to 52,182 | 0,49 | 0,49 |
| Cass 2013 (n=9) | 9 | 0,000 | 0,000 to 33,627 | 0,81 | 0,81 |
| Chen 2018 (n=7) | 7 | 0,000 | 0,000 to 40,962 | 0,65 | 0,65 |
| Dahlgren 2004 (n=4) | 4 | 0,000 | 0,000 to 60,236 | 0,40 | 0,40 |
| Flake 2000 (n=15) | 15 | 0,000 | 0,000 to 21,802 | 1,29 | 1,29 |
| George 2007 (n=3) | 3 | 0,000 | 0,000 to 70,760 | 0,32 | 0,32 |
| Hedrick 2003 (n=43) | 43 | 0,000 | 0,000 to 8,221 | 3,56 | 3,56 |
| Hedrick 2005 (n=9) | 9 | 0,000 | 0,000 to 33,627 | 0,81 | 0,81 |
| Kern 2007 (n=5) | 5 | 0,000 | 0,000 to 52,182 | 0,49 | 0,49 |
| Kornacki 2017 (n=4) | 4 | 0,000 | 0,000 to 60,236 | 0,40 | 0,40 |
| Kunisaki 2007 (n=14) | 14 | 0,000 | 0,000 to 23,164 | 1,21 | 1,21 |
| Laje 2012 (n=17) | 17 | 0,000 | 0,000 to 19,506 | 1,46 | 1,46 |
| Laje 2013 (n=4) | 4 | 0,000 | 0,000 to 60,236 | 0,40 | 0,40 |
| Laje 2015 (n=13) | 13 | 0,000 | 0,000 to 24,705 | 1,13 | 1,13 |
| Lazar 2011 (n=12) | 12 | 0,000 | 0,000 to 26,465 | 1,05 | 1,05 |
| Noah 2002 (n=34) | 34 | 0,000 | 0,000 to 10,282 | 2,83 | 2,83 |
| Pellicer 2007 (n=3) | 3 | 0,000 | 0,000 to 70,760 | 0,32 | 0,32 |
| Stoffan 2012 (n=7) | 7 | 0,000 | 0,000 to 40,962 | 0,65 | 0,65 |
| Tuncay Ozgunen 2010 (n=3) | 3 | 0,000 | 0,000 to 70,760 | 0,32 | 0,32 |
| Zamora 2013 (n=26) | 26 | 0,000 | 0,000 to 13,227 | 2,18 | 2,18 |
| Bennett 2014 (n=43) | 43 | 0,000 | 0,000 to 8,221 | 3,56 | 3,56 |
| Botelho 2017 (n=45) | 45 | 0,000 | 0,000 to 7,871 | 3,72 | 3,72 |
| Bruner 1999 (n=29) | 29 | 3,448 | 0,0873 to 17,764 | 2,43 | 2,43 |
| Bruner 2000 (n=4) | 4 | 0,000 | 0,000 to 60,236 | 0,40 | 0,40 |
| Farmer 2003 (n=12) | 12 | 0,000 | 0,000 to 26,465 | 1,05 | 1,05 |
| Friszer 2016 (n=3) | 3 | 0,000 | 0,000 to 70,760 | 0,32 | 0,32 |
| Johnson 2016 (n=91) | 91 | 0,000 | 0,000 to 3,973 | 7,44 | 7,44 |
| Marenco 2013 (n=4) | 4 | 0,000 | 0,000 to 60,236 | 0,40 | 0,40 |
| Moldenhauer 2015 (n=100) | 100 | 0,000 | 0,000 to 3,622 | 8,17 | 8,17 |
| Moron 2018 (n=237) | 237 | 0,422 | 0,0107 to 2,328 | 19,26 | 19,26 |
| Ochsenbein-Kolble 2017 (n=30) | 30 | 3,333 | 0,0844 to 17,217 | 2,51 | 2,51 |
| Sinskey 2017 (n=47) | 47 | 0,000 | 0,000 to 7,549 | 3,88 | 3,88 |
| Soni 2016 (n=88) | 88 | 0,000 | 0,000 to 4,105 | 7,20 | 7,20 |
| Zamlynski 2014 (n=46) | 46 | 0,000 | 0,000 to 7,706 | 3,80 | 3,80 |
| Flake 2000 (n=15) | 15 | 0,000 | 0,000 to 21,802 | 1,29 | 1,29 |
| Harrison 1990 (n=6) | 6 | 0,000 | 0,000 to 45,926 | 0,57 | 0,57 |
| Harrison 1993 (n=14) | 14 | 0,000 | 0,000 to 23,164 | 1,21 | 1,21 |
| Harrison 1998 (n=13) | 13 | 0,000 | 0,000 to 24,705 | 1,13 | 1,13 |
| Adzick 2003 (n=22) | 22 | 0,000 | 0,000 to 15,437 | 1,86 | 1,86 |
| Hedrick 2004 (n=4) | 4 | 0,000 | 0,000 to 60,236 | 0,40 | 0,40 |
| Golombeck 2006 (n=79) | 79 | 0,000 | 0,000 to 4,562 | 6,47 | 6,47 |
| Longaker 1991 (n=17) | 17 | 0,000 | 0,000 to 19,506 | 1,46 | 1,46 |
| Zamora 2013 (n=7) | 7 | 0,000 | 0,000 to 40,962 | 0,65 | 0,65 |
| Total (fixed effects) | 1193 | 0,915 | 0,462 to 1,618 | 100,00 | 100,00 |
| Total (random effects) | 1193 | 0,915 | 0,460 to 1,521 | 100,00 | 100,00 |

## Test for heterogeneity

| Q | 13,8863 |
| --- | --- |
| DF | 42 |
| Significance level | P = 1,0000 |
| I^2^ (inconsistency) | 0,00% |
| 95% CI for I^2^ | 0,00 to 0,00 |

# Meta-analysis: intraoperative fetoscopic delivery

| Variable for studies | Study |
| --- | --- |
| Variable for total number of cases | N |
| Variable for number of positive cases | Outcome |

| Study | Sample size | Proportion (%) | 95% CI | Weight (%) | |
| --- | --- | --- | --- | --- | --- |
|  |  |  |  | Fixed | Random |
| Aboudiab 2017 (n=18) | 18 | 0,000 | 0,000 to 18,530 | 0,20 | 0,20 |
| Baschat 2013 (n=147) | 147 | 0,000 | 0,000 to 2,478 | 1,55 | 1,55 |
| Chalouhi 2016 (n=22) | 22 | 0,000 | 0,000 to 15,437 | 0,24 | 0,24 |
| Chang 2006 (n=27) | 27 | 3,704 | 0,0937 to 18,971 | 0,29 | 0,29 |
| Chang 2016 (n=100) | 100 | 0,000 | 0,000 to 3,622 | 1,06 | 1,06 |
| Chmait 2013 (n=318) | 318 | 0,000 | 0,000 to 1,153 | 3,35 | 3,35 |
| Chmait 2017 (n=19) | 19 | 0,000 | 0,000 to 17,647 | 0,21 | 0,21 |
| Crombleholme 2007 (n=20) | 20 | 0,000 | 0,000 to 16,843 | 0,22 | 0,22 |
| De Lia 1995 (n=26) | 26 | 0,000 | 0,000 to 13,227 | 0,28 | 0,28 |
| De Lia 1999 (n=67) | 67 | 0,000 | 0,000 to 5,357 | 0,71 | 0,71 |
| De Lia 2009 (n=10) | 10 | 0,000 | 0,000 to 30,850 | 0,12 | 0,12 |
| Deprest 1998 (n=6) | 6 | 0,000 | 0,000 to 45,926 | 0,073 | 0,073 |
| Draga 2016 (n=37) | 37 | 0,000 | 0,000 to 9,489 | 0,40 | 0,40 |
| Duron 2014 (n=85) | 85 | 0,000 | 0,000 to 4,247 | 0,90 | 0,90 |
| Ek 2012 (n=) | 67 | 0,000 | 0,000 to 5,357 | 0,71 | 0,71 |
| Habli 2009 (n=152) | 152 | 0,000 | 0,000 to 2,398 | 1,61 | 1,61 |
| Has 2014 (n=85) | 85 | 0,000 | 0,000 to 4,247 | 0,90 | 0,90 |
| Hecher 2000 (n=200) | 200 | 0,000 | 0,000 to 1,828 | 2,11 | 2,11 |
| Hernandez-Andrade 2011 (n=35) | 35 | 0,000 | 0,000 to 10,003 | 0,38 | 0,38 |
| Huber 2008 (n=176) | 176 | 0,568 | 0,0144 to 3,125 | 1,86 | 1,86 |
| Ishii 2014 (n=16) | 16 | 0,000 | 0,000 to 20,591 | 0,18 | 0,18 |
| Ishii 2015 (n=10) | 10 | 0,000 | 0,000 to 30,850 | 0,12 | 0,12 |
| Lanna 2017 (n=373) | 373 | 0,000 | 0,000 to 0,984 | 3,93 | 3,93 |
| Lecointre 2017 (n=200) | 200 | 0,000 | 0,000 to 1,828 | 2,11 | 2,11 |
| Malshe 2017 (n=203) | 203 | 0,000 | 0,000 to 1,801 | 2,14 | 2,14 |
| Martinez 2012 (n=500) | 500 | 0,000 | 0,000 to 0,735 | 5,26 | 5,26 |
| Middeldorp 2007 (n=100) | 100 | 0,000 | 0,000 to 3,622 | 1,06 | 1,06 |
| Miyadahira 2018 (n=67) | 67 | 0,000 | 0,000 to 5,357 | 0,71 | 0,71 |
| Molina-Garcia 2009 (n=22) | 22 | 0,000 | 0,000 to 15,437 | 0,24 | 0,24 |
| Morris 2010 (n=164) | 164 | 0,000 | 0,000 to 2,224 | 1,73 | 1,73 |
| Mullers 2015 (n=105) | 105 | 0,000 | 0,000 to 3,452 | 1,11 | 1,11 |
| Nakata 2016 (n=6) | 6 | 0,000 | 0,000 to 45,926 | 0,073 | 0,073 |
| Nguyen 2012 (n=98) | 98 | 0,000 | 0,000 to 3,694 | 1,04 | 1,04 |
| Ozawa 2017 (n=11) | 11 | 0,000 | 0,000 to 28,491 | 0,13 | 0,13 |
| Papanna 2010 (n=48) | 48 | 0,000 | 0,000 to 7,397 | 0,51 | 0,51 |
| Papanna 2012 (n=163) | 163 | 0,000 | 0,000 to 2,238 | 1,72 | 1,72 |
| Peeters 2014 (n=338) | 338 | 0,000 | 0,000 to 1,085 | 3,56 | 3,56 |
| Persico 2016 (n=106) | 106 | 0,000 | 0,000 to 3,420 | 1,12 | 1,12 |
| Quintero 2000 (n=92) | 92 | 0,000 | 0,000 to 3,930 | 0,98 | 0,98 |
| Quintero 2001 (n=11) | 11 | 0,000 | 0,000 to 28,491 | 0,13 | 0,13 |
| Rossi 2008 (n=266) | 266 | 0,000 | 0,000 to 1,377 | 2,80 | 2,80 |
| Ruano 2009 (n=19) | 19 | 0,000 | 0,000 to 17,647 | 0,21 | 0,21 |
| Ruegg 2018 (n=37) | 37 | 0,000 | 0,000 to 9,489 | 0,40 | 0,40 |
| Rustico 2012 (n=150) | 150 | 0,000 | 0,000 to 2,429 | 1,59 | 1,59 |
| Said 2008 (n=10) | 10 | 0,000 | 0,000 to 30,850 | 0,12 | 0,12 |
| Senat 2004 (n=72) | 72 | 0,000 | 0,000 to 4,994 | 0,77 | 0,77 |
| Sepulveda 2007 (n=33) | 33 | 0,000 | 0,000 to 10,576 | 0,36 | 0,36 |
| Shamshirsaz 2015 (n=55) | 55 | 0,000 | 0,000 to 6,487 | 0,59 | 0,59 |
| Slaghekke 2014 (n=274) | 274 | 0,000 | 0,000 to 1,337 | 2,89 | 2,89 |
| Taniguchi 2015 (n=3) | 3 | 0,000 | 0,000 to 70,760 | 0,042 | 0,042 |
| Tchirikov 2011 (n=80) | 80 | 0,000 | 0,000 to 4,506 | 0,85 | 0,85 |
| Teoh 2013 (n=49) | 49 | 0,000 | 0,000 to 7,252 | 0,52 | 0,52 |
| Thia 2017 (n=5) | 5 | 0,000 | 0,000 to 52,182 | 0,063 | 0,063 |
| Ville 1997 (n=132) | 132 | 0,000 | 0,000 to 2,756 | 1,40 | 1,40 |
| Ville 1998 (n=44) | 44 | 0,000 | 0,000 to 8,042 | 0,47 | 0,47 |
| Weingertner 2011 (n=100) | 100 | 0,000 | 0,000 to 3,622 | 1,06 | 1,06 |
| Wilson 2016 (n=151) | 151 | 0,000 | 0,000 to 2,413 | 1,60 | 1,60 |
| Yamamoto 2005 (n=175) | 175 | 0,000 | 0,000 to 2,086 | 1,85 | 1,85 |
| Yang 2010 (n=30) | 30 | 0,000 | 0,000 to 11,570 | 0,33 | 0,33 |
| Zaretsky 2018 (n=749) | 749 | 0,000 | 0,000 to 0,491 | 7,87 | 7,87 |
| Zhao 2016 (n=62) | 62 | 0,000 | 0,000 to 5,776 | 0,66 | 0,66 |
| Bebbington 2012 (n=146) | 146 | 0,000 | 0,000 to 2,495 | 1,54 | 1,54 |
| Berg 2014 (n=7) | 7 | 0,000 | 0,000 to 40,962 | 0,084 | 0,084 |
| Delabaere 2013 (n=30) | 30 | 3,333 | 0,0844 to 17,217 | 0,33 | 0,33 |
| Deprest 2000 (n=10) | 10 | 0,000 | 0,000 to 30,850 | 0,12 | 0,12 |
| Gallot 2003 (n=11) | 11 | 9,091 | 0,230 to 41,278 | 0,13 | 0,13 |
| Gouverneur 2009 (n=54) | 54 | 0,000 | 0,000 to 6,603 | 0,58 | 0,58 |
| Gul 2008 (n=9) | 9 | 0,000 | 0,000 to 33,627 | 0,10 | 0,10 |
| Has 2014 (n=71) | 71 | 0,000 | 0,000 to 5,063 | 0,76 | 0,76 |
| He 2010 (n=14) | 14 | 0,000 | 0,000 to 23,164 | 0,16 | 0,16 |
| Ilagan 2008 (n=27) | 27 | 0,000 | 0,000 to 12,770 | 0,29 | 0,29 |
| Jelin 2010 (n=7) | 7 | 0,000 | 0,000 to 40,962 | 0,084 | 0,084 |
| King 2017 (n=43) | 43 | 0,000 | 0,000 to 8,221 | 0,46 | 0,46 |
| Lanna 2012 (n=118) | 118 | 0,000 | 0,000 to 3,078 | 1,25 | 1,25 |
| Lee 2013 (n=98) | 98 | 0,000 | 0,000 to 3,694 | 1,04 | 1,04 |
| Lewi 2006 (n=80) | 80 | 0,000 | 0,000 to 4,506 | 0,85 | 0,85 |
| Moise 2008 (n=9) | 9 | 0,000 | 0,000 to 33,627 | 0,10 | 0,10 |
| Nobili 2013 (n=48) | 48 | 0,000 | 0,000 to 7,397 | 0,51 | 0,51 |
| Paramasivam 2010 (n=35) | 35 | 0,000 | 0,000 to 10,003 | 0,38 | 0,38 |
| Peng 2016 (n=93) | 93 | 0,000 | 0,000 to 3,889 | 0,99 | 0,99 |
| Quintero 1996 (n=13) | 13 | 0,000 | 0,000 to 24,705 | 0,15 | 0,15 |
| Quintero 2006 (n=51) | 51 | 0,000 | 0,000 to 6,978 | 0,55 | 0,55 |
| Roman 2010 (n=60) | 60 | 0,000 | 0,000 to 5,963 | 0,64 | 0,64 |
| Schou 2018 (n=102) | 102 | 0,000 | 0,000 to 3,552 | 1,08 | 1,08 |
| Sugibayashi 2016 (n=40) | 40 | 0,000 | 0,000 to 8,810 | 0,43 | 0,43 |
| Takano 2015 (n=10) | 10 | 0,000 | 0,000 to 30,850 | 0,12 | 0,12 |
| Taylor 2002 (n=15) | 15 | 0,000 | 0,000 to 21,802 | 0,17 | 0,17 |
| Tsao 2002 (n=13) | 13 | 0,000 | 0,000 to 24,705 | 0,15 | 0,15 |
| Zhang 2018 (n=25) | 25 | 0,000 | 0,000 to 13,719 | 0,27 | 0,27 |
| Deprest 2005 (n=20) | 20 | 0,000 | 0,000 to 16,843 | 0,22 | 0,22 |
| Harrison 1998 (n=8) | 8 | 0,000 | 0,000 to 36,942 | 0,094 | 0,094 |
| Harrison 2003 (n=11) | 11 | 0,000 | 0,000 to 28,491 | 0,13 | 0,13 |
| Jani 2005 (n=24) | 24 | 0,000 | 0,000 to 14,247 | 0,26 | 0,26 |
| Jani 2006 (n=28) | 28 | 0,000 | 0,000 to 12,344 | 0,30 | 0,30 |
| Jani 2009 (n=210) | 210 | 0,000 | 0,000 to 1,741 | 2,22 | 2,22 |
| Jimenez 2017 (n=201) | 201 | 0,000 | 0,000 to 1,819 | 2,12 | 2,12 |
| Kosinski 2017 (n=28) | 28 | 0,000 | 0,000 to 12,344 | 0,30 | 0,30 |
| Manrique 2008 (n=11) | 11 | 0,000 | 0,000 to 28,491 | 0,13 | 0,13 |
| Peralta 2011 (n=8) | 8 | 0,000 | 0,000 to 36,942 | 0,094 | 0,094 |
| Persico 2017 (n=21) | 21 | 0,000 | 0,000 to 16,110 | 0,23 | 0,23 |
| Ruano 2012 (n=35) | 35 | 0,000 | 0,000 to 10,003 | 0,38 | 0,38 |
| Ruano 2012 (n=20) | 20 | 0,000 | 0,000 to 16,843 | 0,22 | 0,22 |
| Ruano 2013 (n=17) | 17 | 0,000 | 0,000 to 19,506 | 0,19 | 0,19 |
| Arens 2017 (n=59) | 59 | 0,000 | 0,000 to 6,061 | 0,63 | 0,63 |
| Belfort 2017 (n=22) | 22 | 0,000 | 0,000 to 15,437 | 0,24 | 0,24 |
| Bruner 2000 (n=4) | 4 | 0,000 | 0,000 to 60,236 | 0,052 | 0,052 |
| Degenhardt 2014 (n=51) | 51 | 0,000 | 0,000 to 6,978 | 0,55 | 0,55 |
| Kohn 2018 (n=34) | 34 | 0,000 | 0,000 to 10,282 | 0,37 | 0,37 |
| Pedreira 2014 (n=4) | 4 | 0,000 | 0,000 to 60,236 | 0,052 | 0,052 |
| Pedreira 2016 (n=10) | 10 | 0,000 | 0,000 to 30,850 | 0,12 | 0,12 |
| Verbeek 2012 (n=19) | 19 | 0,000 | 0,000 to 17,647 | 0,21 | 0,21 |
| Ziemann 2018 (n=65) | 65 | 0,000 | 0,000 to 5,517 | 0,69 | 0,69 |
| Morris 2013 (n=16) | 16 | 0,000 | 0,000 to 20,591 | 0,18 | 0,18 |
| Ruano 2010 (n=11) | 11 | 0,000 | 0,000 to 28,491 | 0,13 | 0,13 |
| Welsh 2003 (n=13) | 13 | 0,000 | 0,000 to 24,705 | 0,15 | 0,15 |
| Cavalheiro 2011 (n=30) | 30 | 0,000 | 0,000 to 11,570 | 0,33 | 0,33 |
| Mallman 2017 (n=78) | 78 | 0,000 | 0,000 to 4,619 | 0,83 | 0,83 |
| Golombeck 2006 (n=99) | 99 | 0,000 | 0,000 to 3,658 | 1,05 | 1,05 |
| Kohl 2006 (n=16) | 16 | 0,000 | 0,000 to 20,591 | 0,18 | 0,18 |
| Kohl 2010 (n=37) | 37 | 2,703 | 0,0684 to 14,160 | 0,40 | 0,40 |
| Nivatpumin 2016 (n=152) | 152 | 0,000 | 0,000 to 2,398 | 1,61 | 1,61 |
| Peralta 2010 (n=56) | 56 | 0,000 | 0,000 to 6,375 | 0,60 | 0,60 |
| Total (fixed effects) | 9403 | 0,263 | 0,170 to 0,388 | 100,00 | 100,00 |
| Total (random effects) | 9403 | 0,263 | 0,170 to 0,376 | 100,00 | 100,00 |

## Test for heterogeneity

| Q | 48,1709 |
| --- | --- |
| DF | 121 |
| Significance level | P = 1,0000 |
| I^2^ (inconsistency) | 0,00% |
| 95% CI for I^2^ | 0,00 to 0,00 |

# Meta-analysis: intraoperative open abruption

| Variable for studies | Study |
| --- | --- |
| Variable for total number of cases | N |
| Variable for number of positive cases | Outcome |

| Study | Sample size | Proportion (%) | 95% CI | Weight (%) | |
| --- | --- | --- | --- | --- | --- |
|  |  |  |  | Fixed | Random |
| Barthod 2013 (n=5) | 5 | 0,000 | 0,000 to 52,182 | 0,49 | 0,49 |
| Cass 2013 (n=9) | 9 | 0,000 | 0,000 to 33,627 | 0,81 | 0,81 |
| Chen 2018 (n=7) | 7 | 0,000 | 0,000 to 40,962 | 0,65 | 0,65 |
| Dahlgren 2004 (n=4) | 4 | 0,000 | 0,000 to 60,236 | 0,40 | 0,40 |
| Flake 2000 (n=15) | 15 | 0,000 | 0,000 to 21,802 | 1,29 | 1,29 |
| George 2007 (n=3) | 3 | 0,000 | 0,000 to 70,760 | 0,32 | 0,32 |
| Hedrick 2003 (n=43) | 43 | 2,326 | 0,0589 to 12,289 | 3,56 | 3,56 |
| Hedrick 2005 (n=9) | 9 | 0,000 | 0,000 to 33,627 | 0,81 | 0,81 |
| Kern 2007 (n=5) | 5 | 0,000 | 0,000 to 52,182 | 0,49 | 0,49 |
| Kornacki 2017 (n=4) | 4 | 0,000 | 0,000 to 60,236 | 0,40 | 0,40 |
| Kunisaki 2007 (n=14) | 14 | 0,000 | 0,000 to 23,164 | 1,21 | 1,21 |
| Laje 2012 (n=17) | 17 | 5,882 | 0,149 to 28,689 | 1,46 | 1,46 |
| Laje 2013 (n=4) | 4 | 0,000 | 0,000 to 60,236 | 0,40 | 0,40 |
| Laje 2015 (n=13) | 13 | 0,000 | 0,000 to 24,705 | 1,13 | 1,13 |
| Lazar 2011 (n=12) | 12 | 0,000 | 0,000 to 26,465 | 1,05 | 1,05 |
| Noah 2002 (n=34) | 34 | 0,000 | 0,000 to 10,282 | 2,83 | 2,83 |
| Pellicer 2007 (n=3) | 3 | 33,333 | 0,840 to 90,570 | 0,32 | 0,32 |
| Stoffan 2012 (n=7) | 7 | 14,286 | 0,361 to 57,872 | 0,65 | 0,65 |
| Tuncay Ozgunen 2010 (n=3) | 3 | 0,000 | 0,000 to 70,760 | 0,32 | 0,32 |
| Zamora 2013 (n=26) | 26 | 0,000 | 0,000 to 13,227 | 2,18 | 2,18 |
| Bennett 2014 (n=43) | 43 | 2,326 | 0,0589 to 12,289 | 3,56 | 3,56 |
| Botelho 2017 (n=45) | 45 | 0,000 | 0,000 to 7,871 | 3,72 | 3,72 |
| Bruner 1999 (n=29) | 29 | 3,448 | 0,0873 to 17,764 | 2,43 | 2,43 |
| Bruner 2000 (n=4) | 4 | 0,000 | 0,000 to 60,236 | 0,40 | 0,40 |
| Farmer 2003 (n=12) | 12 | 0,000 | 0,000 to 26,465 | 1,05 | 1,05 |
| Friszer 2016 (n=3) | 3 | 0,000 | 0,000 to 70,760 | 0,32 | 0,32 |
| Johnson 2016 (n=91) | 91 | 0,000 | 0,000 to 3,973 | 7,44 | 7,44 |
| Marenco 2013 (n=4) | 4 | 0,000 | 0,000 to 60,236 | 0,40 | 0,40 |
| Moldenhauer 2015 (n=100) | 100 | 0,000 | 0,000 to 3,622 | 8,17 | 8,17 |
| Moron 2018 (n=237) | 237 | 0,844 | 0,102 to 3,015 | 19,26 | 19,26 |
| Ochsenbein-Kolble 2017 (n=30) | 30 | 3,333 | 0,0844 to 17,217 | 2,51 | 2,51 |
| Sinskey 2017 (n=47) | 47 | 0,000 | 0,000 to 7,549 | 3,88 | 3,88 |
| Soni 2016 (n=88) | 88 | 0,000 | 0,000 to 4,105 | 7,20 | 7,20 |
| Zamlynski 2014 (n=46) | 46 | 0,000 | 0,000 to 7,706 | 3,80 | 3,80 |
| Flake 2000 (n=15) | 15 | 0,000 | 0,000 to 21,802 | 1,29 | 1,29 |
| Harrison 1990 (n=6) | 6 | 0,000 | 0,000 to 45,926 | 0,57 | 0,57 |
| Harrison 1993 (n=14) | 14 | 0,000 | 0,000 to 23,164 | 1,21 | 1,21 |
| Harrison 1998 (n=13) | 13 | 0,000 | 0,000 to 24,705 | 1,13 | 1,13 |
| Adzick 2003 (n=22) | 22 | 0,000 | 0,000 to 15,437 | 1,86 | 1,86 |
| Hedrick 2004 (n=4) | 4 | 0,000 | 0,000 to 60,236 | 0,40 | 0,40 |
| Golombeck 2006 (n=79) | 79 | 0,000 | 0,000 to 4,562 | 6,47 | 6,47 |
| Longaker 1991 (n=17) | 17 | 0,000 | 0,000 to 19,506 | 1,46 | 1,46 |
| Zamora 2013 (n=7) | 7 | 0,000 | 0,000 to 40,962 | 0,65 | 0,65 |
| Total (fixed effects) | 1193 | 1,282 | 0,732 to 2,078 | 100,00 | 100,00 |
| Total (random effects) | 1193 | 1,282 | 0,731 to 1,984 | 100,00 | 100,00 |

## Test for heterogeneity

| Q | 25,4476 |
| --- | --- |
| DF | 42 |
| Significance level | P = 0,9795 |
| I^2^ (inconsistency) | 0,00% |
| 95% CI for I^2^ | 0,00 to 0,00 |

# Meta-analysis: intraoperative fetoscopic abruption

| Variable for studies | Study |
| --- | --- |
| Variable for total number of cases | N |
| Variable for number of positive cases | Outcome |

| Study | Sample size | Proportion (%) | 95% CI | Weight (%) | |
| --- | --- | --- | --- | --- | --- |
|  |  |  |  | Fixed | Random |
| Aboudiab 2017 (n=18) | 18 | 0,000 | 0,000 to 18,530 | 0,20 | 0,20 |
| Baschat 2013 (n=147) | 147 | 0,000 | 0,000 to 2,478 | 1,55 | 1,55 |
| Chalouhi 2016 (n=22) | 22 | 0,000 | 0,000 to 15,437 | 0,24 | 0,24 |
| Chang 2006 (n=27) | 27 | 0,000 | 0,000 to 12,770 | 0,29 | 0,29 |
| Chang 2016 (n=100) | 100 | 0,000 | 0,000 to 3,622 | 1,06 | 1,06 |
| Chmait 2013 (n=318) | 318 | 0,000 | 0,000 to 1,153 | 3,35 | 3,35 |
| Chmait 2017 (n=19) | 19 | 0,000 | 0,000 to 17,647 | 0,21 | 0,21 |
| Crombleholme 2007 (n=20) | 20 | 0,000 | 0,000 to 16,843 | 0,22 | 0,22 |
| De Lia 1995 (n=26) | 26 | 0,000 | 0,000 to 13,227 | 0,28 | 0,28 |
| De Lia 1999 (n=67) | 67 | 0,000 | 0,000 to 5,357 | 0,71 | 0,71 |
| De Lia 2009 (n=10) | 10 | 0,000 | 0,000 to 30,850 | 0,12 | 0,12 |
| Deprest 1998 (n=6) | 6 | 0,000 | 0,000 to 45,926 | 0,073 | 0,073 |
| Draga 2016 (n=37) | 37 | 0,000 | 0,000 to 9,489 | 0,40 | 0,40 |
| Duron 2014 (n=85) | 85 | 0,000 | 0,000 to 4,247 | 0,90 | 0,90 |
| Ek 2012 (n=) | 67 | 0,000 | 0,000 to 5,357 | 0,71 | 0,71 |
| Habli 2009 (n=152) | 152 | 0,000 | 0,000 to 2,398 | 1,61 | 1,61 |
| Has 2014 (n=85) | 85 | 0,000 | 0,000 to 4,247 | 0,90 | 0,90 |
| Hecher 2000 (n=200) | 200 | 0,000 | 0,000 to 1,828 | 2,11 | 2,11 |
| Hernandez-Andrade 2011 (n=35) | 35 | 0,000 | 0,000 to 10,003 | 0,38 | 0,38 |
| Huber 2008 (n=176) | 176 | 0,000 | 0,000 to 2,074 | 1,86 | 1,86 |
| Ishii 2014 (n=16) | 16 | 0,000 | 0,000 to 20,591 | 0,18 | 0,18 |
| Ishii 2015 (n=10) | 10 | 0,000 | 0,000 to 30,850 | 0,12 | 0,12 |
| Lanna 2017 (n=373) | 373 | 0,000 | 0,000 to 0,984 | 3,93 | 3,93 |
| Lecointre 2017 (n=200) | 200 | 0,000 | 0,000 to 1,828 | 2,11 | 2,11 |
| Malshe 2017 (n=203) | 203 | 0,000 | 0,000 to 1,801 | 2,14 | 2,14 |
| Martinez 2012 (n=500) | 500 | 0,000 | 0,000 to 0,735 | 5,26 | 5,26 |
| Middeldorp 2007 (n=100) | 100 | 0,000 | 0,000 to 3,622 | 1,06 | 1,06 |
| Miyadahira 2018 (n=67) | 67 | 0,000 | 0,000 to 5,357 | 0,71 | 0,71 |
| Molina-Garcia 2009 (n=22) | 22 | 0,000 | 0,000 to 15,437 | 0,24 | 0,24 |
| Morris 2010 (n=164) | 164 | 0,000 | 0,000 to 2,224 | 1,73 | 1,73 |
| Mullers 2015 (n=105) | 105 | 0,000 | 0,000 to 3,452 | 1,11 | 1,11 |
| Nakata 2016 (n=6) | 6 | 0,000 | 0,000 to 45,926 | 0,073 | 0,073 |
| Nguyen 2012 (n=98) | 98 | 0,000 | 0,000 to 3,694 | 1,04 | 1,04 |
| Ozawa 2017 (n=11) | 11 | 0,000 | 0,000 to 28,491 | 0,13 | 0,13 |
| Papanna 2010 (n=48) | 48 | 2,083 | 0,0527 to 11,070 | 0,51 | 0,51 |
| Papanna 2012 (n=163) | 163 | 0,000 | 0,000 to 2,238 | 1,72 | 1,72 |
| Peeters 2014 (n=338) | 338 | 0,000 | 0,000 to 1,085 | 3,56 | 3,56 |
| Persico 2016 (n=106) | 106 | 0,000 | 0,000 to 3,420 | 1,12 | 1,12 |
| Quintero 2000 (n=92) | 92 | 0,000 | 0,000 to 3,930 | 0,98 | 0,98 |
| Quintero 2001 (n=11) | 11 | 0,000 | 0,000 to 28,491 | 0,13 | 0,13 |
| Rossi 2008 (n=266) | 266 | 0,000 | 0,000 to 1,377 | 2,80 | 2,80 |
| Ruano 2009 (n=19) | 19 | 0,000 | 0,000 to 17,647 | 0,21 | 0,21 |
| Ruegg 2018 (n=37) | 37 | 0,000 | 0,000 to 9,489 | 0,40 | 0,40 |
| Rustico 2012 (n=150) | 150 | 3,333 | 1,091 to 7,607 | 1,59 | 1,59 |
| Said 2008 (n=10) | 10 | 0,000 | 0,000 to 30,850 | 0,12 | 0,12 |
| Senat 2004 (n=72) | 72 | 0,000 | 0,000 to 4,994 | 0,77 | 0,77 |
| Sepulveda 2007 (n=33) | 33 | 0,000 | 0,000 to 10,576 | 0,36 | 0,36 |
| Shamshirsaz 2015 (n=55) | 55 | 0,000 | 0,000 to 6,487 | 0,59 | 0,59 |
| Slaghekke 2014 (n=274) | 274 | 0,000 | 0,000 to 1,337 | 2,89 | 2,89 |
| Taniguchi 2015 (n=3) | 3 | 0,000 | 0,000 to 70,760 | 0,042 | 0,042 |
| Tchirikov 2011 (n=80) | 80 | 0,000 | 0,000 to 4,506 | 0,85 | 0,85 |
| Teoh 2013 (n=49) | 49 | 0,000 | 0,000 to 7,252 | 0,52 | 0,52 |
| Thia 2017 (n=5) | 5 | 0,000 | 0,000 to 52,182 | 0,063 | 0,063 |
| Ville 1997 (n=132) | 132 | 0,000 | 0,000 to 2,756 | 1,40 | 1,40 |
| Ville 1998 (n=44) | 44 | 0,000 | 0,000 to 8,042 | 0,47 | 0,47 |
| Weingertner 2011 (n=100) | 100 | 0,000 | 0,000 to 3,622 | 1,06 | 1,06 |
| Wilson 2016 (n=151) | 151 | 0,000 | 0,000 to 2,413 | 1,60 | 1,60 |
| Yamamoto 2005 (n=175) | 175 | 0,000 | 0,000 to 2,086 | 1,85 | 1,85 |
| Yang 2010 (n=30) | 30 | 0,000 | 0,000 to 11,570 | 0,33 | 0,33 |
| Zaretsky 2018 (n=749) | 749 | 0,000 | 0,000 to 0,491 | 7,87 | 7,87 |
| Zhao 2016 (n=62) | 62 | 0,000 | 0,000 to 5,776 | 0,66 | 0,66 |
| Bebbington 2012 (n=146) | 146 | 0,000 | 0,000 to 2,495 | 1,54 | 1,54 |
| Berg 2014 (n=7) | 7 | 0,000 | 0,000 to 40,962 | 0,084 | 0,084 |
| Delabaere 2013 (n=30) | 30 | 0,000 | 0,000 to 11,570 | 0,33 | 0,33 |
| Deprest 2000 (n=10) | 10 | 0,000 | 0,000 to 30,850 | 0,12 | 0,12 |
| Gallot 2003 (n=11) | 11 | 0,000 | 0,000 to 28,491 | 0,13 | 0,13 |
| Gouverneur 2009 (n=54) | 54 | 0,000 | 0,000 to 6,603 | 0,58 | 0,58 |
| Gul 2008 (n=9) | 9 | 0,000 | 0,000 to 33,627 | 0,10 | 0,10 |
| Has 2014 (n=71) | 71 | 0,000 | 0,000 to 5,063 | 0,76 | 0,76 |
| He 2010 (n=14) | 14 | 0,000 | 0,000 to 23,164 | 0,16 | 0,16 |
| Ilagan 2008 (n=27) | 27 | 0,000 | 0,000 to 12,770 | 0,29 | 0,29 |
| Jelin 2010 (n=7) | 7 | 0,000 | 0,000 to 40,962 | 0,084 | 0,084 |
| King 2017 (n=43) | 43 | 0,000 | 0,000 to 8,221 | 0,46 | 0,46 |
| Lanna 2012 (n=118) | 118 | 0,000 | 0,000 to 3,078 | 1,25 | 1,25 |
| Lee 2013 (n=98) | 98 | 0,000 | 0,000 to 3,694 | 1,04 | 1,04 |
| Lewi 2006 (n=80) | 80 | 0,000 | 0,000 to 4,506 | 0,85 | 0,85 |
| Moise 2008 (n=9) | 9 | 0,000 | 0,000 to 33,627 | 0,10 | 0,10 |
| Nobili 2013 (n=48) | 48 | 0,000 | 0,000 to 7,397 | 0,51 | 0,51 |
| Paramasivam 2010 (n=35) | 35 | 0,000 | 0,000 to 10,003 | 0,38 | 0,38 |
| Peng 2016 (n=93) | 93 | 0,000 | 0,000 to 3,889 | 0,99 | 0,99 |
| Quintero 1996 (n=13) | 13 | 0,000 | 0,000 to 24,705 | 0,15 | 0,15 |
| Quintero 2006 (n=51) | 51 | 5,882 | 1,230 to 16,242 | 0,55 | 0,55 |
| Roman 2010 (n=60) | 60 | 0,000 | 0,000 to 5,963 | 0,64 | 0,64 |
| Schou 2018 (n=102) | 102 | 0,000 | 0,000 to 3,552 | 1,08 | 1,08 |
| Sugibayashi 2016 (n=40) | 40 | 0,000 | 0,000 to 8,810 | 0,43 | 0,43 |
| Takano 2015 (n=10) | 10 | 0,000 | 0,000 to 30,850 | 0,12 | 0,12 |
| Taylor 2002 (n=15) | 15 | 0,000 | 0,000 to 21,802 | 0,17 | 0,17 |
| Tsao 2002 (n=13) | 13 | 0,000 | 0,000 to 24,705 | 0,15 | 0,15 |
| Zhang 2018 (n=25) | 25 | 0,000 | 0,000 to 13,719 | 0,27 | 0,27 |
| Deprest 2005 (n=20) | 20 | 0,000 | 0,000 to 16,843 | 0,22 | 0,22 |
| Harrison 1998 (n=8) | 8 | 0,000 | 0,000 to 36,942 | 0,094 | 0,094 |
| Harrison 2003 (n=11) | 11 | 0,000 | 0,000 to 28,491 | 0,13 | 0,13 |
| Jani 2005 (n=24) | 24 | 0,000 | 0,000 to 14,247 | 0,26 | 0,26 |
| Jani 2006 (n=28) | 28 | 0,000 | 0,000 to 12,344 | 0,30 | 0,30 |
| Jani 2009 (n=210) | 210 | 0,000 | 0,000 to 1,741 | 2,22 | 2,22 |
| Jimenez 2017 (n=201) | 201 | 0,000 | 0,000 to 1,819 | 2,12 | 2,12 |
| Kosinski 2017 (n=28) | 28 | 0,000 | 0,000 to 12,344 | 0,30 | 0,30 |
| Manrique 2008 (n=11) | 11 | 0,000 | 0,000 to 28,491 | 0,13 | 0,13 |
| Peralta 2011 (n=8) | 8 | 0,000 | 0,000 to 36,942 | 0,094 | 0,094 |
| Persico 2017 (n=21) | 21 | 0,000 | 0,000 to 16,110 | 0,23 | 0,23 |
| Ruano 2012 (n=35) | 35 | 0,000 | 0,000 to 10,003 | 0,38 | 0,38 |
| Ruano 2012 (n=20) | 20 | 0,000 | 0,000 to 16,843 | 0,22 | 0,22 |
| Ruano 2013 (n=17) | 17 | 0,000 | 0,000 to 19,506 | 0,19 | 0,19 |
| Arens 2017 (n=59) | 59 | 0,000 | 0,000 to 6,061 | 0,63 | 0,63 |
| Belfort 2017 (n=22) | 22 | 0,000 | 0,000 to 15,437 | 0,24 | 0,24 |
| Bruner 2000 (n=4) | 4 | 0,000 | 0,000 to 60,236 | 0,052 | 0,052 |
| Degenhardt 2014 (n=51) | 51 | 0,000 | 0,000 to 6,978 | 0,55 | 0,55 |
| Kohn 2018 (n=34) | 34 | 0,000 | 0,000 to 10,282 | 0,37 | 0,37 |
| Pedreira 2014 (n=4) | 4 | 0,000 | 0,000 to 60,236 | 0,052 | 0,052 |
| Pedreira 2016 (n=10) | 10 | 0,000 | 0,000 to 30,850 | 0,12 | 0,12 |
| Verbeek 2012 (n=19) | 19 | 0,000 | 0,000 to 17,647 | 0,21 | 0,21 |
| Ziemann 2018 (n=65) | 65 | 0,000 | 0,000 to 5,517 | 0,69 | 0,69 |
| Morris 2013 (n=16) | 16 | 0,000 | 0,000 to 20,591 | 0,18 | 0,18 |
| Ruano 2010 (n=11) | 11 | 0,000 | 0,000 to 28,491 | 0,13 | 0,13 |
| Welsh 2003 (n=13) | 13 | 0,000 | 0,000 to 24,705 | 0,15 | 0,15 |
| Cavalheiro 2011 (n=30) | 30 | 0,000 | 0,000 to 11,570 | 0,33 | 0,33 |
| Mallman 2017 (n=78) | 78 | 0,000 | 0,000 to 4,619 | 0,83 | 0,83 |
| Golombeck 2006 (n=99) | 99 | 0,000 | 0,000 to 3,658 | 1,05 | 1,05 |
| Kohl 2006 (n=16) | 16 | 0,000 | 0,000 to 20,591 | 0,18 | 0,18 |
| Kohl 2010 (n=37) | 37 | 0,000 | 0,000 to 9,489 | 0,40 | 0,40 |
| Nivatpumin 2016 (n=152) | 152 | 0,000 | 0,000 to 2,398 | 1,61 | 1,61 |
| Peralta 2010 (n=56) | 56 | 0,000 | 0,000 to 6,375 | 0,60 | 0,60 |
| Total (fixed effects) | 9403 | 0,278 | 0,182 to 0,406 | 100,00 | 100,00 |
| Total (random effects) | 9403 | 0,278 | 0,182 to 0,394 | 100,00 | 100,00 |

## Test for heterogeneity

| Q | 57,1623 |
| --- | --- |
| DF | 121 |
| Significance level | P = 1,0000 |
| I^2^ (inconsistency) | 0,00% |
| 95% CI for I^2^ | 0,00 to 0,00 |

# Meta-analysis: intraoperative open bleeding

| Variable for studies | Study |
| --- | --- |
| Variable for total number of cases | N |
| Variable for number of positive cases | Outcome |

| Study | Sample size | Proportion (%) | 95% CI | Weight (%) | |
| --- | --- | --- | --- | --- | --- |
|  |  |  |  | Fixed | Random |
| Barthod 2013 (n=5) | 5 | 0,000 | 0,000 to 52,182 | 0,49 | 0,96 |
| Cass 2013 (n=9) | 9 | 0,000 | 0,000 to 33,627 | 0,81 | 1,48 |
| Chen 2018 (n=7) | 7 | 0,000 | 0,000 to 40,962 | 0,65 | 1,23 |
| Dahlgren 2004 (n=4) | 4 | 50,000 | 6,759 to 93,241 | 0,40 | 0,82 |
| Flake 2000 (n=15) | 15 | 0,000 | 0,000 to 21,802 | 1,29 | 2,12 |
| George 2007 (n=3) | 3 | 100,000 | 29,240 to 100,000 | 0,32 | 0,67 |
| Hedrick 2003 (n=43) | 43 | 4,651 | 0,568 to 15,811 | 3,56 | 3,89 |
| Hedrick 2005 (n=9) | 9 | 0,000 | 0,000 to 33,627 | 0,81 | 1,48 |
| Kern 2007 (n=5) | 5 | 20,000 | 0,505 to 71,642 | 0,49 | 0,96 |
| Kornacki 2017 (n=4) | 4 | 0,000 | 0,000 to 60,236 | 0,40 | 0,82 |
| Kunisaki 2007 (n=14) | 14 | 0,000 | 0,000 to 23,164 | 1,21 | 2,02 |
| Laje 2012 (n=17) | 17 | 11,765 | 1,458 to 36,441 | 1,46 | 2,30 |
| Laje 2013 (n=4) | 4 | 25,000 | 0,631 to 80,588 | 0,40 | 0,82 |
| Laje 2015 (n=13) | 13 | 0,000 | 0,000 to 24,705 | 1,13 | 1,92 |
| Lazar 2011 (n=12) | 12 | 0,000 | 0,000 to 26,465 | 1,05 | 1,82 |
| Noah 2002 (n=34) | 34 | 0,000 | 0,000 to 10,282 | 2,83 | 3,46 |
| Pellicer 2007 (n=3) | 3 | 0,000 | 0,000 to 70,760 | 0,32 | 0,67 |
| Stoffan 2012 (n=7) | 7 | 0,000 | 0,000 to 40,962 | 0,65 | 1,23 |
| Tuncay Ozgunen 2010 (n=3) | 3 | 0,000 | 0,000 to 70,760 | 0,32 | 0,67 |
| Zamora 2013 (n=26) | 26 | 0,000 | 0,000 to 13,227 | 2,18 | 2,99 |
| Bennett 2014 (n=43) | 43 | 0,000 | 0,000 to 8,221 | 3,56 | 3,89 |
| Botelho 2017 (n=45) | 45 | 0,000 | 0,000 to 7,871 | 3,72 | 3,97 |
| Bruner 1999 (n=29) | 29 | 0,000 | 0,000 to 11,944 | 2,43 | 3,18 |
| Bruner 2000 (n=4) | 4 | 0,000 | 0,000 to 60,236 | 0,40 | 0,82 |
| Farmer 2003 (n=12) | 12 | 8,333 | 0,211 to 38,480 | 1,05 | 1,82 |
| Friszer 2016 (n=3) | 3 | 0,000 | 0,000 to 70,760 | 0,32 | 0,67 |
| Johnson 2016 (n=91) | 91 | 0,000 | 0,000 to 3,973 | 7,44 | 5,18 |
| Marenco 2013 (n=4) | 4 | 0,000 | 0,000 to 60,236 | 0,40 | 0,82 |
| Moldenhauer 2015 (n=100) | 100 | 0,000 | 0,000 to 3,622 | 8,17 | 5,33 |
| Moron 2018 (n=237) | 237 | 0,000 | 0,000 to 1,544 | 19,26 | 6,38 |
| Ochsenbein-Kolble 2017 (n=30) | 30 | 0,000 | 0,000 to 11,570 | 2,51 | 3,24 |
| Sinskey 2017 (n=47) | 47 | 0,000 | 0,000 to 7,549 | 3,88 | 4,05 |
| Soni 2016 (n=88) | 88 | 0,000 | 0,000 to 4,105 | 7,20 | 5,13 |
| Zamlynski 2014 (n=46) | 46 | 0,000 | 0,000 to 7,706 | 3,80 | 4,01 |
| Flake 2000 (n=15) | 15 | 0,000 | 0,000 to 21,802 | 1,29 | 2,12 |
| Harrison 1990 (n=6) | 6 | 0,000 | 0,000 to 45,926 | 0,57 | 1,10 |
| Harrison 1993 (n=14) | 14 | 0,000 | 0,000 to 23,164 | 1,21 | 2,02 |
| Harrison 1998 (n=13) | 13 | 0,000 | 0,000 to 24,705 | 1,13 | 1,92 |
| Adzick 2003 (n=22) | 22 | 0,000 | 0,000 to 15,437 | 1,86 | 2,71 |
| Hedrick 2004 (n=4) | 4 | 25,000 | 0,631 to 80,588 | 0,40 | 0,82 |
| Golombeck 2006 (n=79) | 79 | 0,000 | 0,000 to 4,562 | 6,47 | 4,96 |
| Longaker 1991 (n=17) | 17 | 0,000 | 0,000 to 19,506 | 1,46 | 2,30 |
| Zamora 2013 (n=7) | 7 | 0,000 | 0,000 to 40,962 | 0,65 | 1,23 |
| Total (fixed effects) | 1193 | 1,041 | 0,553 to 1,778 | 100,00 | 100,00 |
| Total (random effects) | 1193 | 1,971 | 0,972 to 3,310 | 100,00 | 100,00 |

## Test for heterogeneity

| Q | 70,4505 |
| --- | --- |
| DF | 42 |
| Significance level | P = 0,0039 |
| I^2^ (inconsistency) | 40,38% |
| 95% CI for I^2^ | 13,92 to 58,71 |

# Meta-analysis: intraoperative fetoscopic bleeding

| Variable for studies | Study |
| --- | --- |
| Variable for total number of cases | N |
| Variable for number of positive cases | Outcome |

| Study | Sample size | Proportion (%) | 95% CI | Weight (%) | |
| --- | --- | --- | --- | --- | --- |
|  |  |  |  | Fixed | Random |
| Aboudiab 2017 (n=18) | 18 | 0,000 | 0,000 to 18,530 | 0,20 | 0,54 |
| Baschat 2013 (n=147) | 147 | 2,041 | 0,423 to 5,848 | 1,55 | 1,25 |
| Chalouhi 2016 (n=22) | 22 | 0,000 | 0,000 to 15,437 | 0,24 | 0,61 |
| Chang 2006 (n=27) | 27 | 3,704 | 0,0937 to 18,971 | 0,29 | 0,68 |
| Chang 2016 (n=100) | 100 | 3,000 | 0,623 to 8,518 | 1,06 | 1,15 |
| Chmait 2013 (n=318) | 318 | 0,943 | 0,195 to 2,732 | 3,35 | 1,39 |
| Chmait 2017 (n=19) | 19 | 0,000 | 0,000 to 17,647 | 0,21 | 0,56 |
| Crombleholme 2007 (n=20) | 20 | 5,000 | 0,127 to 24,873 | 0,22 | 0,58 |
| De Lia 1995 (n=26) | 26 | 23,077 | 8,974 to 43,648 | 0,28 | 0,67 |
| De Lia 1999 (n=67) | 67 | 0,000 | 0,000 to 5,357 | 0,71 | 1,02 |
| De Lia 2009 (n=10) | 10 | 10,000 | 0,253 to 44,502 | 0,12 | 0,37 |
| Deprest 1998 (n=6) | 6 | 0,000 | 0,000 to 45,926 | 0,073 | 0,25 |
| Draga 2016 (n=37) | 37 | 0,000 | 0,000 to 9,489 | 0,40 | 0,80 |
| Duron 2014 (n=85) | 85 | 3,529 | 0,734 to 9,970 | 0,90 | 1,10 |
| Ek 2012 (n=) | 67 | 0,000 | 0,000 to 5,357 | 0,71 | 1,02 |
| Habli 2009 (n=152) | 152 | 1,974 | 0,409 to 5,659 | 1,61 | 1,26 |
| Has 2014 (n=85) | 85 | 2,353 | 0,286 to 8,242 | 0,90 | 1,10 |
| Hecher 2000 (n=200) | 200 | 0,000 | 0,000 to 1,828 | 2,11 | 1,32 |
| Hernandez-Andrade 2011 (n=35) | 35 | 34,286 | 19,132 to 52,211 | 0,38 | 0,78 |
| Huber 2008 (n=176) | 176 | 0,568 | 0,0144 to 3,125 | 1,86 | 1,29 |
| Ishii 2014 (n=16) | 16 | 0,000 | 0,000 to 20,591 | 0,18 | 0,50 |
| Ishii 2015 (n=10) | 10 | 0,000 | 0,000 to 30,850 | 0,12 | 0,37 |
| Lanna 2017 (n=373) | 373 | 0,000 | 0,000 to 0,984 | 3,93 | 1,41 |
| Lecointre 2017 (n=200) | 200 | 0,000 | 0,000 to 1,828 | 2,11 | 1,32 |
| Malshe 2017 (n=203) | 203 | 0,000 | 0,000 to 1,801 | 2,14 | 1,32 |
| Martinez 2012 (n=500) | 500 | 0,000 | 0,000 to 0,735 | 5,26 | 1,45 |
| Middeldorp 2007 (n=100) | 100 | 0,000 | 0,000 to 3,622 | 1,06 | 1,15 |
| Miyadahira 2018 (n=67) | 67 | 0,000 | 0,000 to 5,357 | 0,71 | 1,02 |
| Molina-Garcia 2009 (n=22) | 22 | 0,000 | 0,000 to 15,437 | 0,24 | 0,61 |
| Morris 2010 (n=164) | 164 | 0,000 | 0,000 to 2,224 | 1,73 | 1,27 |
| Mullers 2015 (n=105) | 105 | 2,857 | 0,593 to 8,123 | 1,11 | 1,16 |
| Nakata 2016 (n=6) | 6 | 0,000 | 0,000 to 45,926 | 0,073 | 0,25 |
| Nguyen 2012 (n=98) | 98 | 0,000 | 0,000 to 3,694 | 1,04 | 1,14 |
| Ozawa 2017 (n=11) | 11 | 0,000 | 0,000 to 28,491 | 0,13 | 0,39 |
| Papanna 2010 (n=48) | 48 | 4,167 | 0,509 to 14,254 | 0,51 | 0,90 |
| Papanna 2012 (n=163) | 163 | 0,000 | 0,000 to 2,238 | 1,72 | 1,27 |
| Peeters 2014 (n=338) | 338 | 7,692 | 5,086 to 11,068 | 3,56 | 1,40 |
| Persico 2016 (n=106) | 106 | 0,943 | 0,0239 to 5,144 | 1,12 | 1,16 |
| Quintero 2000 (n=92) | 92 | 1,087 | 0,0275 to 5,908 | 0,98 | 1,12 |
| Quintero 2001 (n=11) | 11 | 0,000 | 0,000 to 28,491 | 0,13 | 0,39 |
| Rossi 2008 (n=266) | 266 | 9,023 | 5,867 to 13,127 | 2,80 | 1,37 |
| Ruano 2009 (n=19) | 19 | 0,000 | 0,000 to 17,647 | 0,21 | 0,56 |
| Ruegg 2018 (n=37) | 37 | 5,405 | 0,661 to 18,195 | 0,40 | 0,80 |
| Rustico 2012 (n=150) | 150 | 0,667 | 0,0169 to 3,658 | 1,59 | 1,25 |
| Said 2008 (n=10) | 10 | 0,000 | 0,000 to 30,850 | 0,12 | 0,37 |
| Senat 2004 (n=72) | 72 | 0,000 | 0,000 to 4,994 | 0,77 | 1,04 |
| Sepulveda 2007 (n=33) | 33 | 9,091 | 1,915 to 24,332 | 0,36 | 0,76 |
| Shamshirsaz 2015 (n=55) | 55 | 7,273 | 2,017 to 17,587 | 0,59 | 0,95 |
| Slaghekke 2014 (n=274) | 274 | 4,015 | 2,021 to 7,070 | 2,89 | 1,37 |
| Taniguchi 2015 (n=3) | 3 | 0,000 | 0,000 to 70,760 | 0,042 | 0,16 |
| Tchirikov 2011 (n=80) | 80 | 0,000 | 0,000 to 4,506 | 0,85 | 1,08 |
| Teoh 2013 (n=49) | 49 | 0,000 | 0,000 to 7,252 | 0,52 | 0,91 |
| Thia 2017 (n=5) | 5 | 20,000 | 0,505 to 71,642 | 0,063 | 0,22 |
| Ville 1997 (n=132) | 132 | 3,030 | 0,832 to 7,577 | 1,40 | 1,22 |
| Ville 1998 (n=44) | 44 | 4,545 | 0,555 to 15,473 | 0,47 | 0,87 |
| Weingertner 2011 (n=100) | 100 | 0,000 | 0,000 to 3,622 | 1,06 | 1,15 |
| Wilson 2016 (n=151) | 151 | 0,662 | 0,0168 to 3,634 | 1,60 | 1,26 |
| Yamamoto 2005 (n=175) | 175 | 8,000 | 4,443 to 13,058 | 1,85 | 1,29 |
| Yang 2010 (n=30) | 30 | 10,000 | 2,112 to 26,529 | 0,33 | 0,72 |
| Zaretsky 2018 (n=749) | 749 | 0,000 | 0,000 to 0,491 | 7,87 | 1,48 |
| Zhao 2016 (n=62) | 62 | 6,452 | 1,786 to 15,703 | 0,66 | 0,99 |
| Bebbington 2012 (n=146) | 146 | 0,000 | 0,000 to 2,495 | 1,54 | 1,25 |
| Berg 2014 (n=7) | 7 | 0,000 | 0,000 to 40,962 | 0,084 | 0,28 |
| Delabaere 2013 (n=30) | 30 | 3,333 | 0,0844 to 17,217 | 0,33 | 0,72 |
| Deprest 2000 (n=10) | 10 | 0,000 | 0,000 to 30,850 | 0,12 | 0,37 |
| Gallot 2003 (n=11) | 11 | 9,091 | 0,230 to 41,278 | 0,13 | 0,39 |
| Gouverneur 2009 (n=54) | 54 | 0,000 | 0,000 to 6,603 | 0,58 | 0,94 |
| Gul 2008 (n=9) | 9 | 0,000 | 0,000 to 33,627 | 0,10 | 0,34 |
| Has 2014 (n=71) | 71 | 0,000 | 0,000 to 5,063 | 0,76 | 1,04 |
| He 2010 (n=14) | 14 | 0,000 | 0,000 to 23,164 | 0,16 | 0,46 |
| Ilagan 2008 (n=27) | 27 | 0,000 | 0,000 to 12,770 | 0,29 | 0,68 |
| Jelin 2010 (n=7) | 7 | 0,000 | 0,000 to 40,962 | 0,084 | 0,28 |
| King 2017 (n=43) | 43 | 0,000 | 0,000 to 8,221 | 0,46 | 0,86 |
| Lanna 2012 (n=118) | 118 | 0,000 | 0,000 to 3,078 | 1,25 | 1,19 |
| Lee 2013 (n=98) | 98 | 0,000 | 0,000 to 3,694 | 1,04 | 1,14 |
| Lewi 2006 (n=80) | 80 | 0,000 | 0,000 to 4,506 | 0,85 | 1,08 |
| Moise 2008 (n=9) | 9 | 0,000 | 0,000 to 33,627 | 0,10 | 0,34 |
| Nobili 2013 (n=48) | 48 | 0,000 | 0,000 to 7,397 | 0,51 | 0,90 |
| Paramasivam 2010 (n=35) | 35 | 0,000 | 0,000 to 10,003 | 0,38 | 0,78 |
| Peng 2016 (n=93) | 93 | 0,000 | 0,000 to 3,889 | 0,99 | 1,12 |
| Quintero 1996 (n=13) | 13 | 30,769 | 9,092 to 61,426 | 0,15 | 0,44 |
| Quintero 2006 (n=51) | 51 | 5,882 | 1,230 to 16,242 | 0,55 | 0,92 |
| Roman 2010 (n=60) | 60 | 0,000 | 0,000 to 5,963 | 0,64 | 0,98 |
| Schou 2018 (n=102) | 102 | 0,000 | 0,000 to 3,552 | 1,08 | 1,15 |
| Sugibayashi 2016 (n=40) | 40 | 0,000 | 0,000 to 8,810 | 0,43 | 0,83 |
| Takano 2015 (n=10) | 10 | 0,000 | 0,000 to 30,850 | 0,12 | 0,37 |
| Taylor 2002 (n=15) | 15 | 6,667 | 0,169 to 31,948 | 0,17 | 0,48 |
| Tsao 2002 (n=13) | 13 | 0,000 | 0,000 to 24,705 | 0,15 | 0,44 |
| Zhang 2018 (n=25) | 25 | 0,000 | 0,000 to 13,719 | 0,27 | 0,65 |
| Deprest 2005 (n=20) | 20 | 0,000 | 0,000 to 16,843 | 0,22 | 0,58 |
| Harrison 1998 (n=8) | 8 | 0,000 | 0,000 to 36,942 | 0,094 | 0,31 |
| Harrison 2003 (n=11) | 11 | 0,000 | 0,000 to 28,491 | 0,13 | 0,39 |
| Jani 2005 (n=24) | 24 | 0,000 | 0,000 to 14,247 | 0,26 | 0,64 |
| Jani 2006 (n=28) | 28 | 0,000 | 0,000 to 12,344 | 0,30 | 0,70 |
| Jani 2009 (n=210) | 210 | 0,476 | 0,0121 to 2,624 | 2,22 | 1,33 |
| Jimenez 2017 (n=201) | 201 | 0,000 | 0,000 to 1,819 | 2,12 | 1,32 |
| Kosinski 2017 (n=28) | 28 | 0,000 | 0,000 to 12,344 | 0,30 | 0,70 |
| Manrique 2008 (n=11) | 11 | 0,000 | 0,000 to 28,491 | 0,13 | 0,39 |
| Peralta 2011 (n=8) | 8 | 0,000 | 0,000 to 36,942 | 0,094 | 0,31 |
| Persico 2017 (n=21) | 21 | 0,000 | 0,000 to 16,110 | 0,23 | 0,59 |
| Ruano 2012 (n=35) | 35 | 0,000 | 0,000 to 10,003 | 0,38 | 0,78 |
| Ruano 2012 (n=20) | 20 | 0,000 | 0,000 to 16,843 | 0,22 | 0,58 |
| Ruano 2013 (n=17) | 17 | 0,000 | 0,000 to 19,506 | 0,19 | 0,52 |
| Arens 2017 (n=59) | 59 | 0,000 | 0,000 to 6,061 | 0,63 | 0,97 |
| Belfort 2017 (n=22) | 22 | 0,000 | 0,000 to 15,437 | 0,24 | 0,61 |
| Bruner 2000 (n=4) | 4 | 0,000 | 0,000 to 60,236 | 0,052 | 0,19 |
| Degenhardt 2014 (n=51) | 51 | 0,000 | 0,000 to 6,978 | 0,55 | 0,92 |
| Kohn 2018 (n=34) | 34 | 0,000 | 0,000 to 10,282 | 0,37 | 0,77 |
| Pedreira 2014 (n=4) | 4 | 0,000 | 0,000 to 60,236 | 0,052 | 0,19 |
| Pedreira 2016 (n=10) | 10 | 0,000 | 0,000 to 30,850 | 0,12 | 0,37 |
| Verbeek 2012 (n=19) | 19 | 15,789 | 3,383 to 39,578 | 0,21 | 0,56 |
| Ziemann 2018 (n=65) | 65 | 0,000 | 0,000 to 5,517 | 0,69 | 1,01 |
| Morris 2013 (n=16) | 16 | 0,000 | 0,000 to 20,591 | 0,18 | 0,50 |
| Ruano 2010 (n=11) | 11 | 0,000 | 0,000 to 28,491 | 0,13 | 0,39 |
| Welsh 2003 (n=13) | 13 | 0,000 | 0,000 to 24,705 | 0,15 | 0,44 |
| Cavalheiro 2011 (n=30) | 30 | 0,000 | 0,000 to 11,570 | 0,33 | 0,72 |
| Mallman 2017 (n=78) | 78 | 0,000 | 0,000 to 4,619 | 0,83 | 1,07 |
| Golombeck 2006 (n=99) | 99 | 0,000 | 0,000 to 3,658 | 1,05 | 1,14 |
| Kohl 2006 (n=16) | 16 | 0,000 | 0,000 to 20,591 | 0,18 | 0,50 |
| Kohl 2010 (n=37) | 37 | 10,811 | 3,025 to 25,418 | 0,40 | 0,80 |
| Nivatpumin 2016 (n=152) | 152 | 0,000 | 0,000 to 2,398 | 1,61 | 1,26 |
| Peralta 2010 (n=56) | 56 | 1,786 | 0,0452 to 9,553 | 0,60 | 0,95 |
| Total (fixed effects) | 9403 | 1,211 | 1,001 to 1,452 | 100,00 | 100,00 |
| Total (random effects) | 9403 | 1,743 | 1,248 to 2,319 | 100,00 | 100,00 |

## Test for heterogeneity

| Q | 383,1181 |
| --- | --- |
| DF | 121 |
| Significance level | P < 0,0001 |
| I^2^ (inconsistency) | 68,42% |
| 95% CI for I^2^ | 61,97 to 73,77 |

# Meta-analysis: intraoperative open transfusion

| Variable for studies | Study |
| --- | --- |
| Variable for total number of cases | N |
| Variable for number of positive cases | Outcome |

| Study | Sample size | Proportion (%) | 95% CI | Weight (%) | |
| --- | --- | --- | --- | --- | --- |
|  |  |  |  | Fixed | Random |
| Barthod 2013 (n=5) | 5 | 0,000 | 0,000 to 52,182 | 0,49 | 0,49 |
| Cass 2013 (n=9) | 9 | 0,000 | 0,000 to 33,627 | 0,81 | 0,81 |
| Chen 2018 (n=7) | 7 | 0,000 | 0,000 to 40,962 | 0,65 | 0,65 |
| Dahlgren 2004 (n=4) | 4 | 25,000 | 0,631 to 80,588 | 0,40 | 0,40 |
| Flake 2000 (n=15) | 15 | 0,000 | 0,000 to 21,802 | 1,29 | 1,29 |
| George 2007 (n=3) | 3 | 0,000 | 0,000 to 70,760 | 0,32 | 0,32 |
| Hedrick 2003 (n=43) | 43 | 4,651 | 0,568 to 15,811 | 3,56 | 3,56 |
| Hedrick 2005 (n=9) | 9 | 0,000 | 0,000 to 33,627 | 0,81 | 0,81 |
| Kern 2007 (n=5) | 5 | 0,000 | 0,000 to 52,182 | 0,49 | 0,49 |
| Kornacki 2017 (n=4) | 4 | 0,000 | 0,000 to 60,236 | 0,40 | 0,40 |
| Kunisaki 2007 (n=14) | 14 | 0,000 | 0,000 to 23,164 | 1,21 | 1,21 |
| Laje 2012 (n=17) | 17 | 11,765 | 1,458 to 36,441 | 1,46 | 1,46 |
| Laje 2013 (n=4) | 4 | 25,000 | 0,631 to 80,588 | 0,40 | 0,40 |
| Laje 2015 (n=13) | 13 | 0,000 | 0,000 to 24,705 | 1,13 | 1,13 |
| Lazar 2011 (n=12) | 12 | 0,000 | 0,000 to 26,465 | 1,05 | 1,05 |
| Noah 2002 (n=34) | 34 | 0,000 | 0,000 to 10,282 | 2,83 | 2,83 |
| Pellicer 2007 (n=3) | 3 | 0,000 | 0,000 to 70,760 | 0,32 | 0,32 |
| Stoffan 2012 (n=7) | 7 | 0,000 | 0,000 to 40,962 | 0,65 | 0,65 |
| Tuncay Ozgunen 2010 (n=3) | 3 | 0,000 | 0,000 to 70,760 | 0,32 | 0,32 |
| Zamora 2013 (n=26) | 26 | 0,000 | 0,000 to 13,227 | 2,18 | 2,18 |
| Bennett 2014 (n=43) | 43 | 0,000 | 0,000 to 8,221 | 3,56 | 3,56 |
| Botelho 2017 (n=45) | 45 | 0,000 | 0,000 to 7,871 | 3,72 | 3,72 |
| Bruner 1999 (n=29) | 29 | 0,000 | 0,000 to 11,944 | 2,43 | 2,43 |
| Bruner 2000 (n=4) | 4 | 0,000 | 0,000 to 60,236 | 0,40 | 0,40 |
| Farmer 2003 (n=12) | 12 | 0,000 | 0,000 to 26,465 | 1,05 | 1,05 |
| Friszer 2016 (n=3) | 3 | 0,000 | 0,000 to 70,760 | 0,32 | 0,32 |
| Johnson 2016 (n=91) | 91 | 0,000 | 0,000 to 3,973 | 7,44 | 7,44 |
| Marenco 2013 (n=4) | 4 | 0,000 | 0,000 to 60,236 | 0,40 | 0,40 |
| Moldenhauer 2015 (n=100) | 100 | 0,000 | 0,000 to 3,622 | 8,17 | 8,17 |
| Moron 2018 (n=237) | 237 | 0,000 | 0,000 to 1,544 | 19,26 | 19,26 |
| Ochsenbein-Kolble 2017 (n=30) | 30 | 0,000 | 0,000 to 11,570 | 2,51 | 2,51 |
| Sinskey 2017 (n=47) | 47 | 4,255 | 0,520 to 14,541 | 3,88 | 3,88 |
| Soni 2016 (n=88) | 88 | 0,000 | 0,000 to 4,105 | 7,20 | 7,20 |
| Zamlynski 2014 (n=46) | 46 | 0,000 | 0,000 to 7,706 | 3,80 | 3,80 |
| Flake 2000 (n=15) | 15 | 0,000 | 0,000 to 21,802 | 1,29 | 1,29 |
| Harrison 1990 (n=6) | 6 | 0,000 | 0,000 to 45,926 | 0,57 | 0,57 |
| Harrison 1993 (n=14) | 14 | 0,000 | 0,000 to 23,164 | 1,21 | 1,21 |
| Harrison 1998 (n=13) | 13 | 0,000 | 0,000 to 24,705 | 1,13 | 1,13 |
| Adzick 2003 (n=22) | 22 | 0,000 | 0,000 to 15,437 | 1,86 | 1,86 |
| Hedrick 2004 (n=4) | 4 | 25,000 | 0,631 to 80,588 | 0,40 | 0,40 |
| Golombeck 2006 (n=79) | 79 | 0,000 | 0,000 to 4,562 | 6,47 | 6,47 |
| Longaker 1991 (n=17) | 17 | 0,000 | 0,000 to 19,506 | 1,46 | 1,46 |
| Zamora 2013 (n=7) | 7 | 0,000 | 0,000 to 40,962 | 0,65 | 0,65 |
| Total (fixed effects) | 1193 | 1,005 | 0,527 to 1,733 | 100,00 | 100,00 |
| Total (random effects) | 1193 | 1,005 | 0,525 to 1,637 | 100,00 | 100,00 |

## Test for heterogeneity

| Q | 38,7995 |
| --- | --- |
| DF | 42 |
| Significance level | P = 0,6123 |
| I^2^ (inconsistency) | 0,00% |
| 95% CI for I^2^ | 0,00 to 29,79 |

# Meta-analysis: intraoperative fetoscopic transfusion

| Variable for studies | Study |
| --- | --- |
| Variable for total number of cases | N |
| Variable for number of positive cases | Outcome |

| Study | Sample size | Proportion (%) | 95% CI | Weight (%) | |
| --- | --- | --- | --- | --- | --- |
|  |  |  |  | Fixed | Random |
| Aboudiab 2017 (n=18) | 18 | 0,000 | 0,000 to 18,530 | 0,20 | 0,20 |
| Baschat 2013 (n=147) | 147 | 0,000 | 0,000 to 2,478 | 1,55 | 1,55 |
| Chalouhi 2016 (n=22) | 22 | 0,000 | 0,000 to 15,437 | 0,24 | 0,24 |
| Chang 2006 (n=27) | 27 | 0,000 | 0,000 to 12,770 | 0,29 | 0,29 |
| Chang 2016 (n=100) | 100 | 0,000 | 0,000 to 3,622 | 1,06 | 1,06 |
| Chmait 2013 (n=318) | 318 | 0,000 | 0,000 to 1,153 | 3,35 | 3,35 |
| Chmait 2017 (n=19) | 19 | 0,000 | 0,000 to 17,647 | 0,21 | 0,21 |
| Crombleholme 2007 (n=20) | 20 | 0,000 | 0,000 to 16,843 | 0,22 | 0,22 |
| De Lia 1995 (n=26) | 26 | 0,000 | 0,000 to 13,227 | 0,28 | 0,28 |
| De Lia 1999 (n=67) | 67 | 0,000 | 0,000 to 5,357 | 0,71 | 0,71 |
| De Lia 2009 (n=10) | 10 | 0,000 | 0,000 to 30,850 | 0,12 | 0,12 |
| Deprest 1998 (n=6) | 6 | 0,000 | 0,000 to 45,926 | 0,073 | 0,073 |
| Draga 2016 (n=37) | 37 | 0,000 | 0,000 to 9,489 | 0,40 | 0,40 |
| Duron 2014 (n=85) | 85 | 0,000 | 0,000 to 4,247 | 0,90 | 0,90 |
| Ek 2012 (n=) | 67 | 0,000 | 0,000 to 5,357 | 0,71 | 0,71 |
| Habli 2009 (n=152) | 152 | 0,000 | 0,000 to 2,398 | 1,61 | 1,61 |
| Has 2014 (n=85) | 85 | 0,000 | 0,000 to 4,247 | 0,90 | 0,90 |
| Hecher 2000 (n=200) | 200 | 0,000 | 0,000 to 1,828 | 2,11 | 2,11 |
| Hernandez-Andrade 2011 (n=35) | 35 | 0,000 | 0,000 to 10,003 | 0,38 | 0,38 |
| Huber 2008 (n=176) | 176 | 0,000 | 0,000 to 2,074 | 1,86 | 1,86 |
| Ishii 2014 (n=16) | 16 | 0,000 | 0,000 to 20,591 | 0,18 | 0,18 |
| Ishii 2015 (n=10) | 10 | 0,000 | 0,000 to 30,850 | 0,12 | 0,12 |
| Lanna 2017 (n=373) | 373 | 0,000 | 0,000 to 0,984 | 3,93 | 3,93 |
| Lecointre 2017 (n=200) | 200 | 0,000 | 0,000 to 1,828 | 2,11 | 2,11 |
| Malshe 2017 (n=203) | 203 | 0,000 | 0,000 to 1,801 | 2,14 | 2,14 |
| Martinez 2012 (n=500) | 500 | 0,000 | 0,000 to 0,735 | 5,26 | 5,26 |
| Middeldorp 2007 (n=100) | 100 | 0,000 | 0,000 to 3,622 | 1,06 | 1,06 |
| Miyadahira 2018 (n=67) | 67 | 0,000 | 0,000 to 5,357 | 0,71 | 0,71 |
| Molina-Garcia 2009 (n=22) | 22 | 0,000 | 0,000 to 15,437 | 0,24 | 0,24 |
| Morris 2010 (n=164) | 164 | 0,000 | 0,000 to 2,224 | 1,73 | 1,73 |
| Mullers 2015 (n=105) | 105 | 0,000 | 0,000 to 3,452 | 1,11 | 1,11 |
| Nakata 2016 (n=6) | 6 | 0,000 | 0,000 to 45,926 | 0,073 | 0,073 |
| Nguyen 2012 (n=98) | 98 | 0,000 | 0,000 to 3,694 | 1,04 | 1,04 |
| Ozawa 2017 (n=11) | 11 | 0,000 | 0,000 to 28,491 | 0,13 | 0,13 |
| Papanna 2010 (n=48) | 48 | 0,000 | 0,000 to 7,397 | 0,51 | 0,51 |
| Papanna 2012 (n=163) | 163 | 0,000 | 0,000 to 2,238 | 1,72 | 1,72 |
| Peeters 2014 (n=338) | 338 | 0,000 | 0,000 to 1,085 | 3,56 | 3,56 |
| Persico 2016 (n=106) | 106 | 0,943 | 0,0239 to 5,144 | 1,12 | 1,12 |
| Quintero 2000 (n=92) | 92 | 1,087 | 0,0275 to 5,908 | 0,98 | 0,98 |
| Quintero 2001 (n=11) | 11 | 0,000 | 0,000 to 28,491 | 0,13 | 0,13 |
| Rossi 2008 (n=266) | 266 | 0,000 | 0,000 to 1,377 | 2,80 | 2,80 |
| Ruano 2009 (n=19) | 19 | 0,000 | 0,000 to 17,647 | 0,21 | 0,21 |
| Ruegg 2018 (n=37) | 37 | 0,000 | 0,000 to 9,489 | 0,40 | 0,40 |
| Rustico 2012 (n=150) | 150 | 0,000 | 0,000 to 2,429 | 1,59 | 1,59 |
| Said 2008 (n=10) | 10 | 0,000 | 0,000 to 30,850 | 0,12 | 0,12 |
| Senat 2004 (n=72) | 72 | 0,000 | 0,000 to 4,994 | 0,77 | 0,77 |
| Sepulveda 2007 (n=33) | 33 | 0,000 | 0,000 to 10,576 | 0,36 | 0,36 |
| Shamshirsaz 2015 (n=55) | 55 | 0,000 | 0,000 to 6,487 | 0,59 | 0,59 |
| Slaghekke 2014 (n=274) | 274 | 0,000 | 0,000 to 1,337 | 2,89 | 2,89 |
| Taniguchi 2015 (n=3) | 3 | 0,000 | 0,000 to 70,760 | 0,042 | 0,042 |
| Tchirikov 2011 (n=80) | 80 | 0,000 | 0,000 to 4,506 | 0,85 | 0,85 |
| Teoh 2013 (n=49) | 49 | 0,000 | 0,000 to 7,252 | 0,52 | 0,52 |
| Thia 2017 (n=5) | 5 | 0,000 | 0,000 to 52,182 | 0,063 | 0,063 |
| Ville 1997 (n=132) | 132 | 0,000 | 0,000 to 2,756 | 1,40 | 1,40 |
| Ville 1998 (n=44) | 44 | 0,000 | 0,000 to 8,042 | 0,47 | 0,47 |
| Weingertner 2011 (n=100) | 100 | 0,000 | 0,000 to 3,622 | 1,06 | 1,06 |
| Wilson 2016 (n=151) | 151 | 0,000 | 0,000 to 2,413 | 1,60 | 1,60 |
| Yamamoto 2005 (n=175) | 175 | 0,000 | 0,000 to 2,086 | 1,85 | 1,85 |
| Yang 2010 (n=30) | 30 | 0,000 | 0,000 to 11,570 | 0,33 | 0,33 |
| Zaretsky 2018 (n=749) | 749 | 0,000 | 0,000 to 0,491 | 7,87 | 7,87 |
| Zhao 2016 (n=62) | 62 | 0,000 | 0,000 to 5,776 | 0,66 | 0,66 |
| Bebbington 2012 (n=146) | 146 | 0,000 | 0,000 to 2,495 | 1,54 | 1,54 |
| Berg 2014 (n=7) | 7 | 0,000 | 0,000 to 40,962 | 0,084 | 0,084 |
| Delabaere 2013 (n=30) | 30 | 0,000 | 0,000 to 11,570 | 0,33 | 0,33 |
| Deprest 2000 (n=10) | 10 | 0,000 | 0,000 to 30,850 | 0,12 | 0,12 |
| Gallot 2003 (n=11) | 11 | 0,000 | 0,000 to 28,491 | 0,13 | 0,13 |
| Gouverneur 2009 (n=54) | 54 | 0,000 | 0,000 to 6,603 | 0,58 | 0,58 |
| Gul 2008 (n=9) | 9 | 0,000 | 0,000 to 33,627 | 0,10 | 0,10 |
| Has 2014 (n=71) | 71 | 0,000 | 0,000 to 5,063 | 0,76 | 0,76 |
| He 2010 (n=14) | 14 | 0,000 | 0,000 to 23,164 | 0,16 | 0,16 |
| Ilagan 2008 (n=27) | 27 | 0,000 | 0,000 to 12,770 | 0,29 | 0,29 |
| Jelin 2010 (n=7) | 7 | 0,000 | 0,000 to 40,962 | 0,084 | 0,084 |
| King 2017 (n=43) | 43 | 0,000 | 0,000 to 8,221 | 0,46 | 0,46 |
| Lanna 2012 (n=118) | 118 | 0,000 | 0,000 to 3,078 | 1,25 | 1,25 |
| Lee 2013 (n=98) | 98 | 0,000 | 0,000 to 3,694 | 1,04 | 1,04 |
| Lewi 2006 (n=80) | 80 | 0,000 | 0,000 to 4,506 | 0,85 | 0,85 |
| Moise 2008 (n=9) | 9 | 0,000 | 0,000 to 33,627 | 0,10 | 0,10 |
| Nobili 2013 (n=48) | 48 | 0,000 | 0,000 to 7,397 | 0,51 | 0,51 |
| Paramasivam 2010 (n=35) | 35 | 0,000 | 0,000 to 10,003 | 0,38 | 0,38 |
| Peng 2016 (n=93) | 93 | 0,000 | 0,000 to 3,889 | 0,99 | 0,99 |
| Quintero 1996 (n=13) | 13 | 0,000 | 0,000 to 24,705 | 0,15 | 0,15 |
| Quintero 2006 (n=51) | 51 | 0,000 | 0,000 to 6,978 | 0,55 | 0,55 |
| Roman 2010 (n=60) | 60 | 0,000 | 0,000 to 5,963 | 0,64 | 0,64 |
| Schou 2018 (n=102) | 102 | 0,000 | 0,000 to 3,552 | 1,08 | 1,08 |
| Sugibayashi 2016 (n=40) | 40 | 0,000 | 0,000 to 8,810 | 0,43 | 0,43 |
| Takano 2015 (n=10) | 10 | 0,000 | 0,000 to 30,850 | 0,12 | 0,12 |
| Taylor 2002 (n=15) | 15 | 0,000 | 0,000 to 21,802 | 0,17 | 0,17 |
| Tsao 2002 (n=13) | 13 | 0,000 | 0,000 to 24,705 | 0,15 | 0,15 |
| Zhang 2018 (n=25) | 25 | 0,000 | 0,000 to 13,719 | 0,27 | 0,27 |
| Deprest 2005 (n=20) | 20 | 0,000 | 0,000 to 16,843 | 0,22 | 0,22 |
| Harrison 1998 (n=8) | 8 | 0,000 | 0,000 to 36,942 | 0,094 | 0,094 |
| Harrison 2003 (n=11) | 11 | 0,000 | 0,000 to 28,491 | 0,13 | 0,13 |
| Jani 2005 (n=24) | 24 | 0,000 | 0,000 to 14,247 | 0,26 | 0,26 |
| Jani 2006 (n=28) | 28 | 0,000 | 0,000 to 12,344 | 0,30 | 0,30 |
| Jani 2009 (n=210) | 210 | 0,476 | 0,0121 to 2,624 | 2,22 | 2,22 |
| Jimenez 2017 (n=201) | 201 | 0,000 | 0,000 to 1,819 | 2,12 | 2,12 |
| Kosinski 2017 (n=28) | 28 | 0,000 | 0,000 to 12,344 | 0,30 | 0,30 |
| Manrique 2008 (n=11) | 11 | 0,000 | 0,000 to 28,491 | 0,13 | 0,13 |
| Peralta 2011 (n=8) | 8 | 0,000 | 0,000 to 36,942 | 0,094 | 0,094 |
| Persico 2017 (n=21) | 21 | 0,000 | 0,000 to 16,110 | 0,23 | 0,23 |
| Ruano 2012 (n=35) | 35 | 0,000 | 0,000 to 10,003 | 0,38 | 0,38 |
| Ruano 2012 (n=20) | 20 | 0,000 | 0,000 to 16,843 | 0,22 | 0,22 |
| Ruano 2013 (n=17) | 17 | 0,000 | 0,000 to 19,506 | 0,19 | 0,19 |
| Arens 2017 (n=59) | 59 | 0,000 | 0,000 to 6,061 | 0,63 | 0,63 |
| Belfort 2017 (n=22) | 22 | 0,000 | 0,000 to 15,437 | 0,24 | 0,24 |
| Bruner 2000 (n=4) | 4 | 0,000 | 0,000 to 60,236 | 0,052 | 0,052 |
| Degenhardt 2014 (n=51) | 51 | 0,000 | 0,000 to 6,978 | 0,55 | 0,55 |
| Kohn 2018 (n=34) | 34 | 0,000 | 0,000 to 10,282 | 0,37 | 0,37 |
| Pedreira 2014 (n=4) | 4 | 0,000 | 0,000 to 60,236 | 0,052 | 0,052 |
| Pedreira 2016 (n=10) | 10 | 0,000 | 0,000 to 30,850 | 0,12 | 0,12 |
| Verbeek 2012 (n=19) | 19 | 0,000 | 0,000 to 17,647 | 0,21 | 0,21 |
| Ziemann 2018 (n=65) | 65 | 0,000 | 0,000 to 5,517 | 0,69 | 0,69 |
| Morris 2013 (n=16) | 16 | 0,000 | 0,000 to 20,591 | 0,18 | 0,18 |
| Ruano 2010 (n=11) | 11 | 0,000 | 0,000 to 28,491 | 0,13 | 0,13 |
| Welsh 2003 (n=13) | 13 | 0,000 | 0,000 to 24,705 | 0,15 | 0,15 |
| Cavalheiro 2011 (n=30) | 30 | 0,000 | 0,000 to 11,570 | 0,33 | 0,33 |
| Mallman 2017 (n=78) | 78 | 0,000 | 0,000 to 4,619 | 0,83 | 0,83 |
| Golombeck 2006 (n=99) | 99 | 0,000 | 0,000 to 3,658 | 1,05 | 1,05 |
| Kohl 2006 (n=16) | 16 | 0,000 | 0,000 to 20,591 | 0,18 | 0,18 |
| Kohl 2010 (n=37) | 37 | 0,000 | 0,000 to 9,489 | 0,40 | 0,40 |
| Nivatpumin 2016 (n=152) | 152 | 0,000 | 0,000 to 2,398 | 1,61 | 1,61 |
| Peralta 2010 (n=56) | 56 | 1,786 | 0,0452 to 9,553 | 0,60 | 0,60 |
| Total (fixed effects) | 9403 | 0,269 | 0,175 to 0,395 | 100,00 | 100,00 |
| Total (random effects) | 9403 | 0,269 | 0,175 to 0,383 | 100,00 | 100,00 |

## Test for heterogeneity

| Q | 40,5647 |
| --- | --- |
| DF | 121 |
| Significance level | P = 1,0000 |
| I^2^ (inconsistency) | 0,00% |
| 95% CI for I^2^ | 0,00 to 0,00 |

# Meta-analysis: intraoperative fetoscopic skin burns

| Variable for studies | Study |
| --- | --- |
| Variable for total number of cases | N |
| Variable for number of positive cases | Outcome |

| Study | Sample size | Proportion (%) | 95% CI | Weight (%) | |
| --- | --- | --- | --- | --- | --- |
|  |  |  |  | Fixed | Random |
| Aboudiab 2017 (n=18) | 18 | 0,000 | 0,000 to 18,530 | 0,20 | 0,20 |
| Baschat 2013 (n=147) | 147 | 0,000 | 0,000 to 2,478 | 1,55 | 1,55 |
| Chalouhi 2016 (n=22) | 22 | 0,000 | 0,000 to 15,437 | 0,24 | 0,24 |
| Chang 2006 (n=27) | 27 | 0,000 | 0,000 to 12,770 | 0,29 | 0,29 |
| Chang 2016 (n=100) | 100 | 0,000 | 0,000 to 3,622 | 1,06 | 1,06 |
| Chmait 2013 (n=318) | 318 | 0,000 | 0,000 to 1,153 | 3,35 | 3,35 |
| Chmait 2017 (n=19) | 19 | 0,000 | 0,000 to 17,647 | 0,21 | 0,21 |
| Crombleholme 2007 (n=20) | 20 | 0,000 | 0,000 to 16,843 | 0,22 | 0,22 |
| De Lia 1995 (n=26) | 26 | 0,000 | 0,000 to 13,227 | 0,28 | 0,28 |
| De Lia 1999 (n=67) | 67 | 0,000 | 0,000 to 5,357 | 0,71 | 0,71 |
| De Lia 2009 (n=10) | 10 | 0,000 | 0,000 to 30,850 | 0,12 | 0,12 |
| Deprest 1998 (n=6) | 6 | 0,000 | 0,000 to 45,926 | 0,073 | 0,073 |
| Draga 2016 (n=37) | 37 | 0,000 | 0,000 to 9,489 | 0,40 | 0,40 |
| Duron 2014 (n=85) | 85 | 0,000 | 0,000 to 4,247 | 0,90 | 0,90 |
| Ek 2012 (n=) | 67 | 0,000 | 0,000 to 5,357 | 0,71 | 0,71 |
| Habli 2009 (n=152) | 152 | 0,000 | 0,000 to 2,398 | 1,61 | 1,61 |
| Has 2014 (n=85) | 85 | 0,000 | 0,000 to 4,247 | 0,90 | 0,90 |
| Hecher 2000 (n=200) | 200 | 0,000 | 0,000 to 1,828 | 2,11 | 2,11 |
| Hernandez-Andrade 2011 (n=35) | 35 | 0,000 | 0,000 to 10,003 | 0,38 | 0,38 |
| Huber 2008 (n=176) | 176 | 0,000 | 0,000 to 2,074 | 1,86 | 1,86 |
| Ishii 2014 (n=16) | 16 | 0,000 | 0,000 to 20,591 | 0,18 | 0,18 |
| Ishii 2015 (n=10) | 10 | 0,000 | 0,000 to 30,850 | 0,12 | 0,12 |
| Lanna 2017 (n=373) | 373 | 0,000 | 0,000 to 0,984 | 3,93 | 3,93 |
| Lecointre 2017 (n=200) | 200 | 0,000 | 0,000 to 1,828 | 2,11 | 2,11 |
| Malshe 2017 (n=203) | 203 | 0,000 | 0,000 to 1,801 | 2,14 | 2,14 |
| Martinez 2012 (n=500) | 500 | 0,000 | 0,000 to 0,735 | 5,26 | 5,26 |
| Middeldorp 2007 (n=100) | 100 | 0,000 | 0,000 to 3,622 | 1,06 | 1,06 |
| Miyadahira 2018 (n=67) | 67 | 0,000 | 0,000 to 5,357 | 0,71 | 0,71 |
| Molina-Garcia 2009 (n=22) | 22 | 0,000 | 0,000 to 15,437 | 0,24 | 0,24 |
| Morris 2010 (n=164) | 164 | 0,000 | 0,000 to 2,224 | 1,73 | 1,73 |
| Mullers 2015 (n=105) | 105 | 0,000 | 0,000 to 3,452 | 1,11 | 1,11 |
| Nakata 2016 (n=6) | 6 | 0,000 | 0,000 to 45,926 | 0,073 | 0,073 |
| Nguyen 2012 (n=98) | 98 | 0,000 | 0,000 to 3,694 | 1,04 | 1,04 |
| Ozawa 2017 (n=11) | 11 | 0,000 | 0,000 to 28,491 | 0,13 | 0,13 |
| Papanna 2010 (n=48) | 48 | 0,000 | 0,000 to 7,397 | 0,51 | 0,51 |
| Papanna 2012 (n=163) | 163 | 0,000 | 0,000 to 2,238 | 1,72 | 1,72 |
| Peeters 2014 (n=338) | 338 | 0,000 | 0,000 to 1,085 | 3,56 | 3,56 |
| Persico 2016 (n=106) | 106 | 0,000 | 0,000 to 3,420 | 1,12 | 1,12 |
| Quintero 2000 (n=92) | 92 | 0,000 | 0,000 to 3,930 | 0,98 | 0,98 |
| Quintero 2001 (n=11) | 11 | 0,000 | 0,000 to 28,491 | 0,13 | 0,13 |
| Rossi 2008 (n=266) | 266 | 0,000 | 0,000 to 1,377 | 2,80 | 2,80 |
| Ruano 2009 (n=19) | 19 | 0,000 | 0,000 to 17,647 | 0,21 | 0,21 |
| Ruegg 2018 (n=37) | 37 | 0,000 | 0,000 to 9,489 | 0,40 | 0,40 |
| Rustico 2012 (n=150) | 150 | 0,000 | 0,000 to 2,429 | 1,59 | 1,59 |
| Said 2008 (n=10) | 10 | 0,000 | 0,000 to 30,850 | 0,12 | 0,12 |
| Senat 2004 (n=72) | 72 | 0,000 | 0,000 to 4,994 | 0,77 | 0,77 |
| Sepulveda 2007 (n=33) | 33 | 0,000 | 0,000 to 10,576 | 0,36 | 0,36 |
| Shamshirsaz 2015 (n=55) | 55 | 0,000 | 0,000 to 6,487 | 0,59 | 0,59 |
| Slaghekke 2014 (n=274) | 274 | 0,000 | 0,000 to 1,337 | 2,89 | 2,89 |
| Taniguchi 2015 (n=3) | 3 | 0,000 | 0,000 to 70,760 | 0,042 | 0,042 |
| Tchirikov 2011 (n=80) | 80 | 0,000 | 0,000 to 4,506 | 0,85 | 0,85 |
| Teoh 2013 (n=49) | 49 | 0,000 | 0,000 to 7,252 | 0,52 | 0,52 |
| Thia 2017 (n=5) | 5 | 0,000 | 0,000 to 52,182 | 0,063 | 0,063 |
| Ville 1997 (n=132) | 132 | 0,000 | 0,000 to 2,756 | 1,40 | 1,40 |
| Ville 1998 (n=44) | 44 | 0,000 | 0,000 to 8,042 | 0,47 | 0,47 |
| Weingertner 2011 (n=100) | 100 | 0,000 | 0,000 to 3,622 | 1,06 | 1,06 |
| Wilson 2016 (n=151) | 151 | 0,000 | 0,000 to 2,413 | 1,60 | 1,60 |
| Yamamoto 2005 (n=175) | 175 | 0,000 | 0,000 to 2,086 | 1,85 | 1,85 |
| Yang 2010 (n=30) | 30 | 0,000 | 0,000 to 11,570 | 0,33 | 0,33 |
| Zaretsky 2018 (n=749) | 749 | 0,000 | 0,000 to 0,491 | 7,87 | 7,87 |
| Zhao 2016 (n=62) | 62 | 0,000 | 0,000 to 5,776 | 0,66 | 0,66 |
| Bebbington 2012 (n=146) | 146 | 0,000 | 0,000 to 2,495 | 1,54 | 1,54 |
| Berg 2014 (n=7) | 7 | 0,000 | 0,000 to 40,962 | 0,084 | 0,084 |
| Delabaere 2013 (n=30) | 30 | 0,000 | 0,000 to 11,570 | 0,33 | 0,33 |
| Deprest 2000 (n=10) | 10 | 0,000 | 0,000 to 30,850 | 0,12 | 0,12 |
| Gallot 2003 (n=11) | 11 | 0,000 | 0,000 to 28,491 | 0,13 | 0,13 |
| Gouverneur 2009 (n=54) | 54 | 0,000 | 0,000 to 6,603 | 0,58 | 0,58 |
| Gul 2008 (n=9) | 9 | 0,000 | 0,000 to 33,627 | 0,10 | 0,10 |
| Has 2014 (n=71) | 71 | 0,000 | 0,000 to 5,063 | 0,76 | 0,76 |
| He 2010 (n=14) | 14 | 0,000 | 0,000 to 23,164 | 0,16 | 0,16 |
| Ilagan 2008 (n=27) | 27 | 0,000 | 0,000 to 12,770 | 0,29 | 0,29 |
| Jelin 2010 (n=7) | 7 | 0,000 | 0,000 to 40,962 | 0,084 | 0,084 |
| King 2017 (n=43) | 43 | 0,000 | 0,000 to 8,221 | 0,46 | 0,46 |
| Lanna 2012 (n=118) | 118 | 0,000 | 0,000 to 3,078 | 1,25 | 1,25 |
| Lee 2013 (n=98) | 98 | 2,041 | 0,248 to 7,178 | 1,04 | 1,04 |
| Lewi 2006 (n=80) | 80 | 0,000 | 0,000 to 4,506 | 0,85 | 0,85 |
| Moise 2008 (n=9) | 9 | 0,000 | 0,000 to 33,627 | 0,10 | 0,10 |
| Nobili 2013 (n=48) | 48 | 0,000 | 0,000 to 7,397 | 0,51 | 0,51 |
| Paramasivam 2010 (n=35) | 35 | 0,000 | 0,000 to 10,003 | 0,38 | 0,38 |
| Peng 2016 (n=93) | 93 | 0,000 | 0,000 to 3,889 | 0,99 | 0,99 |
| Quintero 1996 (n=13) | 13 | 0,000 | 0,000 to 24,705 | 0,15 | 0,15 |
| Quintero 2006 (n=51) | 51 | 0,000 | 0,000 to 6,978 | 0,55 | 0,55 |
| Roman 2010 (n=60) | 60 | 0,000 | 0,000 to 5,963 | 0,64 | 0,64 |
| Schou 2018 (n=102) | 102 | 0,000 | 0,000 to 3,552 | 1,08 | 1,08 |
| Sugibayashi 2016 (n=40) | 40 | 2,500 | 0,0633 to 13,159 | 0,43 | 0,43 |
| Takano 2015 (n=10) | 10 | 0,000 | 0,000 to 30,850 | 0,12 | 0,12 |
| Taylor 2002 (n=15) | 15 | 0,000 | 0,000 to 21,802 | 0,17 | 0,17 |
| Tsao 2002 (n=13) | 13 | 7,692 | 0,195 to 36,030 | 0,15 | 0,15 |
| Zhang 2018 (n=25) | 25 | 0,000 | 0,000 to 13,719 | 0,27 | 0,27 |
| Deprest 2005 (n=20) | 20 | 0,000 | 0,000 to 16,843 | 0,22 | 0,22 |
| Harrison 1998 (n=8) | 8 | 0,000 | 0,000 to 36,942 | 0,094 | 0,094 |
| Harrison 2003 (n=11) | 11 | 0,000 | 0,000 to 28,491 | 0,13 | 0,13 |
| Jani 2005 (n=24) | 24 | 0,000 | 0,000 to 14,247 | 0,26 | 0,26 |
| Jani 2006 (n=28) | 28 | 0,000 | 0,000 to 12,344 | 0,30 | 0,30 |
| Jani 2009 (n=210) | 210 | 0,000 | 0,000 to 1,741 | 2,22 | 2,22 |
| Jimenez 2017 (n=201) | 201 | 0,000 | 0,000 to 1,819 | 2,12 | 2,12 |
| Kosinski 2017 (n=28) | 28 | 0,000 | 0,000 to 12,344 | 0,30 | 0,30 |
| Manrique 2008 (n=11) | 11 | 0,000 | 0,000 to 28,491 | 0,13 | 0,13 |
| Peralta 2011 (n=8) | 8 | 0,000 | 0,000 to 36,942 | 0,094 | 0,094 |
| Persico 2017 (n=21) | 21 | 0,000 | 0,000 to 16,110 | 0,23 | 0,23 |
| Ruano 2012 (n=35) | 35 | 0,000 | 0,000 to 10,003 | 0,38 | 0,38 |
| Ruano 2012 (n=20) | 20 | 0,000 | 0,000 to 16,843 | 0,22 | 0,22 |
| Ruano 2013 (n=17) | 17 | 0,000 | 0,000 to 19,506 | 0,19 | 0,19 |
| Arens 2017 (n=59) | 59 | 0,000 | 0,000 to 6,061 | 0,63 | 0,63 |
| Belfort 2017 (n=22) | 22 | 0,000 | 0,000 to 15,437 | 0,24 | 0,24 |
| Bruner 2000 (n=4) | 4 | 0,000 | 0,000 to 60,236 | 0,052 | 0,052 |
| Degenhardt 2014 (n=51) | 51 | 0,000 | 0,000 to 6,978 | 0,55 | 0,55 |
| Kohn 2018 (n=34) | 34 | 0,000 | 0,000 to 10,282 | 0,37 | 0,37 |
| Pedreira 2014 (n=4) | 4 | 0,000 | 0,000 to 60,236 | 0,052 | 0,052 |
| Pedreira 2016 (n=10) | 10 | 0,000 | 0,000 to 30,850 | 0,12 | 0,12 |
| Verbeek 2012 (n=19) | 19 | 0,000 | 0,000 to 17,647 | 0,21 | 0,21 |
| Ziemann 2018 (n=65) | 65 | 0,000 | 0,000 to 5,517 | 0,69 | 0,69 |
| Morris 2013 (n=16) | 16 | 0,000 | 0,000 to 20,591 | 0,18 | 0,18 |
| Ruano 2010 (n=11) | 11 | 0,000 | 0,000 to 28,491 | 0,13 | 0,13 |
| Welsh 2003 (n=13) | 13 | 0,000 | 0,000 to 24,705 | 0,15 | 0,15 |
| Cavalheiro 2011 (n=30) | 30 | 0,000 | 0,000 to 11,570 | 0,33 | 0,33 |
| Mallman 2017 (n=78) | 78 | 0,000 | 0,000 to 4,619 | 0,83 | 0,83 |
| Golombeck 2006 (n=99) | 99 | 0,000 | 0,000 to 3,658 | 1,05 | 1,05 |
| Kohl 2006 (n=16) | 16 | 0,000 | 0,000 to 20,591 | 0,18 | 0,18 |
| Kohl 2010 (n=37) | 37 | 0,000 | 0,000 to 9,489 | 0,40 | 0,40 |
| Nivatpumin 2016 (n=152) | 152 | 0,000 | 0,000 to 2,398 | 1,61 | 1,61 |
| Peralta 2010 (n=56) | 56 | 0,000 | 0,000 to 6,375 | 0,60 | 0,60 |
| Total (fixed effects) | 9403 | 0,257 | 0,165 to 0,380 | 100,00 | 100,00 |
| Total (random effects) | 9403 | 0,257 | 0,165 to 0,368 | 100,00 | 100,00 |

## Test for heterogeneity

| Q | 44,9028 |
| --- | --- |
| DF | 121 |
| Significance level | P = 1,0000 |
| I^2^ (inconsistency) | 0,00% |
| 95% CI for I^2^ | 0,00 to 0,00 |

# Meta-analysis: postoperative fetoscopic delivery

| Variable for studies | Study |
| --- | --- |
| Variable for total number of cases | N |
| Variable for number of positive cases | Outcome |

| Study | Sample size | Proportion (%) | 95% CI | Weight (%) | |
| --- | --- | --- | --- | --- | --- |
|  |  |  |  | Fixed | Random |
| Aboudiab 2017 (n=18) | 18 | 0,000 | 0,000 to 18,530 | 0,20 | 0,20 |
| Baschat 2013 (n=147) | 147 | 0,000 | 0,000 to 2,478 | 1,55 | 1,55 |
| Chalouhi 2016 (n=22) | 22 | 0,000 | 0,000 to 15,437 | 0,24 | 0,24 |
| Chang 2006 (n=27) | 27 | 0,000 | 0,000 to 12,770 | 0,29 | 0,29 |
| Chang 2016 (n=100) | 100 | 0,000 | 0,000 to 3,622 | 1,06 | 1,06 |
| Chmait 2013 (n=318) | 318 | 0,000 | 0,000 to 1,153 | 3,35 | 3,35 |
| Chmait 2017 (n=19) | 19 | 0,000 | 0,000 to 17,647 | 0,21 | 0,21 |
| Crombleholme 2007 (n=20) | 20 | 0,000 | 0,000 to 16,843 | 0,22 | 0,22 |
| De Lia 1995 (n=26) | 26 | 0,000 | 0,000 to 13,227 | 0,28 | 0,28 |
| De Lia 1999 (n=67) | 67 | 0,000 | 0,000 to 5,357 | 0,71 | 0,71 |
| De Lia 2009 (n=10) | 10 | 0,000 | 0,000 to 30,850 | 0,12 | 0,12 |
| Deprest 1998 (n=6) | 6 | 0,000 | 0,000 to 45,926 | 0,073 | 0,073 |
| Draga 2016 (n=37) | 37 | 0,000 | 0,000 to 9,489 | 0,40 | 0,40 |
| Duron 2014 (n=85) | 85 | 0,000 | 0,000 to 4,247 | 0,90 | 0,90 |
| Ek 2012 (n=) | 67 | 0,000 | 0,000 to 5,357 | 0,71 | 0,71 |
| Habli 2009 (n=152) | 152 | 0,000 | 0,000 to 2,398 | 1,61 | 1,61 |
| Has 2014 (n=85) | 85 | 0,000 | 0,000 to 4,247 | 0,90 | 0,90 |
| Hecher 2000 (n=200) | 200 | 0,000 | 0,000 to 1,828 | 2,11 | 2,11 |
| Hernandez-Andrade 2011 (n=35) | 35 | 0,000 | 0,000 to 10,003 | 0,38 | 0,38 |
| Huber 2008 (n=176) | 176 | 0,000 | 0,000 to 2,074 | 1,86 | 1,86 |
| Ishii 2014 (n=16) | 16 | 0,000 | 0,000 to 20,591 | 0,18 | 0,18 |
| Ishii 2015 (n=10) | 10 | 0,000 | 0,000 to 30,850 | 0,12 | 0,12 |
| Lanna 2017 (n=373) | 373 | 0,000 | 0,000 to 0,984 | 3,93 | 3,93 |
| Lecointre 2017 (n=200) | 200 | 0,000 | 0,000 to 1,828 | 2,11 | 2,11 |
| Malshe 2017 (n=203) | 203 | 0,000 | 0,000 to 1,801 | 2,14 | 2,14 |
| Martinez 2012 (n=500) | 500 | 0,000 | 0,000 to 0,735 | 5,26 | 5,26 |
| Middeldorp 2007 (n=100) | 100 | 0,000 | 0,000 to 3,622 | 1,06 | 1,06 |
| Miyadahira 2018 (n=67) | 67 | 0,000 | 0,000 to 5,357 | 0,71 | 0,71 |
| Molina-Garcia 2009 (n=22) | 22 | 0,000 | 0,000 to 15,437 | 0,24 | 0,24 |
| Morris 2010 (n=164) | 164 | 0,000 | 0,000 to 2,224 | 1,73 | 1,73 |
| Mullers 2015 (n=105) | 105 | 0,000 | 0,000 to 3,452 | 1,11 | 1,11 |
| Nakata 2016 (n=6) | 6 | 0,000 | 0,000 to 45,926 | 0,073 | 0,073 |
| Nguyen 2012 (n=98) | 98 | 0,000 | 0,000 to 3,694 | 1,04 | 1,04 |
| Ozawa 2017 (n=11) | 11 | 0,000 | 0,000 to 28,491 | 0,13 | 0,13 |
| Papanna 2010 (n=48) | 48 | 0,000 | 0,000 to 7,397 | 0,51 | 0,51 |
| Papanna 2012 (n=163) | 163 | 0,000 | 0,000 to 2,238 | 1,72 | 1,72 |
| Peeters 2014 (n=338) | 338 | 0,000 | 0,000 to 1,085 | 3,56 | 3,56 |
| Persico 2016 (n=106) | 106 | 0,000 | 0,000 to 3,420 | 1,12 | 1,12 |
| Quintero 2000 (n=92) | 92 | 0,000 | 0,000 to 3,930 | 0,98 | 0,98 |
| Quintero 2001 (n=11) | 11 | 0,000 | 0,000 to 28,491 | 0,13 | 0,13 |
| Rossi 2008 (n=266) | 266 | 0,000 | 0,000 to 1,377 | 2,80 | 2,80 |
| Ruano 2009 (n=19) | 19 | 0,000 | 0,000 to 17,647 | 0,21 | 0,21 |
| Ruegg 2018 (n=37) | 37 | 0,000 | 0,000 to 9,489 | 0,40 | 0,40 |
| Rustico 2012 (n=150) | 150 | 0,000 | 0,000 to 2,429 | 1,59 | 1,59 |
| Said 2008 (n=10) | 10 | 0,000 | 0,000 to 30,850 | 0,12 | 0,12 |
| Senat 2004 (n=72) | 72 | 0,000 | 0,000 to 4,994 | 0,77 | 0,77 |
| Sepulveda 2007 (n=33) | 33 | 0,000 | 0,000 to 10,576 | 0,36 | 0,36 |
| Shamshirsaz 2015 (n=55) | 55 | 0,000 | 0,000 to 6,487 | 0,59 | 0,59 |
| Slaghekke 2014 (n=274) | 274 | 0,000 | 0,000 to 1,337 | 2,89 | 2,89 |
| Taniguchi 2015 (n=3) | 3 | 0,000 | 0,000 to 70,760 | 0,042 | 0,042 |
| Tchirikov 2011 (n=80) | 80 | 0,000 | 0,000 to 4,506 | 0,85 | 0,85 |
| Teoh 2013 (n=49) | 49 | 0,000 | 0,000 to 7,252 | 0,52 | 0,52 |
| Thia 2017 (n=5) | 5 | 0,000 | 0,000 to 52,182 | 0,063 | 0,063 |
| Ville 1997 (n=132) | 132 | 0,000 | 0,000 to 2,756 | 1,40 | 1,40 |
| Ville 1998 (n=44) | 44 | 0,000 | 0,000 to 8,042 | 0,47 | 0,47 |
| Weingertner 2011 (n=100) | 100 | 0,000 | 0,000 to 3,622 | 1,06 | 1,06 |
| Wilson 2016 (n=151) | 151 | 0,000 | 0,000 to 2,413 | 1,60 | 1,60 |
| Yamamoto 2005 (n=175) | 175 | 0,571 | 0,0145 to 3,143 | 1,85 | 1,85 |
| Yang 2010 (n=30) | 30 | 0,000 | 0,000 to 11,570 | 0,33 | 0,33 |
| Zaretsky 2018 (n=749) | 749 | 0,000 | 0,000 to 0,491 | 7,87 | 7,87 |
| Zhao 2016 (n=62) | 62 | 0,000 | 0,000 to 5,776 | 0,66 | 0,66 |
| Bebbington 2012 (n=146) | 146 | 0,000 | 0,000 to 2,495 | 1,54 | 1,54 |
| Berg 2014 (n=7) | 7 | 0,000 | 0,000 to 40,962 | 0,084 | 0,084 |
| Delabaere 2013 (n=30) | 30 | 0,000 | 0,000 to 11,570 | 0,33 | 0,33 |
| Deprest 2000 (n=10) | 10 | 0,000 | 0,000 to 30,850 | 0,12 | 0,12 |
| Gallot 2003 (n=11) | 11 | 0,000 | 0,000 to 28,491 | 0,13 | 0,13 |
| Gouverneur 2009 (n=54) | 54 | 0,000 | 0,000 to 6,603 | 0,58 | 0,58 |
| Gul 2008 (n=9) | 9 | 0,000 | 0,000 to 33,627 | 0,10 | 0,10 |
| Has 2014 (n=71) | 71 | 0,000 | 0,000 to 5,063 | 0,76 | 0,76 |
| He 2010 (n=14) | 14 | 0,000 | 0,000 to 23,164 | 0,16 | 0,16 |
| Ilagan 2008 (n=27) | 27 | 0,000 | 0,000 to 12,770 | 0,29 | 0,29 |
| Jelin 2010 (n=7) | 7 | 0,000 | 0,000 to 40,962 | 0,084 | 0,084 |
| King 2017 (n=43) | 43 | 0,000 | 0,000 to 8,221 | 0,46 | 0,46 |
| Lanna 2012 (n=118) | 118 | 0,000 | 0,000 to 3,078 | 1,25 | 1,25 |
| Lee 2013 (n=98) | 98 | 0,000 | 0,000 to 3,694 | 1,04 | 1,04 |
| Lewi 2006 (n=80) | 80 | 0,000 | 0,000 to 4,506 | 0,85 | 0,85 |
| Moise 2008 (n=9) | 9 | 0,000 | 0,000 to 33,627 | 0,10 | 0,10 |
| Nobili 2013 (n=48) | 48 | 0,000 | 0,000 to 7,397 | 0,51 | 0,51 |
| Paramasivam 2010 (n=35) | 35 | 0,000 | 0,000 to 10,003 | 0,38 | 0,38 |
| Peng 2016 (n=93) | 93 | 0,000 | 0,000 to 3,889 | 0,99 | 0,99 |
| Quintero 1996 (n=13) | 13 | 0,000 | 0,000 to 24,705 | 0,15 | 0,15 |
| Quintero 2006 (n=51) | 51 | 0,000 | 0,000 to 6,978 | 0,55 | 0,55 |
| Roman 2010 (n=60) | 60 | 0,000 | 0,000 to 5,963 | 0,64 | 0,64 |
| Schou 2018 (n=102) | 102 | 0,000 | 0,000 to 3,552 | 1,08 | 1,08 |
| Sugibayashi 2016 (n=40) | 40 | 0,000 | 0,000 to 8,810 | 0,43 | 0,43 |
| Takano 2015 (n=10) | 10 | 0,000 | 0,000 to 30,850 | 0,12 | 0,12 |
| Taylor 2002 (n=15) | 15 | 6,667 | 0,169 to 31,948 | 0,17 | 0,17 |
| Tsao 2002 (n=13) | 13 | 0,000 | 0,000 to 24,705 | 0,15 | 0,15 |
| Zhang 2018 (n=25) | 25 | 0,000 | 0,000 to 13,719 | 0,27 | 0,27 |
| Deprest 2005 (n=20) | 20 | 0,000 | 0,000 to 16,843 | 0,22 | 0,22 |
| Harrison 1998 (n=8) | 8 | 0,000 | 0,000 to 36,942 | 0,094 | 0,094 |
| Harrison 2003 (n=11) | 11 | 0,000 | 0,000 to 28,491 | 0,13 | 0,13 |
| Jani 2005 (n=24) | 24 | 0,000 | 0,000 to 14,247 | 0,26 | 0,26 |
| Jani 2006 (n=28) | 28 | 0,000 | 0,000 to 12,344 | 0,30 | 0,30 |
| Jani 2009 (n=210) | 210 | 0,000 | 0,000 to 1,741 | 2,22 | 2,22 |
| Jimenez 2017 (n=201) | 201 | 0,000 | 0,000 to 1,819 | 2,12 | 2,12 |
| Kosinski 2017 (n=28) | 28 | 0,000 | 0,000 to 12,344 | 0,30 | 0,30 |
| Manrique 2008 (n=11) | 11 | 0,000 | 0,000 to 28,491 | 0,13 | 0,13 |
| Peralta 2011 (n=8) | 8 | 0,000 | 0,000 to 36,942 | 0,094 | 0,094 |
| Persico 2017 (n=21) | 21 | 0,000 | 0,000 to 16,110 | 0,23 | 0,23 |
| Ruano 2012 (n=35) | 35 | 0,000 | 0,000 to 10,003 | 0,38 | 0,38 |
| Ruano 2012 (n=20) | 20 | 0,000 | 0,000 to 16,843 | 0,22 | 0,22 |
| Ruano 2013 (n=17) | 17 | 0,000 | 0,000 to 19,506 | 0,19 | 0,19 |
| Arens 2017 (n=59) | 59 | 0,000 | 0,000 to 6,061 | 0,63 | 0,63 |
| Belfort 2017 (n=22) | 22 | 0,000 | 0,000 to 15,437 | 0,24 | 0,24 |
| Bruner 2000 (n=4) | 4 | 0,000 | 0,000 to 60,236 | 0,052 | 0,052 |
| Degenhardt 2014 (n=51) | 51 | 0,000 | 0,000 to 6,978 | 0,55 | 0,55 |
| Kohn 2018 (n=34) | 34 | 0,000 | 0,000 to 10,282 | 0,37 | 0,37 |
| Pedreira 2014 (n=4) | 4 | 0,000 | 0,000 to 60,236 | 0,052 | 0,052 |
| Pedreira 2016 (n=10) | 10 | 0,000 | 0,000 to 30,850 | 0,12 | 0,12 |
| Verbeek 2012 (n=19) | 19 | 0,000 | 0,000 to 17,647 | 0,21 | 0,21 |
| Ziemann 2018 (n=65) | 65 | 0,000 | 0,000 to 5,517 | 0,69 | 0,69 |
| Morris 2013 (n=16) | 16 | 0,000 | 0,000 to 20,591 | 0,18 | 0,18 |
| Ruano 2010 (n=11) | 11 | 0,000 | 0,000 to 28,491 | 0,13 | 0,13 |
| Welsh 2003 (n=13) | 13 | 0,000 | 0,000 to 24,705 | 0,15 | 0,15 |
| Cavalheiro 2011 (n=30) | 30 | 0,000 | 0,000 to 11,570 | 0,33 | 0,33 |
| Mallman 2017 (n=78) | 78 | 0,000 | 0,000 to 4,619 | 0,83 | 0,83 |
| Golombeck 2006 (n=99) | 99 | 0,000 | 0,000 to 3,658 | 1,05 | 1,05 |
| Kohl 2006 (n=16) | 16 | 6,250 | 0,158 to 30,232 | 0,18 | 0,18 |
| Kohl 2010 (n=37) | 37 | 0,000 | 0,000 to 9,489 | 0,40 | 0,40 |
| Nivatpumin 2016 (n=152) | 152 | 0,000 | 0,000 to 2,398 | 1,61 | 1,61 |
| Peralta 2010 (n=56) | 56 | 0,000 | 0,000 to 6,375 | 0,60 | 0,60 |
| Total (fixed effects) | 9403 | 0,254 | 0,163 to 0,377 | 100,00 | 100,00 |
| Total (random effects) | 9403 | 0,254 | 0,163 to 0,365 | 100,00 | 100,00 |

## Test for heterogeneity

| Q | 41,9514 |
| --- | --- |
| DF | 121 |
| Significance level | P = 1,0000 |
| I^2^ (inconsistency) | 0,00% |
| 95% CI for I^2^ | 0,00 to 0,00 |

# Meta-analysis: postoperative open abruption

| Variable for studies | Study |
| --- | --- |
| Variable for total number of cases | N |
| Variable for number of positive cases | Outcome |

| Study | Sample size | Proportion (%) | 95% CI | Weight (%) | |
| --- | --- | --- | --- | --- | --- |
|  |  |  |  | Fixed | Random |
| Barthod 2013 (n=5) | 5 | 0,000 | 0,000 to 52,182 | 0,49 | 0,49 |
| Cass 2013 (n=9) | 9 | 0,000 | 0,000 to 33,627 | 0,81 | 0,81 |
| Chen 2018 (n=7) | 7 | 0,000 | 0,000 to 40,962 | 0,65 | 0,65 |
| Dahlgren 2004 (n=4) | 4 | 0,000 | 0,000 to 60,236 | 0,40 | 0,40 |
| Flake 2000 (n=15) | 15 | 0,000 | 0,000 to 21,802 | 1,29 | 1,29 |
| George 2007 (n=3) | 3 | 0,000 | 0,000 to 70,760 | 0,32 | 0,32 |
| Hedrick 2003 (n=43) | 43 | 0,000 | 0,000 to 8,221 | 3,56 | 3,56 |
| Hedrick 2005 (n=9) | 9 | 0,000 | 0,000 to 33,627 | 0,81 | 0,81 |
| Kern 2007 (n=5) | 5 | 0,000 | 0,000 to 52,182 | 0,49 | 0,49 |
| Kornacki 2017 (n=4) | 4 | 0,000 | 0,000 to 60,236 | 0,40 | 0,40 |
| Kunisaki 2007 (n=14) | 14 | 0,000 | 0,000 to 23,164 | 1,21 | 1,21 |
| Laje 2012 (n=17) | 17 | 0,000 | 0,000 to 19,506 | 1,46 | 1,46 |
| Laje 2013 (n=4) | 4 | 0,000 | 0,000 to 60,236 | 0,40 | 0,40 |
| Laje 2015 (n=13) | 13 | 0,000 | 0,000 to 24,705 | 1,13 | 1,13 |
| Lazar 2011 (n=12) | 12 | 0,000 | 0,000 to 26,465 | 1,05 | 1,05 |
| Noah 2002 (n=34) | 34 | 0,000 | 0,000 to 10,282 | 2,83 | 2,83 |
| Pellicer 2007 (n=3) | 3 | 0,000 | 0,000 to 70,760 | 0,32 | 0,32 |
| Stoffan 2012 (n=7) | 7 | 0,000 | 0,000 to 40,962 | 0,65 | 0,65 |
| Tuncay Ozgunen 2010 (n=3) | 3 | 0,000 | 0,000 to 70,760 | 0,32 | 0,32 |
| Zamora 2013 (n=26) | 26 | 3,846 | 0,0973 to 19,637 | 2,18 | 2,18 |
| Bennett 2014 (n=43) | 43 | 0,000 | 0,000 to 8,221 | 3,56 | 3,56 |
| Botelho 2017 (n=45) | 45 | 2,222 | 0,0562 to 11,770 | 3,72 | 3,72 |
| Bruner 1999 (n=29) | 29 | 0,000 | 0,000 to 11,944 | 2,43 | 2,43 |
| Bruner 2000 (n=4) | 4 | 0,000 | 0,000 to 60,236 | 0,40 | 0,40 |
| Farmer 2003 (n=12) | 12 | 0,000 | 0,000 to 26,465 | 1,05 | 1,05 |
| Friszer 2016 (n=3) | 3 | 0,000 | 0,000 to 70,760 | 0,32 | 0,32 |
| Johnson 2016 (n=91) | 91 | 6,593 | 2,458 to 13,800 | 7,44 | 7,44 |
| Marenco 2013 (n=4) | 4 | 0,000 | 0,000 to 60,236 | 0,40 | 0,40 |
| Moldenhauer 2015 (n=100) | 100 | 2,000 | 0,243 to 7,038 | 8,17 | 8,17 |
| Moron 2018 (n=237) | 237 | 0,000 | 0,000 to 1,544 | 19,26 | 19,26 |
| Ochsenbein-Kolble 2017 (n=30) | 30 | 0,000 | 0,000 to 11,570 | 2,51 | 2,51 |
| Sinskey 2017 (n=47) | 47 | 0,000 | 0,000 to 7,549 | 3,88 | 3,88 |
| Soni 2016 (n=88) | 88 | 0,000 | 0,000 to 4,105 | 7,20 | 7,20 |
| Zamlynski 2014 (n=46) | 46 | 4,348 | 0,531 to 14,839 | 3,80 | 3,80 |
| Flake 2000 (n=15) | 15 | 0,000 | 0,000 to 21,802 | 1,29 | 1,29 |
| Harrison 1990 (n=6) | 6 | 0,000 | 0,000 to 45,926 | 0,57 | 0,57 |
| Harrison 1993 (n=14) | 14 | 0,000 | 0,000 to 23,164 | 1,21 | 1,21 |
| Harrison 1998 (n=13) | 13 | 0,000 | 0,000 to 24,705 | 1,13 | 1,13 |
| Adzick 2003 (n=22) | 22 | 0,000 | 0,000 to 15,437 | 1,86 | 1,86 |
| Hedrick 2004 (n=4) | 4 | 0,000 | 0,000 to 60,236 | 0,40 | 0,40 |
| Golombeck 2006 (n=79) | 79 | 8,861 | 3,637 to 17,408 | 6,47 | 6,47 |
| Longaker 1991 (n=17) | 17 | 0,000 | 0,000 to 19,506 | 1,46 | 1,46 |
| Zamora 2013 (n=7) | 7 | 0,000 | 0,000 to 40,962 | 0,65 | 0,65 |
| Total (fixed effects) | 1193 | 1,809 | 1,142 to 2,717 | 100,00 | 100,00 |
| Total (random effects) | 1193 | 1,809 | 1,141 to 2,626 | 100,00 | 100,00 |

## Test for heterogeneity

| Q | 38,0185 |
| --- | --- |
| DF | 42 |
| Significance level | P = 0,6464 |
| I^2^ (inconsistency) | 0,00% |
| 95% CI for I^2^ | 0,00 to 28,35 |

# Meta-analysis: postoperative fetoscopic abruption

| Variable for studies | Study |
| --- | --- |
| Variable for total number of cases | N |
| Variable for number of positive cases | Outcome |

| Study | Sample size | Proportion (%) | 95% CI | Weight (%) | |
| --- | --- | --- | --- | --- | --- |
|  |  |  |  | Fixed | Random |
| Aboudiab 2017 (n=18) | 18 | 5,556 | 0,141 to 27,294 | 0,20 | 0,50 |
| Baschat 2013 (n=147) | 147 | 4,762 | 1,936 to 9,565 | 1,56 | 1,33 |
| Chalouhi 2016 (n=22) | 22 | 0,000 | 0,000 to 15,437 | 0,24 | 0,57 |
| Chang 2006 (n=27) | 27 | 0,000 | 0,000 to 12,770 | 0,30 | 0,65 |
| Chang 2016 (n=100) | 100 | 0,000 | 0,000 to 3,622 | 1,07 | 1,19 |
| Chmait 2013 (n=318) | 318 | 0,629 | 0,0763 to 2,253 | 3,37 | 1,53 |
| Chmait 2017 (n=19) | 19 | 0,000 | 0,000 to 17,647 | 0,21 | 0,52 |
| Crombleholme 2007 (n=20) | 20 | 0,000 | 0,000 to 16,843 | 0,22 | 0,54 |
| De Lia 1995 (n=26) | 26 | 0,000 | 0,000 to 13,227 | 0,29 | 0,63 |
| De Lia 1999 (n=67) | 67 | 0,000 | 0,000 to 5,357 | 0,72 | 1,03 |
| De Lia 2009 (n=10) | 10 | 0,000 | 0,000 to 30,850 | 0,12 | 0,33 |
| Deprest 1998 (n=6) | 6 | 0,000 | 0,000 to 45,926 | 0,074 | 0,22 |
| Draga 2016 (n=37) | 37 | 0,000 | 0,000 to 9,489 | 0,40 | 0,78 |
| Duron 2014 (n=85) | 85 | 0,000 | 0,000 to 4,247 | 0,91 | 1,13 |
| Ek 2012 (n=) | 67 | 4,478 | 0,933 to 12,533 | 0,72 | 1,03 |
| Habli 2009 (n=152) | 152 | 7,895 | 4,146 to 13,384 | 1,62 | 1,34 |
| Has 2014 (n=85) | 85 | 0,000 | 0,000 to 4,247 | 0,91 | 1,13 |
| Hecher 2000 (n=200) | 200 | 0,000 | 0,000 to 1,828 | 2,12 | 1,42 |
| Hernandez-Andrade 2011 (n=35) | 35 | 0,000 | 0,000 to 10,003 | 0,38 | 0,75 |
| Huber 2008 (n=176) | 176 | 0,568 | 0,0144 to 3,125 | 1,87 | 1,38 |
| Ishii 2014 (n=16) | 16 | 0,000 | 0,000 to 20,591 | 0,18 | 0,46 |
| Ishii 2015 (n=10) | 10 | 0,000 | 0,000 to 30,850 | 0,12 | 0,33 |
| Lanna 2017 (n=373) | 373 | 5,630 | 3,518 to 8,478 | 3,95 | 1,56 |
| Lecointre 2017 (n=200) | 200 | 0,000 | 0,000 to 1,828 | 2,12 | 1,42 |
| Malshe 2017 (n=203) | 203 | 5,911 | 3,091 to 10,098 | 2,16 | 1,42 |
| Martinez 2012 (n=500) | 500 | 0,000 | 0,000 to 0,735 | 5,29 | 1,61 |
| Middeldorp 2007 (n=100) | 100 | 0,000 | 0,000 to 3,622 | 1,07 | 1,19 |
| Miyadahira 2018 (n=67) | 67 | 1,493 | 0,0378 to 8,038 | 0,72 | 1,03 |
| Molina-Garcia 2009 (n=22) | 22 | 0,000 | 0,000 to 15,437 | 0,24 | 0,57 |
| Morris 2010 (n=164) | 164 | 0,000 | 0,000 to 2,224 | 1,74 | 1,36 |
| Mullers 2015 (n=105) | 105 | 0,000 | 0,000 to 3,452 | 1,12 | 1,21 |
| Nakata 2016 (n=6) | 6 | 0,000 | 0,000 to 45,926 | 0,074 | 0,22 |
| Nguyen 2012 (n=98) | 98 | 0,000 | 0,000 to 3,694 | 1,05 | 1,18 |
| Ozawa 2017 (n=11) | 11 | 0,000 | 0,000 to 28,491 | 0,13 | 0,35 |
| Papanna 2010 (n=48) | 48 | 0,000 | 0,000 to 7,397 | 0,52 | 0,89 |
| Papanna 2012 (n=163) | 163 | 3,067 | 1,003 to 7,013 | 1,73 | 1,36 |
| Peeters 2014 (n=338) | 338 | 0,000 | 0,000 to 1,085 | 3,58 | 1,54 |
| Persico 2016 (n=106) | 106 | 0,000 | 0,000 to 3,420 | 1,13 | 1,21 |
| Quintero 2000 (n=92) | 92 | 0,000 | 0,000 to 3,930 | 0,98 | 1,16 |
| Quintero 2001 (n=11) | 11 | 18,182 | 2,283 to 51,776 | 0,13 | 0,35 |
| Rossi 2008 (n=266) | 266 | 0,000 | 0,000 to 1,377 | 2,82 | 1,49 |
| Ruano 2009 (n=19) | 19 | 0,000 | 0,000 to 17,647 | 0,21 | 0,52 |
| Ruegg 2018 (n=37) | 37 | 2,703 | 0,0684 to 14,160 | 0,40 | 0,78 |
| Rustico 2012 (n=150) | 150 | 3,333 | 1,091 to 7,607 | 1,60 | 1,33 |
| Said 2008 (n=10) | 10 | 0,000 | 0,000 to 30,850 | 0,12 | 0,33 |
| Senat 2004 (n=72) | 72 | 1,389 | 0,0352 to 7,497 | 0,77 | 1,06 |
| Sepulveda 2007 (n=33) | 33 | 0,000 | 0,000 to 10,576 | 0,36 | 0,73 |
| Shamshirsaz 2015 (n=55) | 55 | 3,636 | 0,443 to 12,526 | 0,59 | 0,95 |
| Slaghekke 2014 (n=274) | 274 | 0,000 | 0,000 to 1,337 | 2,91 | 1,50 |
| Taniguchi 2015 (n=3) | 3 | 0,000 | 0,000 to 70,760 | 0,042 | 0,14 |
| Tchirikov 2011 (n=80) | 80 | 0,000 | 0,000 to 4,506 | 0,86 | 1,10 |
| Teoh 2013 (n=49) | 49 | 0,000 | 0,000 to 7,252 | 0,53 | 0,90 |
| Thia 2017 (n=5) | 5 | 0,000 | 0,000 to 52,182 | 0,063 | 0,20 |
| Ville 1997 (n=132) | 132 | 0,000 | 0,000 to 2,756 | 1,41 | 1,29 |
| Ville 1998 (n=44) | 44 | 0,000 | 0,000 to 8,042 | 0,48 | 0,85 |
| Weingertner 2011 (n=100) | 100 | 0,000 | 0,000 to 3,622 | 1,07 | 1,19 |
| Wilson 2016 (n=151) | 151 | 0,000 | 0,000 to 2,413 | 1,61 | 1,34 |
| Yamamoto 2005 (n=175) | 175 | 1,714 | 0,355 to 4,928 | 1,86 | 1,38 |
| Yang 2010 (n=30) | 30 | 0,000 | 0,000 to 11,570 | 0,33 | 0,69 |
| Zaretsky 2018 (n=749) | 749 | 6,008 | 4,416 to 7,957 | 7,93 | 1,65 |
| Bebbington 2012 (n=146) | 146 | 0,000 | 0,000 to 2,495 | 1,55 | 1,33 |
| Berg 2014 (n=7) | 7 | 14,286 | 0,361 to 57,872 | 0,085 | 0,25 |
| Delabaere 2013 (n=30) | 30 | 0,000 | 0,000 to 11,570 | 0,33 | 0,69 |
| Deprest 2000 (n=10) | 10 | 10,000 | 0,253 to 44,502 | 0,12 | 0,33 |
| Gallot 2003 (n=11) | 11 | 0,000 | 0,000 to 28,491 | 0,13 | 0,35 |
| Gouverneur 2009 (n=54) | 54 | 0,000 | 0,000 to 6,603 | 0,58 | 0,94 |
| Gul 2008 (n=9) | 9 | 0,000 | 0,000 to 33,627 | 0,11 | 0,30 |
| Has 2014 (n=71) | 71 | 0,000 | 0,000 to 5,063 | 0,76 | 1,06 |
| He 2010 (n=14) | 14 | 0,000 | 0,000 to 23,164 | 0,16 | 0,42 |
| Ilagan 2008 (n=27) | 27 | 11,111 | 2,353 to 29,159 | 0,30 | 0,65 |
| Jelin 2010 (n=7) | 7 | 0,000 | 0,000 to 40,962 | 0,085 | 0,25 |
| King 2017 (n=43) | 43 | 9,302 | 2,593 to 22,135 | 0,47 | 0,84 |
| Lanna 2012 (n=118) | 118 | 0,000 | 0,000 to 3,078 | 1,26 | 1,25 |
| Lee 2013 (n=98) | 98 | 0,000 | 0,000 to 3,694 | 1,05 | 1,18 |
| Lewi 2006 (n=80) | 80 | 0,000 | 0,000 to 4,506 | 0,86 | 1,10 |
| Moise 2008 (n=9) | 9 | 0,000 | 0,000 to 33,627 | 0,11 | 0,30 |
| Nobili 2013 (n=48) | 48 | 0,000 | 0,000 to 7,397 | 0,52 | 0,89 |
| Paramasivam 2010 (n=35) | 35 | 0,000 | 0,000 to 10,003 | 0,38 | 0,75 |
| Peng 2016 (n=93) | 93 | 0,000 | 0,000 to 3,889 | 0,99 | 1,16 |
| Quintero 1996 (n=13) | 13 | 7,692 | 0,195 to 36,030 | 0,15 | 0,40 |
| Quintero 2006 (n=51) | 51 | 0,000 | 0,000 to 6,978 | 0,55 | 0,91 |
| Roman 2010 (n=60) | 60 | 0,000 | 0,000 to 5,963 | 0,64 | 0,98 |
| Schou 2018 (n=102) | 102 | 0,000 | 0,000 to 3,552 | 1,09 | 1,20 |
| Sugibayashi 2016 (n=40) | 40 | 0,000 | 0,000 to 8,810 | 0,43 | 0,81 |
| Takano 2015 (n=10) | 10 | 0,000 | 0,000 to 30,850 | 0,12 | 0,33 |
| Taylor 2002 (n=15) | 15 | 6,667 | 0,169 to 31,948 | 0,17 | 0,44 |
| Tsao 2002 (n=13) | 13 | 0,000 | 0,000 to 24,705 | 0,15 | 0,40 |
| Zhang 2018 (n=25) | 25 | 0,000 | 0,000 to 13,719 | 0,27 | 0,62 |
| Deprest 2005 (n=20) | 20 | 0,000 | 0,000 to 16,843 | 0,22 | 0,54 |
| Harrison 1998 (n=8) | 8 | 0,000 | 0,000 to 36,942 | 0,095 | 0,28 |
| Harrison 2003 (n=11) | 11 | 27,273 | 6,022 to 60,974 | 0,13 | 0,35 |
| Jani 2005 (n=24) | 24 | 0,000 | 0,000 to 14,247 | 0,26 | 0,60 |
| Jani 2006 (n=28) | 28 | 0,000 | 0,000 to 12,344 | 0,31 | 0,66 |
| Jani 2009 (n=210) | 210 | 0,000 | 0,000 to 1,741 | 2,23 | 1,43 |
| Jimenez 2017 (n=201) | 201 | 0,000 | 0,000 to 1,819 | 2,13 | 1,42 |
| Kosinski 2017 (n=28) | 28 | 0,000 | 0,000 to 12,344 | 0,31 | 0,66 |
| Manrique 2008 (n=11) | 11 | 0,000 | 0,000 to 28,491 | 0,13 | 0,35 |
| Peralta 2011 (n=8) | 8 | 0,000 | 0,000 to 36,942 | 0,095 | 0,28 |
| Persico 2017 (n=21) | 21 | 0,000 | 0,000 to 16,110 | 0,23 | 0,55 |
| Ruano 2012 (n=35) | 35 | 2,857 | 0,0723 to 14,917 | 0,38 | 0,75 |
| Ruano 2012 (n=20) | 20 | 0,000 | 0,000 to 16,843 | 0,22 | 0,54 |
| Ruano 2013 (n=17) | 17 | 0,000 | 0,000 to 19,506 | 0,19 | 0,48 |
| Arens 2017 (n=59) | 59 | 0,000 | 0,000 to 6,061 | 0,63 | 0,98 |
| Belfort 2017 (n=22) | 22 | 9,091 | 1,121 to 29,161 | 0,24 | 0,57 |
| Bruner 2000 (n=4) | 4 | 25,000 | 0,631 to 80,588 | 0,053 | 0,17 |
| Degenhardt 2014 (n=51) | 51 | 0,000 | 0,000 to 6,978 | 0,55 | 0,91 |
| Kohn 2018 (n=34) | 34 | 8,824 | 1,858 to 23,678 | 0,37 | 0,74 |
| Pedreira 2014 (n=4) | 4 | 0,000 | 0,000 to 60,236 | 0,053 | 0,17 |
| Pedreira 2016 (n=10) | 10 | 0,000 | 0,000 to 30,850 | 0,12 | 0,33 |
| Verbeek 2012 (n=19) | 19 | 0,000 | 0,000 to 17,647 | 0,21 | 0,52 |
| Ziemann 2018 (n=65) | 65 | 0,000 | 0,000 to 5,517 | 0,70 | 1,02 |
| Morris 2013 (n=16) | 16 | 0,000 | 0,000 to 20,591 | 0,18 | 0,46 |
| Ruano 2010 (n=11) | 11 | 0,000 | 0,000 to 28,491 | 0,13 | 0,35 |
| Welsh 2003 (n=13) | 13 | 0,000 | 0,000 to 24,705 | 0,15 | 0,40 |
| Cavalheiro 2011 (n=30) | 30 | 0,000 | 0,000 to 11,570 | 0,33 | 0,69 |
| Mallman 2017 (n=78) | 78 | 1,282 | 0,0325 to 6,937 | 0,83 | 1,09 |
| Golombeck 2006 (n=99) | 99 | 4,040 | 1,112 to 10,023 | 1,06 | 1,19 |
| Kohl 2006 (n=16) | 16 | 0,000 | 0,000 to 20,591 | 0,18 | 0,46 |
| Kohl 2010 (n=37) | 37 | 0,000 | 0,000 to 9,489 | 0,40 | 0,78 |
| Nivatpumin 2016 (n=152) | 152 | 0,000 | 0,000 to 2,398 | 1,62 | 1,34 |
| Peralta 2010 (n=56) | 56 | 0,000 | 0,000 to 6,375 | 0,60 | 0,95 |
| Total (fixed effects) | 9341 | 1,194 | 0,985 to 1,434 | 100,00 | 100,00 |
| Total (random effects) | 9341 | 1,292 | 0,903 to 1,749 | 100,00 | 100,00 |

## Test for heterogeneity

| Q | 312,4093 |
| --- | --- |
| DF | 120 |
| Significance level | P < 0,0001 |
| I^2^ (inconsistency) | 61,59% |
| 95% CI for I^2^ | 53,24 to 68,45 |

# Meta-analysis: postoperative open transfusion

| Variable for studies | Study |
| --- | --- |
| Variable for total number of cases | N |
| Variable for number of positive cases | Outcome |

| Study | Sample size | Proportion (%) | 95% CI | Weight (%) | |
| --- | --- | --- | --- | --- | --- |
|  |  |  |  | Fixed | Random |
| Barthod 2013 (n=5) | 5 | 0,000 | 0,000 to 52,182 | 0,49 | 1,14 |
| Cass 2013 (n=9) | 9 | 22,222 | 2,814 to 60,009 | 0,81 | 1,68 |
| Chen 2018 (n=7) | 7 | 0,000 | 0,000 to 40,962 | 0,65 | 1,43 |
| Dahlgren 2004 (n=4) | 4 | 0,000 | 0,000 to 60,236 | 0,40 | 0,98 |
| Flake 2000 (n=15) | 15 | 0,000 | 0,000 to 21,802 | 1,29 | 2,28 |
| George 2007 (n=3) | 3 | 0,000 | 0,000 to 70,760 | 0,32 | 0,82 |
| Hedrick 2003 (n=43) | 43 | 0,000 | 0,000 to 8,221 | 3,56 | 3,69 |
| Hedrick 2005 (n=9) | 9 | 11,111 | 0,281 to 48,250 | 0,81 | 1,68 |
| Kern 2007 (n=5) | 5 | 0,000 | 0,000 to 52,182 | 0,49 | 1,14 |
| Kornacki 2017 (n=4) | 4 | 0,000 | 0,000 to 60,236 | 0,40 | 0,98 |
| Kunisaki 2007 (n=14) | 14 | 0,000 | 0,000 to 23,164 | 1,21 | 2,20 |
| Laje 2012 (n=17) | 17 | 0,000 | 0,000 to 19,506 | 1,46 | 2,45 |
| Laje 2013 (n=4) | 4 | 0,000 | 0,000 to 60,236 | 0,40 | 0,98 |
| Laje 2015 (n=13) | 13 | 0,000 | 0,000 to 24,705 | 1,13 | 2,10 |
| Lazar 2011 (n=12) | 12 | 8,333 | 0,211 to 38,480 | 1,05 | 2,01 |
| Noah 2002 (n=34) | 34 | 8,824 | 1,858 to 23,678 | 2,83 | 3,39 |
| Pellicer 2007 (n=3) | 3 | 0,000 | 0,000 to 70,760 | 0,32 | 0,82 |
| Stoffan 2012 (n=7) | 7 | 0,000 | 0,000 to 40,962 | 0,65 | 1,43 |
| Tuncay Ozgunen 2010 (n=3) | 3 | 0,000 | 0,000 to 70,760 | 0,32 | 0,82 |
| Zamora 2013 (n=26) | 26 | 23,077 | 8,974 to 43,648 | 2,18 | 3,02 |
| Bennett 2014 (n=43) | 43 | 0,000 | 0,000 to 8,221 | 3,56 | 3,69 |
| Botelho 2017 (n=45) | 45 | 0,000 | 0,000 to 7,871 | 3,72 | 3,75 |
| Bruner 1999 (n=29) | 29 | 0,000 | 0,000 to 11,944 | 2,43 | 3,17 |
| Bruner 2000 (n=4) | 4 | 0,000 | 0,000 to 60,236 | 0,40 | 0,98 |
| Farmer 2003 (n=12) | 12 | 0,000 | 0,000 to 26,465 | 1,05 | 2,01 |
| Friszer 2016 (n=3) | 3 | 0,000 | 0,000 to 70,760 | 0,32 | 0,82 |
| Johnson 2016 (n=91) | 91 | 0,000 | 0,000 to 3,973 | 7,44 | 4,53 |
| Marenco 2013 (n=4) | 4 | 0,000 | 0,000 to 60,236 | 0,40 | 0,98 |
| Moldenhauer 2015 (n=100) | 100 | 0,000 | 0,000 to 3,622 | 8,17 | 4,61 |
| Moron 2018 (n=237) | 237 | 0,000 | 0,000 to 1,544 | 19,26 | 5,19 |
| Ochsenbein-Kolble 2017 (n=30) | 30 | 0,000 | 0,000 to 11,570 | 2,51 | 3,22 |
| Sinskey 2017 (n=47) | 47 | 0,000 | 0,000 to 7,549 | 3,88 | 3,81 |
| Soni 2016 (n=88) | 88 | 0,000 | 0,000 to 4,105 | 7,20 | 4,50 |
| Zamlynski 2014 (n=46) | 46 | 6,522 | 1,366 to 17,896 | 3,80 | 3,78 |
| Flake 2000 (n=15) | 15 | 0,000 | 0,000 to 21,802 | 1,29 | 2,28 |
| Harrison 1990 (n=6) | 6 | 0,000 | 0,000 to 45,926 | 0,57 | 1,29 |
| Harrison 1993 (n=14) | 14 | 0,000 | 0,000 to 23,164 | 1,21 | 2,20 |
| Harrison 1998 (n=13) | 13 | 0,000 | 0,000 to 24,705 | 1,13 | 2,10 |
| Adzick 2003 (n=22) | 22 | 9,091 | 1,121 to 29,161 | 1,86 | 2,79 |
| Hedrick 2004 (n=4) | 4 | 0,000 | 0,000 to 60,236 | 0,40 | 0,98 |
| Golombeck 2006 (n=79) | 79 | 13,924 | 7,161 to 23,550 | 6,47 | 4,39 |
| Longaker 1991 (n=17) | 17 | 11,765 | 1,458 to 36,441 | 1,46 | 2,45 |
| Zamora 2013 (n=7) | 7 | 14,286 | 0,361 to 57,872 | 0,65 | 1,43 |
| Total (fixed effects) | 1193 | 2,010 | 1,303 to 2,956 | 100,00 | 100,00 |
| Total (random effects) | 1193 | 3,360 | 1,853 to 5,293 | 100,00 | 100,00 |

## Test for heterogeneity

| Q | 89,8817 |
| --- | --- |
| DF | 42 |
| Significance level | P < 0,0001 |
| I^2^ (inconsistency) | 53,27% |
| 95% CI for I^2^ | 33,89 to 66,97 |

# Meta-analysis: postoperative fetoscopic transfusion

| Variable for studies | Study |
| --- | --- |
| Variable for total number of cases | N |
| Variable for number of positive cases | Outcome |

| Study | Sample size | Proportion (%) | 95% CI | Weight (%) | |
| --- | --- | --- | --- | --- | --- |
|  |  |  |  | Fixed | Random |
| Aboudiab 2017 (n=18) | 18 | 0,000 | 0,000 to 18,530 | 0,20 | 0,20 |
| Baschat 2013 (n=147) | 147 | 0,000 | 0,000 to 2,478 | 1,55 | 1,55 |
| Chalouhi 2016 (n=22) | 22 | 0,000 | 0,000 to 15,437 | 0,24 | 0,24 |
| Chang 2006 (n=27) | 27 | 0,000 | 0,000 to 12,770 | 0,29 | 0,29 |
| Chang 2016 (n=100) | 100 | 0,000 | 0,000 to 3,622 | 1,06 | 1,06 |
| Chmait 2013 (n=318) | 318 | 0,000 | 0,000 to 1,153 | 3,35 | 3,35 |
| Chmait 2017 (n=19) | 19 | 0,000 | 0,000 to 17,647 | 0,21 | 0,21 |
| Crombleholme 2007 (n=20) | 20 | 0,000 | 0,000 to 16,843 | 0,22 | 0,22 |
| De Lia 1995 (n=26) | 26 | 0,000 | 0,000 to 13,227 | 0,28 | 0,28 |
| De Lia 1999 (n=67) | 67 | 0,000 | 0,000 to 5,357 | 0,71 | 0,71 |
| De Lia 2009 (n=10) | 10 | 0,000 | 0,000 to 30,850 | 0,12 | 0,12 |
| Deprest 1998 (n=6) | 6 | 0,000 | 0,000 to 45,926 | 0,073 | 0,073 |
| Draga 2016 (n=37) | 37 | 0,000 | 0,000 to 9,489 | 0,40 | 0,40 |
| Duron 2014 (n=85) | 85 | 3,529 | 0,734 to 9,970 | 0,90 | 0,90 |
| Ek 2012 (n=) | 67 | 0,000 | 0,000 to 5,357 | 0,71 | 0,71 |
| Habli 2009 (n=152) | 152 | 0,000 | 0,000 to 2,398 | 1,61 | 1,61 |
| Has 2014 (n=85) | 85 | 0,000 | 0,000 to 4,247 | 0,90 | 0,90 |
| Hecher 2000 (n=200) | 200 | 0,000 | 0,000 to 1,828 | 2,11 | 2,11 |
| Hernandez-Andrade 2011 (n=35) | 35 | 0,000 | 0,000 to 10,003 | 0,38 | 0,38 |
| Huber 2008 (n=176) | 176 | 0,000 | 0,000 to 2,074 | 1,86 | 1,86 |
| Ishii 2014 (n=16) | 16 | 0,000 | 0,000 to 20,591 | 0,18 | 0,18 |
| Ishii 2015 (n=10) | 10 | 0,000 | 0,000 to 30,850 | 0,12 | 0,12 |
| Lanna 2017 (n=373) | 373 | 0,000 | 0,000 to 0,984 | 3,93 | 3,93 |
| Lecointre 2017 (n=200) | 200 | 0,000 | 0,000 to 1,828 | 2,11 | 2,11 |
| Malshe 2017 (n=203) | 203 | 0,000 | 0,000 to 1,801 | 2,14 | 2,14 |
| Martinez 2012 (n=500) | 500 | 0,000 | 0,000 to 0,735 | 5,26 | 5,26 |
| Middeldorp 2007 (n=100) | 100 | 0,000 | 0,000 to 3,622 | 1,06 | 1,06 |
| Miyadahira 2018 (n=67) | 67 | 0,000 | 0,000 to 5,357 | 0,71 | 0,71 |
| Molina-Garcia 2009 (n=22) | 22 | 0,000 | 0,000 to 15,437 | 0,24 | 0,24 |
| Morris 2010 (n=164) | 164 | 0,000 | 0,000 to 2,224 | 1,73 | 1,73 |
| Mullers 2015 (n=105) | 105 | 0,000 | 0,000 to 3,452 | 1,11 | 1,11 |
| Nakata 2016 (n=6) | 6 | 0,000 | 0,000 to 45,926 | 0,073 | 0,073 |
| Nguyen 2012 (n=98) | 98 | 1,020 | 0,0258 to 5,554 | 1,04 | 1,04 |
| Ozawa 2017 (n=11) | 11 | 0,000 | 0,000 to 28,491 | 0,13 | 0,13 |
| Papanna 2010 (n=48) | 48 | 0,000 | 0,000 to 7,397 | 0,51 | 0,51 |
| Papanna 2012 (n=163) | 163 | 0,000 | 0,000 to 2,238 | 1,72 | 1,72 |
| Peeters 2014 (n=338) | 338 | 0,000 | 0,000 to 1,085 | 3,56 | 3,56 |
| Persico 2016 (n=106) | 106 | 0,000 | 0,000 to 3,420 | 1,12 | 1,12 |
| Quintero 2000 (n=92) | 92 | 0,000 | 0,000 to 3,930 | 0,98 | 0,98 |
| Quintero 2001 (n=11) | 11 | 0,000 | 0,000 to 28,491 | 0,13 | 0,13 |
| Rossi 2008 (n=266) | 266 | 0,752 | 0,0912 to 2,690 | 2,80 | 2,80 |
| Ruano 2009 (n=19) | 19 | 0,000 | 0,000 to 17,647 | 0,21 | 0,21 |
| Ruegg 2018 (n=37) | 37 | 0,000 | 0,000 to 9,489 | 0,40 | 0,40 |
| Rustico 2012 (n=150) | 150 | 0,000 | 0,000 to 2,429 | 1,59 | 1,59 |
| Said 2008 (n=10) | 10 | 0,000 | 0,000 to 30,850 | 0,12 | 0,12 |
| Senat 2004 (n=72) | 72 | 0,000 | 0,000 to 4,994 | 0,77 | 0,77 |
| Sepulveda 2007 (n=33) | 33 | 0,000 | 0,000 to 10,576 | 0,36 | 0,36 |
| Shamshirsaz 2015 (n=55) | 55 | 0,000 | 0,000 to 6,487 | 0,59 | 0,59 |
| Slaghekke 2014 (n=274) | 274 | 0,000 | 0,000 to 1,337 | 2,89 | 2,89 |
| Taniguchi 2015 (n=3) | 3 | 0,000 | 0,000 to 70,760 | 0,042 | 0,042 |
| Tchirikov 2011 (n=80) | 80 | 0,000 | 0,000 to 4,506 | 0,85 | 0,85 |
| Teoh 2013 (n=49) | 49 | 0,000 | 0,000 to 7,252 | 0,52 | 0,52 |
| Thia 2017 (n=5) | 5 | 0,000 | 0,000 to 52,182 | 0,063 | 0,063 |
| Ville 1997 (n=132) | 132 | 0,758 | 0,0192 to 4,149 | 1,40 | 1,40 |
| Ville 1998 (n=44) | 44 | 0,000 | 0,000 to 8,042 | 0,47 | 0,47 |
| Weingertner 2011 (n=100) | 100 | 0,000 | 0,000 to 3,622 | 1,06 | 1,06 |
| Wilson 2016 (n=151) | 151 | 0,000 | 0,000 to 2,413 | 1,60 | 1,60 |
| Yamamoto 2005 (n=175) | 175 | 0,000 | 0,000 to 2,086 | 1,85 | 1,85 |
| Yang 2010 (n=30) | 30 | 0,000 | 0,000 to 11,570 | 0,33 | 0,33 |
| Zaretsky 2018 (n=749) | 749 | 0,000 | 0,000 to 0,491 | 7,87 | 7,87 |
| Zhao 2016 (n=62) | 62 | 0,000 | 0,000 to 5,776 | 0,66 | 0,66 |
| Bebbington 2012 (n=146) | 146 | 0,000 | 0,000 to 2,495 | 1,54 | 1,54 |
| Berg 2014 (n=7) | 7 | 0,000 | 0,000 to 40,962 | 0,084 | 0,084 |
| Delabaere 2013 (n=30) | 30 | 0,000 | 0,000 to 11,570 | 0,33 | 0,33 |
| Deprest 2000 (n=10) | 10 | 0,000 | 0,000 to 30,850 | 0,12 | 0,12 |
| Gallot 2003 (n=11) | 11 | 0,000 | 0,000 to 28,491 | 0,13 | 0,13 |
| Gouverneur 2009 (n=54) | 54 | 0,000 | 0,000 to 6,603 | 0,58 | 0,58 |
| Gul 2008 (n=9) | 9 | 0,000 | 0,000 to 33,627 | 0,10 | 0,10 |
| Has 2014 (n=71) | 71 | 0,000 | 0,000 to 5,063 | 0,76 | 0,76 |
| He 2010 (n=14) | 14 | 0,000 | 0,000 to 23,164 | 0,16 | 0,16 |
| Ilagan 2008 (n=27) | 27 | 0,000 | 0,000 to 12,770 | 0,29 | 0,29 |
| Jelin 2010 (n=7) | 7 | 0,000 | 0,000 to 40,962 | 0,084 | 0,084 |
| King 2017 (n=43) | 43 | 0,000 | 0,000 to 8,221 | 0,46 | 0,46 |
| Lanna 2012 (n=118) | 118 | 0,000 | 0,000 to 3,078 | 1,25 | 1,25 |
| Lee 2013 (n=98) | 98 | 0,000 | 0,000 to 3,694 | 1,04 | 1,04 |
| Lewi 2006 (n=80) | 80 | 0,000 | 0,000 to 4,506 | 0,85 | 0,85 |
| Moise 2008 (n=9) | 9 | 0,000 | 0,000 to 33,627 | 0,10 | 0,10 |
| Nobili 2013 (n=48) | 48 | 0,000 | 0,000 to 7,397 | 0,51 | 0,51 |
| Paramasivam 2010 (n=35) | 35 | 0,000 | 0,000 to 10,003 | 0,38 | 0,38 |
| Peng 2016 (n=93) | 93 | 0,000 | 0,000 to 3,889 | 0,99 | 0,99 |
| Quintero 1996 (n=13) | 13 | 0,000 | 0,000 to 24,705 | 0,15 | 0,15 |
| Quintero 2006 (n=51) | 51 | 0,000 | 0,000 to 6,978 | 0,55 | 0,55 |
| Roman 2010 (n=60) | 60 | 0,000 | 0,000 to 5,963 | 0,64 | 0,64 |
| Schou 2018 (n=102) | 102 | 0,000 | 0,000 to 3,552 | 1,08 | 1,08 |
| Sugibayashi 2016 (n=40) | 40 | 0,000 | 0,000 to 8,810 | 0,43 | 0,43 |
| Takano 2015 (n=10) | 10 | 0,000 | 0,000 to 30,850 | 0,12 | 0,12 |
| Taylor 2002 (n=15) | 15 | 0,000 | 0,000 to 21,802 | 0,17 | 0,17 |
| Tsao 2002 (n=13) | 13 | 0,000 | 0,000 to 24,705 | 0,15 | 0,15 |
| Zhang 2018 (n=25) | 25 | 0,000 | 0,000 to 13,719 | 0,27 | 0,27 |
| Deprest 2005 (n=20) | 20 | 0,000 | 0,000 to 16,843 | 0,22 | 0,22 |
| Harrison 1998 (n=8) | 8 | 0,000 | 0,000 to 36,942 | 0,094 | 0,094 |
| Harrison 2003 (n=11) | 11 | 0,000 | 0,000 to 28,491 | 0,13 | 0,13 |
| Jani 2005 (n=24) | 24 | 0,000 | 0,000 to 14,247 | 0,26 | 0,26 |
| Jani 2006 (n=28) | 28 | 0,000 | 0,000 to 12,344 | 0,30 | 0,30 |
| Jani 2009 (n=210) | 210 | 0,000 | 0,000 to 1,741 | 2,22 | 2,22 |
| Jimenez 2017 (n=201) | 201 | 0,000 | 0,000 to 1,819 | 2,12 | 2,12 |
| Kosinski 2017 (n=28) | 28 | 0,000 | 0,000 to 12,344 | 0,30 | 0,30 |
| Manrique 2008 (n=11) | 11 | 0,000 | 0,000 to 28,491 | 0,13 | 0,13 |
| Peralta 2011 (n=8) | 8 | 0,000 | 0,000 to 36,942 | 0,094 | 0,094 |
| Persico 2017 (n=21) | 21 | 0,000 | 0,000 to 16,110 | 0,23 | 0,23 |
| Ruano 2012 (n=35) | 35 | 0,000 | 0,000 to 10,003 | 0,38 | 0,38 |
| Ruano 2012 (n=20) | 20 | 0,000 | 0,000 to 16,843 | 0,22 | 0,22 |
| Ruano 2013 (n=17) | 17 | 0,000 | 0,000 to 19,506 | 0,19 | 0,19 |
| Arens 2017 (n=59) | 59 | 0,000 | 0,000 to 6,061 | 0,63 | 0,63 |
| Belfort 2017 (n=22) | 22 | 0,000 | 0,000 to 15,437 | 0,24 | 0,24 |
| Bruner 2000 (n=4) | 4 | 0,000 | 0,000 to 60,236 | 0,052 | 0,052 |
| Degenhardt 2014 (n=51) | 51 | 0,000 | 0,000 to 6,978 | 0,55 | 0,55 |
| Kohn 2018 (n=34) | 34 | 0,000 | 0,000 to 10,282 | 0,37 | 0,37 |
| Pedreira 2014 (n=4) | 4 | 0,000 | 0,000 to 60,236 | 0,052 | 0,052 |
| Pedreira 2016 (n=10) | 10 | 0,000 | 0,000 to 30,850 | 0,12 | 0,12 |
| Verbeek 2012 (n=19) | 19 | 0,000 | 0,000 to 17,647 | 0,21 | 0,21 |
| Ziemann 2018 (n=65) | 65 | 0,000 | 0,000 to 5,517 | 0,69 | 0,69 |
| Morris 2013 (n=16) | 16 | 0,000 | 0,000 to 20,591 | 0,18 | 0,18 |
| Ruano 2010 (n=11) | 11 | 0,000 | 0,000 to 28,491 | 0,13 | 0,13 |
| Welsh 2003 (n=13) | 13 | 0,000 | 0,000 to 24,705 | 0,15 | 0,15 |
| Cavalheiro 2011 (n=30) | 30 | 0,000 | 0,000 to 11,570 | 0,33 | 0,33 |
| Mallman 2017 (n=78) | 78 | 0,000 | 0,000 to 4,619 | 0,83 | 0,83 |
| Golombeck 2006 (n=99) | 99 | 2,020 | 0,246 to 7,108 | 1,05 | 1,05 |
| Kohl 2006 (n=16) | 16 | 6,250 | 0,158 to 30,232 | 0,18 | 0,18 |
| Kohl 2010 (n=37) | 37 | 0,000 | 0,000 to 9,489 | 0,40 | 0,40 |
| Nivatpumin 2016 (n=152) | 152 | 1,316 | 0,160 to 4,672 | 1,61 | 1,61 |
| Peralta 2010 (n=56) | 56 | 0,000 | 0,000 to 6,375 | 0,60 | 0,60 |
| Total (fixed effects) | 9403 | 0,318 | 0,215 to 0,453 | 100,00 | 100,00 |
| Total (random effects) | 9403 | 0,318 | 0,215 to 0,441 | 100,00 | 100,00 |

## Test for heterogeneity

| Q | 57,2613 |
| --- | --- |
| DF | 121 |
| Significance level | P = 1,0000 |
| I^2^ (inconsistency) | 0,00% |
| 95% CI for I^2^ | 0,00 to 0,00 |

# Meta-analysis: postoperative open chorioamnionitis

| Variable for studies | Study |
| --- | --- |
| Variable for total number of cases | N |
| Variable for number of positive cases | Outcome |

| Study | Sample size | Proportion (%) | 95% CI | Weight (%) | |
| --- | --- | --- | --- | --- | --- |
|  |  |  |  | Fixed | Random |
| Barthod 2013 (n=5) | 5 | 0,000 | 0,000 to 52,182 | 0,49 | 0,55 |
| Cass 2013 (n=9) | 9 | 0,000 | 0,000 to 33,627 | 0,81 | 0,91 |
| Chen 2018 (n=7) | 7 | 0,000 | 0,000 to 40,962 | 0,65 | 0,73 |
| Dahlgren 2004 (n=4) | 4 | 0,000 | 0,000 to 60,236 | 0,40 | 0,46 |
| Flake 2000 (n=15) | 15 | 0,000 | 0,000 to 21,802 | 1,29 | 1,43 |
| George 2007 (n=3) | 3 | 0,000 | 0,000 to 70,760 | 0,32 | 0,37 |
| Hedrick 2003 (n=43) | 43 | 4,651 | 0,568 to 15,811 | 3,56 | 3,76 |
| Hedrick 2005 (n=9) | 9 | 11,111 | 0,281 to 48,250 | 0,81 | 0,91 |
| Kern 2007 (n=5) | 5 | 0,000 | 0,000 to 52,182 | 0,49 | 0,55 |
| Kornacki 2017 (n=4) | 4 | 0,000 | 0,000 to 60,236 | 0,40 | 0,46 |
| Kunisaki 2007 (n=14) | 14 | 0,000 | 0,000 to 23,164 | 1,21 | 1,35 |
| Laje 2012 (n=17) | 17 | 0,000 | 0,000 to 19,506 | 1,46 | 1,61 |
| Laje 2013 (n=4) | 4 | 0,000 | 0,000 to 60,236 | 0,40 | 0,46 |
| Laje 2015 (n=13) | 13 | 0,000 | 0,000 to 24,705 | 1,13 | 1,26 |
| Lazar 2011 (n=12) | 12 | 8,333 | 0,211 to 38,480 | 1,05 | 1,17 |
| Noah 2002 (n=34) | 34 | 14,706 | 4,953 to 31,057 | 2,83 | 3,04 |
| Pellicer 2007 (n=3) | 3 | 0,000 | 0,000 to 70,760 | 0,32 | 0,37 |
| Stoffan 2012 (n=7) | 7 | 0,000 | 0,000 to 40,962 | 0,65 | 0,73 |
| Tuncay Ozgunen 2010 (n=3) | 3 | 0,000 | 0,000 to 70,760 | 0,32 | 0,37 |
| Zamora 2013 (n=26) | 26 | 3,846 | 0,0973 to 19,637 | 2,18 | 2,37 |
| Bennett 2014 (n=43) | 43 | 0,000 | 0,000 to 8,221 | 3,56 | 3,76 |
| Botelho 2017 (n=45) | 45 | 0,000 | 0,000 to 7,871 | 3,72 | 3,92 |
| Bruner 1999 (n=29) | 29 | 0,000 | 0,000 to 11,944 | 2,43 | 2,62 |
| Bruner 2000 (n=4) | 4 | 0,000 | 0,000 to 60,236 | 0,40 | 0,46 |
| Farmer 2003 (n=12) | 12 | 16,667 | 2,086 to 48,414 | 1,05 | 1,17 |
| Friszer 2016 (n=3) | 3 | 0,000 | 0,000 to 70,760 | 0,32 | 0,37 |
| Johnson 2016 (n=91) | 91 | 2,198 | 0,267 to 7,715 | 7,44 | 7,29 |
| Marenco 2013 (n=4) | 4 | 0,000 | 0,000 to 60,236 | 0,40 | 0,46 |
| Moldenhauer 2015 (n=100) | 100 | 4,000 | 1,100 to 9,926 | 8,17 | 7,89 |
| Moron 2018 (n=237) | 237 | 2,954 | 1,196 to 5,991 | 19,26 | 15,43 |
| Ochsenbein-Kolble 2017 (n=30) | 30 | 0,000 | 0,000 to 11,570 | 2,51 | 2,71 |
| Sinskey 2017 (n=47) | 47 | 0,000 | 0,000 to 7,549 | 3,88 | 4,08 |
| Soni 2016 (n=88) | 88 | 4,545 | 1,252 to 11,231 | 7,20 | 7,08 |
| Zamlynski 2014 (n=46) | 46 | 4,348 | 0,531 to 14,839 | 3,80 | 4,00 |
| Flake 2000 (n=15) | 15 | 13,333 | 1,658 to 40,460 | 1,29 | 1,43 |
| Harrison 1990 (n=6) | 6 | 16,667 | 0,421 to 64,123 | 0,57 | 0,64 |
| Harrison 1993 (n=14) | 14 | 0,000 | 0,000 to 23,164 | 1,21 | 1,35 |
| Harrison 1998 (n=13) | 13 | 0,000 | 0,000 to 24,705 | 1,13 | 1,26 |
| Adzick 2003 (n=22) | 22 | 4,545 | 0,115 to 22,844 | 1,86 | 2,04 |
| Hedrick 2004 (n=4) | 4 | 50,000 | 6,759 to 93,241 | 0,40 | 0,46 |
| Golombeck 2006 (n=79) | 79 | 8,861 | 3,637 to 17,408 | 6,47 | 6,46 |
| Longaker 1991 (n=17) | 17 | 5,882 | 0,149 to 28,689 | 1,46 | 1,61 |
| Zamora 2013 (n=7) | 7 | 0,000 | 0,000 to 40,962 | 0,65 | 0,73 |
| Total (fixed effects) | 1193 | 4,075 | 3,043 to 5,332 | 100,00 | 100,00 |
| Total (random effects) | 1193 | 4,133 | 3,031 to 5,398 | 100,00 | 100,00 |

## Test for heterogeneity

| Q | 44,0350 |
| --- | --- |
| DF | 42 |
| Significance level | P = 0,3855 |
| I^2^ (inconsistency) | 4,62% |
| 95% CI for I^2^ | 0,00 to 32,00 |

# Meta-analysis: postoperative fetoscopic chorioamnionitis

| Variable for studies | Study |
| --- | --- |
| Variable for total number of cases | N |
| Variable for number of positive cases | Outcome |

| Study | Sample size | Proportion (%) | 95% CI | Weight (%) | |
| --- | --- | --- | --- | --- | --- |
|  |  |  |  | Fixed | Random |
| Aboudiab 2017 (n=18) | 18 | 0,000 | 0,000 to 18,530 | 0,20 | 0,46 |
| Baschat 2013 (n=147) | 147 | 4,762 | 1,936 to 9,565 | 1,55 | 1,36 |
| Chalouhi 2016 (n=22) | 22 | 4,545 | 0,115 to 22,844 | 0,24 | 0,53 |
| Chang 2006 (n=27) | 27 | 0,000 | 0,000 to 12,770 | 0,29 | 0,61 |
| Chang 2016 (n=100) | 100 | 0,000 | 0,000 to 3,622 | 1,06 | 1,20 |
| Chmait 2013 (n=318) | 318 | 0,943 | 0,195 to 2,732 | 3,35 | 1,60 |
| Chmait 2017 (n=19) | 19 | 10,526 | 1,301 to 33,138 | 0,21 | 0,48 |
| Crombleholme 2007 (n=20) | 20 | 0,000 | 0,000 to 16,843 | 0,22 | 0,50 |
| De Lia 1995 (n=26) | 26 | 0,000 | 0,000 to 13,227 | 0,28 | 0,60 |
| De Lia 1999 (n=67) | 67 | 0,000 | 0,000 to 5,357 | 0,71 | 1,02 |
| De Lia 2009 (n=10) | 10 | 0,000 | 0,000 to 30,850 | 0,12 | 0,30 |
| Deprest 1998 (n=6) | 6 | 0,000 | 0,000 to 45,926 | 0,073 | 0,20 |
| Draga 2016 (n=37) | 37 | 2,703 | 0,0684 to 14,160 | 0,40 | 0,75 |
| Duron 2014 (n=85) | 85 | 0,000 | 0,000 to 4,247 | 0,90 | 1,13 |
| Ek 2012 (n=) | 67 | 0,000 | 0,000 to 5,357 | 0,71 | 1,02 |
| Habli 2009 (n=152) | 152 | 1,316 | 0,160 to 4,672 | 1,61 | 1,37 |
| Has 2014 (n=85) | 85 | 0,000 | 0,000 to 4,247 | 0,90 | 1,13 |
| Hecher 2000 (n=200) | 200 | 0,000 | 0,000 to 1,828 | 2,11 | 1,47 |
| Hernandez-Andrade 2011 (n=35) | 35 | 0,000 | 0,000 to 10,003 | 0,38 | 0,72 |
| Huber 2008 (n=176) | 176 | 0,000 | 0,000 to 2,074 | 1,86 | 1,43 |
| Ishii 2014 (n=16) | 16 | 0,000 | 0,000 to 20,591 | 0,18 | 0,43 |
| Ishii 2015 (n=10) | 10 | 0,000 | 0,000 to 30,850 | 0,12 | 0,30 |
| Lanna 2017 (n=373) | 373 | 0,000 | 0,000 to 0,984 | 3,93 | 1,64 |
| Lecointre 2017 (n=200) | 200 | 0,000 | 0,000 to 1,828 | 2,11 | 1,47 |
| Malshe 2017 (n=203) | 203 | 6,404 | 3,454 to 10,702 | 2,14 | 1,47 |
| Martinez 2012 (n=500) | 500 | 0,000 | 0,000 to 0,735 | 5,26 | 1,70 |
| Middeldorp 2007 (n=100) | 100 | 2,000 | 0,243 to 7,038 | 1,06 | 1,20 |
| Miyadahira 2018 (n=67) | 67 | 0,000 | 0,000 to 5,357 | 0,71 | 1,02 |
| Molina-Garcia 2009 (n=22) | 22 | 0,000 | 0,000 to 15,437 | 0,24 | 0,53 |
| Morris 2010 (n=164) | 164 | 0,000 | 0,000 to 2,224 | 1,73 | 1,40 |
| Mullers 2015 (n=105) | 105 | 0,952 | 0,0241 to 5,192 | 1,11 | 1,22 |
| Nakata 2016 (n=6) | 6 | 0,000 | 0,000 to 45,926 | 0,073 | 0,20 |
| Nguyen 2012 (n=98) | 98 | 0,000 | 0,000 to 3,694 | 1,04 | 1,19 |
| Ozawa 2017 (n=11) | 11 | 0,000 | 0,000 to 28,491 | 0,13 | 0,32 |
| Papanna 2010 (n=48) | 48 | 0,000 | 0,000 to 7,397 | 0,51 | 0,86 |
| Papanna 2012 (n=163) | 163 | 3,067 | 1,003 to 7,013 | 1,72 | 1,40 |
| Peeters 2014 (n=338) | 338 | 0,000 | 0,000 to 1,085 | 3,56 | 1,62 |
| Persico 2016 (n=106) | 106 | 0,000 | 0,000 to 3,420 | 1,12 | 1,23 |
| Quintero 2000 (n=92) | 92 | 2,174 | 0,264 to 7,633 | 0,98 | 1,16 |
| Quintero 2001 (n=11) | 11 | 0,000 | 0,000 to 28,491 | 0,13 | 0,32 |
| Rossi 2008 (n=266) | 266 | 0,000 | 0,000 to 1,377 | 2,80 | 1,56 |
| Ruano 2009 (n=19) | 19 | 0,000 | 0,000 to 17,647 | 0,21 | 0,48 |
| Ruegg 2018 (n=37) | 37 | 16,216 | 6,193 to 32,014 | 0,40 | 0,75 |
| Rustico 2012 (n=150) | 150 | 3,333 | 1,091 to 7,607 | 1,59 | 1,37 |
| Said 2008 (n=10) | 10 | 0,000 | 0,000 to 30,850 | 0,12 | 0,30 |
| Senat 2004 (n=72) | 72 | 0,000 | 0,000 to 4,994 | 0,77 | 1,05 |
| Sepulveda 2007 (n=33) | 33 | 0,000 | 0,000 to 10,576 | 0,36 | 0,70 |
| Shamshirsaz 2015 (n=55) | 55 | 5,455 | 1,139 to 15,123 | 0,59 | 0,93 |
| Slaghekke 2014 (n=274) | 274 | 0,365 | 0,00924 to 2,017 | 2,89 | 1,56 |
| Taniguchi 2015 (n=3) | 3 | 0,000 | 0,000 to 70,760 | 0,042 | 0,12 |
| Tchirikov 2011 (n=80) | 80 | 0,000 | 0,000 to 4,506 | 0,85 | 1,10 |
| Teoh 2013 (n=49) | 49 | 0,000 | 0,000 to 7,252 | 0,52 | 0,87 |
| Thia 2017 (n=5) | 5 | 0,000 | 0,000 to 52,182 | 0,063 | 0,18 |
| Ville 1997 (n=132) | 132 | 0,000 | 0,000 to 2,756 | 1,40 | 1,32 |
| Ville 1998 (n=44) | 44 | 4,545 | 0,555 to 15,473 | 0,47 | 0,82 |
| Weingertner 2011 (n=100) | 100 | 0,000 | 0,000 to 3,622 | 1,06 | 1,20 |
| Wilson 2016 (n=151) | 151 | 0,000 | 0,000 to 2,413 | 1,60 | 1,37 |
| Yamamoto 2005 (n=175) | 175 | 2,286 | 0,626 to 5,749 | 1,85 | 1,42 |
| Yang 2010 (n=30) | 30 | 0,000 | 0,000 to 11,570 | 0,33 | 0,66 |
| Zaretsky 2018 (n=749) | 749 | 0,000 | 0,000 to 0,491 | 7,87 | 1,76 |
| Zhao 2016 (n=62) | 62 | 12,903 | 5,739 to 23,851 | 0,66 | 0,98 |
| Bebbington 2012 (n=146) | 146 | 0,000 | 0,000 to 2,495 | 1,54 | 1,36 |
| Berg 2014 (n=7) | 7 | 14,286 | 0,361 to 57,872 | 0,084 | 0,23 |
| Delabaere 2013 (n=30) | 30 | 0,000 | 0,000 to 11,570 | 0,33 | 0,66 |
| Deprest 2000 (n=10) | 10 | 0,000 | 0,000 to 30,850 | 0,12 | 0,30 |
| Gallot 2003 (n=11) | 11 | 0,000 | 0,000 to 28,491 | 0,13 | 0,32 |
| Gouverneur 2009 (n=54) | 54 | 0,000 | 0,000 to 6,603 | 0,58 | 0,92 |
| Gul 2008 (n=9) | 9 | 0,000 | 0,000 to 33,627 | 0,10 | 0,28 |
| Has 2014 (n=71) | 71 | 1,408 | 0,0357 to 7,599 | 0,76 | 1,05 |
| He 2010 (n=14) | 14 | 7,143 | 0,181 to 33,868 | 0,16 | 0,39 |
| Ilagan 2008 (n=27) | 27 | 7,407 | 0,910 to 24,290 | 0,29 | 0,61 |
| Jelin 2010 (n=7) | 7 | 0,000 | 0,000 to 40,962 | 0,084 | 0,23 |
| King 2017 (n=43) | 43 | 2,326 | 0,0589 to 12,289 | 0,46 | 0,81 |
| Lanna 2012 (n=118) | 118 | 0,847 | 0,0215 to 4,631 | 1,25 | 1,27 |
| Lee 2013 (n=98) | 98 | 0,000 | 0,000 to 3,694 | 1,04 | 1,19 |
| Lewi 2006 (n=80) | 80 | 1,250 | 0,0316 to 6,769 | 0,85 | 1,10 |
| Moise 2008 (n=9) | 9 | 0,000 | 0,000 to 33,627 | 0,10 | 0,28 |
| Nobili 2013 (n=48) | 48 | 0,000 | 0,000 to 7,397 | 0,51 | 0,86 |
| Paramasivam 2010 (n=35) | 35 | 0,000 | 0,000 to 10,003 | 0,38 | 0,72 |
| Peng 2016 (n=93) | 93 | 10,753 | 5,278 to 18,887 | 0,99 | 1,17 |
| Quintero 1996 (n=13) | 13 | 0,000 | 0,000 to 24,705 | 0,15 | 0,37 |
| Quintero 2006 (n=51) | 51 | 0,000 | 0,000 to 6,978 | 0,55 | 0,89 |
| Roman 2010 (n=60) | 60 | 0,000 | 0,000 to 5,963 | 0,64 | 0,97 |
| Schou 2018 (n=102) | 102 | 0,980 | 0,0248 to 5,342 | 1,08 | 1,21 |
| Sugibayashi 2016 (n=40) | 40 | 0,000 | 0,000 to 8,810 | 0,43 | 0,78 |
| Takano 2015 (n=10) | 10 | 0,000 | 0,000 to 30,850 | 0,12 | 0,30 |
| Taylor 2002 (n=15) | 15 | 0,000 | 0,000 to 21,802 | 0,17 | 0,41 |
| Tsao 2002 (n=13) | 13 | 0,000 | 0,000 to 24,705 | 0,15 | 0,37 |
| Zhang 2018 (n=25) | 25 | 0,000 | 0,000 to 13,719 | 0,27 | 0,58 |
| Deprest 2005 (n=20) | 20 | 0,000 | 0,000 to 16,843 | 0,22 | 0,50 |
| Harrison 1998 (n=8) | 8 | 0,000 | 0,000 to 36,942 | 0,094 | 0,25 |
| Harrison 2003 (n=11) | 11 | 0,000 | 0,000 to 28,491 | 0,13 | 0,32 |
| Jani 2005 (n=24) | 24 | 0,000 | 0,000 to 14,247 | 0,26 | 0,57 |
| Jani 2006 (n=28) | 28 | 0,000 | 0,000 to 12,344 | 0,30 | 0,63 |
| Jani 2009 (n=210) | 210 | 2,381 | 0,778 to 5,469 | 2,22 | 1,49 |
| Jimenez 2017 (n=201) | 201 | 0,000 | 0,000 to 1,819 | 2,12 | 1,47 |
| Kosinski 2017 (n=28) | 28 | 0,000 | 0,000 to 12,344 | 0,30 | 0,63 |
| Manrique 2008 (n=11) | 11 | 0,000 | 0,000 to 28,491 | 0,13 | 0,32 |
| Peralta 2011 (n=8) | 8 | 0,000 | 0,000 to 36,942 | 0,094 | 0,25 |
| Persico 2017 (n=21) | 21 | 0,000 | 0,000 to 16,110 | 0,23 | 0,52 |
| Ruano 2012 (n=35) | 35 | 0,000 | 0,000 to 10,003 | 0,38 | 0,72 |
| Ruano 2012 (n=20) | 20 | 5,000 | 0,127 to 24,873 | 0,22 | 0,50 |
| Ruano 2013 (n=17) | 17 | 5,882 | 0,149 to 28,689 | 0,19 | 0,45 |
| Arens 2017 (n=59) | 59 | 1,695 | 0,0429 to 9,086 | 0,63 | 0,96 |
| Belfort 2017 (n=22) | 22 | 0,000 | 0,000 to 15,437 | 0,24 | 0,53 |
| Bruner 2000 (n=4) | 4 | 0,000 | 0,000 to 60,236 | 0,052 | 0,15 |
| Degenhardt 2014 (n=51) | 51 | 5,882 | 1,230 to 16,242 | 0,55 | 0,89 |
| Kohn 2018 (n=34) | 34 | 2,941 | 0,0744 to 15,327 | 0,37 | 0,71 |
| Pedreira 2014 (n=4) | 4 | 50,000 | 6,759 to 93,241 | 0,052 | 0,15 |
| Pedreira 2016 (n=10) | 10 | 0,000 | 0,000 to 30,850 | 0,12 | 0,30 |
| Verbeek 2012 (n=19) | 19 | 0,000 | 0,000 to 17,647 | 0,21 | 0,48 |
| Ziemann 2018 (n=65) | 65 | 4,615 | 0,962 to 12,901 | 0,69 | 1,00 |
| Morris 2013 (n=16) | 16 | 18,750 | 4,047 to 45,646 | 0,18 | 0,43 |
| Ruano 2010 (n=11) | 11 | 0,000 | 0,000 to 28,491 | 0,13 | 0,32 |
| Welsh 2003 (n=13) | 13 | 7,692 | 0,195 to 36,030 | 0,15 | 0,37 |
| Cavalheiro 2011 (n=30) | 30 | 0,000 | 0,000 to 11,570 | 0,33 | 0,66 |
| Mallman 2017 (n=78) | 78 | 7,692 | 2,875 to 15,995 | 0,83 | 1,09 |
| Golombeck 2006 (n=99) | 99 | 1,010 | 0,0256 to 5,500 | 1,05 | 1,20 |
| Kohl 2006 (n=16) | 16 | 0,000 | 0,000 to 20,591 | 0,18 | 0,43 |
| Kohl 2010 (n=37) | 37 | 0,000 | 0,000 to 9,489 | 0,40 | 0,75 |
| Nivatpumin 2016 (n=152) | 152 | 0,000 | 0,000 to 2,398 | 1,61 | 1,37 |
| Peralta 2010 (n=56) | 56 | 0,000 | 0,000 to 6,375 | 0,60 | 0,94 |
| Total (fixed effects) | 9403 | 0,927 | 0,745 to 1,141 | 100,00 | 100,00 |
| Total (random effects) | 9403 | 1,454 | 1,063 to 1,906 | 100,00 | 100,00 |

## Test for heterogeneity

| Q | 279,5173 |
| --- | --- |
| DF | 121 |
| Significance level | P < 0,0001 |
| I^2^ (inconsistency) | 56,71% |
| 95% CI for I^2^ | 47,02 to 64,63 |

# Meta-analysis: postoperative open chorioamnionitis PPROM

| Variable for studies | Study |
| --- | --- |
| Variable for total number of cases | N |
| Variable for number of positive cases | Outcome |

| Study | Sample size | Proportion (%) | 95% CI | Weight (%) | |
| --- | --- | --- | --- | --- | --- |
|  |  |  |  | Fixed | Random |
| Hedrick 2003 (n=43) | 2 | 0,000 | 0,000 to 84,189 | 4,84 | 5,85 |
| Hedrick 2005 (n=9) | 1 | 100,000 | 2,500 to 100,000 | 3,23 | 5,15 |
| Lazar 2011 (n=12) | 1 | 0,000 | 0,000 to 97,500 | 3,23 | 5,15 |
| Noah 2002 (n=34) | 5 | 0,000 | 0,000 to 52,182 | 9,68 | 6,77 |
| Zamora 2013 (n=26) | 1 | 0,000 | 0,000 to 97,500 | 3,23 | 5,15 |
| Farmer 2003 (n=12) | 2 | 100,000 | 15,811 to 100,000 | 4,84 | 5,85 |
| Johnson 2016 (n=91) | 2 | 0,000 | 0,000 to 84,189 | 4,84 | 5,85 |
| Moldenhauer 2015 (n=100) | 4 | 100,000 | 39,764 to 100,000 | 8,06 | 6,56 |
| Moron 2018 (n=237) | 7 | 100,000 | 59,038 to 100,000 | 12,90 | 7,04 |
| Soni 2016 (n=88) | 4 | 100,000 | 39,764 to 100,000 | 8,06 | 6,56 |
| Zamlynski 2014 (n=46) | 2 | 0,000 | 0,000 to 84,189 | 4,84 | 5,85 |
| Flake 2000 (n=15) | 2 | 0,000 | 0,000 to 84,189 | 4,84 | 5,85 |
| Harrison 1990 (n=6) | 1 | 100,000 | 2,500 to 100,000 | 3,23 | 5,15 |
| Adzick 2003 (n=22) | 1 | 0,000 | 0,000 to 97,500 | 3,23 | 5,15 |
| Hedrick 2004 (n=4) | 2 | 100,000 | 15,811 to 100,000 | 4,84 | 5,85 |
| Golombeck 2006 (n=79) | 7 | 0,000 | 0,000 to 40,962 | 12,90 | 7,04 |
| Longaker 1991 (n=17) | 1 | 100,000 | 2,500 to 100,000 | 3,23 | 5,15 |
| Total (fixed effects) | 45 | 48,740 | 35,831 to 61,774 | 100,00 | 100,00 |
| Total (random effects) | 45 | 47,784 | 23,010 to 73,157 | 100,00 | 100,00 |

## Test for heterogeneity

| Q | 67,3901 |
| --- | --- |
| DF | 16 |
| Significance level | P < 0,0001 |
| I^2^ (inconsistency) | 76,26% |
| 95% CI for I^2^ | 62,15 to 85,11 |

# Meta-analysis: postoperative fetoscopic chorioamnionitis PPROM

| Variable for studies | Study |
| --- | --- |
| Variable for total number of cases | Outcome_1 |
| Variable for number of positive cases | Outcome_2 |

| Study | Sample size | Proportion (%) | 95% CI | Weight (%) | |
| --- | --- | --- | --- | --- | --- |
|  |  |  |  | Fixed | Random |
| Baschat 2013 (n=147) | 7 | 0,000 | 0,000 to 40,962 | 5,19 | 3,18 |
| Chalouhi 2016 (n=22) | 1 | 0,000 | 0,000 to 97,500 | 1,30 | 2,18 |
| Chmait 2013 (n=318) | 3 | 0,000 | 0,000 to 70,760 | 2,60 | 2,76 |
| Chmait 2017 (n=19) | 2 | 100,000 | 15,811 to 100,000 | 1,95 | 2,54 |
| Draga 2016 (n=37) | 1 | 100,000 | 2,500 to 100,000 | 1,30 | 2,18 |
| Habli 2009 (n=152) | 2 | 0,000 | 0,000 to 84,189 | 1,95 | 2,54 |
| Malshe 2017 (n=203) | 13 | 0,000 | 0,000 to 24,705 | 9,09 | 3,40 |
| Middeldorp 2007 (n=100) | 2 | 100,000 | 15,811 to 100,000 | 1,95 | 2,54 |
| Mullers 2015 (n=105) | 1 | 0,000 | 0,000 to 97,500 | 1,30 | 2,18 |
| Papanna 2012 (n=163) | 5 | 0,000 | 0,000 to 52,182 | 3,90 | 3,02 |
| Quintero 2000 (n=92) | 2 | 0,000 | 0,000 to 84,189 | 1,95 | 2,54 |
| Ruegg 2018 (n=37) | 6 | 0,000 | 0,000 to 45,926 | 4,55 | 3,11 |
| Rustico 2012 (n=150) | 5 | 100,000 | 47,818 to 100,000 | 3,90 | 3,02 |
| Shamshirsaz 2015 (n=55) | 3 | 0,000 | 0,000 to 70,760 | 2,60 | 2,76 |
| Slaghekke 2014 (n=274) | 1 | 0,000 | 0,000 to 97,500 | 1,30 | 2,18 |
| Ville 1998 (n=44) | 2 | 0,000 | 0,000 to 84,189 | 1,95 | 2,54 |
| Yamamoto 2005 (n=175) | 4 | 100,000 | 39,764 to 100,000 | 3,25 | 2,91 |
| Zhao 2016 (n=62) | 8 | 0,000 | 0,000 to 36,942 | 5,84 | 3,23 |
| Berg 2014 (n=7) | 1 | 0,000 | 0,000 to 97,500 | 1,30 | 2,18 |
| Has 2014 (n=71) | 1 | 0,000 | 0,000 to 97,500 | 1,30 | 2,18 |
| He 2010 (n=14) | 1 | 100,000 | 2,500 to 100,000 | 1,30 | 2,18 |
| Ilagan 2008 (n=27) | 2 | 0,000 | 0,000 to 84,189 | 1,95 | 2,54 |
| King 2017 (n=43) | 1 | 100,000 | 2,500 to 100,000 | 1,30 | 2,18 |
| Lanna 2012 (n=118) | 1 | 100,000 | 2,500 to 100,000 | 1,30 | 2,18 |
| Lewi 2006 (n=80) | 1 | 0,000 | 0,000 to 97,500 | 1,30 | 2,18 |
| Peng 2016 (n=93) | 10 | 0,000 | 0,000 to 30,850 | 7,14 | 3,31 |
| Schou 2018 (n=102) | 1 | 100,000 | 2,500 to 100,000 | 1,30 | 2,18 |
| Jani 2009 (n=210) | 5 | 100,000 | 47,818 to 100,000 | 3,90 | 3,02 |
| Ruano 2012 (n=20) | 1 | 100,000 | 2,500 to 100,000 | 1,30 | 2,18 |
| Ruano 2013 (n=17) | 1 | 0,000 | 0,000 to 97,500 | 1,30 | 2,18 |
| Arens 2017 (n=59) | 1 | 0,000 | 0,000 to 97,500 | 1,30 | 2,18 |
| Degenhardt 2014 (n=51) | 3 | 33,333 | 0,840 to 90,570 | 2,60 | 2,76 |
| Kohn 2018 (n=34) | 1 | 100,000 | 2,500 to 100,000 | 1,30 | 2,18 |
| Pedreira 2014 (n=4) | 2 | 100,000 | 15,811 to 100,000 | 1,95 | 2,54 |
| Verbeek 2012 (n=19) | 3 | 0,000 | 0,000 to 70,760 | 2,60 | 2,76 |
| Morris 2013 (n=16) | 3 | 100,000 | 29,240 to 100,000 | 2,60 | 2,76 |
| Welsh 2003 (n=13) | 1 | 100,000 | 2,500 to 100,000 | 1,30 | 2,18 |
| Mallman 2017 (n=78) | 6 | 0,000 | 0,000 to 45,926 | 4,55 | 3,11 |
| Golombeck 2006 (n=99) | 1 | 0,000 | 0,000 to 97,500 | 1,30 | 2,18 |
| Total (fixed effects) | 115 | 27,798 | 20,889 to 35,582 | 100,00 | 100,00 |
| Total (random effects) | 115 | 36,313 | 22,000 to 51,991 | 100,00 | 100,00 |

## Test for heterogeneity

| Q | 141,4983 |
| --- | --- |
| DF | 38 |
| Significance level | P < 0,0001 |
| I^2^ (inconsistency) | 73,14% |
| 95% CI for I^2^ | 63,18 to 80,41 |

# Meta-analysis: postoperative open pulmonary oedema

| Variable for studies | Study |
| --- | --- |
| Variable for total number of cases | N |
| Variable for number of positive cases | Outcome |

| Study | Sample size | Proportion (%) | 95% CI | Weight (%) | |
| --- | --- | --- | --- | --- | --- |
|  |  |  |  | Fixed | Random |
| Barthod 2013 (n=5) | 5 | 0,000 | 0,000 to 52,182 | 0,49 | 1,37 |
| Cass 2013 (n=9) | 9 | 0,000 | 0,000 to 33,627 | 0,81 | 1,90 |
| Chen 2018 (n=7) | 7 | 0,000 | 0,000 to 40,962 | 0,65 | 1,66 |
| Dahlgren 2004 (n=4) | 4 | 0,000 | 0,000 to 60,236 | 0,40 | 1,21 |
| Flake 2000 (n=15) | 15 | 0,000 | 0,000 to 21,802 | 1,29 | 2,42 |
| George 2007 (n=3) | 3 | 0,000 | 0,000 to 70,760 | 0,32 | 1,02 |
| Hedrick 2003 (n=43) | 43 | 0,000 | 0,000 to 8,221 | 3,56 | 3,41 |
| Hedrick 2005 (n=9) | 9 | 0,000 | 0,000 to 33,627 | 0,81 | 1,90 |
| Kern 2007 (n=5) | 5 | 0,000 | 0,000 to 52,182 | 0,49 | 1,37 |
| Kornacki 2017 (n=4) | 4 | 0,000 | 0,000 to 60,236 | 0,40 | 1,21 |
| Kunisaki 2007 (n=14) | 14 | 0,000 | 0,000 to 23,164 | 1,21 | 2,35 |
| Laje 2012 (n=17) | 17 | 0,000 | 0,000 to 19,506 | 1,46 | 2,55 |
| Laje 2013 (n=4) | 4 | 0,000 | 0,000 to 60,236 | 0,40 | 1,21 |
| Laje 2015 (n=13) | 13 | 0,000 | 0,000 to 24,705 | 1,13 | 2,27 |
| Lazar 2011 (n=12) | 12 | 0,000 | 0,000 to 26,465 | 1,05 | 2,19 |
| Noah 2002 (n=34) | 34 | 0,000 | 0,000 to 10,282 | 2,83 | 3,22 |
| Pellicer 2007 (n=3) | 3 | 0,000 | 0,000 to 70,760 | 0,32 | 1,02 |
| Stoffan 2012 (n=7) | 7 | 0,000 | 0,000 to 40,962 | 0,65 | 1,66 |
| Tuncay Ozgunen 2010 (n=3) | 3 | 0,000 | 0,000 to 70,760 | 0,32 | 1,02 |
| Zamora 2013 (n=26) | 26 | 0,000 | 0,000 to 13,227 | 2,18 | 2,97 |
| Bennett 2014 (n=43) | 43 | 0,000 | 0,000 to 8,221 | 3,56 | 3,41 |
| Botelho 2017 (n=45) | 45 | 0,000 | 0,000 to 7,871 | 3,72 | 3,44 |
| Bruner 1999 (n=29) | 29 | 0,000 | 0,000 to 11,944 | 2,43 | 3,07 |
| Bruner 2000 (n=4) | 4 | 0,000 | 0,000 to 60,236 | 0,40 | 1,21 |
| Farmer 2003 (n=12) | 12 | 8,333 | 0,211 to 38,480 | 1,05 | 2,19 |
| Friszer 2016 (n=3) | 3 | 0,000 | 0,000 to 70,760 | 0,32 | 1,02 |
| Johnson 2016 (n=91) | 91 | 5,495 | 1,808 to 12,358 | 7,44 | 3,88 |
| Marenco 2013 (n=4) | 4 | 0,000 | 0,000 to 60,236 | 0,40 | 1,21 |
| Moldenhauer 2015 (n=100) | 100 | 2,000 | 0,243 to 7,038 | 8,17 | 3,93 |
| Moron 2018 (n=237) | 237 | 2,532 | 0,935 to 5,429 | 19,26 | 4,21 |
| Ochsenbein-Kolble 2017 (n=30) | 30 | 0,000 | 0,000 to 11,570 | 2,51 | 3,10 |
| Sinskey 2017 (n=47) | 47 | 0,000 | 0,000 to 7,549 | 3,88 | 3,48 |
| Soni 2016 (n=88) | 88 | 0,000 | 0,000 to 4,105 | 7,20 | 3,87 |
| Zamlynski 2014 (n=46) | 46 | 2,174 | 0,0550 to 11,527 | 3,80 | 3,46 |
| Flake 2000 (n=15) | 15 | 6,667 | 0,169 to 31,948 | 1,29 | 2,42 |
| Harrison 1990 (n=6) | 6 | 0,000 | 0,000 to 45,926 | 0,57 | 1,52 |
| Harrison 1993 (n=14) | 14 | 7,143 | 0,181 to 33,868 | 1,21 | 2,35 |
| Harrison 1998 (n=13) | 13 | 84,615 | 54,553 to 98,079 | 1,13 | 2,27 |
| Adzick 2003 (n=22) | 22 | 9,091 | 1,121 to 29,161 | 1,86 | 2,81 |
| Hedrick 2004 (n=4) | 4 | 25,000 | 0,631 to 80,588 | 0,40 | 1,21 |
| Golombeck 2006 (n=79) | 79 | 27,848 | 18,345 to 39,074 | 6,47 | 3,81 |
| Longaker 1991 (n=17) | 17 | 5,882 | 0,149 to 28,689 | 1,46 | 2,55 |
| Zamora 2013 (n=7) | 7 | 0,000 | 0,000 to 40,962 | 0,65 | 1,66 |
| Total (fixed effects) | 1193 | 3,649 | 2,675 to 4,851 | 100,00 | 100,00 |
| Total (random effects) | 1193 | 4,320 | 2,322 to 6,899 | 100,00 | 100,00 |

## Test for heterogeneity

| Q | 127,5342 |
| --- | --- |
| DF | 42 |
| Significance level | P < 0,0001 |
| I^2^ (inconsistency) | 67,07% |
| 95% CI for I^2^ | 54,77 to 76,02 |

# Meta-analysis: postoperative fetoscopic pulmonary oedema

| Variable for studies | Study |
| --- | --- |
| Variable for total number of cases | N |
| Variable for number of positive cases | Outcome |

| Study | Sample size | Proportion (%) | 95% CI | Weight (%) | |
| --- | --- | --- | --- | --- | --- |
|  |  |  |  | Fixed | Random |
| Aboudiab 2017 (n=18) | 18 | 0,000 | 0,000 to 18,530 | 0,20 | 0,33 |
| Baschat 2013 (n=147) | 147 | 0,000 | 0,000 to 2,478 | 1,55 | 1,55 |
| Chalouhi 2016 (n=22) | 22 | 0,000 | 0,000 to 15,437 | 0,24 | 0,40 |
| Chang 2006 (n=27) | 27 | 0,000 | 0,000 to 12,770 | 0,29 | 0,47 |
| Chang 2016 (n=100) | 100 | 0,000 | 0,000 to 3,622 | 1,06 | 1,24 |
| Chmait 2013 (n=318) | 318 | 0,000 | 0,000 to 1,153 | 3,35 | 2,18 |
| Chmait 2017 (n=19) | 19 | 0,000 | 0,000 to 17,647 | 0,21 | 0,35 |
| Crombleholme 2007 (n=20) | 20 | 0,000 | 0,000 to 16,843 | 0,22 | 0,37 |
| De Lia 1995 (n=26) | 26 | 0,000 | 0,000 to 13,227 | 0,28 | 0,46 |
| De Lia 1999 (n=67) | 67 | 0,000 | 0,000 to 5,357 | 0,71 | 0,95 |
| De Lia 2009 (n=10) | 10 | 0,000 | 0,000 to 30,850 | 0,12 | 0,20 |
| Deprest 1998 (n=6) | 6 | 0,000 | 0,000 to 45,926 | 0,073 | 0,13 |
| Draga 2016 (n=37) | 37 | 0,000 | 0,000 to 9,489 | 0,40 | 0,61 |
| Duron 2014 (n=85) | 85 | 3,529 | 0,734 to 9,970 | 0,90 | 1,12 |
| Ek 2012 (n=) | 67 | 0,000 | 0,000 to 5,357 | 0,71 | 0,95 |
| Habli 2009 (n=152) | 152 | 0,000 | 0,000 to 2,398 | 1,61 | 1,58 |
| Has 2014 (n=85) | 85 | 0,000 | 0,000 to 4,247 | 0,90 | 1,12 |
| Hecher 2000 (n=200) | 200 | 0,000 | 0,000 to 1,828 | 2,11 | 1,81 |
| Hernandez-Andrade 2011 (n=35) | 35 | 0,000 | 0,000 to 10,003 | 0,38 | 0,58 |
| Huber 2008 (n=176) | 176 | 0,000 | 0,000 to 2,074 | 1,86 | 1,70 |
| Ishii 2014 (n=16) | 16 | 0,000 | 0,000 to 20,591 | 0,18 | 0,30 |
| Ishii 2015 (n=10) | 10 | 0,000 | 0,000 to 30,850 | 0,12 | 0,20 |
| Lanna 2017 (n=373) | 373 | 0,000 | 0,000 to 0,984 | 3,93 | 2,30 |
| Lecointre 2017 (n=200) | 200 | 0,000 | 0,000 to 1,828 | 2,11 | 1,81 |
| Malshe 2017 (n=203) | 203 | 0,000 | 0,000 to 1,801 | 2,14 | 1,82 |
| Martinez 2012 (n=500) | 500 | 0,000 | 0,000 to 0,735 | 5,26 | 2,50 |
| Middeldorp 2007 (n=100) | 100 | 1,000 | 0,0253 to 5,446 | 1,06 | 1,24 |
| Miyadahira 2018 (n=67) | 67 | 0,000 | 0,000 to 5,357 | 0,71 | 0,95 |
| Molina-Garcia 2009 (n=22) | 22 | 0,000 | 0,000 to 15,437 | 0,24 | 0,40 |
| Morris 2010 (n=164) | 164 | 0,000 | 0,000 to 2,224 | 1,73 | 1,64 |
| Mullers 2015 (n=105) | 105 | 0,000 | 0,000 to 3,452 | 1,11 | 1,28 |
| Nakata 2016 (n=6) | 6 | 16,667 | 0,421 to 64,123 | 0,073 | 0,13 |
| Nguyen 2012 (n=98) | 98 | 0,000 | 0,000 to 3,694 | 1,04 | 1,23 |
| Ozawa 2017 (n=11) | 11 | 0,000 | 0,000 to 28,491 | 0,13 | 0,22 |
| Papanna 2010 (n=48) | 48 | 0,000 | 0,000 to 7,397 | 0,51 | 0,74 |
| Papanna 2012 (n=163) | 163 | 0,000 | 0,000 to 2,238 | 1,72 | 1,64 |
| Peeters 2014 (n=338) | 338 | 0,000 | 0,000 to 1,085 | 3,56 | 2,23 |
| Persico 2016 (n=106) | 106 | 0,000 | 0,000 to 3,420 | 1,12 | 1,29 |
| Quintero 2000 (n=92) | 92 | 0,000 | 0,000 to 3,930 | 0,98 | 1,18 |
| Quintero 2001 (n=11) | 11 | 0,000 | 0,000 to 28,491 | 0,13 | 0,22 |
| Rossi 2008 (n=266) | 266 | 1,128 | 0,233 to 3,260 | 2,80 | 2,04 |
| Ruano 2009 (n=19) | 19 | 0,000 | 0,000 to 17,647 | 0,21 | 0,35 |
| Ruegg 2018 (n=37) | 37 | 8,108 | 1,704 to 21,910 | 0,40 | 0,61 |
| Rustico 2012 (n=150) | 150 | 0,667 | 0,0169 to 3,658 | 1,59 | 1,57 |
| Said 2008 (n=10) | 10 | 0,000 | 0,000 to 30,850 | 0,12 | 0,20 |
| Senat 2004 (n=72) | 72 | 0,000 | 0,000 to 4,994 | 0,77 | 1,00 |
| Sepulveda 2007 (n=33) | 33 | 0,000 | 0,000 to 10,576 | 0,36 | 0,55 |
| Shamshirsaz 2015 (n=55) | 55 | 0,000 | 0,000 to 6,487 | 0,59 | 0,82 |
| Slaghekke 2014 (n=274) | 274 | 0,000 | 0,000 to 1,337 | 2,89 | 2,06 |
| Taniguchi 2015 (n=3) | 3 | 0,000 | 0,000 to 70,760 | 0,042 | 0,076 |
| Tchirikov 2011 (n=80) | 80 | 0,000 | 0,000 to 4,506 | 0,85 | 1,07 |
| Teoh 2013 (n=49) | 49 | 0,000 | 0,000 to 7,252 | 0,52 | 0,76 |
| Thia 2017 (n=5) | 5 | 0,000 | 0,000 to 52,182 | 0,063 | 0,11 |
| Ville 1997 (n=132) | 132 | 0,758 | 0,0192 to 4,149 | 1,40 | 1,46 |
| Ville 1998 (n=44) | 44 | 2,273 | 0,0575 to 12,024 | 0,47 | 0,70 |
| Weingertner 2011 (n=100) | 100 | 0,000 | 0,000 to 3,622 | 1,06 | 1,24 |
| Wilson 2016 (n=151) | 151 | 0,000 | 0,000 to 2,413 | 1,60 | 1,57 |
| Yamamoto 2005 (n=175) | 175 | 0,000 | 0,000 to 2,086 | 1,85 | 1,70 |
| Yang 2010 (n=30) | 30 | 0,000 | 0,000 to 11,570 | 0,33 | 0,51 |
| Zaretsky 2018 (n=749) | 749 | 0,000 | 0,000 to 0,491 | 7,87 | 2,73 |
| Zhao 2016 (n=62) | 62 | 0,000 | 0,000 to 5,776 | 0,66 | 0,90 |
| Bebbington 2012 (n=146) | 146 | 0,000 | 0,000 to 2,495 | 1,54 | 1,55 |
| Berg 2014 (n=7) | 7 | 0,000 | 0,000 to 40,962 | 0,084 | 0,15 |
| Delabaere 2013 (n=30) | 30 | 0,000 | 0,000 to 11,570 | 0,33 | 0,51 |
| Deprest 2000 (n=10) | 10 | 0,000 | 0,000 to 30,850 | 0,12 | 0,20 |
| Gallot 2003 (n=11) | 11 | 0,000 | 0,000 to 28,491 | 0,13 | 0,22 |
| Gouverneur 2009 (n=54) | 54 | 0,000 | 0,000 to 6,603 | 0,58 | 0,81 |
| Gul 2008 (n=9) | 9 | 0,000 | 0,000 to 33,627 | 0,10 | 0,18 |
| Has 2014 (n=71) | 71 | 0,000 | 0,000 to 5,063 | 0,76 | 0,99 |
| He 2010 (n=14) | 14 | 0,000 | 0,000 to 23,164 | 0,16 | 0,27 |
| Ilagan 2008 (n=27) | 27 | 0,000 | 0,000 to 12,770 | 0,29 | 0,47 |
| Jelin 2010 (n=7) | 7 | 0,000 | 0,000 to 40,962 | 0,084 | 0,15 |
| King 2017 (n=43) | 43 | 0,000 | 0,000 to 8,221 | 0,46 | 0,68 |
| Lanna 2012 (n=118) | 118 | 0,000 | 0,000 to 3,078 | 1,25 | 1,37 |
| Lee 2013 (n=98) | 98 | 0,000 | 0,000 to 3,694 | 1,04 | 1,23 |
| Lewi 2006 (n=80) | 80 | 0,000 | 0,000 to 4,506 | 0,85 | 1,07 |
| Moise 2008 (n=9) | 9 | 0,000 | 0,000 to 33,627 | 0,10 | 0,18 |
| Nobili 2013 (n=48) | 48 | 0,000 | 0,000 to 7,397 | 0,51 | 0,74 |
| Paramasivam 2010 (n=35) | 35 | 0,000 | 0,000 to 10,003 | 0,38 | 0,58 |
| Peng 2016 (n=93) | 93 | 0,000 | 0,000 to 3,889 | 0,99 | 1,19 |
| Quintero 1996 (n=13) | 13 | 0,000 | 0,000 to 24,705 | 0,15 | 0,25 |
| Quintero 2006 (n=51) | 51 | 0,000 | 0,000 to 6,978 | 0,55 | 0,78 |
| Roman 2010 (n=60) | 60 | 0,000 | 0,000 to 5,963 | 0,64 | 0,88 |
| Schou 2018 (n=102) | 102 | 0,000 | 0,000 to 3,552 | 1,08 | 1,26 |
| Sugibayashi 2016 (n=40) | 40 | 0,000 | 0,000 to 8,810 | 0,43 | 0,65 |
| Takano 2015 (n=10) | 10 | 0,000 | 0,000 to 30,850 | 0,12 | 0,20 |
| Taylor 2002 (n=15) | 15 | 0,000 | 0,000 to 21,802 | 0,17 | 0,29 |
| Tsao 2002 (n=13) | 13 | 0,000 | 0,000 to 24,705 | 0,15 | 0,25 |
| Zhang 2018 (n=25) | 25 | 0,000 | 0,000 to 13,719 | 0,27 | 0,44 |
| Deprest 2005 (n=20) | 20 | 0,000 | 0,000 to 16,843 | 0,22 | 0,37 |
| Harrison 1998 (n=8) | 8 | 37,500 | 8,523 to 75,514 | 0,094 | 0,17 |
| Harrison 2003 (n=11) | 11 | 27,273 | 6,022 to 60,974 | 0,13 | 0,22 |
| Jani 2005 (n=24) | 24 | 0,000 | 0,000 to 14,247 | 0,26 | 0,43 |
| Jani 2006 (n=28) | 28 | 0,000 | 0,000 to 12,344 | 0,30 | 0,48 |
| Jani 2009 (n=210) | 210 | 0,000 | 0,000 to 1,741 | 2,22 | 1,85 |
| Jimenez 2017 (n=201) | 201 | 0,000 | 0,000 to 1,819 | 2,12 | 1,81 |
| Kosinski 2017 (n=28) | 28 | 0,000 | 0,000 to 12,344 | 0,30 | 0,48 |
| Manrique 2008 (n=11) | 11 | 0,000 | 0,000 to 28,491 | 0,13 | 0,22 |
| Peralta 2011 (n=8) | 8 | 0,000 | 0,000 to 36,942 | 0,094 | 0,17 |
| Persico 2017 (n=21) | 21 | 0,000 | 0,000 to 16,110 | 0,23 | 0,38 |
| Ruano 2012 (n=35) | 35 | 0,000 | 0,000 to 10,003 | 0,38 | 0,58 |
| Ruano 2012 (n=20) | 20 | 0,000 | 0,000 to 16,843 | 0,22 | 0,37 |
| Ruano 2013 (n=17) | 17 | 0,000 | 0,000 to 19,506 | 0,19 | 0,32 |
| Arens 2017 (n=59) | 59 | 1,695 | 0,0429 to 9,086 | 0,63 | 0,87 |
| Belfort 2017 (n=22) | 22 | 9,091 | 1,121 to 29,161 | 0,24 | 0,40 |
| Bruner 2000 (n=4) | 4 | 25,000 | 0,631 to 80,588 | 0,052 | 0,095 |
| Degenhardt 2014 (n=51) | 51 | 1,961 | 0,0496 to 10,447 | 0,55 | 0,78 |
| Kohn 2018 (n=34) | 34 | 0,000 | 0,000 to 10,282 | 0,37 | 0,57 |
| Pedreira 2014 (n=4) | 4 | 0,000 | 0,000 to 60,236 | 0,052 | 0,095 |
| Pedreira 2016 (n=10) | 10 | 0,000 | 0,000 to 30,850 | 0,12 | 0,20 |
| Verbeek 2012 (n=19) | 19 | 0,000 | 0,000 to 17,647 | 0,21 | 0,35 |
| Ziemann 2018 (n=65) | 65 | 0,000 | 0,000 to 5,517 | 0,69 | 0,93 |
| Morris 2013 (n=16) | 16 | 0,000 | 0,000 to 20,591 | 0,18 | 0,30 |
| Ruano 2010 (n=11) | 11 | 0,000 | 0,000 to 28,491 | 0,13 | 0,22 |
| Welsh 2003 (n=13) | 13 | 0,000 | 0,000 to 24,705 | 0,15 | 0,25 |
| Cavalheiro 2011 (n=30) | 30 | 0,000 | 0,000 to 11,570 | 0,33 | 0,51 |
| Mallman 2017 (n=78) | 78 | 0,000 | 0,000 to 4,619 | 0,83 | 1,06 |
| Golombeck 2006 (n=99) | 99 | 17,172 | 10,333 to 26,064 | 1,05 | 1,23 |
| Kohl 2006 (n=16) | 16 | 6,250 | 0,158 to 30,232 | 0,18 | 0,30 |
| Kohl 2010 (n=37) | 37 | 0,000 | 0,000 to 9,489 | 0,40 | 0,61 |
| Nivatpumin 2016 (n=152) | 152 | 3,289 | 1,077 to 7,509 | 1,61 | 1,58 |
| Peralta 2010 (n=56) | 56 | 0,000 | 0,000 to 6,375 | 0,60 | 0,84 |
| Total (fixed effects) | 9403 | 0,437 | 0,315 to 0,592 | 100,00 | 100,00 |
| Total (random effects) | 9403 | 0,630 | 0,432 to 0,865 | 100,00 | 100,00 |

## Test for heterogeneity

| Q | 175,1532 |
| --- | --- |
| DF | 121 |
| Significance level | P = 0,0009 |
| I^2^ (inconsistency) | 30,92% |
| 95% CI for I^2^ | 13,31 to 44,95 |

# Meta-analysis: delivery open rupture

| Variable for studies | Study |
| --- | --- |
| Variable for total number of cases | N |
| Variable for number of positive cases | Outcome |

| Study | Sample size | Proportion (%) | 95% CI | Weight (%) | |
| --- | --- | --- | --- | --- | --- |
|  |  |  |  | Fixed | Random |
| Bennett 2014 (n=43) | 43 | 0,000 | 0,000 to 8,221 | 4,49 | 4,49 |
| Botelho 2017 (n=45) | 45 | 0,000 | 0,000 to 7,871 | 4,70 | 4,70 |
| Bruner 1999 (n=29) | 29 | 3,448 | 0,0873 to 17,764 | 3,06 | 3,06 |
| Bruner 2000 (n=4) | 4 | 0,000 | 0,000 to 60,236 | 0,51 | 0,51 |
| Farmer 2003 (n=12) | 12 | 0,000 | 0,000 to 26,465 | 1,33 | 1,33 |
| Friszer 2016 (n=3) | 3 | 0,000 | 0,000 to 70,760 | 0,41 | 0,41 |
| Johnson 2016 (n=91) | 91 | 0,000 | 0,000 to 3,973 | 9,40 | 9,40 |
| Marenco 2013 (n=4) | 4 | 0,000 | 0,000 to 60,236 | 0,51 | 0,51 |
| Moldenhauer 2015 (n=100) | 100 | 0,000 | 0,000 to 3,622 | 10,32 | 10,32 |
| Moron 2018 (n=237) | 237 | 0,844 | 0,102 to 3,015 | 24,31 | 24,31 |
| Ochsenbein-Kolble 2017 (n=30) | 30 | 0,000 | 0,000 to 11,570 | 3,17 | 3,17 |
| Sinskey 2017 (n=47) | 47 | 0,000 | 0,000 to 7,549 | 4,90 | 4,90 |
| Soni 2016 (n=88) | 88 | 0,000 | 0,000 to 4,105 | 9,09 | 9,09 |
| Zamlynski 2014 (n=46) | 46 | 2,174 | 0,0550 to 11,527 | 4,80 | 4,80 |
| Flake 2000 (n=15) | 15 | 0,000 | 0,000 to 21,802 | 1,63 | 1,63 |
| Harrison 1990 (n=6) | 6 | 0,000 | 0,000 to 45,926 | 0,72 | 0,72 |
| Harrison 1993 (n=14) | 14 | 0,000 | 0,000 to 23,164 | 1,53 | 1,53 |
| Harrison 1998 (n=13) | 13 | 0,000 | 0,000 to 24,705 | 1,43 | 1,43 |
| Adzick 2003 (n=22) | 22 | 0,000 | 0,000 to 15,437 | 2,35 | 2,35 |
| Hedrick 2004 (n=4) | 4 | 0,000 | 0,000 to 60,236 | 0,51 | 0,51 |
| Golombeck 2006 (n=79) | 79 | 0,000 | 0,000 to 4,562 | 8,17 | 8,17 |
| Longaker 1991 (n=17) | 17 | 0,000 | 0,000 to 19,506 | 1,84 | 1,84 |
| Zamora 2013 (n=7) | 7 | 14,286 | 0,361 to 57,872 | 0,82 | 0,82 |
| Total (fixed effects) | 956 | 0,900 | 0,409 to 1,713 | 100,00 | 100,00 |
| Total (random effects) | 956 | 0,900 | 0,405 to 1,588 | 100,00 | 100,00 |

## Test for heterogeneity

| Q | 12,2124 |
| --- | --- |
| DF | 22 |
| Significance level | P = 0,9528 |
| I^2^ (inconsistency) | 0,00% |
| 95% CI for I^2^ | 0,00 to 1,64 |

# Meta-analysis: delivery open dehiscence

| Variable for studies | Study |
| --- | --- |
| Variable for total number of cases | N |
| Variable for number of positive cases | Outcome |

| Study | Sample size | Proportion (%) | 95% CI | Weight (%) | |
| --- | --- | --- | --- | --- | --- |
|  |  |  |  | Fixed | Random |
| Bennett 2014 (n=43) | 43 | 6,977 | 1,463 to 19,061 | 4,49 | 5,75 |
| Botelho 2017 (n=45) | 45 | 4,444 | 0,543 to 15,149 | 4,70 | 5,87 |
| Bruner 1999 (n=29) | 29 | 0,000 | 0,000 to 11,944 | 3,06 | 4,68 |
| Bruner 2000 (n=4) | 4 | 25,000 | 0,631 to 80,588 | 0,51 | 1,19 |
| Farmer 2003 (n=12) | 12 | 0,000 | 0,000 to 26,465 | 1,33 | 2,65 |
| Friszer 2016 (n=3) | 3 | 0,000 | 0,000 to 70,760 | 0,41 | 0,97 |
| Johnson 2016 (n=91) | 91 | 10,989 | 5,397 to 19,282 | 9,40 | 7,72 |
| Marenco 2013 (n=4) | 4 | 0,000 | 0,000 to 60,236 | 0,51 | 1,19 |
| Moldenhauer 2015 (n=100) | 100 | 7,000 | 2,861 to 13,892 | 10,32 | 7,94 |
| Moron 2018 (n=237) | 237 | 2,954 | 1,196 to 5,991 | 24,31 | 9,57 |
| Ochsenbein-Kolble 2017 (n=30) | 30 | 0,000 | 0,000 to 11,570 | 3,17 | 4,77 |
| Sinskey 2017 (n=47) | 47 | 0,000 | 0,000 to 7,549 | 4,90 | 5,99 |
| Soni 2016 (n=88) | 88 | 0,000 | 0,000 to 4,105 | 9,09 | 7,64 |
| Zamlynski 2014 (n=46) | 46 | 10,870 | 3,625 to 23,570 | 4,80 | 5,93 |
| Flake 2000 (n=15) | 15 | 0,000 | 0,000 to 21,802 | 1,63 | 3,09 |
| Harrison 1990 (n=6) | 6 | 0,000 | 0,000 to 45,926 | 0,72 | 1,60 |
| Harrison 1993 (n=14) | 14 | 0,000 | 0,000 to 23,164 | 1,53 | 2,95 |
| Harrison 1998 (n=13) | 13 | 0,000 | 0,000 to 24,705 | 1,43 | 2,80 |
| Adzick 2003 (n=22) | 22 | 4,545 | 0,115 to 22,844 | 2,35 | 3,97 |
| Hedrick 2004 (n=4) | 4 | 0,000 | 0,000 to 60,236 | 0,51 | 1,19 |
| Golombeck 2006 (n=79) | 79 | 0,000 | 0,000 to 4,562 | 8,17 | 7,37 |
| Longaker 1991 (n=17) | 17 | 0,000 | 0,000 to 19,506 | 1,84 | 3,37 |
| Zamora 2013 (n=7) | 7 | 14,286 | 0,361 to 57,872 | 0,82 | 1,79 |
| Total (fixed effects) | 956 | 3,596 | 2,521 to 4,962 | 100,00 | 100,00 |
| Total (random effects) | 956 | 3,674 | 2,010 to 5,812 | 100,00 | 100,00 |

## Test for heterogeneity

| Q | 42,7065 |
| --- | --- |
| DF | 22 |
| Significance level | P = 0,0051 |
| I^2^ (inconsistency) | 48,49% |
| 95% CI for I^2^ | 16,39 to 68,26 |

# Meta-analysis: delivery open transfusion

| Variable for studies | Study |
| --- | --- |
| Variable for total number of cases | N |
| Variable for number of positive cases | Outcome |

| Study | Sample size | Proportion (%) | 95% CI | Weight (%) | |
| --- | --- | --- | --- | --- | --- |
|  |  |  |  | Fixed | Random |
| Barthod 2013 (n=5) | 5 | 0,000 | 0,000 to 52,182 | 0,49 | 0,49 |
| Cass 2013 (n=9) | 9 | 0,000 | 0,000 to 33,627 | 0,81 | 0,81 |
| Chen 2018 (n=7) | 7 | 0,000 | 0,000 to 40,962 | 0,65 | 0,65 |
| Dahlgren 2004 (n=4) | 4 | 0,000 | 0,000 to 60,236 | 0,40 | 0,40 |
| Flake 2000 (n=15) | 15 | 0,000 | 0,000 to 21,802 | 1,29 | 1,29 |
| George 2007 (n=3) | 3 | 0,000 | 0,000 to 70,760 | 0,32 | 0,32 |
| Hedrick 2003 (n=43) | 43 | 0,000 | 0,000 to 8,221 | 3,56 | 3,56 |
| Hedrick 2005 (n=9) | 9 | 0,000 | 0,000 to 33,627 | 0,81 | 0,81 |
| Kern 2007 (n=5) | 5 | 0,000 | 0,000 to 52,182 | 0,49 | 0,49 |
| Kornacki 2017 (n=4) | 4 | 0,000 | 0,000 to 60,236 | 0,40 | 0,40 |
| Kunisaki 2007 (n=14) | 14 | 0,000 | 0,000 to 23,164 | 1,21 | 1,21 |
| Laje 2012 (n=17) | 17 | 0,000 | 0,000 to 19,506 | 1,46 | 1,46 |
| Laje 2013 (n=4) | 4 | 0,000 | 0,000 to 60,236 | 0,40 | 0,40 |
| Laje 2015 (n=13) | 13 | 0,000 | 0,000 to 24,705 | 1,13 | 1,13 |
| Lazar 2011 (n=12) | 12 | 0,000 | 0,000 to 26,465 | 1,05 | 1,05 |
| Noah 2002 (n=34) | 34 | 0,000 | 0,000 to 10,282 | 2,83 | 2,83 |
| Pellicer 2007 (n=3) | 3 | 0,000 | 0,000 to 70,760 | 0,32 | 0,32 |
| Stoffan 2012 (n=7) | 7 | 0,000 | 0,000 to 40,962 | 0,65 | 0,65 |
| Tuncay Ozgunen 2010 (n=3) | 3 | 0,000 | 0,000 to 70,760 | 0,32 | 0,32 |
| Zamora 2013 (n=26) | 26 | 0,000 | 0,000 to 13,227 | 2,18 | 2,18 |
| Bennett 2014 (n=43) | 43 | 0,000 | 0,000 to 8,221 | 3,56 | 3,56 |
| Botelho 2017 (n=45) | 45 | 0,000 | 0,000 to 7,871 | 3,72 | 3,72 |
| Bruner 1999 (n=29) | 29 | 0,000 | 0,000 to 11,944 | 2,43 | 2,43 |
| Bruner 2000 (n=4) | 4 | 0,000 | 0,000 to 60,236 | 0,40 | 0,40 |
| Farmer 2003 (n=12) | 12 | 0,000 | 0,000 to 26,465 | 1,05 | 1,05 |
| Friszer 2016 (n=3) | 3 | 0,000 | 0,000 to 70,760 | 0,32 | 0,32 |
| Johnson 2016 (n=91) | 91 | 8,791 | 3,872 to 16,589 | 7,44 | 7,44 |
| Marenco 2013 (n=4) | 4 | 0,000 | 0,000 to 60,236 | 0,40 | 0,40 |
| Moldenhauer 2015 (n=100) | 100 | 3,000 | 0,623 to 8,518 | 8,17 | 8,17 |
| Moron 2018 (n=237) | 237 | 2,110 | 0,688 to 4,854 | 19,26 | 19,26 |
| Ochsenbein-Kolble 2017 (n=30) | 30 | 0,000 | 0,000 to 11,570 | 2,51 | 2,51 |
| Sinskey 2017 (n=47) | 47 | 0,000 | 0,000 to 7,549 | 3,88 | 3,88 |
| Soni 2016 (n=88) | 88 | 0,000 | 0,000 to 4,105 | 7,20 | 7,20 |
| Zamlynski 2014 (n=46) | 46 | 0,000 | 0,000 to 7,706 | 3,80 | 3,80 |
| Flake 2000 (n=15) | 15 | 0,000 | 0,000 to 21,802 | 1,29 | 1,29 |
| Harrison 1990 (n=6) | 6 | 0,000 | 0,000 to 45,926 | 0,57 | 0,57 |
| Harrison 1993 (n=14) | 14 | 0,000 | 0,000 to 23,164 | 1,21 | 1,21 |
| Harrison 1998 (n=13) | 13 | 0,000 | 0,000 to 24,705 | 1,13 | 1,13 |
| Adzick 2003 (n=22) | 22 | 0,000 | 0,000 to 15,437 | 1,86 | 1,86 |
| Hedrick 2004 (n=4) | 4 | 0,000 | 0,000 to 60,236 | 0,40 | 0,40 |
| Golombeck 2006 (n=79) | 79 | 0,000 | 0,000 to 4,562 | 6,47 | 6,47 |
| Longaker 1991 (n=17) | 17 | 0,000 | 0,000 to 19,506 | 1,46 | 1,46 |
| Zamora 2013 (n=7) | 7 | 14,286 | 0,361 to 57,872 | 0,65 | 0,65 |
| Total (fixed effects) | 1193 | 1,829 | 1,157 to 2,741 | 100,00 | 100,00 |
| Total (random effects) | 1193 | 1,829 | 1,157 to 2,650 | 100,00 | 100,00 |

## Test for heterogeneity

| Q | 27,1056 |
| --- | --- |
| DF | 42 |
| Significance level | P = 0,9637 |
| I^2^ (inconsistency) | 0,00% |
| 95% CI for I^2^ | 0,00 to 0,00 |

# Meta-analysis: combined open severe

| Variable for studies | Study |
| --- | --- |
| Variable for total number of cases | N |
| Variable for number of positive cases | Outcome |

| Study | Sample size | Proportion (%) | 95% CI | Weight (%) | |
| --- | --- | --- | --- | --- | --- |
|  |  |  |  | Fixed | Random |
| Barthod 2013 (n=5) | 5 | 0,000 | 0,000 to 52,182 | 0,49 | 0,66 |
| Cass 2013 (n=9) | 9 | 0,000 | 0,000 to 33,627 | 0,81 | 1,07 |
| Chen 2018 (n=7) | 7 | 0,000 | 0,000 to 40,962 | 0,65 | 0,87 |
| Dahlgren 2004 (n=4) | 4 | 0,000 | 0,000 to 60,236 | 0,40 | 0,55 |
| Flake 2000 (n=15) | 15 | 0,000 | 0,000 to 21,802 | 1,29 | 1,66 |
| George 2007 (n=3) | 3 | 0,000 | 0,000 to 70,760 | 0,32 | 0,44 |
| Hedrick 2003 (n=43) | 43 | 2,326 | 0,0589 to 12,289 | 3,56 | 3,96 |
| Hedrick 2005 (n=9) | 9 | 0,000 | 0,000 to 33,627 | 0,81 | 1,07 |
| Kern 2007 (n=5) | 5 | 0,000 | 0,000 to 52,182 | 0,49 | 0,66 |
| Kornacki 2017 (n=4) | 4 | 0,000 | 0,000 to 60,236 | 0,40 | 0,55 |
| Kunisaki 2007 (n=14) | 14 | 0,000 | 0,000 to 23,164 | 1,21 | 1,56 |
| Laje 2012 (n=17) | 17 | 5,882 | 0,149 to 28,689 | 1,46 | 1,85 |
| Laje 2013 (n=4) | 4 | 0,000 | 0,000 to 60,236 | 0,40 | 0,55 |
| Laje 2015 (n=13) | 13 | 0,000 | 0,000 to 24,705 | 1,13 | 1,47 |
| Lazar 2011 (n=12) | 12 | 0,000 | 0,000 to 26,465 | 1,05 | 1,37 |
| Noah 2002 (n=34) | 34 | 0,000 | 0,000 to 10,282 | 2,83 | 3,29 |
| Pellicer 2007 (n=3) | 3 | 33,333 | 0,840 to 90,570 | 0,32 | 0,44 |
| Stoffan 2012 (n=7) | 7 | 14,286 | 0,361 to 57,872 | 0,65 | 0,87 |
| Tuncay Ozgunen 2010 (n=3) | 3 | 0,000 | 0,000 to 70,760 | 0,32 | 0,44 |
| Zamora 2013 (n=26) | 26 | 3,846 | 0,0973 to 19,637 | 2,18 | 2,64 |
| Bennett 2014 (n=43) | 43 | 4,651 | 0,568 to 15,811 | 3,56 | 3,96 |
| Botelho 2017 (n=45) | 45 | 2,222 | 0,0562 to 11,770 | 3,72 | 4,10 |
| Bruner 1999 (n=29) | 29 | 13,793 | 3,889 to 31,664 | 2,43 | 2,89 |
| Bruner 2000 (n=4) | 4 | 0,000 | 0,000 to 60,236 | 0,40 | 0,55 |
| Farmer 2003 (n=12) | 12 | 0,000 | 0,000 to 26,465 | 1,05 | 1,37 |
| Friszer 2016 (n=3) | 3 | 0,000 | 0,000 to 70,760 | 0,32 | 0,44 |
| Johnson 2016 (n=91) | 91 | 6,593 | 2,458 to 13,800 | 7,44 | 6,74 |
| Marenco 2013 (n=4) | 4 | 0,000 | 0,000 to 60,236 | 0,40 | 0,55 |
| Moldenhauer 2015 (n=100) | 100 | 3,000 | 0,623 to 8,518 | 8,17 | 7,15 |
| Moron 2018 (n=237) | 237 | 2,110 | 0,688 to 4,854 | 19,26 | 11,16 |
| Ochsenbein-Kolble 2017 (n=30) | 30 | 10,000 | 2,112 to 26,529 | 2,51 | 2,97 |
| Sinskey 2017 (n=47) | 47 | 0,000 | 0,000 to 7,549 | 3,88 | 4,24 |
| Soni 2016 (n=88) | 88 | 0,000 | 0,000 to 4,105 | 7,20 | 6,60 |
| Zamlynski 2014 (n=46) | 46 | 10,870 | 3,625 to 23,570 | 3,80 | 4,17 |
| Flake 2000 (n=15) | 15 | 6,667 | 0,169 to 31,948 | 1,29 | 1,66 |
| Harrison 1990 (n=6) | 6 | 0,000 | 0,000 to 45,926 | 0,57 | 0,76 |
| Harrison 1993 (n=14) | 14 | 7,143 | 0,181 to 33,868 | 1,21 | 1,56 |
| Harrison 1998 (n=13) | 13 | 0,000 | 0,000 to 24,705 | 1,13 | 1,47 |
| Adzick 2003 (n=22) | 22 | 9,091 | 1,121 to 29,161 | 1,86 | 2,30 |
| Hedrick 2004 (n=4) | 4 | 0,000 | 0,000 to 60,236 | 0,40 | 0,55 |
| Golombeck 2006 (n=79) | 79 | 11,392 | 5,344 to 20,528 | 6,47 | 6,15 |
| Longaker 1991 (n=17) | 17 | 0,000 | 0,000 to 19,506 | 1,46 | 1,85 |
| Zamora 2013 (n=7) | 7 | 14,286 | 0,361 to 57,872 | 0,65 | 0,87 |
| Total (fixed effects) | 1193 | 4,220 | 3,169 to 5,495 | 100,00 | 100,00 |
| Total (random effects) | 1193 | 4,514 | 3,242 to 5,983 | 100,00 | 100,00 |

## Test for heterogeneity

| Q | 48,8694 |
| --- | --- |
| DF | 42 |
| Significance level | P = 0,2165 |
| I^2^ (inconsistency) | 14,06% |
| 95% CI for I^2^ | 0,00 to 41,38 |

# Meta-analysis: conbined open minor

| Variable for studies | Study |
| --- | --- |
| Variable for total number of cases | N |
| Variable for number of positive cases | Outcome |

| Study | Sample size | Proportion (%) | 95% CI | Weight (%) | |
| --- | --- | --- | --- | --- | --- |
|  |  |  |  | Fixed | Random |
| Barthod 2013 (n=5) | 5 | 0,000 | 0,000 to 52,182 | 0,49 | 1,71 |
| Cass 2013 (n=9) | 9 | 22,222 | 2,814 to 60,009 | 0,81 | 2,14 |
| Chen 2018 (n=7) | 7 | 0,000 | 0,000 to 40,962 | 0,65 | 1,96 |
| Dahlgren 2004 (n=4) | 4 | 75,000 | 19,412 to 99,369 | 0,40 | 1,56 |
| Flake 2000 (n=15) | 15 | 0,000 | 0,000 to 21,802 | 1,29 | 2,49 |
| George 2007 (n=3) | 3 | 100,000 | 29,240 to 100,000 | 0,32 | 1,37 |
| Hedrick 2003 (n=43) | 43 | 13,953 | 5,298 to 27,932 | 3,56 | 3,00 |
| Hedrick 2005 (n=9) | 9 | 22,222 | 2,814 to 60,009 | 0,81 | 2,14 |
| Kern 2007 (n=5) | 5 | 20,000 | 0,505 to 71,642 | 0,49 | 1,71 |
| Kornacki 2017 (n=4) | 4 | 0,000 | 0,000 to 60,236 | 0,40 | 1,56 |
| Kunisaki 2007 (n=14) | 14 | 0,000 | 0,000 to 23,164 | 1,21 | 2,44 |
| Laje 2012 (n=17) | 17 | 23,529 | 6,811 to 49,899 | 1,46 | 2,56 |
| Laje 2013 (n=4) | 4 | 50,000 | 6,759 to 93,241 | 0,40 | 1,56 |
| Laje 2015 (n=13) | 13 | 0,000 | 0,000 to 24,705 | 1,13 | 2,39 |
| Lazar 2011 (n=12) | 12 | 16,667 | 2,086 to 48,414 | 1,05 | 2,34 |
| Noah 2002 (n=34) | 34 | 38,235 | 22,167 to 56,436 | 2,83 | 2,91 |
| Pellicer 2007 (n=3) | 3 | 0,000 | 0,000 to 70,760 | 0,32 | 1,37 |
| Stoffan 2012 (n=7) | 7 | 0,000 | 0,000 to 40,962 | 0,65 | 1,96 |
| Tuncay Ozgunen 2010 (n=3) | 3 | 0,000 | 0,000 to 70,760 | 0,32 | 1,37 |
| Zamora 2013 (n=26) | 26 | 26,923 | 11,573 to 47,787 | 2,18 | 2,79 |
| Bennett 2014 (n=43) | 43 | 0,000 | 0,000 to 8,221 | 3,56 | 3,00 |
| Botelho 2017 (n=45) | 45 | 2,222 | 0,0562 to 11,770 | 3,72 | 3,02 |
| Bruner 1999 (n=29) | 29 | 0,000 | 0,000 to 11,944 | 2,43 | 2,84 |
| Bruner 2000 (n=4) | 4 | 0,000 | 0,000 to 60,236 | 0,40 | 1,56 |
| Farmer 2003 (n=12) | 12 | 41,667 | 15,165 to 72,333 | 1,05 | 2,34 |
| Friszer 2016 (n=3) | 3 | 0,000 | 0,000 to 70,760 | 0,32 | 1,37 |
| Johnson 2016 (n=91) | 91 | 16,484 | 9,530 to 25,726 | 7,44 | 3,20 |
| Marenco 2013 (n=4) | 4 | 0,000 | 0,000 to 60,236 | 0,40 | 1,56 |
| Moldenhauer 2015 (n=100) | 100 | 10,000 | 4,900 to 17,622 | 8,17 | 3,22 |
| Moron 2018 (n=237) | 237 | 7,595 | 4,563 to 11,738 | 19,26 | 3,32 |
| Ochsenbein-Kolble 2017 (n=30) | 30 | 0,000 | 0,000 to 11,570 | 2,51 | 2,86 |
| Sinskey 2017 (n=47) | 47 | 4,255 | 0,520 to 14,541 | 3,88 | 3,03 |
| Soni 2016 (n=88) | 88 | 4,545 | 1,252 to 11,231 | 7,20 | 3,19 |
| Zamlynski 2014 (n=46) | 46 | 10,870 | 3,625 to 23,570 | 3,80 | 3,02 |
| Flake 2000 (n=15) | 15 | 13,333 | 1,658 to 40,460 | 1,29 | 2,49 |
| Harrison 1990 (n=6) | 6 | 16,667 | 0,421 to 64,123 | 0,57 | 1,84 |
| Harrison 1993 (n=14) | 14 | 7,143 | 0,181 to 33,868 | 1,21 | 2,44 |
| Harrison 1998 (n=13) | 13 | 84,615 | 54,553 to 98,079 | 1,13 | 2,39 |
| Adzick 2003 (n=22) | 22 | 22,727 | 7,821 to 45,370 | 1,86 | 2,71 |
| Hedrick 2004 (n=4) | 4 | 100,000 | 39,764 to 100,000 | 0,40 | 1,56 |
| Golombeck 2006 (n=79) | 79 | 48,101 | 36,714 to 59,635 | 6,47 | 3,17 |
| Longaker 1991 (n=17) | 17 | 23,529 | 6,811 to 49,899 | 1,46 | 2,56 |
| Zamora 2013 (n=7) | 7 | 28,571 | 3,669 to 70,958 | 0,65 | 1,96 |
| Total (fixed effects) | 1193 | 12,840 | 11,024 to 14,834 | 100,00 | 100,00 |
| Total (random effects) | 1193 | 16,256 | 11,165 to 22,089 | 100,00 | 100,00 |

## Test for heterogeneity

| Q | 235,8622 |
| --- | --- |
| DF | 42 |
| Significance level | P < 0,0001 |
| I^2^ (inconsistency) | 82,19% |
| 95% CI for I^2^ | 76,71 to 86,38 |

# Meta-analysis: combined open all

| Variable for studies | Study |
| --- | --- |
| Variable for total number of cases | N |
| Variable for number of positive cases | Outcome |

| Study | Sample size | Proportion (%) | 95% CI | Weight (%) | |
| --- | --- | --- | --- | --- | --- |
|  |  |  |  | Fixed | Random |
| Barthod 2013 (n=5) | 5 | 0,000 | 0,000 to 52,182 | 0,49 | 1,70 |
| Cass 2013 (n=9) | 9 | 22,222 | 2,814 to 60,009 | 0,81 | 2,13 |
| Chen 2018 (n=7) | 7 | 0,000 | 0,000 to 40,962 | 0,65 | 1,95 |
| Dahlgren 2004 (n=4) | 4 | 75,000 | 19,412 to 99,369 | 0,40 | 1,55 |
| Flake 2000 (n=15) | 15 | 0,000 | 0,000 to 21,802 | 1,29 | 2,49 |
| George 2007 (n=3) | 3 | 100,000 | 29,240 to 100,000 | 0,32 | 1,36 |
| Hedrick 2003 (n=43) | 43 | 16,279 | 6,805 to 30,701 | 3,56 | 3,01 |
| Hedrick 2005 (n=9) | 9 | 22,222 | 2,814 to 60,009 | 0,81 | 2,13 |
| Kern 2007 (n=5) | 5 | 20,000 | 0,505 to 71,642 | 0,49 | 1,70 |
| Kornacki 2017 (n=4) | 4 | 0,000 | 0,000 to 60,236 | 0,40 | 1,55 |
| Kunisaki 2007 (n=14) | 14 | 0,000 | 0,000 to 23,164 | 1,21 | 2,44 |
| Laje 2012 (n=17) | 17 | 29,412 | 10,314 to 55,958 | 1,46 | 2,56 |
| Laje 2013 (n=4) | 4 | 50,000 | 6,759 to 93,241 | 0,40 | 1,55 |
| Laje 2015 (n=13) | 13 | 0,000 | 0,000 to 24,705 | 1,13 | 2,39 |
| Lazar 2011 (n=12) | 12 | 16,667 | 2,086 to 48,414 | 1,05 | 2,34 |
| Noah 2002 (n=34) | 34 | 38,235 | 22,167 to 56,436 | 2,83 | 2,92 |
| Pellicer 2007 (n=3) | 3 | 33,333 | 0,840 to 90,570 | 0,32 | 1,36 |
| Stoffan 2012 (n=7) | 7 | 14,286 | 0,361 to 57,872 | 0,65 | 1,95 |
| Tuncay Ozgunen 2010 (n=3) | 3 | 0,000 | 0,000 to 70,760 | 0,32 | 1,36 |
| Zamora 2013 (n=26) | 26 | 30,769 | 14,326 to 51,790 | 2,18 | 2,80 |
| Bennett 2014 (n=43) | 43 | 4,651 | 0,568 to 15,811 | 3,56 | 3,01 |
| Botelho 2017 (n=45) | 45 | 4,444 | 0,543 to 15,149 | 3,72 | 3,03 |
| Bruner 1999 (n=29) | 29 | 13,793 | 3,889 to 31,664 | 2,43 | 2,85 |
| Bruner 2000 (n=4) | 4 | 0,000 | 0,000 to 60,236 | 0,40 | 1,55 |
| Farmer 2003 (n=12) | 12 | 41,667 | 15,165 to 72,333 | 1,05 | 2,34 |
| Friszer 2016 (n=3) | 3 | 0,000 | 0,000 to 70,760 | 0,32 | 1,36 |
| Johnson 2016 (n=91) | 91 | 23,077 | 14,888 to 33,087 | 7,44 | 3,22 |
| Marenco 2013 (n=4) | 4 | 0,000 | 0,000 to 60,236 | 0,40 | 1,55 |
| Moldenhauer 2015 (n=100) | 100 | 13,000 | 7,107 to 21,204 | 8,17 | 3,24 |
| Moron 2018 (n=237) | 237 | 9,705 | 6,252 to 14,206 | 19,26 | 3,35 |
| Ochsenbein-Kolble 2017 (n=30) | 30 | 10,000 | 2,112 to 26,529 | 2,51 | 2,87 |
| Sinskey 2017 (n=47) | 47 | 4,255 | 0,520 to 14,541 | 3,88 | 3,04 |
| Soni 2016 (n=88) | 88 | 4,545 | 1,252 to 11,231 | 7,20 | 3,21 |
| Zamlynski 2014 (n=46) | 46 | 21,739 | 10,948 to 36,362 | 3,80 | 3,04 |
| Flake 2000 (n=15) | 15 | 20,000 | 4,331 to 48,089 | 1,29 | 2,49 |
| Harrison 1990 (n=6) | 6 | 16,667 | 0,421 to 64,123 | 0,57 | 1,83 |
| Harrison 1993 (n=14) | 14 | 14,286 | 1,779 to 42,813 | 1,21 | 2,44 |
| Harrison 1998 (n=13) | 13 | 84,615 | 54,553 to 98,079 | 1,13 | 2,39 |
| Adzick 2003 (n=22) | 22 | 31,818 | 13,865 to 54,872 | 1,86 | 2,71 |
| Hedrick 2004 (n=4) | 4 | 100,000 | 39,764 to 100,000 | 0,40 | 1,55 |
| Golombeck 2006 (n=79) | 79 | 59,494 | 47,852 to 70,401 | 6,47 | 3,19 |
| Longaker 1991 (n=17) | 17 | 23,529 | 6,811 to 49,899 | 1,46 | 2,56 |
| Zamora 2013 (n=7) | 7 | 42,857 | 9,899 to 81,595 | 0,65 | 1,95 |
| Total (fixed effects) | 1193 | 17,274 | 15,206 to 19,499 | 100,00 | 100,00 |
| Total (random effects) | 1193 | 20,858 | 15,217 to 27,129 | 100,00 | 100,00 |

## Test for heterogeneity

| Q | 230,6951 |
| --- | --- |
| DF | 42 |
| Significance level | P < 0,0001 |
| I^2^ (inconsistency) | 81,79% |
| 95% CI for I^2^ | 76,15 to 86,10 |

# Meta-analysis: combined feto severe

| Variable for studies | Study |
| --- | --- |
| Variable for total number of cases | N |
| Variable for number of positive cases | Outcome |

| Study | Sample size | Proportion (%) | 95% CI | Weight (%) | |
| --- | --- | --- | --- | --- | --- |
|  |  |  |  | Fixed | Random |
| Aboudiab 2017 (n=18) | 18 | 5,556 | 0,141 to 27,294 | 0,20 | 0,52 |
| Baschat 2013 (n=147) | 147 | 4,762 | 1,936 to 9,565 | 1,55 | 1,27 |
| Chalouhi 2016 (n=22) | 22 | 0,000 | 0,000 to 15,437 | 0,24 | 0,59 |
| Chang 2006 (n=27) | 27 | 3,704 | 0,0937 to 18,971 | 0,29 | 0,67 |
| Chang 2016 (n=100) | 100 | 0,000 | 0,000 to 3,622 | 1,06 | 1,16 |
| Chmait 2013 (n=318) | 318 | 0,629 | 0,0763 to 2,253 | 3,35 | 1,44 |
| Chmait 2017 (n=19) | 19 | 0,000 | 0,000 to 17,647 | 0,21 | 0,54 |
| Crombleholme 2007 (n=20) | 20 | 0,000 | 0,000 to 16,843 | 0,22 | 0,56 |
| De Lia 1995 (n=26) | 26 | 0,000 | 0,000 to 13,227 | 0,28 | 0,65 |
| De Lia 1999 (n=67) | 67 | 1,493 | 0,0378 to 8,038 | 0,71 | 1,02 |
| De Lia 2009 (n=10) | 10 | 0,000 | 0,000 to 30,850 | 0,12 | 0,35 |
| Deprest 1998 (n=6) | 6 | 0,000 | 0,000 to 45,926 | 0,073 | 0,24 |
| Draga 2016 (n=37) | 37 | 2,703 | 0,0684 to 14,160 | 0,40 | 0,79 |
| Duron 2014 (n=85) | 85 | 1,176 | 0,0298 to 6,381 | 0,90 | 1,10 |
| Ek 2012 (n=) | 67 | 4,478 | 0,933 to 12,533 | 0,71 | 1,02 |
| Habli 2009 (n=152) | 152 | 8,553 | 4,633 to 14,181 | 1,61 | 1,28 |
| Has 2014 (n=85) | 85 | 0,000 | 0,000 to 4,247 | 0,90 | 1,10 |
| Hecher 2000 (n=200) | 200 | 0,000 | 0,000 to 1,828 | 2,11 | 1,35 |
| Hernandez-Andrade 2011 (n=35) | 35 | 0,000 | 0,000 to 10,003 | 0,38 | 0,77 |
| Huber 2008 (n=176) | 176 | 1,136 | 0,138 to 4,044 | 1,86 | 1,32 |
| Ishii 2014 (n=16) | 16 | 0,000 | 0,000 to 20,591 | 0,18 | 0,48 |
| Ishii 2015 (n=10) | 10 | 0,000 | 0,000 to 30,850 | 0,12 | 0,35 |
| Lanna 2017 (n=373) | 373 | 5,630 | 3,518 to 8,478 | 3,93 | 1,46 |
| Lecointre 2017 (n=200) | 200 | 0,000 | 0,000 to 1,828 | 2,11 | 1,35 |
| Malshe 2017 (n=203) | 203 | 5,911 | 3,091 to 10,098 | 2,14 | 1,35 |
| Martinez 2012 (n=500) | 500 | 0,000 | 0,000 to 0,735 | 5,26 | 1,50 |
| Middeldorp 2007 (n=100) | 100 | 0,000 | 0,000 to 3,622 | 1,06 | 1,16 |
| Miyadahira 2018 (n=67) | 67 | 1,493 | 0,0378 to 8,038 | 0,71 | 1,02 |
| Molina-Garcia 2009 (n=22) | 22 | 0,000 | 0,000 to 15,437 | 0,24 | 0,59 |
| Morris 2010 (n=164) | 164 | 0,000 | 0,000 to 2,224 | 1,73 | 1,30 |
| Mullers 2015 (n=105) | 105 | 0,000 | 0,000 to 3,452 | 1,11 | 1,17 |
| Nakata 2016 (n=6) | 6 | 16,667 | 0,421 to 64,123 | 0,073 | 0,24 |
| Nguyen 2012 (n=98) | 98 | 0,000 | 0,000 to 3,694 | 1,04 | 1,15 |
| Ozawa 2017 (n=11) | 11 | 0,000 | 0,000 to 28,491 | 0,13 | 0,37 |
| Papanna 2010 (n=48) | 48 | 2,083 | 0,0527 to 11,070 | 0,51 | 0,89 |
| Papanna 2012 (n=163) | 163 | 3,067 | 1,003 to 7,013 | 1,72 | 1,30 |
| Peeters 2014 (n=338) | 338 | 0,000 | 0,000 to 1,085 | 3,56 | 1,45 |
| Persico 2016 (n=106) | 106 | 0,000 | 0,000 to 3,420 | 1,12 | 1,18 |
| Quintero 2000 (n=92) | 92 | 0,000 | 0,000 to 3,930 | 0,98 | 1,13 |
| Quintero 2001 (n=11) | 11 | 18,182 | 2,283 to 51,776 | 0,13 | 0,37 |
| Rossi 2008 (n=266) | 266 | 0,000 | 0,000 to 1,377 | 2,80 | 1,41 |
| Ruano 2009 (n=19) | 19 | 0,000 | 0,000 to 17,647 | 0,21 | 0,54 |
| Ruegg 2018 (n=37) | 37 | 2,703 | 0,0684 to 14,160 | 0,40 | 0,79 |
| Rustico 2012 (n=150) | 150 | 8,667 | 4,696 to 14,364 | 1,59 | 1,28 |
| Said 2008 (n=10) | 10 | 0,000 | 0,000 to 30,850 | 0,12 | 0,35 |
| Senat 2004 (n=72) | 72 | 1,389 | 0,0352 to 7,497 | 0,77 | 1,05 |
| Sepulveda 2007 (n=33) | 33 | 0,000 | 0,000 to 10,576 | 0,36 | 0,74 |
| Shamshirsaz 2015 (n=55) | 55 | 3,636 | 0,443 to 12,526 | 0,59 | 0,94 |
| Slaghekke 2014 (n=274) | 274 | 0,000 | 0,000 to 1,337 | 2,89 | 1,41 |
| Taniguchi 2015 (n=3) | 3 | 0,000 | 0,000 to 70,760 | 0,042 | 0,15 |
| Tchirikov 2011 (n=80) | 80 | 0,000 | 0,000 to 4,506 | 0,85 | 1,08 |
| Teoh 2013 (n=49) | 49 | 0,000 | 0,000 to 7,252 | 0,52 | 0,90 |
| Thia 2017 (n=5) | 5 | 0,000 | 0,000 to 52,182 | 0,063 | 0,21 |
| Ville 1997 (n=132) | 132 | 0,000 | 0,000 to 2,756 | 1,40 | 1,24 |
| Ville 1998 (n=44) | 44 | 0,000 | 0,000 to 8,042 | 0,47 | 0,86 |
| Weingertner 2011 (n=100) | 100 | 0,000 | 0,000 to 3,622 | 1,06 | 1,16 |
| Wilson 2016 (n=151) | 151 | 0,000 | 0,000 to 2,413 | 1,60 | 1,28 |
| Yamamoto 2005 (n=175) | 175 | 2,286 | 0,626 to 5,749 | 1,85 | 1,32 |
| Yang 2010 (n=30) | 30 | 0,000 | 0,000 to 11,570 | 0,33 | 0,71 |
| Zaretsky 2018 (n=749) | 749 | 6,008 | 4,416 to 7,957 | 7,87 | 1,53 |
| Zhao 2016 (n=62) | 62 | 0,000 | 0,000 to 5,776 | 0,66 | 0,99 |
| Bebbington 2012 (n=146) | 146 | 0,000 | 0,000 to 2,495 | 1,54 | 1,27 |
| Berg 2014 (n=7) | 7 | 14,286 | 0,361 to 57,872 | 0,084 | 0,27 |
| Delabaere 2013 (n=30) | 30 | 3,333 | 0,0844 to 17,217 | 0,33 | 0,71 |
| Deprest 2000 (n=10) | 10 | 10,000 | 0,253 to 44,502 | 0,12 | 0,35 |
| Gallot 2003 (n=11) | 11 | 9,091 | 0,230 to 41,278 | 0,13 | 0,37 |
| Gouverneur 2009 (n=54) | 54 | 0,000 | 0,000 to 6,603 | 0,58 | 0,94 |
| Gul 2008 (n=9) | 9 | 0,000 | 0,000 to 33,627 | 0,10 | 0,32 |
| Has 2014 (n=71) | 71 | 0,000 | 0,000 to 5,063 | 0,76 | 1,04 |
| He 2010 (n=14) | 14 | 0,000 | 0,000 to 23,164 | 0,16 | 0,44 |
| Ilagan 2008 (n=27) | 27 | 11,111 | 2,353 to 29,159 | 0,29 | 0,67 |
| Jelin 2010 (n=7) | 7 | 0,000 | 0,000 to 40,962 | 0,084 | 0,27 |
| King 2017 (n=43) | 43 | 9,302 | 2,593 to 22,135 | 0,46 | 0,85 |
| Lanna 2012 (n=118) | 118 | 0,000 | 0,000 to 3,078 | 1,25 | 1,21 |
| Lee 2013 (n=98) | 98 | 0,000 | 0,000 to 3,694 | 1,04 | 1,15 |
| Lewi 2006 (n=80) | 80 | 1,250 | 0,0316 to 6,769 | 0,85 | 1,08 |
| Moise 2008 (n=9) | 9 | 11,111 | 0,281 to 48,250 | 0,10 | 0,32 |
| Nobili 2013 (n=48) | 48 | 0,000 | 0,000 to 7,397 | 0,51 | 0,89 |
| Paramasivam 2010 (n=35) | 35 | 0,000 | 0,000 to 10,003 | 0,38 | 0,77 |
| Peng 2016 (n=93) | 93 | 0,000 | 0,000 to 3,889 | 0,99 | 1,14 |
| Quintero 1996 (n=13) | 13 | 7,692 | 0,195 to 36,030 | 0,15 | 0,42 |
| Quintero 2006 (n=51) | 51 | 5,882 | 1,230 to 16,242 | 0,55 | 0,92 |
| Roman 2010 (n=60) | 60 | 0,000 | 0,000 to 5,963 | 0,64 | 0,98 |
| Schou 2018 (n=102) | 102 | 0,000 | 0,000 to 3,552 | 1,08 | 1,17 |
| Sugibayashi 2016 (n=40) | 40 | 0,000 | 0,000 to 8,810 | 0,43 | 0,82 |
| Takano 2015 (n=10) | 10 | 0,000 | 0,000 to 30,850 | 0,12 | 0,35 |
| Taylor 2002 (n=15) | 15 | 13,333 | 1,658 to 40,460 | 0,17 | 0,46 |
| Tsao 2002 (n=13) | 13 | 0,000 | 0,000 to 24,705 | 0,15 | 0,42 |
| Zhang 2018 (n=25) | 25 | 0,000 | 0,000 to 13,719 | 0,27 | 0,64 |
| Deprest 2005 (n=20) | 20 | 0,000 | 0,000 to 16,843 | 0,22 | 0,56 |
| Harrison 1998 (n=8) | 8 | 0,000 | 0,000 to 36,942 | 0,094 | 0,30 |
| Harrison 2003 (n=11) | 11 | 27,273 | 6,022 to 60,974 | 0,13 | 0,37 |
| Jani 2005 (n=24) | 24 | 0,000 | 0,000 to 14,247 | 0,26 | 0,62 |
| Jani 2006 (n=28) | 28 | 0,000 | 0,000 to 12,344 | 0,30 | 0,68 |
| Jani 2009 (n=210) | 210 | 0,000 | 0,000 to 1,741 | 2,22 | 1,36 |
| Jimenez 2017 (n=201) | 201 | 0,000 | 0,000 to 1,819 | 2,12 | 1,35 |
| Kosinski 2017 (n=28) | 28 | 0,000 | 0,000 to 12,344 | 0,30 | 0,68 |
| Manrique 2008 (n=11) | 11 | 0,000 | 0,000 to 28,491 | 0,13 | 0,37 |
| Peralta 2011 (n=8) | 8 | 0,000 | 0,000 to 36,942 | 0,094 | 0,30 |
| Persico 2017 (n=21) | 21 | 0,000 | 0,000 to 16,110 | 0,23 | 0,58 |
| Ruano 2012 (n=35) | 35 | 2,857 | 0,0723 to 14,917 | 0,38 | 0,77 |
| Ruano 2012 (n=20) | 20 | 0,000 | 0,000 to 16,843 | 0,22 | 0,56 |
| Ruano 2013 (n=17) | 17 | 0,000 | 0,000 to 19,506 | 0,19 | 0,50 |
| Arens 2017 (n=59) | 59 | 0,000 | 0,000 to 6,061 | 0,63 | 0,97 |
| Belfort 2017 (n=22) | 22 | 9,091 | 1,121 to 29,161 | 0,24 | 0,59 |
| Bruner 2000 (n=4) | 4 | 25,000 | 0,631 to 80,588 | 0,052 | 0,18 |
| Degenhardt 2014 (n=51) | 51 | 0,000 | 0,000 to 6,978 | 0,55 | 0,92 |
| Kohn 2018 (n=34) | 34 | 8,824 | 1,858 to 23,678 | 0,37 | 0,76 |
| Pedreira 2014 (n=4) | 4 | 0,000 | 0,000 to 60,236 | 0,052 | 0,18 |
| Pedreira 2016 (n=10) | 10 | 0,000 | 0,000 to 30,850 | 0,12 | 0,35 |
| Verbeek 2012 (n=19) | 19 | 0,000 | 0,000 to 17,647 | 0,21 | 0,54 |
| Ziemann 2018 (n=65) | 65 | 0,000 | 0,000 to 5,517 | 0,69 | 1,01 |
| Morris 2013 (n=16) | 16 | 0,000 | 0,000 to 20,591 | 0,18 | 0,48 |
| Ruano 2010 (n=11) | 11 | 0,000 | 0,000 to 28,491 | 0,13 | 0,37 |
| Welsh 2003 (n=13) | 13 | 0,000 | 0,000 to 24,705 | 0,15 | 0,42 |
| Cavalheiro 2011 (n=30) | 30 | 0,000 | 0,000 to 11,570 | 0,33 | 0,71 |
| Mallman 2017 (n=78) | 78 | 1,282 | 0,0325 to 6,937 | 0,83 | 1,07 |
| Golombeck 2006 (n=99) | 99 | 4,040 | 1,112 to 10,023 | 1,05 | 1,16 |
| Kohl 2006 (n=16) | 16 | 6,250 | 0,158 to 30,232 | 0,18 | 0,48 |
| Kohl 2010 (n=37) | 37 | 2,703 | 0,0684 to 14,160 | 0,40 | 0,79 |
| Nivatpumin 2016 (n=152) | 152 | 0,000 | 0,000 to 2,398 | 1,61 | 1,28 |
| Peralta 2010 (n=56) | 56 | 0,000 | 0,000 to 6,375 | 0,60 | 0,95 |
| Total (fixed effects) | 9403 | 1,398 | 1,172 to 1,654 | 100,00 | 100,00 |
| Total (random effects) | 9403 | 1,661 | 1,194 to 2,204 | 100,00 | 100,00 |

## Test for heterogeneity

| Q | 355,3749 |
| --- | --- |
| DF | 121 |
| Significance level | P < 0,0001 |
| I^2^ (inconsistency) | 65,95% |
| 95% CI for I^2^ | 58,84 to 71,83 |

# Meta-analysis: combined fetoscopic minor

| Variable for studies | Study |
| --- | --- |
| Variable for total number of cases | N |
| Variable for number of positive cases | Outcome |

| Study | Sample size | Proportion (%) | 95% CI | Weight (%) | |
| --- | --- | --- | --- | --- | --- |
|  |  |  |  | Fixed | Random |
| Aboudiab 2017 (n=18) | 18 | 0,000 | 0,000 to 18,530 | 0,20 | 0,64 |
| Baschat 2013 (n=147) | 147 | 6,803 | 3,310 to 12,155 | 1,55 | 1,10 |
| Chalouhi 2016 (n=22) | 22 | 4,545 | 0,115 to 22,844 | 0,24 | 0,70 |
| Chang 2006 (n=27) | 27 | 7,407 | 0,910 to 24,290 | 0,29 | 0,76 |
| Chang 2016 (n=100) | 100 | 3,000 | 0,623 to 8,518 | 1,06 | 1,05 |
| Chmait 2013 (n=318) | 318 | 1,887 | 0,695 to 4,061 | 3,35 | 1,17 |
| Chmait 2017 (n=19) | 19 | 10,526 | 1,301 to 33,138 | 0,21 | 0,66 |
| Crombleholme 2007 (n=20) | 20 | 10,000 | 1,235 to 31,698 | 0,22 | 0,68 |
| De Lia 1995 (n=26) | 26 | 26,923 | 11,573 to 47,787 | 0,28 | 0,75 |
| De Lia 1999 (n=67) | 67 | 0,000 | 0,000 to 5,357 | 0,71 | 0,98 |
| De Lia 2009 (n=10) | 10 | 10,000 | 0,253 to 44,502 | 0,12 | 0,48 |
| Deprest 1998 (n=6) | 6 | 0,000 | 0,000 to 45,926 | 0,073 | 0,35 |
| Draga 2016 (n=37) | 37 | 0,000 | 0,000 to 9,489 | 0,40 | 0,85 |
| Duron 2014 (n=85) | 85 | 9,412 | 4,152 to 17,705 | 0,90 | 1,03 |
| Ek 2012 (n=) | 67 | 0,000 | 0,000 to 5,357 | 0,71 | 0,98 |
| Habli 2009 (n=152) | 152 | 3,289 | 1,077 to 7,509 | 1,61 | 1,11 |
| Has 2014 (n=85) | 85 | 2,353 | 0,286 to 8,242 | 0,90 | 1,03 |
| Hecher 2000 (n=200) | 200 | 0,000 | 0,000 to 1,828 | 2,11 | 1,14 |
| Hernandez-Andrade 2011 (n=35) | 35 | 34,286 | 19,132 to 52,211 | 0,38 | 0,83 |
| Huber 2008 (n=176) | 176 | 0,568 | 0,0144 to 3,125 | 1,86 | 1,12 |
| Ishii 2014 (n=16) | 16 | 0,000 | 0,000 to 20,591 | 0,18 | 0,61 |
| Ishii 2015 (n=10) | 10 | 0,000 | 0,000 to 30,850 | 0,12 | 0,48 |
| Lanna 2017 (n=373) | 373 | 0,000 | 0,000 to 0,984 | 3,93 | 1,18 |
| Lecointre 2017 (n=200) | 200 | 0,000 | 0,000 to 1,828 | 2,11 | 1,14 |
| Malshe 2017 (n=203) | 203 | 6,404 | 3,454 to 10,702 | 2,14 | 1,14 |
| Martinez 2012 (n=500) | 500 | 0,000 | 0,000 to 0,735 | 5,26 | 1,19 |
| Middeldorp 2007 (n=100) | 100 | 4,000 | 1,100 to 9,926 | 1,06 | 1,05 |
| Miyadahira 2018 (n=67) | 67 | 0,000 | 0,000 to 5,357 | 0,71 | 0,98 |
| Molina-Garcia 2009 (n=22) | 22 | 0,000 | 0,000 to 15,437 | 0,24 | 0,70 |
| Morris 2010 (n=164) | 164 | 0,000 | 0,000 to 2,224 | 1,73 | 1,12 |
| Mullers 2015 (n=105) | 105 | 3,810 | 1,048 to 9,467 | 1,11 | 1,06 |
| Nakata 2016 (n=6) | 6 | 0,000 | 0,000 to 45,926 | 0,073 | 0,35 |
| Nguyen 2012 (n=98) | 98 | 1,020 | 0,0258 to 5,554 | 1,04 | 1,05 |
| Ozawa 2017 (n=11) | 11 | 0,000 | 0,000 to 28,491 | 0,13 | 0,50 |
| Papanna 2010 (n=48) | 48 | 4,167 | 0,509 to 14,254 | 0,51 | 0,91 |
| Papanna 2012 (n=163) | 163 | 3,067 | 1,003 to 7,013 | 1,72 | 1,12 |
| Peeters 2014 (n=338) | 338 | 7,692 | 5,086 to 11,068 | 3,56 | 1,17 |
| Persico 2016 (n=106) | 106 | 1,887 | 0,229 to 6,650 | 1,12 | 1,06 |
| Quintero 2000 (n=92) | 92 | 4,348 | 1,197 to 10,759 | 0,98 | 1,04 |
| Quintero 2001 (n=11) | 11 | 0,000 | 0,000 to 28,491 | 0,13 | 0,50 |
| Rossi 2008 (n=266) | 266 | 10,902 | 7,424 to 15,281 | 2,80 | 1,16 |
| Ruano 2009 (n=19) | 19 | 0,000 | 0,000 to 17,647 | 0,21 | 0,66 |
| Ruegg 2018 (n=37) | 37 | 37,838 | 22,458 to 55,243 | 0,40 | 0,85 |
| Rustico 2012 (n=150) | 150 | 4,000 | 1,482 to 8,503 | 1,59 | 1,11 |
| Said 2008 (n=10) | 10 | 0,000 | 0,000 to 30,850 | 0,12 | 0,48 |
| Senat 2004 (n=72) | 72 | 0,000 | 0,000 to 4,994 | 0,77 | 1,00 |
| Sepulveda 2007 (n=33) | 33 | 9,091 | 1,915 to 24,332 | 0,36 | 0,82 |
| Shamshirsaz 2015 (n=55) | 55 | 12,727 | 5,274 to 24,480 | 0,59 | 0,94 |
| Slaghekke 2014 (n=274) | 274 | 4,380 | 2,283 to 7,525 | 2,89 | 1,16 |
| Taniguchi 2015 (n=3) | 3 | 33,333 | 0,840 to 90,570 | 0,042 | 0,23 |
| Tchirikov 2011 (n=80) | 80 | 0,000 | 0,000 to 4,506 | 0,85 | 1,02 |
| Teoh 2013 (n=49) | 49 | 0,000 | 0,000 to 7,252 | 0,52 | 0,92 |
| Thia 2017 (n=5) | 5 | 20,000 | 0,505 to 71,642 | 0,063 | 0,32 |
| Ville 1997 (n=132) | 132 | 4,545 | 1,686 to 9,631 | 1,40 | 1,09 |
| Ville 1998 (n=44) | 44 | 11,364 | 3,794 to 24,558 | 0,47 | 0,89 |
| Weingertner 2011 (n=100) | 100 | 0,000 | 0,000 to 3,622 | 1,06 | 1,05 |
| Wilson 2016 (n=151) | 151 | 0,662 | 0,0168 to 3,634 | 1,60 | 1,11 |
| Yamamoto 2005 (n=175) | 175 | 10,286 | 6,211 to 15,768 | 1,85 | 1,12 |
| Yang 2010 (n=30) | 30 | 10,000 | 2,112 to 26,529 | 0,33 | 0,79 |
| Zaretsky 2018 (n=749) | 749 | 0,000 | 0,000 to 0,491 | 7,87 | 1,21 |
| Zhao 2016 (n=62) | 62 | 19,355 | 10,421 to 31,369 | 0,66 | 0,97 |
| Bebbington 2012 (n=146) | 146 | 0,000 | 0,000 to 2,495 | 1,54 | 1,10 |
| Berg 2014 (n=7) | 7 | 0,000 | 0,000 to 40,962 | 0,084 | 0,39 |
| Delabaere 2013 (n=30) | 30 | 3,333 | 0,0844 to 17,217 | 0,33 | 0,79 |
| Deprest 2000 (n=10) | 10 | 0,000 | 0,000 to 30,850 | 0,12 | 0,48 |
| Gallot 2003 (n=11) | 11 | 9,091 | 0,230 to 41,278 | 0,13 | 0,50 |
| Gouverneur 2009 (n=54) | 54 | 0,000 | 0,000 to 6,603 | 0,58 | 0,94 |
| Gul 2008 (n=9) | 9 | 0,000 | 0,000 to 33,627 | 0,10 | 0,45 |
| Has 2014 (n=71) | 71 | 1,408 | 0,0357 to 7,599 | 0,76 | 0,99 |
| He 2010 (n=14) | 14 | 7,143 | 0,181 to 33,868 | 0,16 | 0,57 |
| Ilagan 2008 (n=27) | 27 | 7,407 | 0,910 to 24,290 | 0,29 | 0,76 |
| Jelin 2010 (n=7) | 7 | 14,286 | 0,361 to 57,872 | 0,084 | 0,39 |
| King 2017 (n=43) | 43 | 2,326 | 0,0589 to 12,289 | 0,46 | 0,88 |
| Lanna 2012 (n=118) | 118 | 0,847 | 0,0215 to 4,631 | 1,25 | 1,08 |
| Lee 2013 (n=98) | 98 | 2,041 | 0,248 to 7,178 | 1,04 | 1,05 |
| Lewi 2006 (n=80) | 80 | 0,000 | 0,000 to 4,506 | 0,85 | 1,02 |
| Moise 2008 (n=9) | 9 | 0,000 | 0,000 to 33,627 | 0,10 | 0,45 |
| Nobili 2013 (n=48) | 48 | 0,000 | 0,000 to 7,397 | 0,51 | 0,91 |
| Paramasivam 2010 (n=35) | 35 | 0,000 | 0,000 to 10,003 | 0,38 | 0,83 |
| Peng 2016 (n=93) | 93 | 10,753 | 5,278 to 18,887 | 0,99 | 1,04 |
| Quintero 1996 (n=13) | 13 | 30,769 | 9,092 to 61,426 | 0,15 | 0,55 |
| Quintero 2006 (n=51) | 51 | 5,882 | 1,230 to 16,242 | 0,55 | 0,92 |
| Roman 2010 (n=60) | 60 | 0,000 | 0,000 to 5,963 | 0,64 | 0,96 |
| Schou 2018 (n=102) | 102 | 0,980 | 0,0248 to 5,342 | 1,08 | 1,06 |
| Sugibayashi 2016 (n=40) | 40 | 2,500 | 0,0633 to 13,159 | 0,43 | 0,87 |
| Takano 2015 (n=10) | 10 | 0,000 | 0,000 to 30,850 | 0,12 | 0,48 |
| Taylor 2002 (n=15) | 15 | 6,667 | 0,169 to 31,948 | 0,17 | 0,59 |
| Tsao 2002 (n=13) | 13 | 7,692 | 0,195 to 36,030 | 0,15 | 0,55 |
| Zhang 2018 (n=25) | 25 | 0,000 | 0,000 to 13,719 | 0,27 | 0,74 |
| Deprest 2005 (n=20) | 20 | 0,000 | 0,000 to 16,843 | 0,22 | 0,68 |
| Harrison 1998 (n=8) | 8 | 37,500 | 8,523 to 75,514 | 0,094 | 0,42 |
| Harrison 2003 (n=11) | 11 | 36,364 | 10,926 to 69,210 | 0,13 | 0,50 |
| Jani 2005 (n=24) | 24 | 0,000 | 0,000 to 14,247 | 0,26 | 0,73 |
| Jani 2006 (n=28) | 28 | 0,000 | 0,000 to 12,344 | 0,30 | 0,77 |
| Jani 2009 (n=210) | 210 | 3,333 | 1,350 to 6,747 | 2,22 | 1,14 |
| Jimenez 2017 (n=201) | 201 | 0,000 | 0,000 to 1,819 | 2,12 | 1,14 |
| Kosinski 2017 (n=28) | 28 | 0,000 | 0,000 to 12,344 | 0,30 | 0,77 |
| Manrique 2008 (n=11) | 11 | 0,000 | 0,000 to 28,491 | 0,13 | 0,50 |
| Peralta 2011 (n=8) | 8 | 0,000 | 0,000 to 36,942 | 0,094 | 0,42 |
| Persico 2017 (n=21) | 21 | 0,000 | 0,000 to 16,110 | 0,23 | 0,69 |
| Ruano 2012 (n=35) | 35 | 0,000 | 0,000 to 10,003 | 0,38 | 0,83 |
| Ruano 2012 (n=20) | 20 | 5,000 | 0,127 to 24,873 | 0,22 | 0,68 |
| Ruano 2013 (n=17) | 17 | 5,882 | 0,149 to 28,689 | 0,19 | 0,63 |
| Arens 2017 (n=59) | 59 | 3,390 | 0,413 to 11,715 | 0,63 | 0,96 |
| Belfort 2017 (n=22) | 22 | 9,091 | 1,121 to 29,161 | 0,24 | 0,70 |
| Bruner 2000 (n=4) | 4 | 25,000 | 0,631 to 80,588 | 0,052 | 0,28 |
| Degenhardt 2014 (n=51) | 51 | 7,843 | 2,178 to 18,881 | 0,55 | 0,92 |
| Kohn 2018 (n=34) | 34 | 2,941 | 0,0744 to 15,327 | 0,37 | 0,82 |
| Pedreira 2014 (n=4) | 4 | 50,000 | 6,759 to 93,241 | 0,052 | 0,28 |
| Pedreira 2016 (n=10) | 10 | 0,000 | 0,000 to 30,850 | 0,12 | 0,48 |
| Verbeek 2012 (n=19) | 19 | 31,579 | 12,576 to 56,550 | 0,21 | 0,66 |
| Ziemann 2018 (n=65) | 65 | 0,000 | 0,000 to 5,517 | 0,69 | 0,98 |
| Morris 2013 (n=16) | 16 | 18,750 | 4,047 to 45,646 | 0,18 | 0,61 |
| Ruano 2010 (n=11) | 11 | 0,000 | 0,000 to 28,491 | 0,13 | 0,50 |
| Welsh 2003 (n=13) | 13 | 7,692 | 0,195 to 36,030 | 0,15 | 0,55 |
| Cavalheiro 2011 (n=30) | 30 | 0,000 | 0,000 to 11,570 | 0,33 | 0,79 |
| Mallman 2017 (n=78) | 78 | 7,692 | 2,875 to 15,995 | 0,83 | 1,01 |
| Golombeck 2006 (n=99) | 99 | 20,202 | 12,798 to 29,461 | 1,05 | 1,05 |
| Kohl 2006 (n=16) | 16 | 18,750 | 4,047 to 45,646 | 0,18 | 0,61 |
| Kohl 2010 (n=37) | 37 | 10,811 | 3,025 to 25,418 | 0,40 | 0,85 |
| Nivatpumin 2016 (n=152) | 152 | 4,605 | 1,871 to 9,258 | 1,61 | 1,11 |
| Peralta 2010 (n=56) | 56 | 3,571 | 0,435 to 12,313 | 0,60 | 0,95 |
| Total (fixed effects) | 9403 | 2,662 | 2,348 to 3,006 | 100,00 | 100,00 |
| Total (random effects) | 9403 | 4,328 | 3,328 to 5,452 | 100,00 | 100,00 |

## Test for heterogeneity

| Q | 656,5652 |
| --- | --- |
| DF | 121 |
| Significance level | P < 0,0001 |
| I^2^ (inconsistency) | 81,57% |
| 95% CI for I^2^ | 78,37 to 84,30 |

# Meta-analysis: combined feto all

| Variable for studies | Study |
| --- | --- |
| Variable for total number of cases | N |
| Variable for number of positive cases | Outcome |

| Study | Sample size | Proportion (%) | 95% CI | Weight (%) | |
| --- | --- | --- | --- | --- | --- |
|  |  |  |  | Fixed | Random |
| Aboudiab 2017 (n=18) | 18 | 5,556 | 0,141 to 27,294 | 0,20 | 0,65 |
| Baschat 2013 (n=147) | 147 | 11,565 | 6,883 to 17,870 | 1,55 | 1,09 |
| Chalouhi 2016 (n=22) | 22 | 4,545 | 0,115 to 22,844 | 0,24 | 0,71 |
| Chang 2006 (n=27) | 27 | 11,111 | 2,353 to 29,159 | 0,29 | 0,77 |
| Chang 2016 (n=100) | 100 | 3,000 | 0,623 to 8,518 | 1,06 | 1,04 |
| Chmait 2013 (n=318) | 318 | 2,516 | 1,092 to 4,897 | 3,35 | 1,16 |
| Chmait 2017 (n=19) | 19 | 10,526 | 1,301 to 33,138 | 0,21 | 0,67 |
| Crombleholme 2007 (n=20) | 20 | 10,000 | 1,235 to 31,698 | 0,22 | 0,68 |
| De Lia 1995 (n=26) | 26 | 26,923 | 11,573 to 47,787 | 0,28 | 0,76 |
| De Lia 1999 (n=67) | 67 | 1,493 | 0,0378 to 8,038 | 0,71 | 0,98 |
| De Lia 2009 (n=10) | 10 | 10,000 | 0,253 to 44,502 | 0,12 | 0,49 |
| Deprest 1998 (n=6) | 6 | 0,000 | 0,000 to 45,926 | 0,073 | 0,36 |
| Draga 2016 (n=37) | 37 | 2,703 | 0,0684 to 14,160 | 0,40 | 0,85 |
| Duron 2014 (n=85) | 85 | 10,588 | 4,957 to 19,150 | 0,90 | 1,02 |
| Ek 2012 (n=) | 67 | 4,478 | 0,933 to 12,533 | 0,71 | 0,98 |
| Habli 2009 (n=152) | 152 | 11,842 | 7,172 to 18,069 | 1,61 | 1,10 |
| Has 2014 (n=85) | 85 | 2,353 | 0,286 to 8,242 | 0,90 | 1,02 |
| Hecher 2000 (n=200) | 200 | 0,000 | 0,000 to 1,828 | 2,11 | 1,12 |
| Hernandez-Andrade 2011 (n=35) | 35 | 34,286 | 19,132 to 52,211 | 0,38 | 0,83 |
| Huber 2008 (n=176) | 176 | 1,705 | 0,353 to 4,900 | 1,86 | 1,11 |
| Ishii 2014 (n=16) | 16 | 0,000 | 0,000 to 20,591 | 0,18 | 0,62 |
| Ishii 2015 (n=10) | 10 | 0,000 | 0,000 to 30,850 | 0,12 | 0,49 |
| Lanna 2017 (n=373) | 373 | 5,630 | 3,518 to 8,478 | 3,93 | 1,16 |
| Lecointre 2017 (n=200) | 200 | 0,000 | 0,000 to 1,828 | 2,11 | 1,12 |
| Malshe 2017 (n=203) | 203 | 12,315 | 8,131 to 17,641 | 2,14 | 1,12 |
| Martinez 2012 (n=500) | 500 | 0,000 | 0,000 to 0,735 | 5,26 | 1,18 |
| Middeldorp 2007 (n=100) | 100 | 4,000 | 1,100 to 9,926 | 1,06 | 1,04 |
| Miyadahira 2018 (n=67) | 67 | 1,493 | 0,0378 to 8,038 | 0,71 | 0,98 |
| Molina-Garcia 2009 (n=22) | 22 | 0,000 | 0,000 to 15,437 | 0,24 | 0,71 |
| Morris 2010 (n=164) | 164 | 0,000 | 0,000 to 2,224 | 1,73 | 1,10 |
| Mullers 2015 (n=105) | 105 | 3,810 | 1,048 to 9,467 | 1,11 | 1,05 |
| Nakata 2016 (n=6) | 6 | 16,667 | 0,421 to 64,123 | 0,073 | 0,36 |
| Nguyen 2012 (n=98) | 98 | 1,020 | 0,0258 to 5,554 | 1,04 | 1,04 |
| Ozawa 2017 (n=11) | 11 | 0,000 | 0,000 to 28,491 | 0,13 | 0,51 |
| Papanna 2010 (n=48) | 48 | 6,250 | 1,308 to 17,196 | 0,51 | 0,91 |
| Papanna 2012 (n=163) | 163 | 6,135 | 2,981 to 10,993 | 1,72 | 1,10 |
| Peeters 2014 (n=338) | 338 | 7,692 | 5,086 to 11,068 | 3,56 | 1,16 |
| Persico 2016 (n=106) | 106 | 1,887 | 0,229 to 6,650 | 1,12 | 1,05 |
| Quintero 2000 (n=92) | 92 | 4,348 | 1,197 to 10,759 | 0,98 | 1,03 |
| Quintero 2001 (n=11) | 11 | 18,182 | 2,283 to 51,776 | 0,13 | 0,51 |
| Rossi 2008 (n=266) | 266 | 10,902 | 7,424 to 15,281 | 2,80 | 1,14 |
| Ruano 2009 (n=19) | 19 | 0,000 | 0,000 to 17,647 | 0,21 | 0,67 |
| Ruegg 2018 (n=37) | 37 | 40,541 | 24,754 to 57,900 | 0,40 | 0,85 |
| Rustico 2012 (n=150) | 150 | 12,667 | 7,801 to 19,072 | 1,59 | 1,10 |
| Said 2008 (n=10) | 10 | 0,000 | 0,000 to 30,850 | 0,12 | 0,49 |
| Senat 2004 (n=72) | 72 | 1,389 | 0,0352 to 7,497 | 0,77 | 0,99 |
| Sepulveda 2007 (n=33) | 33 | 9,091 | 1,915 to 24,332 | 0,36 | 0,82 |
| Shamshirsaz 2015 (n=55) | 55 | 16,364 | 7,766 to 28,803 | 0,59 | 0,94 |
| Slaghekke 2014 (n=274) | 274 | 4,380 | 2,283 to 7,525 | 2,89 | 1,15 |
| Taniguchi 2015 (n=3) | 3 | 33,333 | 0,840 to 90,570 | 0,042 | 0,24 |
| Tchirikov 2011 (n=80) | 80 | 0,000 | 0,000 to 4,506 | 0,85 | 1,01 |
| Teoh 2013 (n=49) | 49 | 0,000 | 0,000 to 7,252 | 0,52 | 0,91 |
| Thia 2017 (n=5) | 5 | 20,000 | 0,505 to 71,642 | 0,063 | 0,33 |
| Ville 1997 (n=132) | 132 | 4,545 | 1,686 to 9,631 | 1,40 | 1,08 |
| Ville 1998 (n=44) | 44 | 11,364 | 3,794 to 24,558 | 0,47 | 0,89 |
| Weingertner 2011 (n=100) | 100 | 0,000 | 0,000 to 3,622 | 1,06 | 1,04 |
| Wilson 2016 (n=151) | 151 | 0,662 | 0,0168 to 3,634 | 1,60 | 1,10 |
| Yamamoto 2005 (n=175) | 175 | 12,571 | 8,049 to 18,413 | 1,85 | 1,11 |
| Yang 2010 (n=30) | 30 | 10,000 | 2,112 to 26,529 | 0,33 | 0,79 |
| Zaretsky 2018 (n=749) | 749 | 6,008 | 4,416 to 7,957 | 7,87 | 1,19 |
| Zhao 2016 (n=62) | 62 | 19,355 | 10,421 to 31,369 | 0,66 | 0,96 |
| Bebbington 2012 (n=146) | 146 | 0,000 | 0,000 to 2,495 | 1,54 | 1,09 |
| Berg 2014 (n=7) | 7 | 14,286 | 0,361 to 57,872 | 0,084 | 0,40 |
| Delabaere 2013 (n=30) | 30 | 6,667 | 0,818 to 22,074 | 0,33 | 0,79 |
| Deprest 2000 (n=10) | 10 | 10,000 | 0,253 to 44,502 | 0,12 | 0,49 |
| Gallot 2003 (n=11) | 11 | 18,182 | 2,283 to 51,776 | 0,13 | 0,51 |
| Gouverneur 2009 (n=54) | 54 | 0,000 | 0,000 to 6,603 | 0,58 | 0,94 |
| Gul 2008 (n=9) | 9 | 0,000 | 0,000 to 33,627 | 0,10 | 0,46 |
| Has 2014 (n=71) | 71 | 1,408 | 0,0357 to 7,599 | 0,76 | 0,99 |
| He 2010 (n=14) | 14 | 7,143 | 0,181 to 33,868 | 0,16 | 0,58 |
| Ilagan 2008 (n=27) | 27 | 18,519 | 6,300 to 38,083 | 0,29 | 0,77 |
| Jelin 2010 (n=7) | 7 | 14,286 | 0,361 to 57,872 | 0,084 | 0,40 |
| King 2017 (n=43) | 43 | 11,628 | 3,885 to 25,083 | 0,46 | 0,89 |
| Lanna 2012 (n=118) | 118 | 0,847 | 0,0215 to 4,631 | 1,25 | 1,07 |
| Lee 2013 (n=98) | 98 | 2,041 | 0,248 to 7,178 | 1,04 | 1,04 |
| Lewi 2006 (n=80) | 80 | 1,250 | 0,0316 to 6,769 | 0,85 | 1,01 |
| Moise 2008 (n=9) | 9 | 11,111 | 0,281 to 48,250 | 0,10 | 0,46 |
| Nobili 2013 (n=48) | 48 | 0,000 | 0,000 to 7,397 | 0,51 | 0,91 |
| Paramasivam 2010 (n=35) | 35 | 0,000 | 0,000 to 10,003 | 0,38 | 0,83 |
| Peng 2016 (n=93) | 93 | 10,753 | 5,278 to 18,887 | 0,99 | 1,03 |
| Quintero 1996 (n=13) | 13 | 38,462 | 13,858 to 68,422 | 0,15 | 0,56 |
| Quintero 2006 (n=51) | 51 | 11,765 | 4,442 to 23,868 | 0,55 | 0,92 |
| Roman 2010 (n=60) | 60 | 0,000 | 0,000 to 5,963 | 0,64 | 0,96 |
| Schou 2018 (n=102) | 102 | 0,980 | 0,0248 to 5,342 | 1,08 | 1,05 |
| Sugibayashi 2016 (n=40) | 40 | 2,500 | 0,0633 to 13,159 | 0,43 | 0,87 |
| Takano 2015 (n=10) | 10 | 0,000 | 0,000 to 30,850 | 0,12 | 0,49 |
| Taylor 2002 (n=15) | 15 | 20,000 | 4,331 to 48,089 | 0,17 | 0,60 |
| Tsao 2002 (n=13) | 13 | 7,692 | 0,195 to 36,030 | 0,15 | 0,56 |
| Zhang 2018 (n=25) | 25 | 0,000 | 0,000 to 13,719 | 0,27 | 0,75 |
| Deprest 2005 (n=20) | 20 | 0,000 | 0,000 to 16,843 | 0,22 | 0,68 |
| Harrison 1998 (n=8) | 8 | 37,500 | 8,523 to 75,514 | 0,094 | 0,43 |
| Harrison 2003 (n=11) | 11 | 63,636 | 30,790 to 89,074 | 0,13 | 0,51 |
| Jani 2005 (n=24) | 24 | 0,000 | 0,000 to 14,247 | 0,26 | 0,73 |
| Jani 2006 (n=28) | 28 | 0,000 | 0,000 to 12,344 | 0,30 | 0,78 |
| Jani 2009 (n=210) | 210 | 3,333 | 1,350 to 6,747 | 2,22 | 1,13 |
| Jimenez 2017 (n=201) | 201 | 0,000 | 0,000 to 1,819 | 2,12 | 1,12 |
| Kosinski 2017 (n=28) | 28 | 0,000 | 0,000 to 12,344 | 0,30 | 0,78 |
| Manrique 2008 (n=11) | 11 | 0,000 | 0,000 to 28,491 | 0,13 | 0,51 |
| Peralta 2011 (n=8) | 8 | 0,000 | 0,000 to 36,942 | 0,094 | 0,43 |
| Persico 2017 (n=21) | 21 | 0,000 | 0,000 to 16,110 | 0,23 | 0,70 |
| Ruano 2012 (n=35) | 35 | 2,857 | 0,0723 to 14,917 | 0,38 | 0,83 |
| Ruano 2012 (n=20) | 20 | 5,000 | 0,127 to 24,873 | 0,22 | 0,68 |
| Ruano 2013 (n=17) | 17 | 5,882 | 0,149 to 28,689 | 0,19 | 0,64 |
| Arens 2017 (n=59) | 59 | 3,390 | 0,413 to 11,715 | 0,63 | 0,95 |
| Belfort 2017 (n=22) | 22 | 18,182 | 5,187 to 40,285 | 0,24 | 0,71 |
| Bruner 2000 (n=4) | 4 | 50,000 | 6,759 to 93,241 | 0,052 | 0,28 |
| Degenhardt 2014 (n=51) | 51 | 7,843 | 2,178 to 18,881 | 0,55 | 0,92 |
| Kohn 2018 (n=34) | 34 | 11,765 | 3,300 to 27,450 | 0,37 | 0,83 |
| Pedreira 2014 (n=4) | 4 | 50,000 | 6,759 to 93,241 | 0,052 | 0,28 |
| Pedreira 2016 (n=10) | 10 | 0,000 | 0,000 to 30,850 | 0,12 | 0,49 |
| Verbeek 2012 (n=19) | 19 | 31,579 | 12,576 to 56,550 | 0,21 | 0,67 |
| Ziemann 2018 (n=65) | 65 | 0,000 | 0,000 to 5,517 | 0,69 | 0,97 |
| Morris 2013 (n=16) | 16 | 18,750 | 4,047 to 45,646 | 0,18 | 0,62 |
| Ruano 2010 (n=11) | 11 | 0,000 | 0,000 to 28,491 | 0,13 | 0,51 |
| Welsh 2003 (n=13) | 13 | 7,692 | 0,195 to 36,030 | 0,15 | 0,56 |
| Cavalheiro 2011 (n=30) | 30 | 0,000 | 0,000 to 11,570 | 0,33 | 0,79 |
| Mallman 2017 (n=78) | 78 | 8,974 | 3,685 to 17,620 | 0,83 | 1,01 |
| Golombeck 2006 (n=99) | 99 | 24,242 | 16,192 to 33,890 | 1,05 | 1,04 |
| Kohl 2006 (n=16) | 16 | 25,000 | 7,266 to 52,377 | 0,18 | 0,62 |
| Kohl 2010 (n=37) | 37 | 13,514 | 4,537 to 28,775 | 0,40 | 0,85 |
| Nivatpumin 2016 (n=152) | 152 | 4,605 | 1,871 to 9,258 | 1,61 | 1,10 |
| Peralta 2010 (n=56) | 56 | 3,571 | 0,435 to 12,313 | 0,60 | 0,94 |
| Total (fixed effects) | 9403 | 4,544 | 4,134 to 4,982 | 100,00 | 100,00 |
| Total (random effects) | 9403 | 6,147 | 4,927 to 7,491 | 100,00 | 100,00 |

## Test for heterogeneity

| Q | 690,0093 |
| --- | --- |
| DF | 121 |
| Significance level | P < 0,0001 |
| I^2^ (inconsistency) | 82,46% |
| 95% CI for I^2^ | 79,47 to 85,02 |

# Meta-analysis: combined_open_exit_severe

| Variable for studies | Study |
| --- | --- |
| Variable for total number of cases | N |
| Variable for number of positive cases | Outcome |

| Study | Sample size | Proportion (%) | 95% CI | Weight (%) | |
| --- | --- | --- | --- | --- | --- |
|  |  |  |  | Fixed | Random |
| Barthod 2013 (n=5) | 5 | 0,000 | 0,000 to 52,182 | 2,33 | 2,33 |
| Cass 2013 (n=9) | 9 | 0,000 | 0,000 to 33,627 | 3,89 | 3,89 |
| Chen 2018 (n=7) | 7 | 0,000 | 0,000 to 40,962 | 3,11 | 3,11 |
| Dahlgren 2004 (n=4) | 4 | 0,000 | 0,000 to 60,236 | 1,95 | 1,95 |
| Flake 2000 (n=15) | 15 | 0,000 | 0,000 to 21,802 | 6,23 | 6,23 |
| George 2007 (n=3) | 3 | 0,000 | 0,000 to 70,760 | 1,56 | 1,56 |
| Hedrick 2003 (n=43) | 43 | 2,326 | 0,0589 to 12,289 | 17,12 | 17,12 |
| Hedrick 2005 (n=9) | 9 | 0,000 | 0,000 to 33,627 | 3,89 | 3,89 |
| Kern 2007 (n=5) | 5 | 0,000 | 0,000 to 52,182 | 2,33 | 2,33 |
| Kornacki 2017 (n=4) | 4 | 0,000 | 0,000 to 60,236 | 1,95 | 1,95 |
| Kunisaki 2007 (n=14) | 14 | 0,000 | 0,000 to 23,164 | 5,84 | 5,84 |
| Laje 2012 (n=17) | 17 | 5,882 | 0,149 to 28,689 | 7,00 | 7,00 |
| Laje 2013 (n=4) | 4 | 0,000 | 0,000 to 60,236 | 1,95 | 1,95 |
| Laje 2015 (n=13) | 13 | 0,000 | 0,000 to 24,705 | 5,45 | 5,45 |
| Lazar 2011 (n=12) | 12 | 0,000 | 0,000 to 26,465 | 5,06 | 5,06 |
| Noah 2002 (n=34) | 34 | 0,000 | 0,000 to 10,282 | 13,62 | 13,62 |
| Pellicer 2007 (n=3) | 3 | 33,333 | 0,840 to 90,570 | 1,56 | 1,56 |
| Stoffan 2012 (n=7) | 7 | 14,286 | 0,361 to 57,872 | 3,11 | 3,11 |
| Tuncay Ozgunen 2010 (n=3) | 3 | 0,000 | 0,000 to 70,760 | 1,56 | 1,56 |
| Zamora 2013 (n=26) | 26 | 3,846 | 0,0973 to 19,637 | 10,51 | 10,51 |
| Total (fixed effects) | 237 | 3,618 | 1,692 to 6,691 | 100,00 | 100,00 |
| Total (random effects) | 237 | 3,618 | 1,686 to 6,241 | 100,00 | 100,00 |

## Test for heterogeneity

| Q | 9,0902 |
| --- | --- |
| DF | 19 |
| Significance level | P = 0,9720 |
| I^2^ (inconsistency) | 0,00% |
| 95% CI for I^2^ | 0,00 to 0,00 |

# Meta-analysis: combined open exit minor

| Variable for studies | Study |
| --- | --- |
| Variable for total number of cases | N |
| Variable for number of positive cases | Outcome |

| Study | Sample size | Proportion (%) | 95% CI | Weight (%) | |
| --- | --- | --- | --- | --- | --- |
|  |  |  |  | Fixed | Random |
| Barthod 2013 (n=5) | 5 | 0,000 | 0,000 to 52,182 | 2,33 | 4,10 |
| Cass 2013 (n=9) | 9 | 22,222 | 2,814 to 60,009 | 3,89 | 5,17 |
| Chen 2018 (n=7) | 7 | 0,000 | 0,000 to 40,962 | 3,11 | 4,71 |
| Dahlgren 2004 (n=4) | 4 | 75,000 | 19,412 to 99,369 | 1,95 | 3,71 |
| Flake 2000 (n=15) | 15 | 0,000 | 0,000 to 21,802 | 6,23 | 6,05 |
| George 2007 (n=3) | 3 | 100,000 | 29,240 to 100,000 | 1,56 | 3,26 |
| Hedrick 2003 (n=43) | 43 | 13,953 | 5,298 to 27,932 | 17,12 | 7,40 |
| Hedrick 2005 (n=9) | 9 | 22,222 | 2,814 to 60,009 | 3,89 | 5,17 |
| Kern 2007 (n=5) | 5 | 20,000 | 0,505 to 71,642 | 2,33 | 4,10 |
| Kornacki 2017 (n=4) | 4 | 0,000 | 0,000 to 60,236 | 1,95 | 3,71 |
| Kunisaki 2007 (n=14) | 14 | 0,000 | 0,000 to 23,164 | 5,84 | 5,94 |
| Laje 2012 (n=17) | 17 | 23,529 | 6,811 to 49,899 | 7,00 | 6,25 |
| Laje 2013 (n=4) | 4 | 50,000 | 6,759 to 93,241 | 1,95 | 3,71 |
| Laje 2015 (n=13) | 13 | 0,000 | 0,000 to 24,705 | 5,45 | 5,81 |
| Lazar 2011 (n=12) | 12 | 16,667 | 2,086 to 48,414 | 5,06 | 5,68 |
| Noah 2002 (n=34) | 34 | 38,235 | 22,167 to 56,436 | 13,62 | 7,16 |
| Pellicer 2007 (n=3) | 3 | 0,000 | 0,000 to 70,760 | 1,56 | 3,26 |
| Stoffan 2012 (n=7) | 7 | 0,000 | 0,000 to 40,962 | 3,11 | 4,71 |
| Tuncay Ozgunen 2010 (n=3) | 3 | 0,000 | 0,000 to 70,760 | 1,56 | 3,26 |
| Zamora 2013 (n=26) | 26 | 26,923 | 11,573 to 47,787 | 10,51 | 6,85 |
| Total (fixed effects) | 237 | 17,831 | 13,352 to 23,069 | 100,00 | 100,00 |
| Total (random effects) | 237 | 17,534 | 9,855 to 26,855 | 100,00 | 100,00 |

## Test for heterogeneity

| Q | 55,7283 |
| --- | --- |
| DF | 19 |
| Significance level | P < 0,0001 |
| I^2^ (inconsistency) | 65,91% |
| 95% CI for I^2^ | 45,38 to 78,72 |

# Meta-analysis: combined open exit all

| Variable for studies | Study |
| --- | --- |
| Variable for total number of cases | N |
| Variable for number of positive cases | Outcome |

| Study | Sample size | Proportion (%) | 95% CI | Weight (%) | |
| --- | --- | --- | --- | --- | --- |
|  |  |  |  | Fixed | Random |
| Barthod 2013 (n=5) | 5 | 0,000 | 0,000 to 52,182 | 2,33 | 4,09 |
| Cass 2013 (n=9) | 9 | 22,222 | 2,814 to 60,009 | 3,89 | 5,16 |
| Chen 2018 (n=7) | 7 | 0,000 | 0,000 to 40,962 | 3,11 | 4,70 |
| Dahlgren 2004 (n=4) | 4 | 75,000 | 19,412 to 99,369 | 1,95 | 3,70 |
| Flake 2000 (n=15) | 15 | 0,000 | 0,000 to 21,802 | 6,23 | 6,06 |
| George 2007 (n=3) | 3 | 100,000 | 29,240 to 100,000 | 1,56 | 3,24 |
| Hedrick 2003 (n=43) | 43 | 16,279 | 6,805 to 30,701 | 17,12 | 7,43 |
| Hedrick 2005 (n=9) | 9 | 22,222 | 2,814 to 60,009 | 3,89 | 5,16 |
| Kern 2007 (n=5) | 5 | 20,000 | 0,505 to 71,642 | 2,33 | 4,09 |
| Kornacki 2017 (n=4) | 4 | 0,000 | 0,000 to 60,236 | 1,95 | 3,70 |
| Kunisaki 2007 (n=14) | 14 | 0,000 | 0,000 to 23,164 | 5,84 | 5,95 |
| Laje 2012 (n=17) | 17 | 29,412 | 10,314 to 55,958 | 7,00 | 6,26 |
| Laje 2013 (n=4) | 4 | 50,000 | 6,759 to 93,241 | 1,95 | 3,70 |
| Laje 2015 (n=13) | 13 | 0,000 | 0,000 to 24,705 | 5,45 | 5,82 |
| Lazar 2011 (n=12) | 12 | 16,667 | 2,086 to 48,414 | 5,06 | 5,68 |
| Noah 2002 (n=34) | 34 | 38,235 | 22,167 to 56,436 | 13,62 | 7,19 |
| Pellicer 2007 (n=3) | 3 | 33,333 | 0,840 to 90,570 | 1,56 | 3,24 |
| Stoffan 2012 (n=7) | 7 | 14,286 | 0,361 to 57,872 | 3,11 | 4,70 |
| Tuncay Ozgunen 2010 (n=3) | 3 | 0,000 | 0,000 to 70,760 | 1,56 | 3,24 |
| Zamora 2013 (n=26) | 26 | 30,769 | 14,326 to 51,790 | 10,51 | 6,87 |
| Total (fixed effects) | 237 | 20,042 | 15,324 to 25,465 | 100,00 | 100,00 |
| Total (random effects) | 237 | 20,188 | 12,010 to 29,860 | 100,00 | 100,00 |

## Test for heterogeneity

| Q | 55,1007 |
| --- | --- |
| DF | 19 |
| Significance level | P < 0,0001 |
| I^2^ (inconsistency) | 65,52% |
| 95% CI for I^2^ | 44,68 to 78,51 |

# Meta-analysis: combined open MMC severe

| Variable for studies | Study |
| --- | --- |
| Variable for total number of cases | N |
| Variable for number of positive cases | Outcome |

| Study | Sample size | Proportion (%) | 95% CI | Weight (%) | |
| --- | --- | --- | --- | --- | --- |
|  |  |  |  | Fixed | Random |
| Bennett 2014 (n=43) | 43 | 4,651 | 0,568 to 15,811 | 5,55 | 7,73 |
| Botelho 2017 (n=45) | 45 | 2,222 | 0,0562 to 11,770 | 5,80 | 7,93 |
| Bruner 1999 (n=29) | 29 | 13,793 | 3,889 to 31,664 | 3,78 | 6,05 |
| Bruner 2000 (n=4) | 4 | 0,000 | 0,000 to 60,236 | 0,63 | 1,37 |
| Farmer 2003 (n=12) | 12 | 0,000 | 0,000 to 26,465 | 1,64 | 3,20 |
| Friszer 2016 (n=3) | 3 | 0,000 | 0,000 to 70,760 | 0,50 | 1,11 |
| Johnson 2016 (n=91) | 91 | 6,593 | 2,458 to 13,800 | 11,60 | 11,20 |
| Marenco 2013 (n=4) | 4 | 0,000 | 0,000 to 60,236 | 0,63 | 1,37 |
| Moldenhauer 2015 (n=100) | 100 | 3,000 | 0,623 to 8,518 | 12,74 | 11,63 |
| Moron 2018 (n=237) | 237 | 2,110 | 0,688 to 4,854 | 30,01 | 14,99 |
| Ochsenbein-Kolble 2017 (n=30) | 30 | 10,000 | 2,112 to 26,529 | 3,91 | 6,19 |
| Sinskey 2017 (n=47) | 47 | 0,000 | 0,000 to 7,549 | 6,05 | 8,13 |
| Soni 2016 (n=88) | 88 | 0,000 | 0,000 to 4,105 | 11,22 | 11,05 |
| Zamlynski 2014 (n=46) | 46 | 0,000 | 0,000 to 7,706 | 5,93 | 8,03 |
| Total (fixed effects) | 779 | 2,998 | 1,926 to 4,436 | 100,00 | 100,00 |
| Total (random effects) | 779 | 3,348 | 1,699 to 5,525 | 100,00 | 100,00 |

## Test for heterogeneity

| Q | 23,4601 |
| --- | --- |
| DF | 13 |
| Significance level | P = 0,0365 |
| I^2^ (inconsistency) | 44,59% |
| 95% CI for I^2^ | 0,00 to 70,35 |

# Meta-analysis: combined open MMC minor

| Variable for studies | Study |
| --- | --- |
| Variable for total number of cases | N |
| Variable for number of positive cases | Outcome |

| Study | Sample size | Proportion (%) | 95% CI | Weight (%) | |
| --- | --- | --- | --- | --- | --- |
|  |  |  |  | Fixed | Random |
| Bennett 2014 (n=43) | 43 | 0,000 | 0,000 to 8,221 | 5,55 | 8,18 |
| Botelho 2017 (n=45) | 45 | 2,222 | 0,0562 to 11,770 | 5,80 | 8,32 |
| Bruner 1999 (n=29) | 29 | 0,000 | 0,000 to 11,944 | 3,78 | 6,95 |
| Bruner 2000 (n=4) | 4 | 0,000 | 0,000 to 60,236 | 0,63 | 2,07 |
| Farmer 2003 (n=12) | 12 | 41,667 | 15,165 to 72,333 | 1,64 | 4,30 |
| Friszer 2016 (n=3) | 3 | 0,000 | 0,000 to 70,760 | 0,50 | 1,71 |
| Johnson 2016 (n=91) | 91 | 16,484 | 9,530 to 25,726 | 11,60 | 10,19 |
| Marenco 2013 (n=4) | 4 | 0,000 | 0,000 to 60,236 | 0,63 | 2,07 |
| Moldenhauer 2015 (n=100) | 100 | 10,000 | 4,900 to 17,622 | 12,74 | 10,39 |
| Moron 2018 (n=237) | 237 | 7,595 | 4,563 to 11,738 | 30,01 | 11,82 |
| Ochsenbein-Kolble 2017 (n=30) | 30 | 0,000 | 0,000 to 11,570 | 3,91 | 7,06 |
| Sinskey 2017 (n=47) | 47 | 4,255 | 0,520 to 14,541 | 6,05 | 8,44 |
| Soni 2016 (n=88) | 88 | 4,545 | 1,252 to 11,231 | 11,22 | 10,11 |
| Zamlynski 2014 (n=46) | 46 | 10,870 | 3,625 to 23,570 | 5,93 | 8,38 |
| Total (fixed effects) | 779 | 7,272 | 5,564 to 9,306 | 100,00 | 100,00 |
| Total (random effects) | 779 | 6,631 | 3,629 to 10,451 | 100,00 | 100,00 |

## Test for heterogeneity

| Q | 38,2736 |
| --- | --- |
| DF | 13 |
| Significance level | P = 0,0003 |
| I^2^ (inconsistency) | 66,03% |
| 95% CI for I^2^ | 40,27 to 80,69 |

# Meta-analysis: combined open MMC all complications

| Variable for studies | Study |
| --- | --- |
| Variable for total number of cases | N |
| Variable for number of positive cases | Outcome |

| Study | Sample size | Proportion (%) | 95% CI | Weight (%) | |
| --- | --- | --- | --- | --- | --- |
|  |  |  |  | Fixed | Random |
| Bennett 2014 (n=43) | 43 | 4,651 | 0,568 to 15,811 | 5,55 | 8,12 |
| Botelho 2017 (n=45) | 45 | 4,444 | 0,543 to 15,149 | 5,80 | 8,27 |
| Bruner 1999 (n=29) | 29 | 13,793 | 3,889 to 31,664 | 3,78 | 6,78 |
| Bruner 2000 (n=4) | 4 | 0,000 | 0,000 to 60,236 | 0,63 | 1,89 |
| Farmer 2003 (n=12) | 12 | 41,667 | 15,165 to 72,333 | 1,64 | 4,05 |
| Friszer 2016 (n=3) | 3 | 0,000 | 0,000 to 70,760 | 0,50 | 1,55 |
| Johnson 2016 (n=91) | 91 | 23,077 | 14,888 to 33,087 | 11,60 | 10,42 |
| Marenco 2013 (n=4) | 4 | 0,000 | 0,000 to 60,236 | 0,63 | 1,89 |
| Moldenhauer 2015 (n=100) | 100 | 13,000 | 7,107 to 21,204 | 12,74 | 10,66 |
| Moron 2018 (n=237) | 237 | 9,705 | 6,252 to 14,206 | 30,01 | 12,38 |
| Ochsenbein-Kolble 2017 (n=30) | 30 | 10,000 | 2,112 to 26,529 | 3,91 | 6,90 |
| Sinskey 2017 (n=47) | 47 | 4,255 | 0,520 to 14,541 | 6,05 | 8,42 |
| Soni 2016 (n=88) | 88 | 4,545 | 1,252 to 11,231 | 11,22 | 10,33 |
| Zamlynski 2014 (n=46) | 46 | 21,739 | 10,948 to 36,362 | 5,93 | 8,34 |
| Total (fixed effects) | 779 | 11,228 | 9,115 to 13,634 | 100,00 | 100,00 |
| Total (random effects) | 779 | 11,538 | 7,733 to 15,988 | 100,00 | 100,00 |

## Test for heterogeneity

| Q | 33,9623 |
| --- | --- |
| DF | 13 |
| Significance level | P = 0,0012 |
| I^2^ (inconsistency) | 61,72% |
| 95% CI for I^2^ | 31,59 to 78,58 |

# Meta-analysis: combined feto MMC severe

| Variable for studies | Study |
| --- | --- |
| Variable for total number of cases | N |
| Variable for number of positive cases | Outcome |

| Study | Sample size | Proportion (%) | 95% CI | Weight (%) | |
| --- | --- | --- | --- | --- | --- |
|  |  |  |  | Fixed | Random |
| Arens 2017 (n=59) | 59 | 0,000 | 0,000 to 6,061 | 21,66 | 16,91 |
| Belfort 2017 (n=22) | 22 | 9,091 | 1,121 to 29,161 | 8,30 | 11,14 |
| Bruner 2000 (n=4) | 4 | 25,000 | 0,631 to 80,588 | 1,81 | 3,72 |
| Degenhardt 2014 (n=51) | 51 | 0,000 | 0,000 to 6,978 | 18,77 | 16,11 |
| Kohn 2018 (n=34) | 34 | 8,824 | 1,858 to 23,678 | 12,64 | 13,75 |
| Pedreira 2014 (n=4) | 4 | 0,000 | 0,000 to 60,236 | 1,81 | 3,72 |
| Pedreira 2016 (n=10) | 10 | 0,000 | 0,000 to 30,850 | 3,97 | 6,95 |
| Verbeek 2012 (n=19) | 19 | 0,000 | 0,000 to 17,647 | 7,22 | 10,29 |
| Ziemann 2018 (n=65) | 65 | 0,000 | 0,000 to 5,517 | 23,83 | 17,42 |
| Total (fixed effects) | 268 | 1,916 | 0,652 to 4,315 | 100,00 | 100,00 |
| Total (random effects) | 268 | 2,747 | 0,559 to 6,520 | 100,00 | 100,00 |

## Test for heterogeneity

| Q | 16,0783 |
| --- | --- |
| DF | 8 |
| Significance level | P = 0,0413 |
| I^2^ (inconsistency) | 50,24% |
| 95% CI for I^2^ | 0,00 to 76,75 |

# Meta-analysis: combined fetoscopic MMC minor

| Variable for studies | Study |
| --- | --- |
| Variable for total number of cases | N |
| Variable for number of positive cases | Outcome |

| Study | Sample size | Proportion (%) | 95% CI | Weight (%) | |
| --- | --- | --- | --- | --- | --- |
|  |  |  |  | Fixed | Random |
| Arens 2017 (n=59) | 59 | 3,390 | 0,413 to 11,715 | 21,66 | 14,67 |
| Belfort 2017 (n=22) | 22 | 9,091 | 1,121 to 29,161 | 8,30 | 11,80 |
| Bruner 2000 (n=4) | 4 | 25,000 | 0,631 to 80,588 | 1,81 | 5,52 |
| Degenhardt 2014 (n=51) | 51 | 7,843 | 2,178 to 18,881 | 18,77 | 14,33 |
| Kohn 2018 (n=34) | 34 | 2,941 | 0,0744 to 15,327 | 12,64 | 13,24 |
| Pedreira 2014 (n=4) | 4 | 50,000 | 6,759 to 93,241 | 1,81 | 5,52 |
| Pedreira 2016 (n=10) | 10 | 0,000 | 0,000 to 30,850 | 3,97 | 8,78 |
| Verbeek 2012 (n=19) | 19 | 31,579 | 12,576 to 56,550 | 7,22 | 11,27 |
| Ziemann 2018 (n=65) | 65 | 0,000 | 0,000 to 5,517 | 23,83 | 14,87 |
| Total (fixed effects) | 268 | 5,796 | 3,352 to 9,234 | 100,00 | 100,00 |
| Total (random effects) | 268 | 9,042 | 3,226 to 17,398 | 100,00 | 100,00 |

## Test for heterogeneity

| Q | 29,6240 |
| --- | --- |
| DF | 8 |
| Significance level | P = 0,0002 |
| I^2^ (inconsistency) | 72,99% |
| 95% CI for I^2^ | 47,18 to 86,19 |

# Meta-analysis: combined fetoscopic MMC all

| Variable for studies | Study |
| --- | --- |
| Variable for total number of cases | N |
| Variable for number of positive cases | Outcome |

| Study | Sample size | Proportion (%) | 95% CI | Weight (%) | |
| --- | --- | --- | --- | --- | --- |
|  |  |  |  | Fixed | Random |
| Arens 2017 (n=59) | 59 | 3,390 | 0,413 to 11,715 | 21,66 | 14,06 |
| Belfort 2017 (n=22) | 22 | 18,182 | 5,187 to 40,285 | 8,30 | 11,85 |
| Bruner 2000 (n=4) | 4 | 50,000 | 6,759 to 93,241 | 1,81 | 6,19 |
| Degenhardt 2014 (n=51) | 51 | 7,843 | 2,178 to 18,881 | 18,77 | 13,81 |
| Kohn 2018 (n=34) | 34 | 11,765 | 3,300 to 27,450 | 12,64 | 12,99 |
| Pedreira 2014 (n=4) | 4 | 50,000 | 6,759 to 93,241 | 1,81 | 6,19 |
| Pedreira 2016 (n=10) | 10 | 0,000 | 0,000 to 30,850 | 3,97 | 9,28 |
| Verbeek 2012 (n=19) | 19 | 31,579 | 12,576 to 56,550 | 7,22 | 11,42 |
| Ziemann 2018 (n=65) | 65 | 0,000 | 0,000 to 5,517 | 23,83 | 14,20 |
| Total (fixed effects) | 268 | 7,503 | 4,692 to 11,264 | 100,00 | 100,00 |
| Total (random effects) | 268 | 12,492 | 4,832 to 23,057 | 100,00 | 100,00 |

## Test for heterogeneity

| Q | 37,3965 |
| --- | --- |
| DF | 8 |
| Significance level | P < 0,0001 |
| I^2^ (inconsistency) | 78,61% |
| 95% CI for I^2^ | 59,71 to 88,64 |

# Meta-analysis: combined fetoscopic CDH severe

| Variable for studies | Study |
| --- | --- |
| Variable for total number of cases | N |
| Variable for number of positive cases | Outcome |

| Study | Sample size | Proportion (%) | 95% CI | Weight (%) | |
| --- | --- | --- | --- | --- | --- |
|  |  |  |  | Fixed | Random |
| Deprest 2005 (n=20) | 20 | 0,000 | 0,000 to 16,843 | 3,25 | 5,54 |
| Harrison 2003 (n=11) | 11 | 27,273 | 6,022 to 60,974 | 1,85 | 3,46 |
| Jani 2005 (n=24) | 24 | 0,000 | 0,000 to 14,247 | 3,86 | 6,36 |
| Jani 2006 (n=28) | 28 | 0,000 | 0,000 to 12,344 | 4,48 | 7,12 |
| Jani 2009 (n=210) | 210 | 0,000 | 0,000 to 1,741 | 32,61 | 20,02 |
| Jimenez 2017 (n=201) | 201 | 0,000 | 0,000 to 1,819 | 31,22 | 19,77 |
| Kosinski 2017 (n=28) | 28 | 0,000 | 0,000 to 12,344 | 4,48 | 7,12 |
| Manrique 2008 (n=11) | 11 | 0,000 | 0,000 to 28,491 | 1,85 | 3,46 |
| Peralta 2011 (n=8) | 8 | 0,000 | 0,000 to 36,942 | 1,39 | 2,67 |
| Persico 2017 (n=21) | 21 | 0,000 | 0,000 to 16,110 | 3,40 | 5,75 |
| Ruano 2012 (n=35) | 35 | 2,857 | 0,0723 to 14,917 | 5,56 | 8,33 |
| Ruano 2012 (n=20) | 20 | 0,000 | 0,000 to 16,843 | 3,25 | 5,54 |
| Ruano 2013 (n=17) | 17 | 0,000 | 0,000 to 19,506 | 2,78 | 4,89 |
| Total (fixed effects) | 634 | 0,569 | 0,144 to 1,504 | 100,00 | 100,00 |
| Total (random effects) | 634 | 1,075 | 0,228 to 2,540 | 100,00 | 100,00 |

## Test for heterogeneity

| Q | 17,9049 |
| --- | --- |
| DF | 12 |
| Significance level | P = 0,1186 |
| I^2^ (inconsistency) | 32,98% |
| 95% CI for I^2^ | 0,00 to 65,36 |

# Meta-analysis: combined fetoscopic CDH minor

| Variable for studies | Study |
| --- | --- |
| Variable for total number of cases | N |
| Variable for number of positive cases | Outcome |

| Study | Sample size | Proportion (%) | 95% CI | Weight (%) | |
| --- | --- | --- | --- | --- | --- |
|  |  |  |  | Fixed | Random |
| Deprest 2005 (n=20) | 20 | 0,000 | 0,000 to 16,843 | 3,25 | 6,64 |
| Harrison 2003 (n=11) | 11 | 36,364 | 10,926 to 69,210 | 1,85 | 4,57 |
| Jani 2005 (n=24) | 24 | 0,000 | 0,000 to 14,247 | 3,86 | 7,34 |
| Jani 2006 (n=28) | 28 | 0,000 | 0,000 to 12,344 | 4,48 | 7,96 |
| Jani 2009 (n=210) | 210 | 3,333 | 1,350 to 6,747 | 32,61 | 14,51 |
| Jimenez 2017 (n=201) | 201 | 0,000 | 0,000 to 1,819 | 31,22 | 14,42 |
| Kosinski 2017 (n=28) | 28 | 0,000 | 0,000 to 12,344 | 4,48 | 7,96 |
| Manrique 2008 (n=11) | 11 | 0,000 | 0,000 to 28,491 | 1,85 | 4,57 |
| Peralta 2011 (n=8) | 8 | 0,000 | 0,000 to 36,942 | 1,39 | 3,68 |
| Persico 2017 (n=21) | 21 | 0,000 | 0,000 to 16,110 | 3,40 | 6,82 |
| Ruano 2012 (n=35) | 35 | 0,000 | 0,000 to 10,003 | 5,56 | 8,86 |
| Ruano 2012 (n=20) | 20 | 5,000 | 0,127 to 24,873 | 3,25 | 6,64 |
| Ruano 2013 (n=17) | 17 | 5,882 | 0,149 to 28,689 | 2,78 | 6,03 |
| Total (fixed effects) | 634 | 1,705 | 0,855 to 3,028 | 100,00 | 100,00 |
| Total (random effects) | 634 | 2,385 | 0,705 to 5,023 | 100,00 | 100,00 |

## Test for heterogeneity

| Q | 27,9084 |
| --- | --- |
| DF | 12 |
| Significance level | P = 0,0057 |
| I^2^ (inconsistency) | 57,00% |
| 95% CI for I^2^ | 20,20 to 76,83 |

# Meta-analysis: combined fetoscopic CDH all complications

| Variable for studies | Study |
| --- | --- |
| Variable for total number of cases | N |
| Variable for number of positive cases | Outcome |

| Study | Sample size | Proportion (%) | 95% CI | Weight (%) | |
| --- | --- | --- | --- | --- | --- |
|  |  |  |  | Fixed | Random |
| Deprest 2005 (n=20) | 20 | 0,000 | 0,000 to 16,843 | 3,25 | 7,18 |
| Harrison 2003 (n=11) | 11 | 63,636 | 30,790 to 89,074 | 1,85 | 5,43 |
| Jani 2005 (n=24) | 24 | 0,000 | 0,000 to 14,247 | 3,86 | 7,71 |
| Jani 2006 (n=28) | 28 | 0,000 | 0,000 to 12,344 | 4,48 | 8,15 |
| Jani 2009 (n=210) | 210 | 3,333 | 1,350 to 6,747 | 32,61 | 11,73 |
| Jimenez 2017 (n=201) | 201 | 0,000 | 0,000 to 1,819 | 31,22 | 11,69 |
| Kosinski 2017 (n=28) | 28 | 0,000 | 0,000 to 12,344 | 4,48 | 8,15 |
| Manrique 2008 (n=11) | 11 | 0,000 | 0,000 to 28,491 | 1,85 | 5,43 |
| Peralta 2011 (n=8) | 8 | 0,000 | 0,000 to 36,942 | 1,39 | 4,56 |
| Persico 2017 (n=21) | 21 | 0,000 | 0,000 to 16,110 | 3,40 | 7,33 |
| Ruano 2012 (n=35) | 35 | 2,857 | 0,0723 to 14,917 | 5,56 | 8,75 |
| Ruano 2012 (n=20) | 20 | 5,000 | 0,127 to 24,873 | 3,25 | 7,18 |
| Ruano 2013 (n=17) | 17 | 5,882 | 0,149 to 28,689 | 2,78 | 6,70 |
| Total (fixed effects) | 634 | 2,011 | 1,075 to 3,413 | 100,00 | 100,00 |
| Total (random effects) | 634 | 3,442 | 0,982 to 7,321 | 100,00 | 100,00 |

## Test for heterogeneity

| Q | 43,9322 |
| --- | --- |
| DF | 12 |
| Significance level | P < 0,0001 |
| I^2^ (inconsistency) | 72,69% |
| 95% CI for I^2^ | 52,39 to 84,33 |

# Meta-analysis: combined feto laser severe

| Variable for studies | Study |
| --- | --- |
| Variable for total number of cases | N |
| Variable for number of positive cases | Outcome |

| Study | Sample size | Proportion (%) | 95% CI | Weight (%) | |
| --- | --- | --- | --- | --- | --- |
|  |  |  |  | Fixed | Random |
| Aboudiab 2017 (n=18) | 18 | 5,556 | 0,141 to 27,294 | 0,28 | 0,96 |
| Baschat 2013 (n=147) | 147 | 4,762 | 1,936 to 9,565 | 2,17 | 2,18 |
| Chalouhi 2016 (n=22) | 22 | 0,000 | 0,000 to 15,437 | 0,34 | 1,08 |
| Chang 2006 (n=27) | 27 | 3,704 | 0,0937 to 18,971 | 0,41 | 1,20 |
| Chang 2016 (n=100) | 100 | 0,000 | 0,000 to 3,622 | 1,48 | 2,00 |
| Chmait 2013 (n=318) | 318 | 0,629 | 0,0763 to 2,253 | 4,69 | 2,43 |
| Chmait 2017 (n=19) | 19 | 0,000 | 0,000 to 17,647 | 0,29 | 0,99 |
| Crombleholme 2007 (n=20) | 20 | 0,000 | 0,000 to 16,843 | 0,31 | 1,02 |
| De Lia 1995 (n=26) | 26 | 0,000 | 0,000 to 13,227 | 0,40 | 1,18 |
| De Lia 1999 (n=67) | 67 | 1,493 | 0,0378 to 8,038 | 1,00 | 1,78 |
| De Lia 2009 (n=10) | 10 | 0,000 | 0,000 to 30,850 | 0,16 | 0,65 |
| Deprest 1998 (n=6) | 6 | 0,000 | 0,000 to 45,926 | 0,10 | 0,45 |
| Draga 2016 (n=37) | 37 | 2,703 | 0,0684 to 14,160 | 0,56 | 1,41 |
| Duron 2014 (n=85) | 85 | 1,176 | 0,0298 to 6,381 | 1,26 | 1,92 |
| Ek 2012 (n=) | 67 | 4,478 | 0,933 to 12,533 | 1,00 | 1,78 |
| Habli 2009 (n=152) | 152 | 8,553 | 4,633 to 14,181 | 2,25 | 2,19 |
| Has 2014 (n=85) | 85 | 0,000 | 0,000 to 4,247 | 1,26 | 1,92 |
| Hecher 2000 (n=200) | 200 | 0,000 | 0,000 to 1,828 | 2,95 | 2,29 |
| Hernandez-Andrade 2011 (n=35) | 35 | 0,000 | 0,000 to 10,003 | 0,53 | 1,37 |
| Huber 2008 (n=176) | 176 | 1,136 | 0,138 to 4,044 | 2,60 | 2,25 |
| Ishii 2014 (n=16) | 16 | 0,000 | 0,000 to 20,591 | 0,25 | 0,89 |
| Ishii 2015 (n=10) | 10 | 0,000 | 0,000 to 30,850 | 0,16 | 0,65 |
| Lanna 2017 (n=373) | 373 | 5,630 | 3,518 to 8,478 | 5,49 | 2,46 |
| Lecointre 2017 (n=200) | 200 | 0,000 | 0,000 to 1,828 | 2,95 | 2,29 |
| Malshe 2017 (n=203) | 203 | 5,911 | 3,091 to 10,098 | 3,00 | 2,30 |
| Martinez 2012 (n=500) | 500 | 0,000 | 0,000 to 0,735 | 7,36 | 2,51 |
| Middeldorp 2007 (n=100) | 100 | 0,000 | 0,000 to 3,622 | 1,48 | 2,00 |
| Miyadahira 2018 (n=67) | 67 | 1,493 | 0,0378 to 8,038 | 1,00 | 1,78 |
| Molina-Garcia 2009 (n=22) | 22 | 0,000 | 0,000 to 15,437 | 0,34 | 1,08 |
| Morris 2010 (n=164) | 164 | 0,000 | 0,000 to 2,224 | 2,42 | 2,22 |
| Mullers 2015 (n=105) | 105 | 0,000 | 0,000 to 3,452 | 1,56 | 2,03 |
| Nakata 2016 (n=6) | 6 | 16,667 | 0,421 to 64,123 | 0,10 | 0,45 |
| Nguyen 2012 (n=98) | 98 | 0,000 | 0,000 to 3,694 | 1,45 | 1,99 |
| Ozawa 2017 (n=11) | 11 | 0,000 | 0,000 to 28,491 | 0,18 | 0,69 |
| Papanna 2010 (n=48) | 48 | 2,083 | 0,0527 to 11,070 | 0,72 | 1,58 |
| Papanna 2012 (n=163) | 163 | 3,067 | 1,003 to 7,013 | 2,41 | 2,22 |
| Peeters 2014 (n=338) | 338 | 0,000 | 0,000 to 1,085 | 4,98 | 2,44 |
| Persico 2016 (n=106) | 106 | 0,000 | 0,000 to 3,420 | 1,57 | 2,03 |
| Quintero 2000 (n=92) | 92 | 0,000 | 0,000 to 3,930 | 1,37 | 1,96 |
| Quintero 2001 (n=11) | 11 | 18,182 | 2,283 to 51,776 | 0,18 | 0,69 |
| Rossi 2008 (n=266) | 266 | 0,000 | 0,000 to 1,377 | 3,92 | 2,38 |
| Ruano 2009 (n=19) | 19 | 0,000 | 0,000 to 17,647 | 0,29 | 0,99 |
| Ruegg 2018 (n=37) | 37 | 2,703 | 0,0684 to 14,160 | 0,56 | 1,41 |
| Rustico 2012 (n=150) | 150 | 8,667 | 4,696 to 14,364 | 2,22 | 2,19 |
| Said 2008 (n=10) | 10 | 0,000 | 0,000 to 30,850 | 0,16 | 0,65 |
| Senat 2004 (n=72) | 72 | 1,389 | 0,0352 to 7,497 | 1,07 | 1,83 |
| Sepulveda 2007 (n=33) | 33 | 0,000 | 0,000 to 10,576 | 0,50 | 1,33 |
| Shamshirsaz 2015 (n=55) | 55 | 3,636 | 0,443 to 12,526 | 0,82 | 1,66 |
| Slaghekke 2014 (n=274) | 274 | 0,000 | 0,000 to 1,337 | 4,04 | 2,39 |
| Taniguchi 2015 (n=3) | 3 | 0,000 | 0,000 to 70,760 | 0,059 | 0,28 |
| Tchirikov 2011 (n=80) | 80 | 0,000 | 0,000 to 4,506 | 1,19 | 1,89 |
| Teoh 2013 (n=49) | 49 | 0,000 | 0,000 to 7,252 | 0,73 | 1,59 |
| Thia 2017 (n=5) | 5 | 0,000 | 0,000 to 52,182 | 0,088 | 0,40 |
| Ville 1997 (n=132) | 132 | 0,000 | 0,000 to 2,756 | 1,95 | 2,13 |
| Ville 1998 (n=44) | 44 | 0,000 | 0,000 to 8,042 | 0,66 | 1,52 |
| Weingertner 2011 (n=100) | 100 | 0,000 | 0,000 to 3,622 | 1,48 | 2,00 |
| Wilson 2016 (n=151) | 151 | 0,000 | 0,000 to 2,413 | 2,23 | 2,19 |
| Yamamoto 2005 (n=175) | 175 | 2,286 | 0,626 to 5,749 | 2,59 | 2,25 |
| Yang 2010 (n=30) | 30 | 0,000 | 0,000 to 11,570 | 0,46 | 1,27 |
| Zaretsky 2018 (n=749) | 749 | 6,008 | 4,416 to 7,957 | 11,02 | 2,57 |
| Zhao 2016 (n=62) | 62 | 0,000 | 0,000 to 5,776 | 0,93 | 1,74 |
| Total (fixed effects) | 6746 | 1,497 | 1,222 to 1,814 | 100,00 | 100,00 |
| Total (random effects) | 6746 | 1,507 | 0,913 to 2,246 | 100,00 | 100,00 |

## Test for heterogeneity

| Q | 249,9310 |
| --- | --- |
| DF | 60 |
| Significance level | P < 0,0001 |
| I^2^ (inconsistency) | 75,99% |
| 95% CI for I^2^ | 69,35 to 81,20 |

# Meta-analysis: combined fetoscopic laser minor

| Variable for studies | Study |
| --- | --- |
| Variable for total number of cases | N |
| Variable for number of positive cases | Outcome |

| Study | Sample size | Proportion (%) | 95% CI | Weight (%) | |
| --- | --- | --- | --- | --- | --- |
|  |  |  |  | Fixed | Random |
| Aboudiab 2017 (n=18) | 18 | 0,000 | 0,000 to 18,530 | 0,28 | 1,19 |
| Baschat 2013 (n=147) | 147 | 6,803 | 3,310 to 12,155 | 2,17 | 2,01 |
| Chalouhi 2016 (n=22) | 22 | 4,545 | 0,115 to 22,844 | 0,34 | 1,30 |
| Chang 2006 (n=27) | 27 | 7,407 | 0,910 to 24,290 | 0,41 | 1,40 |
| Chang 2016 (n=100) | 100 | 3,000 | 0,623 to 8,518 | 1,48 | 1,92 |
| Chmait 2013 (n=318) | 318 | 1,887 | 0,695 to 4,061 | 4,69 | 2,13 |
| Chmait 2017 (n=19) | 19 | 10,526 | 1,301 to 33,138 | 0,29 | 1,22 |
| Crombleholme 2007 (n=20) | 20 | 10,000 | 1,235 to 31,698 | 0,31 | 1,25 |
| De Lia 1995 (n=26) | 26 | 26,923 | 11,573 to 47,787 | 0,40 | 1,38 |
| De Lia 1999 (n=67) | 67 | 0,000 | 0,000 to 5,357 | 1,00 | 1,80 |
| De Lia 2009 (n=10) | 10 | 10,000 | 0,253 to 44,502 | 0,16 | 0,89 |
| Deprest 1998 (n=6) | 6 | 0,000 | 0,000 to 45,926 | 0,10 | 0,66 |
| Draga 2016 (n=37) | 37 | 0,000 | 0,000 to 9,489 | 0,56 | 1,56 |
| Duron 2014 (n=85) | 85 | 9,412 | 4,152 to 17,705 | 1,26 | 1,87 |
| Ek 2012 (n=) | 67 | 0,000 | 0,000 to 5,357 | 1,00 | 1,80 |
| Habli 2009 (n=152) | 152 | 3,289 | 1,077 to 7,509 | 2,25 | 2,02 |
| Has 2014 (n=85) | 85 | 2,353 | 0,286 to 8,242 | 1,26 | 1,87 |
| Hecher 2000 (n=200) | 200 | 0,000 | 0,000 to 1,828 | 2,95 | 2,07 |
| Hernandez-Andrade 2011 (n=35) | 35 | 34,286 | 19,132 to 52,211 | 0,53 | 1,53 |
| Huber 2008 (n=176) | 176 | 0,568 | 0,0144 to 3,125 | 2,60 | 2,04 |
| Ishii 2014 (n=16) | 16 | 0,000 | 0,000 to 20,591 | 0,25 | 1,13 |
| Ishii 2015 (n=10) | 10 | 0,000 | 0,000 to 30,850 | 0,16 | 0,89 |
| Lanna 2017 (n=373) | 373 | 0,000 | 0,000 to 0,984 | 5,49 | 2,14 |
| Lecointre 2017 (n=200) | 200 | 0,000 | 0,000 to 1,828 | 2,95 | 2,07 |
| Malshe 2017 (n=203) | 203 | 6,404 | 3,454 to 10,702 | 3,00 | 2,07 |
| Martinez 2012 (n=500) | 500 | 0,000 | 0,000 to 0,735 | 7,36 | 2,16 |
| Middeldorp 2007 (n=100) | 100 | 4,000 | 1,100 to 9,926 | 1,48 | 1,92 |
| Miyadahira 2018 (n=67) | 67 | 0,000 | 0,000 to 5,357 | 1,00 | 1,80 |
| Molina-Garcia 2009 (n=22) | 22 | 0,000 | 0,000 to 15,437 | 0,34 | 1,30 |
| Morris 2010 (n=164) | 164 | 0,000 | 0,000 to 2,224 | 2,42 | 2,03 |
| Mullers 2015 (n=105) | 105 | 3,810 | 1,048 to 9,467 | 1,56 | 1,93 |
| Nakata 2016 (n=6) | 6 | 0,000 | 0,000 to 45,926 | 0,10 | 0,66 |
| Nguyen 2012 (n=98) | 98 | 1,020 | 0,0258 to 5,554 | 1,45 | 1,91 |
| Ozawa 2017 (n=11) | 11 | 0,000 | 0,000 to 28,491 | 0,18 | 0,94 |
| Papanna 2010 (n=48) | 48 | 4,167 | 0,509 to 14,254 | 0,72 | 1,67 |
| Papanna 2012 (n=163) | 163 | 3,067 | 1,003 to 7,013 | 2,41 | 2,03 |
| Peeters 2014 (n=338) | 338 | 7,692 | 5,086 to 11,068 | 4,98 | 2,13 |
| Persico 2016 (n=106) | 106 | 1,887 | 0,229 to 6,650 | 1,57 | 1,94 |
| Quintero 2000 (n=92) | 92 | 4,348 | 1,197 to 10,759 | 1,37 | 1,90 |
| Quintero 2001 (n=11) | 11 | 0,000 | 0,000 to 28,491 | 0,18 | 0,94 |
| Rossi 2008 (n=266) | 266 | 10,902 | 7,424 to 15,281 | 3,92 | 2,11 |
| Ruano 2009 (n=19) | 19 | 0,000 | 0,000 to 17,647 | 0,29 | 1,22 |
| Ruegg 2018 (n=37) | 37 | 37,838 | 22,458 to 55,243 | 0,56 | 1,56 |
| Rustico 2012 (n=150) | 150 | 4,000 | 1,482 to 8,503 | 2,22 | 2,01 |
| Said 2008 (n=10) | 10 | 0,000 | 0,000 to 30,850 | 0,16 | 0,89 |
| Senat 2004 (n=72) | 72 | 0,000 | 0,000 to 4,994 | 1,07 | 1,82 |
| Sepulveda 2007 (n=33) | 33 | 9,091 | 1,915 to 24,332 | 0,50 | 1,50 |
| Shamshirsaz 2015 (n=55) | 55 | 12,727 | 5,274 to 24,480 | 0,82 | 1,72 |
| Slaghekke 2014 (n=274) | 274 | 4,380 | 2,283 to 7,525 | 4,04 | 2,11 |
| Taniguchi 2015 (n=3) | 3 | 33,333 | 0,840 to 90,570 | 0,059 | 0,43 |
| Tchirikov 2011 (n=80) | 80 | 0,000 | 0,000 to 4,506 | 1,19 | 1,86 |
| Teoh 2013 (n=49) | 49 | 0,000 | 0,000 to 7,252 | 0,73 | 1,68 |
| Thia 2017 (n=5) | 5 | 20,000 | 0,505 to 71,642 | 0,088 | 0,59 |
| Ville 1997 (n=132) | 132 | 4,545 | 1,686 to 9,631 | 1,95 | 1,99 |
| Ville 1998 (n=44) | 44 | 11,364 | 3,794 to 24,558 | 0,66 | 1,63 |
| Weingertner 2011 (n=100) | 100 | 0,000 | 0,000 to 3,622 | 1,48 | 1,92 |
| Wilson 2016 (n=151) | 151 | 0,662 | 0,0168 to 3,634 | 2,23 | 2,02 |
| Yamamoto 2005 (n=175) | 175 | 10,286 | 6,211 to 15,768 | 2,59 | 2,04 |
| Yang 2010 (n=30) | 30 | 10,000 | 2,112 to 26,529 | 0,46 | 1,46 |
| Zaretsky 2018 (n=749) | 749 | 0,000 | 0,000 to 0,491 | 11,02 | 2,19 |
| Zhao 2016 (n=62) | 62 | 19,355 | 10,421 to 31,369 | 0,93 | 1,77 |
| Total (fixed effects) | 6746 | 2,380 | 2,031 to 2,771 | 100,00 | 100,00 |
| Total (random effects) | 6746 | 4,029 | 2,734 to 5,561 | 100,00 | 100,00 |

## Test for heterogeneity

| Q | 454,1620 |
| --- | --- |
| DF | 60 |
| Significance level | P < 0,0001 |
| I^2^ (inconsistency) | 86,79% |
| 95% CI for I^2^ | 83,76 to 89,25 |

# Meta-analysis: combined fetoscopic laser all complications

| Variable for studies | Study |
| --- | --- |
| Variable for total number of cases | N |
| Variable for number of positive cases | Outcome |

| Study | Sample size | Proportion (%) | 95% CI | Weight (%) | |
| --- | --- | --- | --- | --- | --- |
|  |  |  |  | Fixed | Random |
| Aboudiab 2017 (n=18) | 18 | 5,556 | 0,141 to 27,294 | 0,28 | 1,17 |
| Baschat 2013 (n=147) | 147 | 11,565 | 6,883 to 17,870 | 2,17 | 2,03 |
| Chalouhi 2016 (n=22) | 22 | 4,545 | 0,115 to 22,844 | 0,34 | 1,28 |
| Chang 2006 (n=27) | 27 | 11,111 | 2,353 to 29,159 | 0,41 | 1,39 |
| Chang 2016 (n=100) | 100 | 3,000 | 0,623 to 8,518 | 1,48 | 1,93 |
| Chmait 2013 (n=318) | 318 | 2,516 | 1,092 to 4,897 | 4,69 | 2,15 |
| Chmait 2017 (n=19) | 19 | 10,526 | 1,301 to 33,138 | 0,29 | 1,20 |
| Crombleholme 2007 (n=20) | 20 | 10,000 | 1,235 to 31,698 | 0,31 | 1,23 |
| De Lia 1995 (n=26) | 26 | 26,923 | 11,573 to 47,787 | 0,40 | 1,37 |
| De Lia 1999 (n=67) | 67 | 1,493 | 0,0378 to 8,038 | 1,00 | 1,80 |
| De Lia 2009 (n=10) | 10 | 10,000 | 0,253 to 44,502 | 0,16 | 0,87 |
| Deprest 1998 (n=6) | 6 | 0,000 | 0,000 to 45,926 | 0,10 | 0,64 |
| Draga 2016 (n=37) | 37 | 2,703 | 0,0684 to 14,160 | 0,56 | 1,54 |
| Duron 2014 (n=85) | 85 | 10,588 | 4,957 to 19,150 | 1,26 | 1,88 |
| Ek 2012 (n=) | 67 | 4,478 | 0,933 to 12,533 | 1,00 | 1,80 |
| Habli 2009 (n=152) | 152 | 11,842 | 7,172 to 18,069 | 2,25 | 2,03 |
| Has 2014 (n=85) | 85 | 2,353 | 0,286 to 8,242 | 1,26 | 1,88 |
| Hecher 2000 (n=200) | 200 | 0,000 | 0,000 to 1,828 | 2,95 | 2,09 |
| Hernandez-Andrade 2011 (n=35) | 35 | 34,286 | 19,132 to 52,211 | 0,53 | 1,52 |
| Huber 2008 (n=176) | 176 | 1,705 | 0,353 to 4,900 | 2,60 | 2,06 |
| Ishii 2014 (n=16) | 16 | 0,000 | 0,000 to 20,591 | 0,25 | 1,11 |
| Ishii 2015 (n=10) | 10 | 0,000 | 0,000 to 30,850 | 0,16 | 0,87 |
| Lanna 2017 (n=373) | 373 | 5,630 | 3,518 to 8,478 | 5,49 | 2,17 |
| Lecointre 2017 (n=200) | 200 | 0,000 | 0,000 to 1,828 | 2,95 | 2,09 |
| Malshe 2017 (n=203) | 203 | 12,315 | 8,131 to 17,641 | 3,00 | 2,09 |
| Martinez 2012 (n=500) | 500 | 0,000 | 0,000 to 0,735 | 7,36 | 2,19 |
| Middeldorp 2007 (n=100) | 100 | 4,000 | 1,100 to 9,926 | 1,48 | 1,93 |
| Miyadahira 2018 (n=67) | 67 | 1,493 | 0,0378 to 8,038 | 1,00 | 1,80 |
| Molina-Garcia 2009 (n=22) | 22 | 0,000 | 0,000 to 15,437 | 0,34 | 1,28 |
| Morris 2010 (n=164) | 164 | 0,000 | 0,000 to 2,224 | 2,42 | 2,05 |
| Mullers 2015 (n=105) | 105 | 3,810 | 1,048 to 9,467 | 1,56 | 1,94 |
| Nakata 2016 (n=6) | 6 | 16,667 | 0,421 to 64,123 | 0,10 | 0,64 |
| Nguyen 2012 (n=98) | 98 | 1,020 | 0,0258 to 5,554 | 1,45 | 1,92 |
| Ozawa 2017 (n=11) | 11 | 0,000 | 0,000 to 28,491 | 0,18 | 0,91 |
| Papanna 2010 (n=48) | 48 | 6,250 | 1,308 to 17,196 | 0,72 | 1,66 |
| Papanna 2012 (n=163) | 163 | 6,135 | 2,981 to 10,993 | 2,41 | 2,05 |
| Peeters 2014 (n=338) | 338 | 7,692 | 5,086 to 11,068 | 4,98 | 2,16 |
| Persico 2016 (n=106) | 106 | 1,887 | 0,229 to 6,650 | 1,57 | 1,95 |
| Quintero 2000 (n=92) | 92 | 4,348 | 1,197 to 10,759 | 1,37 | 1,91 |
| Quintero 2001 (n=11) | 11 | 18,182 | 2,283 to 51,776 | 0,18 | 0,91 |
| Rossi 2008 (n=266) | 266 | 10,902 | 7,424 to 15,281 | 3,92 | 2,13 |
| Ruano 2009 (n=19) | 19 | 0,000 | 0,000 to 17,647 | 0,29 | 1,20 |
| Ruegg 2018 (n=37) | 37 | 40,541 | 24,754 to 57,900 | 0,56 | 1,54 |
| Rustico 2012 (n=150) | 150 | 12,667 | 7,801 to 19,072 | 2,22 | 2,03 |
| Said 2008 (n=10) | 10 | 0,000 | 0,000 to 30,850 | 0,16 | 0,87 |
| Senat 2004 (n=72) | 72 | 1,389 | 0,0352 to 7,497 | 1,07 | 1,82 |
| Sepulveda 2007 (n=33) | 33 | 9,091 | 1,915 to 24,332 | 0,50 | 1,49 |
| Shamshirsaz 2015 (n=55) | 55 | 16,364 | 7,766 to 28,803 | 0,82 | 1,72 |
| Slaghekke 2014 (n=274) | 274 | 4,380 | 2,283 to 7,525 | 4,04 | 2,13 |
| Taniguchi 2015 (n=3) | 3 | 33,333 | 0,840 to 90,570 | 0,059 | 0,42 |
| Tchirikov 2011 (n=80) | 80 | 0,000 | 0,000 to 4,506 | 1,19 | 1,86 |
| Teoh 2013 (n=49) | 49 | 0,000 | 0,000 to 7,252 | 0,73 | 1,67 |
| Thia 2017 (n=5) | 5 | 20,000 | 0,505 to 71,642 | 0,088 | 0,57 |
| Ville 1997 (n=132) | 132 | 4,545 | 1,686 to 9,631 | 1,95 | 2,00 |
| Ville 1998 (n=44) | 44 | 11,364 | 3,794 to 24,558 | 0,66 | 1,63 |
| Weingertner 2011 (n=100) | 100 | 0,000 | 0,000 to 3,622 | 1,48 | 1,93 |
| Wilson 2016 (n=151) | 151 | 0,662 | 0,0168 to 3,634 | 2,23 | 2,03 |
| Yamamoto 2005 (n=175) | 175 | 12,571 | 8,049 to 18,413 | 2,59 | 2,06 |
| Yang 2010 (n=30) | 30 | 10,000 | 2,112 to 26,529 | 0,46 | 1,44 |
| Zaretsky 2018 (n=749) | 749 | 6,008 | 4,416 to 7,957 | 11,02 | 2,22 |
| Zhao 2016 (n=62) | 62 | 19,355 | 10,421 to 31,369 | 0,93 | 1,77 |
| Total (fixed effects) | 6746 | 4,624 | 4,138 to 5,150 | 100,00 | 100,00 |
| Total (random effects) | 6746 | 5,863 | 4,331 to 7,610 | 100,00 | 100,00 |

## Test for heterogeneity

| Q | 425,7183 |
| --- | --- |
| DF | 60 |
| Significance level | P < 0,0001 |
| I^2^ (inconsistency) | 85,91% |
| 95% CI for I^2^ | 82,61 to 88,58 |

# Meta-analysis: combined fetoscopic reduction severe

| Variable for studies | Study |
| --- | --- |
| Variable for total number of cases | N |
| Variable for number of positive cases | Outcome |

| Study | Sample size | Proportion (%) | 95% CI | Weight (%) | |
| --- | --- | --- | --- | --- | --- |
|  |  |  |  | Fixed | Random |
| Bebbington 2012 (n=146) | 146 | 0,000 | 0,000 to 2,495 | 11,60 | 6,44 |
| Berg 2014 (n=7) | 7 | 14,286 | 0,361 to 57,872 | 0,63 | 1,29 |
| Delabaere 2013 (n=30) | 30 | 3,333 | 0,0844 to 17,217 | 2,45 | 3,46 |
| Deprest 2000 (n=10) | 10 | 10,000 | 0,253 to 44,502 | 0,87 | 1,68 |
| Gallot 2003 (n=11) | 11 | 9,091 | 0,230 to 41,278 | 0,95 | 1,79 |
| Gouverneur 2009 (n=54) | 54 | 0,000 | 0,000 to 6,603 | 4,34 | 4,65 |
| Gul 2008 (n=9) | 9 | 0,000 | 0,000 to 33,627 | 0,79 | 1,55 |
| Has 2014 (n=71) | 71 | 0,000 | 0,000 to 5,063 | 5,68 | 5,19 |
| He 2010 (n=14) | 14 | 0,000 | 0,000 to 23,164 | 1,18 | 2,13 |
| Ilagan 2008 (n=27) | 27 | 11,111 | 2,353 to 29,159 | 2,21 | 3,26 |
| Jelin 2010 (n=7) | 7 | 0,000 | 0,000 to 40,962 | 0,63 | 1,29 |
| King 2017 (n=43) | 43 | 9,302 | 2,593 to 22,135 | 3,47 | 4,18 |
| Lanna 2012 (n=118) | 118 | 0,000 | 0,000 to 3,078 | 9,39 | 6,11 |
| Lee 2013 (n=98) | 98 | 0,000 | 0,000 to 3,694 | 7,81 | 5,79 |
| Lewi 2006 (n=80) | 80 | 1,250 | 0,0316 to 6,769 | 6,39 | 5,42 |
| Moise 2008 (n=9) | 9 | 11,111 | 0,281 to 48,250 | 0,79 | 1,55 |
| Nobili 2013 (n=48) | 48 | 0,000 | 0,000 to 7,397 | 3,87 | 4,41 |
| Paramasivam 2010 (n=35) | 35 | 0,000 | 0,000 to 10,003 | 2,84 | 3,77 |
| Peng 2016 (n=93) | 93 | 0,000 | 0,000 to 3,889 | 7,42 | 5,70 |
| Quintero 1996 (n=13) | 13 | 7,692 | 0,195 to 36,030 | 1,10 | 2,02 |
| Quintero 2006 (n=51) | 51 | 5,882 | 1,230 to 16,242 | 4,10 | 4,53 |
| Roman 2010 (n=60) | 60 | 0,000 | 0,000 to 5,963 | 4,81 | 4,86 |
| Schou 2018 (n=102) | 102 | 0,000 | 0,000 to 3,552 | 8,13 | 5,86 |
| Sugibayashi 2016 (n=40) | 40 | 0,000 | 0,000 to 8,810 | 3,24 | 4,04 |
| Takano 2015 (n=10) | 10 | 0,000 | 0,000 to 30,850 | 0,87 | 1,68 |
| Taylor 2002 (n=15) | 15 | 13,333 | 1,658 to 40,460 | 1,26 | 2,23 |
| Tsao 2002 (n=13) | 13 | 0,000 | 0,000 to 24,705 | 1,10 | 2,02 |
| Zhang 2018 (n=25) | 25 | 0,000 | 0,000 to 13,719 | 2,05 | 3,11 |
| Total (fixed effects) | 1239 | 1,248 | 0,713 to 2,025 | 100,00 | 100,00 |
| Total (random effects) | 1239 | 1,980 | 0,966 to 3,346 | 100,00 | 100,00 |

## Test for heterogeneity

| Q | 54,1157 |
| --- | --- |
| DF | 27 |
| Significance level | P = 0,0015 |
| I^2^ (inconsistency) | 50,11% |
| 95% CI for I^2^ | 22,82 to 67,75 |

# Meta-analysis: combined fetoscopic reduction minor

| Variable for studies | Study |
| --- | --- |
| Variable for total number of cases | N |
| Variable for number of positive cases | Outcome |

| Study | Sample size | Proportion (%) | 95% CI | Weight (%) | |
| --- | --- | --- | --- | --- | --- |
|  |  |  |  | Fixed | Random |
| Bebbington 2012 (n=146) | 146 | 0,000 | 0,000 to 2,495 | 11,60 | 6,25 |
| Berg 2014 (n=7) | 7 | 0,000 | 0,000 to 40,962 | 0,63 | 1,35 |
| Delabaere 2013 (n=30) | 30 | 3,333 | 0,0844 to 17,217 | 2,45 | 3,50 |
| Deprest 2000 (n=10) | 10 | 0,000 | 0,000 to 30,850 | 0,87 | 1,74 |
| Gallot 2003 (n=11) | 11 | 9,091 | 0,230 to 41,278 | 0,95 | 1,86 |
| Gouverneur 2009 (n=54) | 54 | 0,000 | 0,000 to 6,603 | 4,34 | 4,63 |
| Gul 2008 (n=9) | 9 | 0,000 | 0,000 to 33,627 | 0,79 | 1,62 |
| Has 2014 (n=71) | 71 | 1,408 | 0,0357 to 7,599 | 5,68 | 5,13 |
| He 2010 (n=14) | 14 | 7,143 | 0,181 to 33,868 | 1,18 | 2,20 |
| Ilagan 2008 (n=27) | 27 | 7,407 | 0,910 to 24,290 | 2,21 | 3,31 |
| Jelin 2010 (n=7) | 7 | 14,286 | 0,361 to 57,872 | 0,63 | 1,35 |
| King 2017 (n=43) | 43 | 2,326 | 0,0589 to 12,289 | 3,47 | 4,19 |
| Lanna 2012 (n=118) | 118 | 0,847 | 0,0215 to 4,631 | 9,39 | 5,95 |
| Lee 2013 (n=98) | 98 | 2,041 | 0,248 to 7,178 | 7,81 | 5,67 |
| Lewi 2006 (n=80) | 80 | 0,000 | 0,000 to 4,506 | 6,39 | 5,34 |
| Moise 2008 (n=9) | 9 | 0,000 | 0,000 to 33,627 | 0,79 | 1,62 |
| Nobili 2013 (n=48) | 48 | 0,000 | 0,000 to 7,397 | 3,87 | 4,40 |
| Paramasivam 2010 (n=35) | 35 | 0,000 | 0,000 to 10,003 | 2,84 | 3,80 |
| Peng 2016 (n=93) | 93 | 10,753 | 5,278 to 18,887 | 7,42 | 5,59 |
| Quintero 1996 (n=13) | 13 | 30,769 | 9,092 to 61,426 | 1,10 | 2,09 |
| Quintero 2006 (n=51) | 51 | 5,882 | 1,230 to 16,242 | 4,10 | 4,52 |
| Roman 2010 (n=60) | 60 | 0,000 | 0,000 to 5,963 | 4,81 | 4,82 |
| Schou 2018 (n=102) | 102 | 0,980 | 0,0248 to 5,342 | 8,13 | 5,73 |
| Sugibayashi 2016 (n=40) | 40 | 2,500 | 0,0633 to 13,159 | 3,24 | 4,05 |
| Takano 2015 (n=10) | 10 | 0,000 | 0,000 to 30,850 | 0,87 | 1,74 |
| Taylor 2002 (n=15) | 15 | 6,667 | 0,169 to 31,948 | 1,26 | 2,30 |
| Tsao 2002 (n=13) | 13 | 7,692 | 0,195 to 36,030 | 1,10 | 2,09 |
| Zhang 2018 (n=25) | 25 | 0,000 | 0,000 to 13,719 | 2,05 | 3,16 |
| Total (fixed effects) | 1239 | 2,328 | 1,570 to 3,316 | 100,00 | 100,00 |
| Total (random effects) | 1239 | 2,996 | 1,676 to 4,683 | 100,00 | 100,00 |

## Test for heterogeneity

| Q | 57,5774 |
| --- | --- |
| DF | 27 |
| Significance level | P = 0,0005 |
| I^2^ (inconsistency) | 53,11% |
| 95% CI for I^2^ | 27,90 to 69,50 |

# Meta-analysis: combined fetoscopic reduction all complications

| Variable for studies | Study |
| --- | --- |
| Variable for total number of cases | N |
| Variable for number of positive cases | Outcome |

| Study | Sample size | Proportion (%) | 95% CI | Weight (%) | |
| --- | --- | --- | --- | --- | --- |
|  |  |  |  | Fixed | Random |
| Bebbington 2012 (n=146) | 146 | 0,000 | 0,000 to 2,495 | 11,60 | 5,20 |
| Berg 2014 (n=7) | 7 | 14,286 | 0,361 to 57,872 | 0,63 | 1,80 |
| Delabaere 2013 (n=30) | 30 | 6,667 | 0,818 to 22,074 | 2,45 | 3,70 |
| Deprest 2000 (n=10) | 10 | 10,000 | 0,253 to 44,502 | 0,87 | 2,22 |
| Gallot 2003 (n=11) | 11 | 18,182 | 2,283 to 51,776 | 0,95 | 2,34 |
| Gouverneur 2009 (n=54) | 54 | 0,000 | 0,000 to 6,603 | 4,34 | 4,40 |
| Gul 2008 (n=9) | 9 | 0,000 | 0,000 to 33,627 | 0,79 | 2,09 |
| Has 2014 (n=71) | 71 | 1,408 | 0,0357 to 7,599 | 5,68 | 4,67 |
| He 2010 (n=14) | 14 | 7,143 | 0,181 to 33,868 | 1,18 | 2,66 |
| Ilagan 2008 (n=27) | 27 | 18,519 | 6,300 to 38,083 | 2,21 | 3,56 |
| Jelin 2010 (n=7) | 7 | 14,286 | 0,361 to 57,872 | 0,63 | 1,80 |
| King 2017 (n=43) | 43 | 11,628 | 3,885 to 25,083 | 3,47 | 4,15 |
| Lanna 2012 (n=118) | 118 | 0,847 | 0,0215 to 4,631 | 9,39 | 5,07 |
| Lee 2013 (n=98) | 98 | 2,041 | 0,248 to 7,178 | 7,81 | 4,94 |
| Lewi 2006 (n=80) | 80 | 1,250 | 0,0316 to 6,769 | 6,39 | 4,78 |
| Moise 2008 (n=9) | 9 | 11,111 | 0,281 to 48,250 | 0,79 | 2,09 |
| Nobili 2013 (n=48) | 48 | 0,000 | 0,000 to 7,397 | 3,87 | 4,27 |
| Paramasivam 2010 (n=35) | 35 | 0,000 | 0,000 to 10,003 | 2,84 | 3,90 |
| Peng 2016 (n=93) | 93 | 10,753 | 5,278 to 18,887 | 7,42 | 4,90 |
| Quintero 1996 (n=13) | 13 | 38,462 | 13,858 to 68,422 | 1,10 | 2,56 |
| Quintero 2006 (n=51) | 51 | 11,765 | 4,442 to 23,868 | 4,10 | 4,34 |
| Roman 2010 (n=60) | 60 | 0,000 | 0,000 to 5,963 | 4,81 | 4,51 |
| Schou 2018 (n=102) | 102 | 0,980 | 0,0248 to 5,342 | 8,13 | 4,97 |
| Sugibayashi 2016 (n=40) | 40 | 2,500 | 0,0633 to 13,159 | 3,24 | 4,06 |
| Takano 2015 (n=10) | 10 | 0,000 | 0,000 to 30,850 | 0,87 | 2,22 |
| Taylor 2002 (n=15) | 15 | 20,000 | 4,331 to 48,089 | 1,26 | 2,75 |
| Tsao 2002 (n=13) | 13 | 7,692 | 0,195 to 36,030 | 1,10 | 2,56 |
| Zhang 2018 (n=25) | 25 | 0,000 | 0,000 to 13,719 | 2,05 | 3,46 |
| Total (fixed effects) | 1239 | 3,318 | 2,402 to 4,458 | 100,00 | 100,00 |
| Total (random effects) | 1239 | 5,201 | 3,003 to 7,958 | 100,00 | 100,00 |

## Test for heterogeneity

| Q | 93,3088 |
| --- | --- |
| DF | 27 |
| Significance level | P < 0,0001 |
| I^2^ (inconsistency) | 71,06% |
| 95% CI for I^2^ | 57,65 to 80,23 |

# Meta-analysis: late open subfertility

| Variable for studies | Study |
| --- | --- |
| Variable for total number of cases | N |
| Variable for number of positive cases | Outcome |

| Study | Sample size | Proportion (%) | 95% CI | Weight (%) | |
| --- | --- | --- | --- | --- | --- |
|  |  |  |  | Fixed | Random |
| Farrell 1999 (n=45) | 45 | 0,000 | 0,000 to 7,871 | 21,30 | 23,27 |
| Thom 2016 (n=87) | 87 | 5,747 | 1,892 to 12,904 | 40,74 | 33,94 |
| Wilson 2010 (n=47) | 47 | 2,128 | 0,0539 to 11,294 | 22,22 | 23,92 |
| Zamora 2013 (n=33) | 33 | 6,061 | 0,743 to 20,226 | 15,74 | 18,88 |
| Total (fixed effects) | 212 | 3,968 | 1,789 to 7,508 | 100,00 | 100,00 |
| Total (random effects) | 212 | 3,807 | 1,216 to 7,757 | 100,00 | 100,00 |

## Test for heterogeneity

| Q | 4,7375 |
| --- | --- |
| DF | 3 |
| Significance level | P = 0,1921 |
| I^2^ (inconsistency) | 36,68% |
| 95% CI for I^2^ | 0,00 to 78,14 |

# Meta-analysis: late open miscarriage

| Variable for studies | Study |
| --- | --- |
| Variable for total number of cases | N |
| Variable for number of positive cases | Outcome |

| Study | Sample size | Proportion (%) | 95% CI | Weight (%) | |
| --- | --- | --- | --- | --- | --- |
|  |  |  |  | Fixed | Random |
| Farrell 1999 (n=45) | 51 | 15,686 | 7,024 to 28,588 | 42,62 | 42,62 |
| Thom 2016 (n=21) | 21 | 19,048 | 5,446 to 41,907 | 18,03 | 18,03 |
| Wilson 2010 (n=47) | 47 | 23,404 | 12,303 to 38,026 | 39,34 | 39,34 |
| Total (fixed effects) | 119 | 19,954 | 13,267 to 28,153 | 100,00 | 100,00 |
| Total (random effects) | 119 | 19,954 | 13,371 to 27,480 | 100,00 | 100,00 |

## Test for heterogeneity

| Q | 0,9108 |
| --- | --- |
| DF | 2 |
| Significance level | P = 0,6342 |
| I^2^ (inconsistency) | 0,00% |
| 95% CI for I^2^ | 0,00 to 92,63 |

# Meta-analysis: late fetoscopic miscarriage

| Variable for studies | Study |
| --- | --- |
| Variable for total number of cases | N |
| Variable for number of positive cases | Outcome |

| Study | Sample size | Proportion (%) | 95% CI | Weight (%) | |
| --- | --- | --- | --- | --- | --- |
|  |  |  |  | Fixed | Random |
| Gregoir 2016 (n=89) | 90 | 13,333 | 7,084 to 22,133 | 43,96 | 43,96 |
| Le Lous 2018 (n=122) | 70 | 15,714 | 8,114 to 26,380 | 34,30 | 34,30 |
| Vergote 2018 (n=92) | 44 | 9,091 | 2,533 to 21,669 | 21,74 | 21,74 |
| Total (fixed effects) | 204 | 13,674 | 9,304 to 19,119 | 100,00 | 100,00 |
| Total (random effects) | 204 | 13,674 | 9,344 to 18,676 | 100,00 | 100,00 |

## Test for heterogeneity

| Q | 0,9497 |
| --- | --- |
| DF | 2 |
| Significance level | P = 0,6220 |
| I^2^ (inconsistency) | 0,00% |
| 95% CI for I^2^ | 0,00 to 92,94 |

# Meta-analysis: late open preterm birth

| Variable for studies | Study |
| --- | --- |
| Variable for total number of cases | N |
| Variable for number of positive cases | Outcome |

| Study | Sample size | Proportion (%) | 95% CI | Weight (%) | |
| --- | --- | --- | --- | --- | --- |
|  |  |  |  | Fixed | Random |
| Farrell 1999 (n=45) | 51 | 15,686 | 7,024 to 28,588 | 37,96 | 30,48 |
| Thom 2016 (n=21) | 21 | 42,857 | 21,820 to 65,979 | 16,06 | 21,91 |
| Wilson 2010 (n=47) | 47 | 17,021 | 7,647 to 30,809 | 35,04 | 29,76 |
| Zamora 2013 (n=14) | 14 | 7,143 | 0,181 to 33,868 | 10,95 | 17,85 |
| Total (fixed effects) | 133 | 19,704 | 13,404 to 27,359 | 100,00 | 100,00 |
| Total (random effects) | 133 | 20,493 | 10,480 to 32,807 | 100,00 | 100,00 |

## Test for heterogeneity

| Q | 7,5575 |
| --- | --- |
| DF | 3 |
| Significance level | P = 0,0561 |
| I^2^ (inconsistency) | 60,30% |
| 95% CI for I^2^ | 0,00 to 86,74 |

# Meta-analysis: late fetoscopic preterm birth

| Variable for studies | Study |
| --- | --- |
| Variable for total number of cases | N |
| Variable for number of positive cases | Outcome |

| Study | Sample size | Proportion (%) | 95% CI | Weight (%) | |
| --- | --- | --- | --- | --- | --- |
|  |  |  |  | Fixed | Random |
| Gregoir 2016 (n=89) | 90 | 7,778 | 3,184 to 15,371 | 43,96 | 35,35 |
| Le Lous 2018 (n=122) | 70 | 0,000 | 0,000 to 5,133 | 34,30 | 33,94 |
| Vergote 2018 (n=92) | 44 | 0,000 | 0,000 to 8,042 | 21,74 | 30,71 |
| Total (fixed effects) | 204 | 2,685 | 0,944 to 5,914 | 100,00 | 100,00 |
| Total (random effects) | 204 | 2,118 | 0,0163 to 9,009 | 100,00 | 100,00 |

## Test for heterogeneity

| Q | 10,4078 |
| --- | --- |
| DF | 2 |
| Significance level | P = 0,0055 |
| I^2^ (inconsistency) | 80,78% |
| 95% CI for I^2^ | 39,78 to 93,87 |

# Meta-analysis: late open rupture

| Variable for studies | Study |
| --- | --- |
| Variable for total number of cases | N |
| Variable for number of positive cases | Outcome |

| Study | Sample size | Proportion (%) | 95% CI | Weight (%) | |
| --- | --- | --- | --- | --- | --- |
|  |  |  |  | Fixed | Random |
| Thom 2016 (n=21) | 21 | 0,000 | 0,000 to 16,110 | 25,88 | 30,67 |
| Wilson 2010 (n=47) | 47 | 10,638 | 3,546 to 23,105 | 56,47 | 45,35 |
| Zamora 2013 (n=14) | 14 | 7,143 | 0,181 to 33,868 | 17,65 | 23,98 |
| Total (fixed effects) | 82 | 7,566 | 2,949 to 15,384 | 100,00 | 100,00 |
| Total (random effects) | 82 | 6,887 | 1,337 to 16,271 | 100,00 | 100,00 |

## Test for heterogeneity

| Q | 3,5157 |
| --- | --- |
| DF | 2 |
| Significance level | P = 0,1724 |
| I^2^ (inconsistency) | 43,11% |
| 95% CI for I^2^ | 0,00 to 82,90 |

# Meta-analysis: late open dehiscence

| Variable for studies | Study |
| --- | --- |
| Variable for total number of cases | N |
| Variable for number of positive cases | Outcome |

| Study | Sample size | Proportion (%) | 95% CI | Weight (%) | |
| --- | --- | --- | --- | --- | --- |
|  |  |  |  | Fixed | Random |
| Thom 2016 (n=21) | 21 | 9,524 | 1,175 to 30,377 | 25,88 | 25,88 |
| Wilson 2010 (n=47) | 47 | 10,638 | 3,546 to 23,105 | 56,47 | 56,47 |
| Zamora 2013 (n=14) | 14 | 7,143 | 0,181 to 33,868 | 17,65 | 17,65 |
| Total (fixed effects) | 82 | 11,086 | 5,306 to 19,755 | 100,00 | 100,00 |
| Total (random effects) | 82 | 11,086 | 5,337 to 18,586 | 100,00 | 100,00 |

## Test for heterogeneity

| Q | 0,03558 |
| --- | --- |
| DF | 2 |
| Significance level | P = 0,9824 |
| I^2^ (inconsistency) | 0,00% |
| 95% CI for I^2^ | 0,00 to 0,00 |

# Meta-analysis: late open attempting pregnancy

| Variable for studies | Study |
| --- | --- |
| Variable for total number of cases | N |
| Variable for number of positive cases | Outcome |

| Study | Sample size | Proportion (%) | 95% CI | Weight (%) | |
| --- | --- | --- | --- | --- | --- |
|  |  |  |  | Fixed | Random |
| Farrell 1999 (n=45) | 45 | 77,778 | 62,911 to 88,795 | 27,38 | 33,21 |
| Thom 2016 (n=87) | 87 | 29,885 | 20,538 to 40,649 | 52,38 | 34,44 |
| Zamora 2013 (n=33) | 33 | 42,424 | 25,476 to 60,785 | 20,24 | 32,35 |
| Total (fixed effects) | 165 | 45,667 | 37,973 to 53,515 | 100,00 | 100,00 |
| Total (random effects) | 165 | 50,111 | 21,552 to 78,629 | 100,00 | 100,00 |

## Test for heterogeneity

| Q | 29,3999 |
| --- | --- |
| DF | 2 |
| Significance level | P < 0,0001 |
| I^2^ (inconsistency) | 93,20% |
| 95% CI for I^2^ | 83,48 to 97,20 |

# Meta-analysis: late fetoscopic attempting pregnancy

| Variable for studies | Study |
| --- | --- |
| Variable for total number of cases | N |
| Variable for number of positive cases | Outcome |

| Study | Sample size | Proportion (%) | 95% CI | Weight (%) | |
| --- | --- | --- | --- | --- | --- |
|  |  |  |  | Fixed | Random |
| Gregoir 2016 (n=89) | 89 | 69,663 | 59,008 to 78,965 | 49,18 | 49,97 |
| Vergote 2018 (n=92) | 92 | 33,696 | 24,170 to 44,304 | 50,82 | 50,03 |
| Total (fixed effects) | 181 | 51,476 | 43,989 to 58,914 | 100,00 | 100,00 |
| Total (random effects) | 181 | 51,762 | 18,633 to 84,031 | 100,00 | 100,00 |

## Test for heterogeneity

| Q | 24,2358 |
| --- | --- |
| DF | 1 |
| Significance level | P < 0,0001 |
| I^2^ (inconsistency) | 95,87% |
| 95% CI for I^2^ | 88,23 to 98,55 |

# Meta-analysis: late open achieving pregnancy

| Variable for studies | Study |
| --- | --- |
| Variable for total number of cases | N |
| Variable for number of positive cases | Outcome |

| Study | Sample size | Proportion (%) | 95% CI | Weight (%) | |
| --- | --- | --- | --- | --- | --- |
|  |  |  |  | Fixed | Random |
| Farrell 1999 (n=45) | 45 | 71,111 | 55,685 to 83,634 | 21,30 | 24,87 |
| Thom 2016 (n=87) | 87 | 24,138 | 15,605 to 34,500 | 40,74 | 26,15 |
| Wilson 2010 (n=47) | 47 | 57,447 | 42,178 to 71,742 | 22,22 | 24,98 |
| Zamora 2013 (n=33) | 33 | 42,424 | 25,476 to 60,785 | 15,74 | 24,00 |
| Total (fixed effects) | 212 | 44,078 | 37,348 to 50,973 | 100,00 | 100,00 |
| Total (random effects) | 212 | 48,334 | 26,741 to 70,260 | 100,00 | 100,00 |

## Test for heterogeneity

| Q | 32,4305 |
| --- | --- |
| DF | 3 |
| Significance level | P < 0,0001 |
| I^2^ (inconsistency) | 90,75% |
| 95% CI for I^2^ | 79,37 to 95,85 |

# Meta-analysis: late fetoscopic achieving pregnancy

| Variable for studies | Study |
| --- | --- |
| Variable for total number of cases | N |
| Variable for number of positive cases | Outcome |

| Study | Sample size | Proportion (%) | 95% CI | Weight (%) | |
| --- | --- | --- | --- | --- | --- |
|  |  |  |  | Fixed | Random |
| Gregoir 2016 (n=89) | 89 | 65,169 | 54,332 to 74,964 | 29,41 | 32,93 |
| Le Lous 2018 (n=122) | 122 | 45,902 | 36,848 to 55,159 | 40,20 | 34,01 |
| Vergote 2018 (n=92) | 92 | 33,696 | 24,170 to 44,304 | 30,39 | 33,06 |
| Total (fixed effects) | 303 | 47,854 | 42,138 to 53,612 | 100,00 | 100,00 |
| Total (random effects) | 303 | 48,204 | 31,455 to 65,163 | 100,00 | 100,00 |

## Test for heterogeneity

| Q | 18,6420 |
| --- | --- |
| DF | 2 |
| Significance level | P = 0,0001 |
| I^2^ (inconsistency) | 89,27% |
| 95% CI for I^2^ | 70,83 to 96,05 |

# Meta-analysis: late open bleeding delivery

| Variable for studies | Study |
| --- | --- |
| Variable for total number of cases | N |
| Variable for number of positive cases | Outcome |

| Study | Sample size | Proportion (%) | 95% CI | Weight (%) | |
| --- | --- | --- | --- | --- | --- |
|  |  |  |  | Fixed | Random |
| Thom 2016 (n=21) | 21 | 9,524 | 1,175 to 30,377 | 31,43 | 31,43 |
| Wilson 2010 (n=47) | 47 | 4,255 | 0,520 to 14,541 | 68,57 | 68,57 |
| Total (fixed effects) | 68 | 6,843 | 2,190 to 15,495 | 100,00 | 100,00 |
| Total (random effects) | 68 | 6,843 | 2,161 to 13,883 | 100,00 | 100,00 |

## Test for heterogeneity

| Q | 0,7723 |
| --- | --- |
| DF | 1 |
| Significance level | P = 0,3795 |
| I^2^ (inconsistency) | 0,00% |
| 95% CI for I^2^ | 0,00 to 0,00 |

# Meta-analysis: late fetoscopic bleeding delivery

| Variable for studies | Study |
| --- | --- |
| Variable for total number of cases | N |
| Variable for number of positive cases | Outcome |

| Study | Sample size | Proportion (%) | 95% CI | Weight (%) | |
| --- | --- | --- | --- | --- | --- |
|  |  |  |  | Fixed | Random |
| Gregoir 2016 (n=90) | 90 | 4,444 | 1,224 to 10,990 | 43,96 | 43,96 |
| Le Lous 2018 (n=70) | 70 | 5,714 | 1,579 to 13,989 | 34,30 | 34,30 |
| Vergote 2018 (n=44) | 44 | 4,545 | 0,555 to 15,473 | 21,74 | 21,74 |
| Total (fixed effects) | 204 | 5,516 | 2,827 to 9,560 | 100,00 | 100,00 |
| Total (random effects) | 204 | 5,516 | 2,828 to 9,028 | 100,00 | 100,00 |

## Test for heterogeneity

| Q | 0,1455 |
| --- | --- |
| DF | 2 |
| Significance level | P = 0,9298 |
| I^2^ (inconsistency) | 0,00% |
| 95% CI for I^2^ | 0,00 to 53,90 |

# Meta-analysis: late fetoscopic abdominal pain

| Variable for studies | Study |
| --- | --- |
| Variable for total number of cases | N |
| Variable for number of positive cases | Outcome |

| Study | Sample size | Proportion (%) | 95% CI | Weight (%) | |
| --- | --- | --- | --- | --- | --- |
|  |  |  |  | Fixed | Random |
| Gregoir 2016 (n=89) | 89 | 5,618 | 1,849 to 12,625 | 49,18 | 49,63 |
| Vergote 2018 (n=92) | 92 | 11,957 | 6,122 to 20,387 | 50,82 | 50,37 |
| Total (fixed effects) | 181 | 9,035 | 5,306 to 14,156 | 100,00 | 100,00 |
| Total (random effects) | 181 | 9,007 | 3,843 to 16,059 | 100,00 | 100,00 |

## Test for heterogeneity

| Q | 2,2018 |
| --- | --- |
| DF | 1 |
| Significance level | P = 0,1378 |
| I^2^ (inconsistency) | 54,58% |
| 95% CI for I^2^ | 0,00 to 88,89 |

# Meta-analysis: late fetoscopic gynae bleeding

| Variable for studies | Study |
| --- | --- |
| Variable for total number of cases | N |
| Variable for number of positive cases | Outcome |

| Study | Sample size | Proportion (%) | 95% CI | Weight (%) | |
| --- | --- | --- | --- | --- | --- |
|  |  |  |  | Fixed | Random |
| Gregoir 2016 (n=89) | 89 | 5,618 | 1,849 to 12,625 | 49,18 | 49,18 |
| Vergote 2018 (n=92) | 92 | 6,522 | 2,431 to 13,656 | 50,82 | 50,82 |
| Total (fixed effects) | 181 | 6,540 | 3,421 to 11,153 | 100,00 | 100,00 |
| Total (random effects) | 181 | 6,540 | 3,426 to 10,565 | 100,00 | 100,00 |

## Test for heterogeneity

| Q | 0,05807 |
| --- | --- |
| DF | 1 |
| Significance level | P = 0,8096 |
| I^2^ (inconsistency) | 0,00% |
| 95% CI for I^2^ | 0,00 to 0,00 |

# Meta-analysis: late open gynae surgery

| Variable for studies | Study |
| --- | --- |
| Variable for total number of cases | N |
| Variable for number of positive cases | Outcome |

| Study | Sample size | Proportion (%) | 95% CI | Weight (%) | |
| --- | --- | --- | --- | --- | --- |
|  |  |  |  | Fixed | Random |
| Wilson 2010 (n=47) | 47 | 12,766 | 4,832 to 25,741 | 58,54 | 53,80 |
| Zamora 2013 (n=33) | 33 | 3,030 | 0,0767 to 15,759 | 41,46 | 46,20 |
| Total (fixed effects) | 80 | 9,131 | 3,890 to 17,545 | 100,00 | 100,00 |
| Total (random effects) | 80 | 8,678 | 1,809 to 19,958 | 100,00 | 100,00 |

## Test for heterogeneity

| Q | 2,2452 |
| --- | --- |
| DF | 1 |
| Significance level | P = 0,1340 |
| I^2^ (inconsistency) | 55,46% |
| 95% CI for I^2^ | 0,00 to 89,20 |

# Meta-analysis: late fetoscopic psychological

| Variable for studies | Study |
| --- | --- |
| Variable for total number of cases | N |
| Variable for number of positive cases | Outcome |

| Study | Sample size | Proportion (%) | 95% CI | Weight (%) | |
| --- | --- | --- | --- | --- | --- |
|  |  |  |  | Fixed | Random |
| Le Lous 2018 (n=122) | 122 | 18,033 | 11,660 to 26,019 | 56,94 | 50,30 |
| Vergote 2018 (n=92) | 92 | 48,913 | 38,341 to 59,557 | 43,06 | 49,70 |
| Total (fixed effects) | 214 | 30,509 | 24,443 to 37,119 | 100,00 | 100,00 |
| Total (random effects) | 214 | 32,564 | 7,703 to 64,581 | 100,00 | 100,00 |

## Test for heterogeneity

| Q | 23,4506 |
| --- | --- |
| DF | 1 |
| Significance level | P < 0,0001 |
| I^2^ (inconsistency) | 95,74% |
| 95% CI for I^2^ | 87,72 to 98,52 |
